# Ring Opening of Triflates Derived from Benzophospholan-3-one Oxides by Aryl Grignard Reagents as a Route to 2-Ethynylphenyl(diaryl)phosphine Oxides

**DOI:** 10.1021/acs.joc.1c01629

**Published:** 2021-10-26

**Authors:** Łukasz Ponikiewski, Sylwia Sowa

**Affiliations:** † Department of Inorganic Chemistry, Faculty of Chemistry, Gdańsk University of Technology, G. Narutowicza St. 11/12, Gdańsk PL-80-233, Poland; ‡ Department of Organic Chemistry, Faculty of Chemistry, Institute of Chemical Sciences, Marie Curie-Sklodowska University in Lublin, 33 Gliniana Street, Lublin PL-20-614, Poland

## Abstract

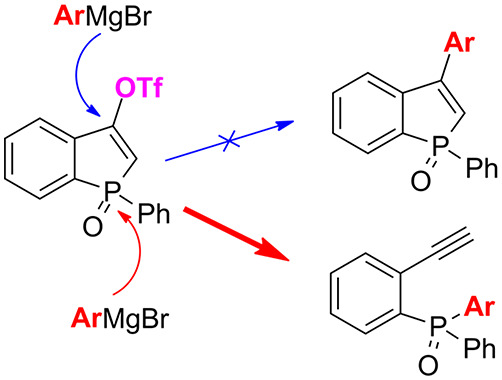

A new simple method for the synthesis of 2-ethynylphenyl(diaryl)phosphine oxides via ring opening of benzophosphol-3-yl triflates has been developed. This process occurs via nucleophilic attack of a Grignard reagent at the phosphorus center, which results in ring opening and cleavage of a leaving group. The reaction proceeds under mild conditions and, within 15–60 min, leads to a library of previously unavailable 2-ethynylphenylphosphine oxides in yields up to 98%.

## Introduction

Compounds possessing an ethynyl fragment became an attractive pattern in modern organic chemistry.^1^ The growing importance of the chemistry of carbon–carbon triple bonds is due to their unique and diverse chemical reactivity and remarkable synthetic potential as they can be used as precursors for many classes of compounds including alkanes, alkenes, aldehydes, and carboxylic acids on both laboratory and industrial scales.^1,2^ Recently, ethynyl compounds have been intensively explored in catalytic reactions mediated by different metals (gold,^3^ copper,^4^ palladium,^5^ rhodium,^6^ and others^7^), providing various cyclic products. Moreover, ethynyl compounds have a wide range of applications in the synthesis of biologically active compounds and have been broadly exploited in drug discovery and development.^8^ Furthermore, an alkyne functionality is frequently used in medicine^9^ and chemical biology^10^ as a “click” probe to label or identify molecular targets and to assess target engagement. The “click-type” reactions of ethynyl compounds have also been exploited to synthesize π-conjugated macromolecules for organic electronics^11^ and other materials.^12^ On the other hand, ethynyl compounds themselves, especially those possessing expanded π-systems, have been involved in the preparation of organic structures for photo-optical purposes and others.^13^ All mentioned applications of alkynes benefit from the rapid development of the synthetic routes, enabling the incorporation of an ethynyl unit into the structure of nearly any organic molecule. The most explored methods here are Sonogashira coupling,^14^ copper-free Sonogashira reactions,^15^ and alkynylation reaction.^16^

**Scheme 1:**
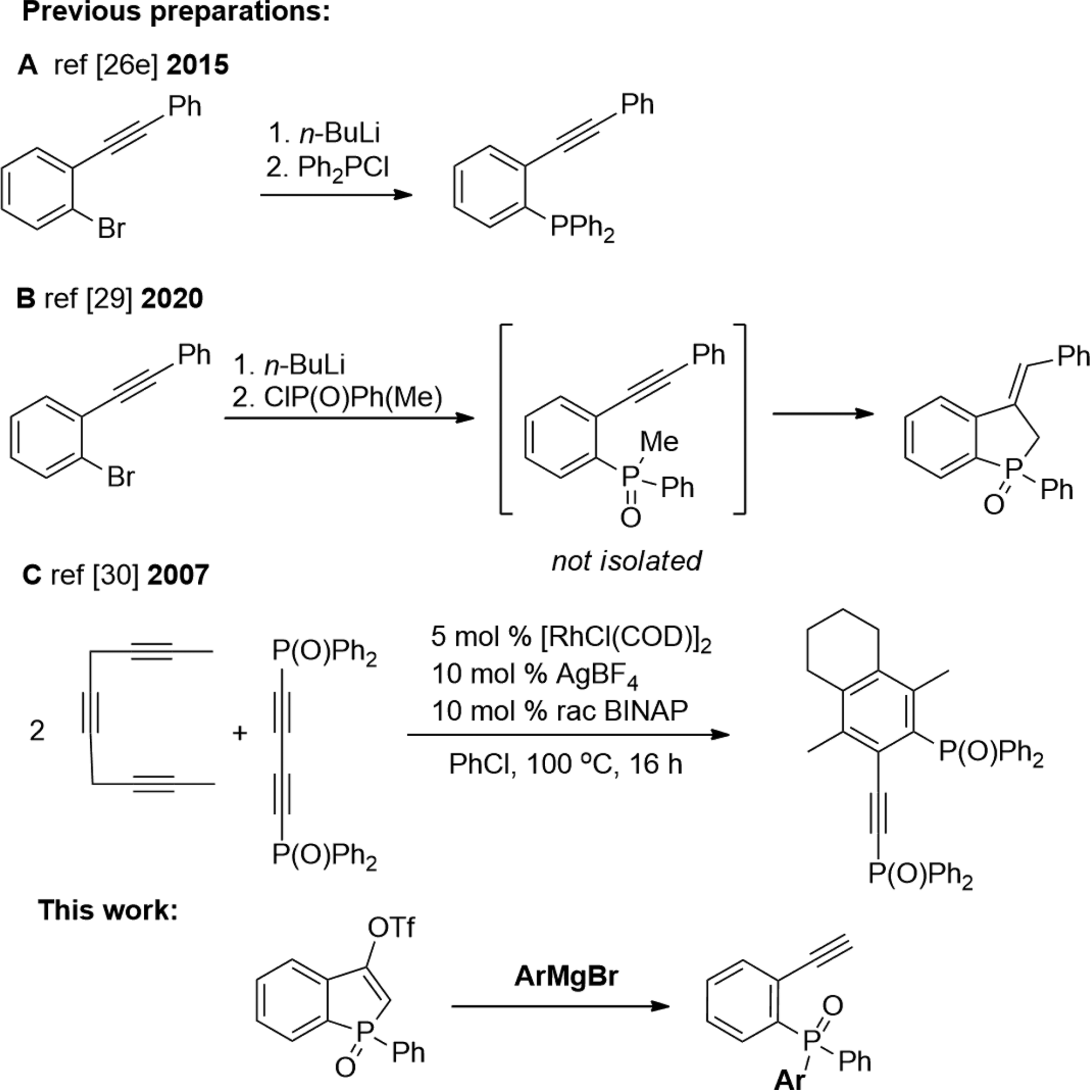
Examples of the Synthesis of 2-Ethynylphenylphosphine Derivatives

In the field of organophosphorus chemistry, (ethynyl)phosphine oxides were employed in the preparations of so-called “smart” polymers^17^ but also as the starting materials for metal-catalyzed coupling,^18^ cyclization,^19^ cycloaddition,^20^ and radical addition/cycloaddition.^21^ Another class of organophosphorus-possessing C–C triple bond compounds mentioned in the literature is *p*-ethynylphenylphosphine oxides. Those compounds have found several applications in the construction of star-shaped fluorescent molecules,^22^ microporous organic polymers (MOPs),^23^ nanostructured carbon materials,^24^ and triazole precursors.^25^ Their *ortho* analogues with phosphorus at a lower oxidation state are typically used for the construction of the benzophosphole skeleton.^26^ The access to those derivatives was achieved via a reaction of *o-*lithiotolane with chlorophosphine (Scheme 1A).^26e,27^ A different route to these derivatives was completed through Sonogashira coupling.^28^ In 2020, Duan et al. showed that methyl(phenyl)phosphinic chloride in a reaction with lithium *o*-phenylethynylbenzene had provided *o*-phenylethynylphosphine oxide, which is immediately transformed into the corresponding benzophosphole oxide (Scheme 1B).^29^ Another compound possessing an ethynyl moiety in the *ortho* position was obtained via [2 + 2 + 2] cyclization (Scheme 1, C).^30^ To the best of our knowledge, the phosphine oxides possessing a terminal ethynyl moiety in the *ortho* position are unknown. The closest known analogues are derivatives of (*o*-ethynylphenyl)phosphonic acid synthesized by Ding^31^ and later Peng.^32^

## Results and Discussion

Herein, we want to present a new route to 2-ethynylphenyl(diaryl)phosphine oxides via unprecedented ring opening of triflates derived from benzophospholan-3-one oxides.

**Scheme 2:**
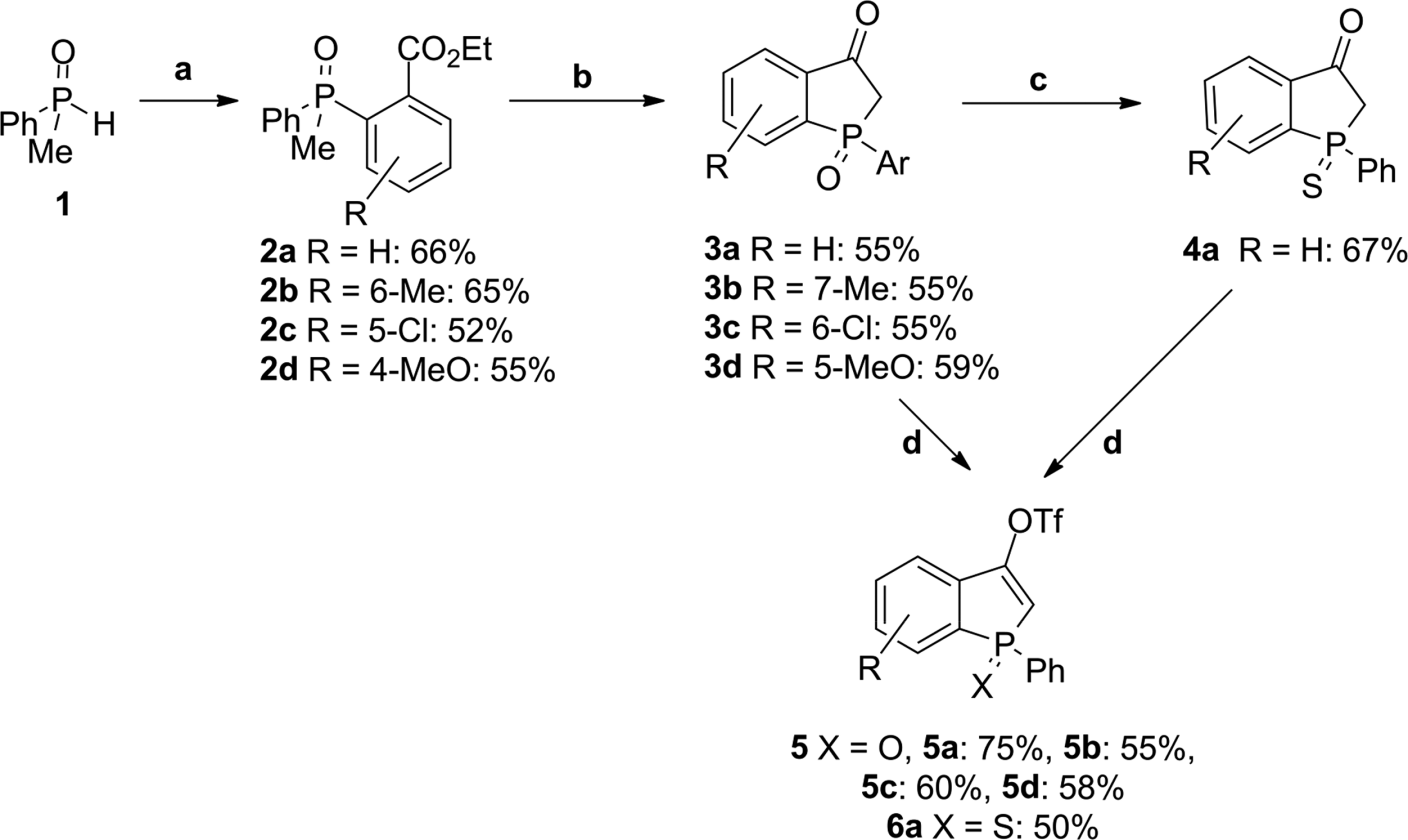
Synthetic Route to Triflates **5** and **6**

We began our study with the preparation of a set of triflates **5** and **6** from benzophospholan-3-one oxides **3** and benzophospholan-3-one sulfide **4a** (Scheme 2, path d). Benzophospholan-3-one oxides **3** and sulfide **4a**, which were starting materials for triflates **5** and **6a**, were prepared in a two-step procedure, utilizing Ullman type coupling of phosphine oxide **1** followed by cyclization in the presence of LDA (Scheme 2, paths a and b). The preparation of benzophosphole sulfide **4a** was achieved via chemoselective reduction of P=O in **3** followed by oxidation of the phosphorus center with sulfur (Scheme 2, path c).

**Scheme 3:**
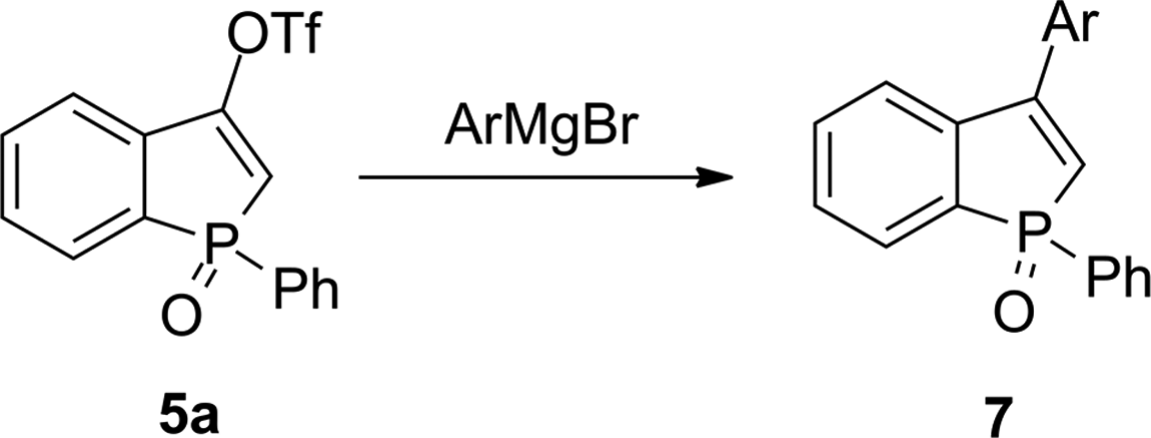
Predicted Outcome of the Reaction of **5a** with Aryl Grignard Reagents

Our primary synthetic goal was to investigate the reactivity of triflates **5** and **6a** toward aryl Grignard reagents in order to prepare 3-arylbenzophosphole oxides **7** according to the reaction depicted in Scheme 3.

In order to check the reactivity of triflates toward the Grignard reagent, the model triflate **5a** was reacted with *p*-tolylmagnesium bromide (Table 1). To our surprise, instead of the expected 1-phenyl-3-(*p*-tolyl)benzophosphole oxide (**7a**), we have observed compound **8a** (as the major product), which, in most cases, remained in the mixture with diphosphorus compound **9** (Table 1, entries 1–5). We realized that the aryl Grignard reagent caused the ring opening of the starting triflate **5a**, which resulted in the formation of phosphine oxide **8a** bearing a 2-ethynylphenyl substituent. Therefore, we decided to focus on the selective transformation of triflates into phosphine oxides **8** possessing a terminal ethynyl group in the *ortho* position, which gives an opportunity for further functionalization.

**Table 1:**
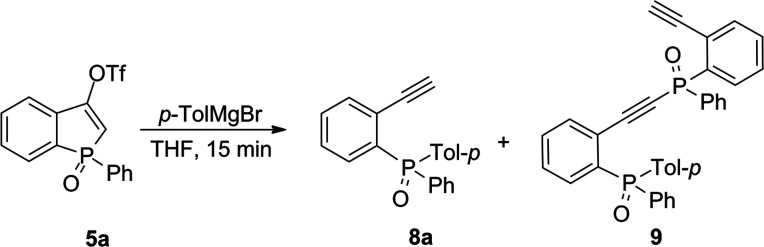
Optimization of Reaction Conditions in a Reaction of **5a** and *p*-TolMgBr^a^

entry	**5a**/*p*-TolMgX (equiv)	temp (°C)	**8a** (%)^b^	**9** (%)^b^
1	1:1.1	–78	36	29^c^
2	1:1.1	–25	71	17
3	1:1.1	rt	76	7
4	1:/1.1	0	65	10
5	1:1.8	0	83	10
6	1:2.5	0	83	0
7^d^	1:1.5	0	98	0

a Reaction conditions: **5a** (0.13 mmol), ArMgBr (0.195 mmol), THF (2 mL).

b Isolated yields.

c Starting material **5a** and 1-phenylbenzophospholan-3-one oxide (**3a**) were present in the crude reaction mixture.

d Reaction was carried out for 30 min.

**Figure 1:**
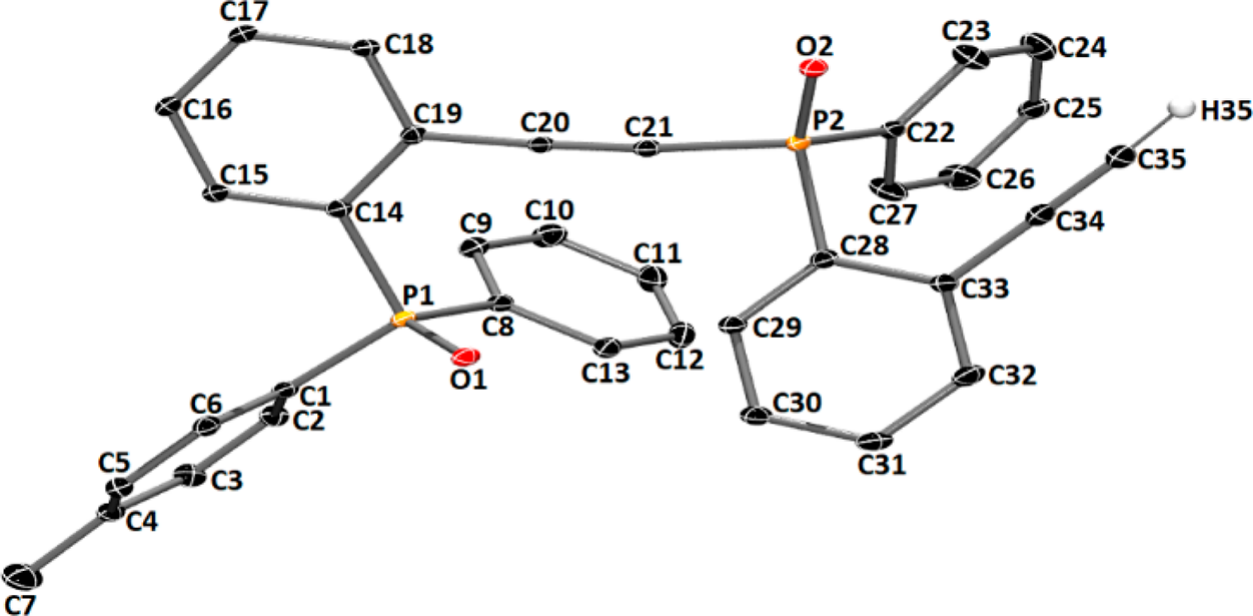
Molecular structure of **9**; ellipsoids 30%, hydrogen atoms have been omitted for clarity (except H35). Important bond distances (Å), angles (deg) for **9**: P1–O1 1.488(2), P2–O2 1.482(2), C20–C21 1.202(4), C34–C35 1.205(6), C35–H35 0.950(2), O1–P1–C1 111.41(14), O1–P1–C8 113.03(15), O1–P1–C14 113.89(15), C20–C21–P2 168.8(3), O2–P2–C22 113.86(16), O2–P2–C28 115.37(15), C33–C34–C35 176.0(3).

First, we screened the influence of the temperature on the reaction of triflate **5a** with *p*-TolMgBr (Table 1, entries 1–4). Preliminary attempts showed that the selectivity of the reaction is significantly affected by temperature. When the reaction of **5a** was carried out at −78 °C, a mixture of products was observed in the crude reaction mixture, and the conversion of **5a** was not complete (Table 1, entry 1). The mixture consisted of **8a**, **9**, and unreacted **5a** and was accompanied by 1-phenylbenzophospholan-3-one oxide (**3a**). We were able to isolate major product **8a** along with diphosphorus compound **9** in 36% and 29% yields, respectively. Under these conditions, selectivity was low, and a relatively high amount of **9** was formed. The structure of compound **9** was confirmed by X-ray analysis (Figure 1). It turned out that compound **9** possesses an alkyne moiety directly connected with the phosphorus atom.

Further attempts have revealed that the full conversion of **5a** can be achieved by increasing the temperature (Table 1, entry 2). Additionally, the amount of **8a** has significantly increased at −25 °C, 0 °C, and rt to 71, 65, and 76% isolated yields, respectively (Table 1, entries 2–4). However, some amounts of **9** have still been found in the reaction mixture and were isolated in 7–17% yields. To optimize the conditions, we decided to maintain the temperature of the reaction at 0 °C. A further study has shown that the change in the **5a**/*p*-TolMgBr ratio strongly affected the outcome of the reaction (Table 1, entries 4–6). In the case where **5a** was reacted with 2.5 equiv of the Grignard reagent, desired **8a** was formed as a sole product isolated in 83% yield (Table 1, entry 6). Finally, the optimal reaction conditions were established by reducing the amount of *p*-TolMgBr and extending the reaction time (Table 1, entry 7). In this case, the complete conversion of **5a** to **8a** was observed, and **8a** was isolated with the highest yield (98%).

In the next stage, the reactivity of triflate **5a** toward a set of aryl Grignard reagents was checked under the optimized reaction conditions (Table 2). In general, triflate **5a** was tolerant toward different substitution patterns in the aryl ring of the Grignard reagent used. In most cases, products **8a**–**k** were obtained in good to very good yields. In the case of tolyl Grignard reagents, there was no difference between the reactivity of the *p*- and *m*-tolyl Grignard reagent, and both led to the formation of **8a** and **8b** in 98% and 84% yields, respectively. In sharp contrast to these reactions, the analogous reaction with *o*-TolMgCl was much less selective and resulted in a mixture of **8c** and ketone **3a**. In this case, product **8c** was formed with the lowest yield (38%). Noteworthy, the reaction with *p*-TolMgBr on a higher scale (0.6 mmol) has also provided desired product **8a** in a high yield (91%).

**Table 2:**
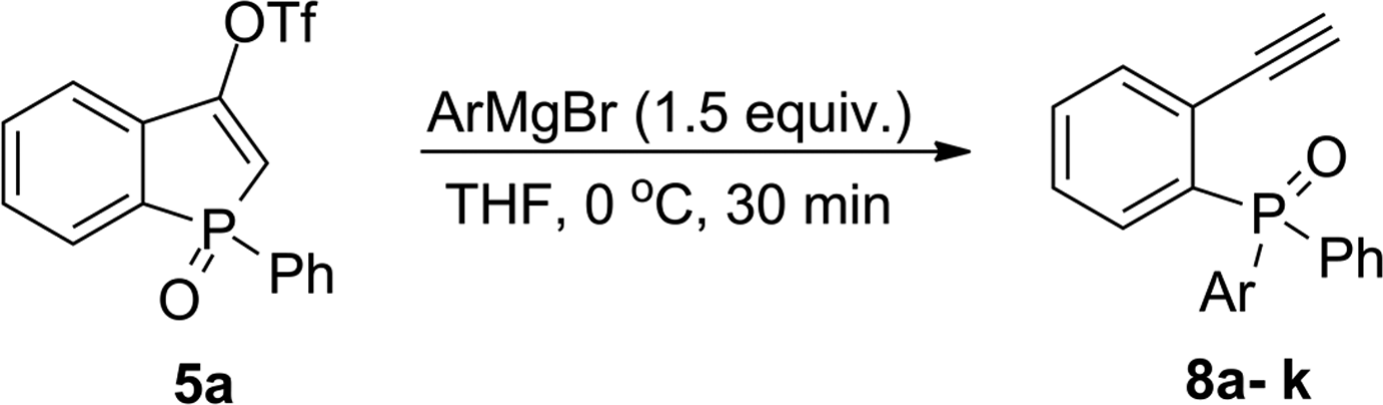

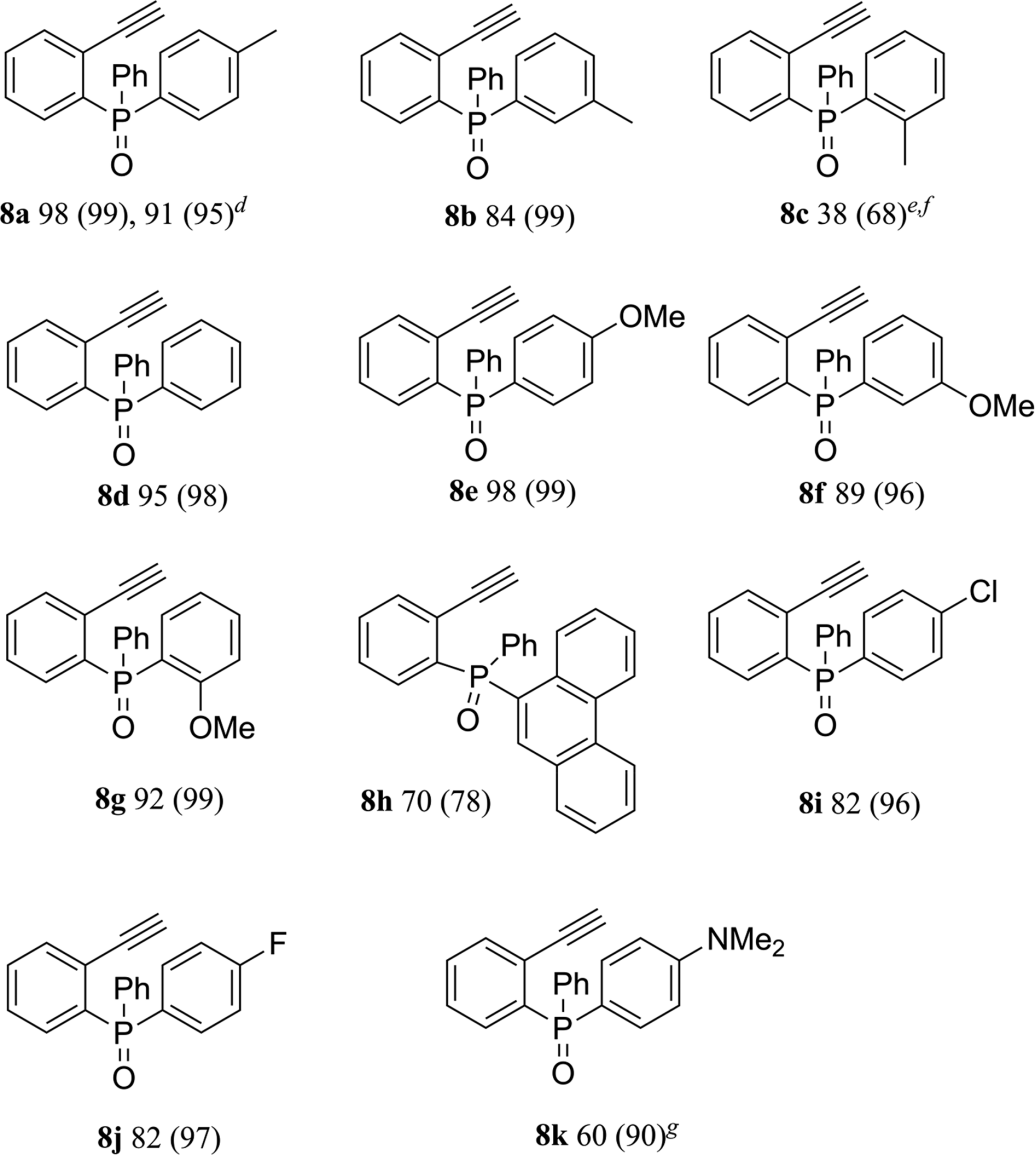
Scope of Different ArMgBr Reagents in the Reaction with Triflate **5a**^a^^,^^b^^,^^c^

a Reaction conditions: **5a** (0.13 mmol), ArMgBr (0.195 mmol), THF (2 mL).

b Isolated yields of product.

c Numbers in parentheses indicate yields according to ^31^P NMR.

d Reaction was carried out on a 0.6 mmol scale.

e Reaction was carried out with *o*-TolMgCl.

f Formation of a small amount of ketone **3a** was observed but not isolated.

g Additionally, 7% of **3a** was isolated.

At the same time, the reactivity of triflate **5a** toward the Grignard reagent possessing electro-donating substituents (anisylmagnesium bromides) was not greatly dependent on the position of the methoxyl group in the phenyl ring. The reactions of compound **5a** with any isomer (*o*-, *m*-, *p*-) of anisylmagnesium bromide provided products **8d**–**f** with 89–98% yields. Another Grignard reagent with an electron-donating substituent, *p*-Me_2_N–C_6_H_4_MgBr, in a reaction with **5a** has provided a mixture of **8k** and **3a**, which was enriched in **8k**. In this case, **8k** and **3a** were isolated in 60 and 7% yields, respectively.

Interestingly, even Grignard reagents possessing halogen atoms in the *para* position in the phenyl ring formed the desired products with a very high selectivity; however, isolated yields of **8i** and **8j** were slightly lower (82% yields for both reagents). In turn, a reaction of **5a** under the same conditions with bulky 9-phenantrylmagnesium bromide has led to product **8h** in 70% yield. Small amounts of other unidentified compounds were also observed but not isolated.

The results achieved for **5a** (Table 2) have prompted us to further investigate the selectivity of this process by changing the substituents in compound **5**. The obtained results are presented in Table 3. To our delight, once again, we were able to isolate the expected 2-ethynylphenyl(diaryl)phosphine oxides **10**–**12** in very good yields. In the case of compounds with electron-donating substituents in benzophosphole rings **5b** and **5d**, a reaction with PhMgBr afforded **10d** and **12d** in high yields −91 and 82%, respectively. Similar results were obtained when compound **5c** possessing a chlorine substituent was subjected to the reaction with PhMgBr. Product **11d** was successfully isolated in a 73% yield. Moreover, the reactions of **5b** with other Grignard reagents (*p*-TolMgBr, *p*-AnMgBr, *p*-Cl–C_6_H_4_MgBr) provided **10–11a**,**e**,**i** as the major products in the crude reaction mixture. Therefore, we assume that the studied reaction is tolerant toward differently substituted aryl Grignard reagents. Unfortunately, the isolation step was less successful in comparison to products formed in reactions with PhMgBr (**10d**, **11d**, **12d**). In these cases, we noted lower isolated yields in the range of 58–66%. Faster isolation of **10**–**12** (higher polarity of the eluent, short chromatography column) resulted in higher yields and moderate purity. In opposite, when compounds were eluted slower (lower polarity of eluent, longer chromatography column), higher purity was noted with a significant reduction of yield. For instance, when isolating **10a** in a lower polarity solvent and a higher polarity solvent, we observed 38% and 66% yields, respectively. It seems that a terminal acetylene moiety can interact with acidic silica gel, which causes a decrease in yield. Unfortunately, some loss during isolation was also observed for products **10k**, **11k**, and **12k** possessing an amino moiety. Even the addition of triethylamine to the eluent during the chromatography column was not beneficial, and all of these products were isolated in a moderate yield, 60–70%.

**Table 3:**
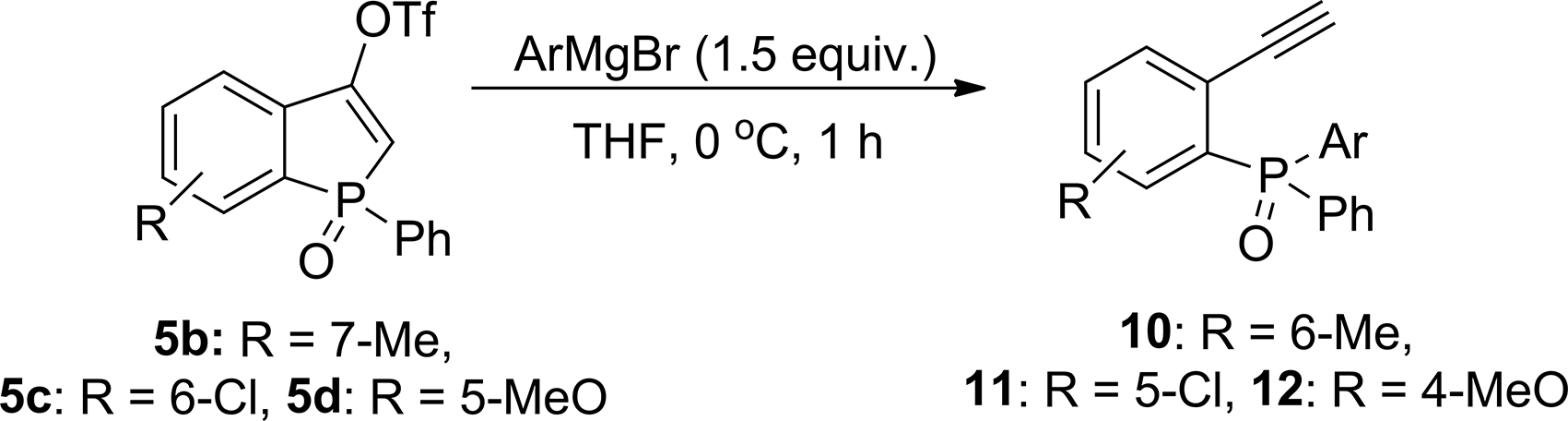

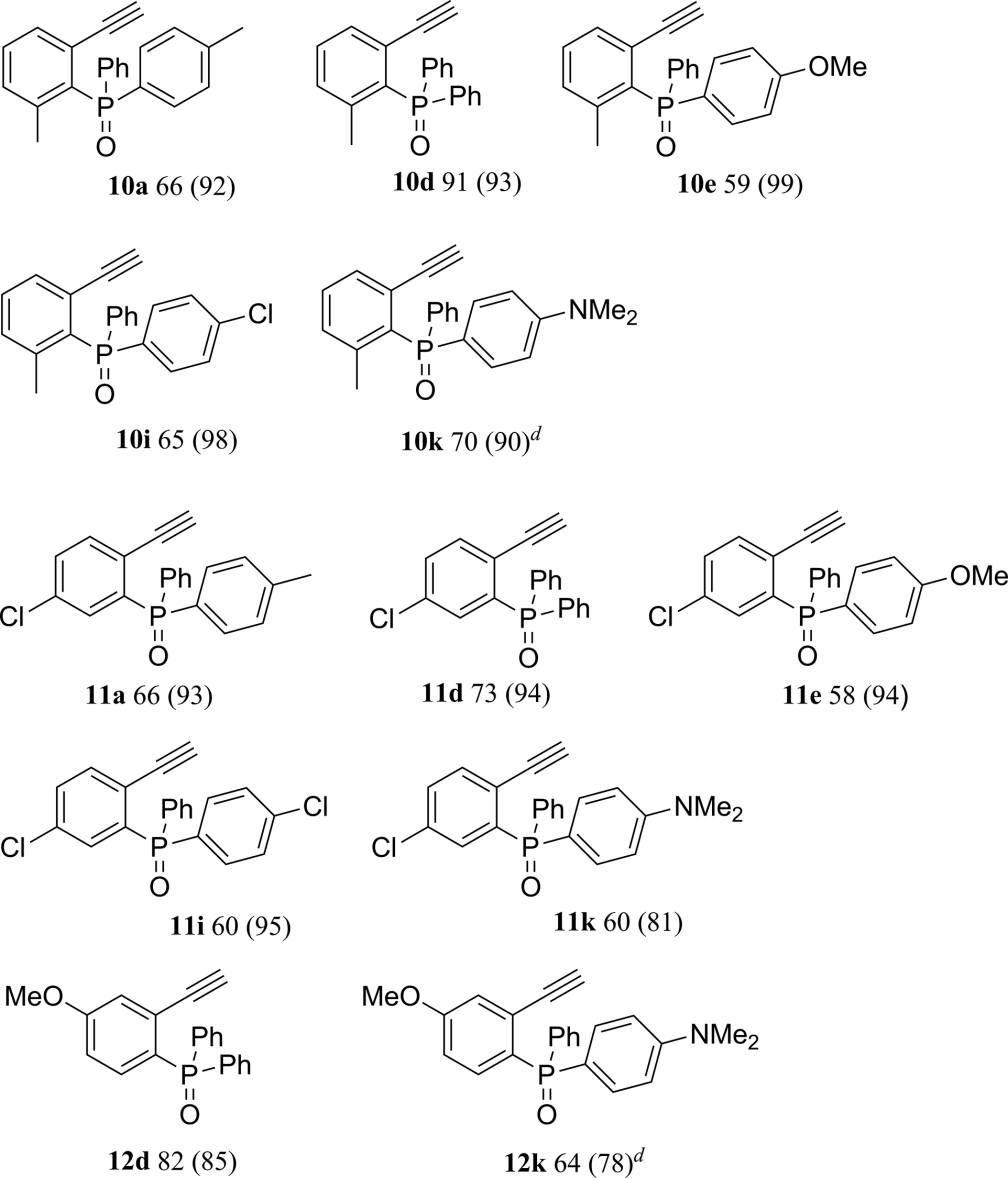
Reactivity of Substituted Benzophosphole Oxides **5b**–**d** with Aryl Grignard Reagents^a^^,^^b^^,^^c^

a Reaction conditions: **5a** (0.13 mmol), ArMgBr (0.195 mmol), THF (2 mL).

b Isolated yields of product.

c Numbers in parentheses indicate yields according to ^31^P NMR.

d Formation of a small amount of ketone **3a** was observed but not isolated.

**Scheme 4:**
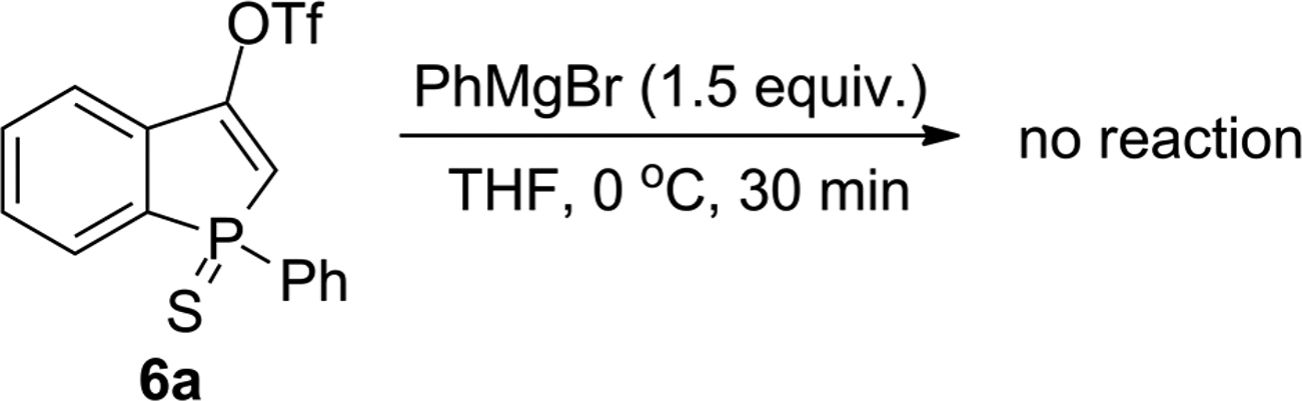
Reactivity of **6a** toward PhMgBr

To verify the influence of the P = X center on the reaction course, sulfide **6a** derived from 1-phenylbenzophosphole sulfide (**4a**) was subjected to the reaction with PhMgBr. In contrast to phosphine oxide **5a**, phosphine sulfide **6a** turned out to be unreactive under the developed conditions, and **6a** has been recovered in 72% yield (Scheme 4).

**Scheme 5:**
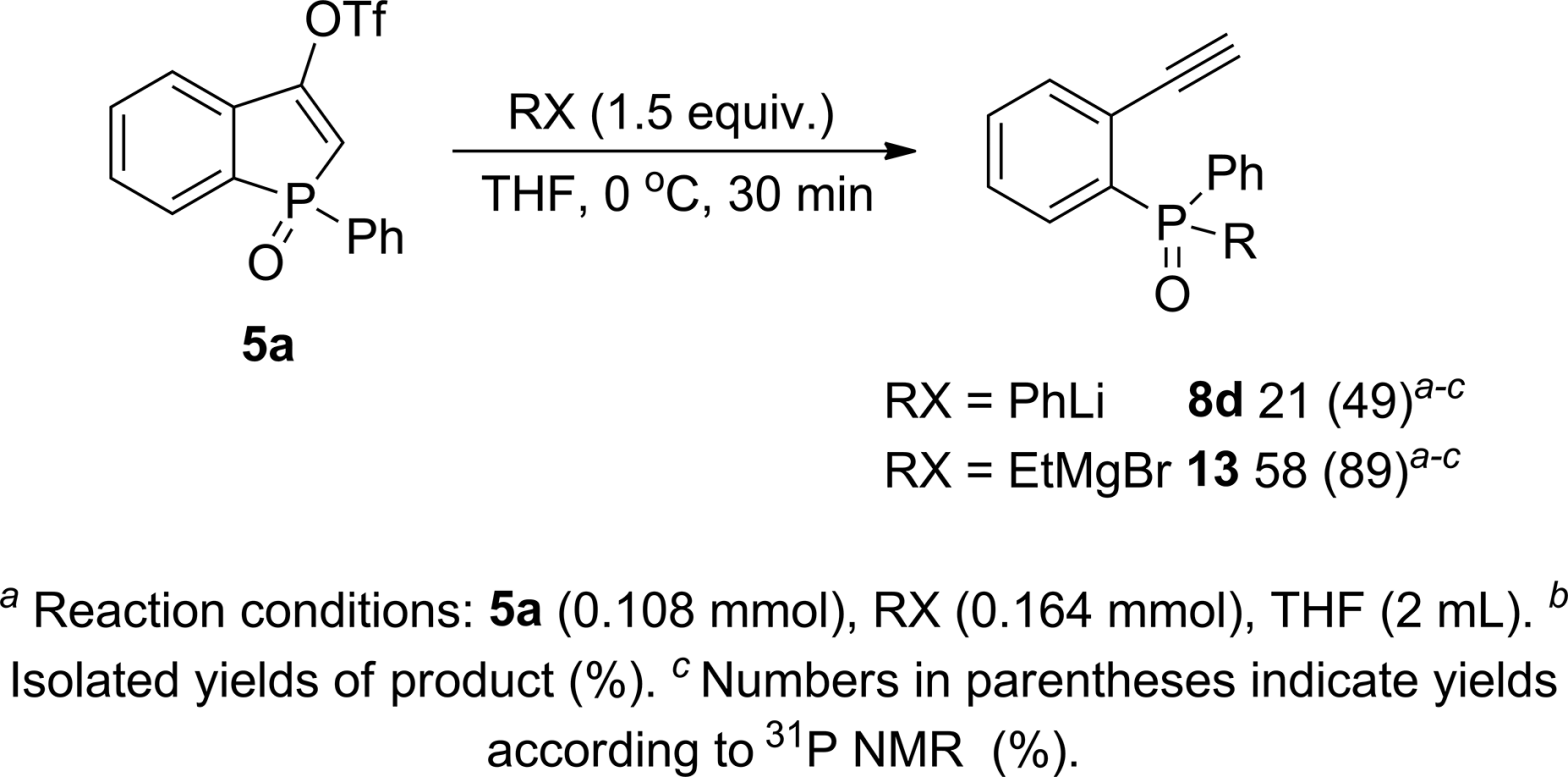
Reactivity of **5a** toward Different Organometallic Nucleophiles

In the last part of the study, we subjected triflate **5a** to the reactions with different organometallic nucleophiles (Scheme 5). In the case of the reaction with phenyllithium, the selectivity of the reaction toward the formation of **8d** has lowered in comparison to the use of PhMgBr. Compound **8d** was still the main product but has remained in the mixture with other unidentified products. Finally, we were able to isolate **8d** in a 21% yield. In turn, the reaction with the alkyl Grignard reagent has led to similar reactivity as it was observed for aryl Grignard reagents. As the result of the reaction of **5a** with EtMgBr, ethyl(2-ethynylphenyl)phenylphosphine oxide (**13**) was observed as a major product in the reaction mixture and isolated in 58% yield.

**Scheme 6:**
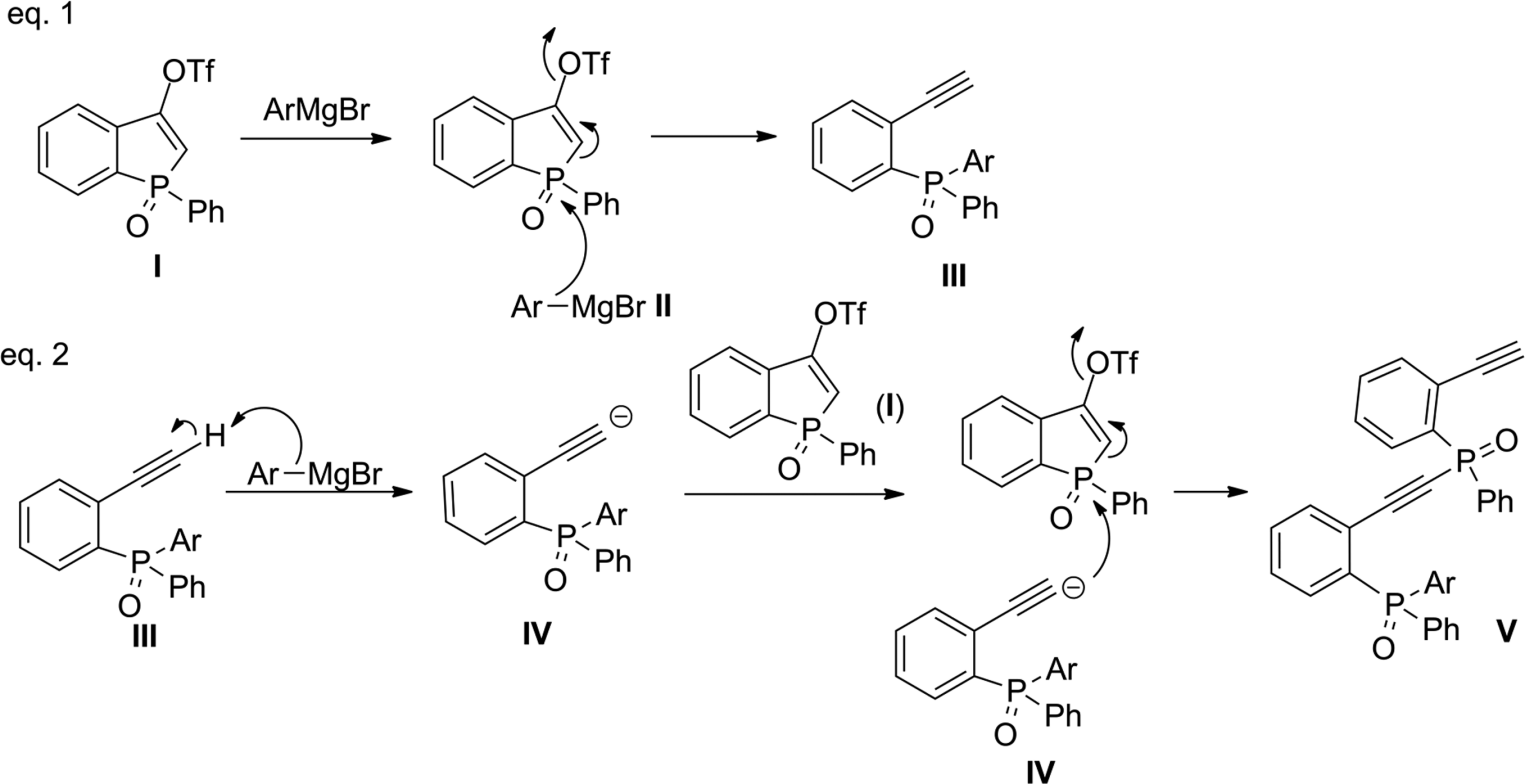
Plausible Mechanism of the Formation of Products **8a** and **9**

A plausible mechanism for the formation of 2-ethynylphenyl(diaryl)phosphine oxides is depicted in Scheme 6 (eq 1). This includes the attack of an aryl Grignard reagent (**II**) on the phosphorus atom in triflate **I**, which, in turn, causes the opening of the five-membered ring and the formation of a triple bond in the *ortho* position to the phosphorus center. The consequence of the ring opening is the cleavage of the leaving group (^−^OTf), resulting in the formation of 2-ethynylphenyl(diaryl)phosphine oxide (**III**).

In eq 2, a plausible mechanism of the subsequent reaction of phosphine oxide **III**, leading to compound **V**, is presented. The deprotonation of the formed phosphine oxide **III** by an aryl Grignard reagent leads to the formation of an anion **IV**, which competes with the Grignard reagent in a reaction with another molecule of **I**. The attack of anion **IV** leads to an analogous ring opening of **I** as it was observed for the Grignard reagent. In accordance with eq 1, the formation of a triple bond in the *ortho* position in compound **V** is observed. As a consequence, product **V** with an alkyne–phosphorus bond is formed.

## Conclusion

In summary, we developed a facile method for the synthesis of 2-ethynylphenyl(diaryl)phosphine oxides from readily available reagents under mild conditions and in a short time. It is the first example of the preparation of arylphosphine oxides possessing a terminal ethynyl moiety in the *ortho* position in a phenyl ring. The mechanism for the formation of both 2-ethynylphenyl(diaryl)phosphine oxides **8** and diphosphorus compound **9** has been proposed. The scope of the method for diverse substitution patterns in a structure of a Grignard reagent, benzophosphole core, and phosphorus atom has been investigated. The application of the method for the higher-scale preparations has been confirmed. The study of reactivity of 2-ethynylphenyl(diaryl)phosphine oxides and compound **9** is underway in our laboratory.

## Experimental Section

All reactions were performed under an argon atmosphere using Schlenk techniques. Only dry solvents were used, and glassware was heated under a vacuum prior use. All chemicals were used as received unless noted otherwise. Solvents for chromatography and crystallization were distilled once before use, and the solvents for extraction were used as received. THF and toluene were distilled from sodium/benzophenone ketyl under argon. DCM was dried using P_4_O_10_ and distilled before use.

^1^H NMR, ^31^P{^1^H} NMR, and ^13^C{^1^H} NMR spectra were recorded on a Bruker Advance 500 spectrometer at an ambient temperature in CDCl_3_ unless otherwise noted. Chemical shifts (δ) are reported in ppm from tetramethylsilane with the solvent as an internal indicator (CDCl_3_ 7.27 ppm for ^1^H and 77 ppm for ^13^C). Structural assignments were made with additional information from DEPT experiments. Mass spectra were recorded on Shimadzu GC–MS QP2010S in electron ionization (EI). Melting points were determined on a Büchi Melting Point M-560 in a capillary tube and were uncorrected. HPLC–HRMS was performed on a Shimazu HRMS ESI-IT-TOF using a reverse-phase stationary phase with water/MeCN (65:35) as an eluent, electrospray ionization (ESI), and an IT-TOF detector. Elementary analyses were performed on a Perkin Elmer CHN 2400. Thin-layer chromatography (TLC) was performed with precoated silica gel plates and visualized by UV light or KMnO_4_ solution or iodide on silica gel. The reaction mixtures were purified by column chromatography over silica gel (60–240 mesh).

Single crystals for **9** were obtained by dissolving 35 mg of **9** (an oil from chromatography column) in about 1 mL of AcOEt. Then 24 h later at 25 °C, colorless crystals appeared. Obtained crystals were isolated and dried for 24 h in rt.

The X-ray intensity data for **9** were collected on a diffractometer IPDS2T equipped with a STOE image plate detector system using a microfocus X-ray source providing Kα radiation by high-grade multilayer X-ray mirror optics for a Mo (λ = 0.71073 Å) wavelength. The measurement was carried out at 120 K. The structure of the compounds was solved by direct methods and refined against *F*^2^ with the Shelxs-2008 and Shelxl-2008 programs^33^ run under WinGX.^34^ Non-hydrogen atoms were refined with anisotropic displacement parameters. The isotropic displacement parameters of all hydrogens were fixed to 1.2 *U*_eq_ for CH and CH_2_ (1.5 times for methyl groups).

The crystallographic data for the structure of **9** reported in this work have been deposited in the Cambridge Crystallograic Data Centre (CCDC 2094907).

The starting compounds were prepared according to reported methods: phenyl(methyl)phosphine oxide,^35^ methyl 2-iodobenzoate,^36^ methyl 2-iodo-6-methylbenzoate,^37^ methyl 2-iodo-5-chlorobenzoate,^38^ 2-iodo-4-methoxybenzoic acid,^39^ methyl 2-iodo-4-methoxybenzoate.^40^

### Syntheses of 2-Methoxycarbonylphenylphosphine Oxides **2** Were Performed According to a Reported Procedure (General Procedure A).^35^

#### (2-Methoxycarbonylphenyl)(methyl)phenylphosphine Oxide (**2a**).^35^

Methyl(phenyl)phosphine oxide (1.248 g, 8.91 mmol) was reacted according to a reported procedure^35^ with methyl 2-iodobenzoate (2.567 g, 9.7 mmol) to afford **2a** (66%, 1.61 g, 5.9 mmol). ^1^H NMR (500 MHz, CDCl_3_): δ 8.33–8.53 (m, 1H), 7.87–7.89 (m, 1H), 7.68–7.71 (m, 1H), 7.56–7.63 (m, 3H), 7.42–7.49 (m, 1H), 7.38–7.42 (m, 2H), 3.54 (s, 3H), 2.22 (d, *J*_P–H_ = 13.87 Hz, 3H). ^13^C{^1^H} NMR (125 MHz, CDCl_3_): δ 167.0 (d, *J*_P–H_ = 2.7 Hz, C), 134.5 (d, *J*_P–H_ = 107.2 Hz, C), 134.3 (d, *J*_P–H_ = 7.3 Hz, CH), 133.8 (d, *J*_P–H_ = 92.6 Hz, C), 133.6 (d, *J*_P–H_ = 6.4 Hz, CH), 131.7 (s), 131.6 (s), 131.2 (d, *J*_P–H_ = 2.7 Hz, CH), 130.2 (d, *J*_P–H_ = 8.2 Hz, CH), 129.8 (d, *J*_P–H_ = 10.0 Hz, CH), 128.2 (d, *J*_P–H_ = 11.8 Hz, CH), 52.7 (s, H_3_CO), 16.3 (d, *J*_P–H_ = 77.2 Hz, CH_3_). ^31^P{^1^H} NMR (202 MHz, CDCl_3_): δ 33.45 (s).

#### [(2-Methoxycarbonyl)-6-methylphenyl]methylphenylphosphine Oxide (**2b**)

Methyl(phenyl)phosphine oxide (2.2 g, 15.7 mmol) was reacted according to a reported procedure^35^ with methyl 2-iodo-6-methylbenzoate (4.77 g, 17.4 mmol) to afford **2b** as a brown solid (65%, 2.96 g, 10.2 mmol). Mp: 73.4–74.2 °C. *R*_*f*_ = 0.34 (30:5:1 CHCl_3_/AcOEt/MeOH). ^1^H NMR (500 MHz, CDCl_3_): δ 7.66–7.70 (m, 2H), 7.49–7.53 (m, 1H), 7.43–7.56 (m, 3H), 7.36–7.40 (m, 1H), 7.27–7.31 (m, 1H), 3.69 (s, 3H), 2.35 (s, 3H), 2.17 (d, *J*_P–H_ = 13.40 Hz, 3H). ^13^C{^1^H} NMR (125 MHz, CDCl_3_): δ 170.2 (d, *J*_C–P_ = 4.5 Hz, C), 142.6 (d, *J*_C–P_= 9.1 Hz, C), 139.1 (d, *J*_C–P_= 7.3 Hz, C), 135.1 (d, *J*_C–P_ = 103.5 Hz, C), 133.6 (d, *J*_P–C_ = 10.0 Hz, CH), 131.6 (d, *J*_C–P_ = 2.7 Hz, CH), 131.2 (d, *J*_C–P_ = 1.8 Hz, CH), 130.1 (d, *J*_C–P_ = 10.9 Hz, 2CH), 129.9 (d, *J*_C–P_ = 94.5 Hz, C), 128.6 (d, *J*_C–P_ = 11.8 Hz, 2CH), 126.1 (d, *J*_C–P_ = 9.1 Hz, CH), 52.6 (s, H_3_CO), 22.7 (d, *J*_C–P_ = 4.5 Hz, CH_3_), 16.6 (d, *J*_C–P_ = 74.6 Hz, CH_3_). ^31^P{^1^H} NMR (202 MHz, CDCl_3_): δ 32.57 (s). GC–MS (EI) *m*/*z*: 274 (29) (M-CH_3_+H)^+^, 273 (100) (M-CH_3_)^+^, 256 (14), 213 (10), 195 (27), 179 (8), 178 (8), 167 (13), 166 (15), 165 (48), 152 (16), 139 (24), 137 (11), 121 (12), 109 (12), 107 (9), 91 (29), 90 (18), 89 (46), 79 (14), 78 (60), 77 (99), 74 (16). HRMS (ESI/Q-TOF) *m*/*z*: [M + Na]^+^calcd for C_16_H_17_O_3_PNa, 311.0808; found, 311.0802.

#### [(2-Methoxycarbonyl)-5-chlorophenyl]methylphenylphosphine Oxide (**2c**)

Methyl(phenyl)phosphine oxide (2.41 g, 17.2 mmol) was reacted according to a reported procedure^35^ with methyl 5-chloro-2-iodo-benzoate (5.16 g, 17.4 mmol) to afford **2c** as a brown solid (52%, 2.74 g, 8.9 mmol). Mp: 111–112 °C. *R*_*f*_ = 0.38 (30:5:1 CHCl_3_/AcOEt/MeOH). ^1^H NMR (500 MHz, CDCl_3_): δ 8.43–8.46 (m, 1H), 7.85–7.88 (m, 1H), 7.58–7.63 (m, 3H), 7.47–7.51 (m, 1H), 7.41–7.45 (m, 2H), 3.56 (s, 3H), 2.25 (d, *J*_P–H_ = 14.19 Hz, 3H). ^13^C{^1^H} NMR (125 MHz, CDCl_3_): δ 166.1 (d, *J*_C–P_ = 1.8 Hz, C), 139.0 (d, *J*_C–P_ = 13.6 Hz, C), 136.7 (d, *J*_C–P_ = 89.0 Hz, C), 134.7 (d, *J*_C–P_ = 7.3 Hz, CH), 134.1 (d, *J*_C–P_ = 108.1 Hz, C), 131.8 (d, *J*_C–P_ = 9.1 Hz, CH), 131.7 (d, *J*_C–P_ = 1.8 Hz, CH), 131.5 (d, *J*_C–P_ = 2.7 Hz, CH), 129.9 (d, *J*_C–P_ = 10.0 Hz, CH), 128.3 (d, *J*_C–P_ = 12.7 Hz, CH), 52.4 (s, CH_3_), 16.0 (d, *J*_C–P_ = 78.1 Hz, CH_3_). ^31^P{^1^H} NMR (202 MHz, CDCl_3_): δ 32.44 (s). GC–MS (EI) *m*/*z*: 295 (22), 294 (7), 293(72) (M-CH_3_)^+^, 263 (6), 165 (9), 163 (11), 154 (11), 153 (8), 152 (18), 151 (17), 139 (28), 138 (17), 126 (10). HRMS (ESI/Q-TOF) *m*/*z*: [M + Na]^+^ calcd for C_15_H_14_ClO_3_PNa, 331.0261; found, 331.0270.

#### [(2-Methoxycarbonyl)-4-methoxyphenyl]methylphenylphosphine Oxide (**2d**)

Methyl(phenyl)phosphine oxide (2.27 g, 16.2 mmol) was reacted according to a reported procedure^35^ with methyl 2-iodo-4-methoxy-benzoate (5.25 g, 17.98 mmol) to afford **2d** as a yellowish solid (55%, 2.71 g, 8.91 mmol). Mp: 115.9–116.5 °C. *R*_*f*_ = 0.28 (30:5:1 CHCl_3_/AcOEt/MeOH). ^1^H NMR (500 MHz, CDCl_3_): δ 8.26–8.31 (m, 1H), 7.58–7.62 (m, 2H), 7.42–7.49 (m, 1H), 7.39–7.41 (m, 3H), 7.17–7.20 (m, 1H), 3.89 (s, 3H), 3.54 (s, 3H), 2.19 (d, *J*_P–H_ = 14.2 Hz, 3H). ^13^C{^1^H} NMR (125 MHz, CDCl_3_): δ 166.8 (d, *J*_C–P_ = 1.8 Hz, C), 162.0 (d, *J*_C–P_ = 1.8 Hz, C), 136.4 (d, *J*_C–P_ = 9.1 Hz, CH), 135.3 (d, *J*_C–P_ = 7.3 Hz, C), 135.1 (d, *J*_C–P_ = 106.3 Hz, C), 131.1 (d, *J*_C–P_ = 2.7 Hz, CH), 129.8 (d, *J*_C–P_ = 10.0 Hz, 2CH), 128.2 (d, *J*_C–P_ = 12.7 Hz, 2CH), 124.8 (d, *J*_C–P_ = 100.0 Hz, C), 116.5 (d, *J*_C–P_ = 2.7 Hz, CH), 116.4 (d, *J*_C–P_ = 5.5 Hz, CH), 55.6 (s, CH_3_), 52.2 (s, CH_3_), 16.6 (d, *J*_C–P_ = 78.1 Hz, CH_3_). ^31^P{^1^H} NMR (202 MHz, CDCl_3_): δ 32.55 (s). GC–MS (EI): *m*/*z* 304 (2) (M)^+^, 290 (18), 289 (100) (M-CH_3_)^+^, 273 (13), 257 (16), 227 (13), 211 (13), 139 (31), 121 (11), 107 (10), 91 (14), 77 (50). HRMS (ESI/Q-TOF) *m*/*z*: [M + H]^+^ calcd for C_16_H_17_O_4_P, 305.0937; found, 305.0929.

### Synthesis of Benzophospholan-3-one Oxides **3** (General Procedure B).^41^

To a Schlenk tube (100 mL) equipped with a magnetic stirrer and an argon inlet phosphine was added oxide **2** (2.0 mmol) in anhydrous THF (15 mL), and the mixture was cooled to −78 °C with a dry ice/acetone bath. Then, LDA (2.5 mL, 5.0 mmol, 2 M solution) was added, and the mixture was stirred at the same temperature for 30 min. After that time, the ice bath was removed. The reaction mixture was stirred at rt overnight and then quenched by the addition of a saturated NH_4_Cl solution (5 mL) and extracted with CHCl_3_ (5 × 10 mL). The collected organic phases were dried over Na_2_SO_4_, the solid was filtered off, and the filtrate was evaporated under reduced pressure. The crude residue was checked using NMR techniques. Then, the crude mixture was dissolved in DCM (30 mL), solution of HCl (1 M, 10 mL) was added, and the mixture was vigorously stirred for 30 min. Then the acidic water phase was removed in a separating funnel, the organic phase was returned to the flask, and another portion of HCl (1 M, 10 mL) was added. The mixture was once again vigorously stirred for 30 min, and after that time, the acidic water phase was separated. The organic phase was washed with water (10 mL) and dried over Na_2_SO_4_. The solid was filtered off, and the filtrate was evaporated under reduced pressure. The residue was purified by short (5 cm) column chromatography on silica gel using CHCl_3_/acetone (2:1 v/v) or CHCl_3_/EtOAc/MeOH (30:5:1 v/v/v) as an eluent.

#### Benzophospholan-3-one Oxide (**3a**).^29^

Compound **2a** (0.55 g, 2.0 mmol) was reacted according to general procedure B to afford **3a** (55%, 0.266 g, 1.1 mmol). ^1^H NMR (500 MHz, CDCl_3_): δ 8.03–8.07 (m, 1H), 7.85–7.90 (m, 1H), 7.75–7.83 (m, 2H), 7.52–7.60 (m, 3H), 7.44–7.48 (m, 2H), 3.12–3.31 (m, 2H). ^13^C{^1^H} NMR (125 MHz, CDCl_3_): δ 194.3 (d, *J*_C–P_ = 12.7 Hz, C), 141.5 (d, *J*_C–P_ = 91.7 Hz, C), 141.3 (d, *J*_C–P_ = 12.7 Hz, C), 135.9 (d, *J*_C–P_ = 10.9 Hz, CH), 133.6 (d, *J*_C–P_ = 2.7 Hz, CH), 132.5 (d, *J*_C–P_ = 2.7 Hz, CH), 130.8 (d, *J*_C–P_ = 105.4 Hz, C), 130.6 (d, *J*_C–P_ = 10.9 Hz, 2CH), 129.3 (d, *J*_C–P_ = 6.4 Hz, CH), 128.9 (d, *J*_C–P_ = 13.6 Hz, CH), 124.6 (d, *J*_C–P_ = 10.9 Hz, CH), 40.1 (d, *J*_C–P_ = 71.8 Hz, CH_2_). ^31^P{^1^H} NMR (202 MHz, CDCl_3_): δ 29.92 (s).

#### 7-Methylbenzophospholan-3-one Oxide (**3b**)

Compound **2b** (0.577 g, 2.0 mmol) was reacted according to general procedure B to afford **3b** as a beige solid (55%, 0.28 g, 1.1 mmol). Mp: 165–166 °C (dec.). *R*_*f*_ = 0.66 (2:1 CHCl_3_/acetone). ^1^H NMR (500 MHz, CDCl_3_): δ 7.78–7.7.92 (m, 1H), 7.64–7.68 (m, 1H), 7.53–7.63 (m, 4H), 7.46–7.58 (m, 2H), 3.09–3.32 (m, 2H), 2.43 (s, 3H). ^13^C{^1^H} NMR (125 MHz, CDCl_3_): δ 194.6 (d, *J*_C–P_ = 13.6 Hz, C), 142.0 (d, *J*_C–P_ = 5.5 Hz, C), 141.8 (d, *J*_C–P_ = 12.7 Hz, C), 139.7 (d, *J*_C–P_ = 90.8 Hz, C), 137.1 (d, *J*_C–P_ = 9.1 Hz, CH), 133.9 (d, *J*_C–P_ = 1.8 Hz, CH), 132.4 (d, *J*_C–P_ = 3.6 Hz, CH), 130.9 (d, *J*_C–P_ = 103.5 Hz, C), 130.6 (d, *J*_C–P_ = 10.9 Hz, CH), 129.0 (d, *J*_C–P_ = 12.7 Hz, CH), 122.2 (d, *J*_C–P_ = 11.8 Hz, CH), 40.5 (d, *J*_C–P_ = 71.5 Hz, CH_2_), 19.4 (d, *J*_C–P_ = 4.5 Hz, CH_3_). ^31^P{^1^H} NMR (202 MHz, CDCl_3_): δ 30.36 (s). GC–MS (EI) *m*/*z*: 258 (7), 257 (13), 256 (63) (M)^+^, 255 (37), 236 (8), 213 (24), 179 (9), 166 (28), 165 (42), 151 (10), 150 (12), 137 (14), 121 (12), 106 (29). HRMS (ESI/Q-TOF) *m*/*z*: calcd for C_15_H_13_O_2_P [M + Na]^+^, 279.0545; found, 279.0553.

#### 6-Chlorobenzophospholan-3-one Oxide (**3c**)

Compound **2c** (0.617 g, 2.0 mmol) was reacted according to general procedure B to afford **3c** as an orange oil (55%, 0.3 g, 1.1 mmol). *R*_*f*_ = 0.36 (30:5:1 CHCl_3_/AcOEt/MeOH). ^1^H NMR (500 MHz, CDCl_3_): δ 7.99–8.01 (m, 1H), 7.81–7.84 (m, 1H), 7.72–7.74 (m, 1H), 7.58–7.62 (m, 3H), 7.49–7.52 (m, 2H), 3.16–3.36 (m, 2H). ^13^C{^1^H} NMR (125 MHz, CDCl_3_): δ 192.9 (d, *J*_C–P_ = 12.7 Hz, C), 143.5 (d, *J*_C–P_ = 108.1 Hz, C), 143.1 (d, *J*_C–P_= 33.6 Hz, C), 139.5 (d, *J*_C–P_ = 12.7 Hz, C), 134.2 (d, *J*_C–P_ = 2.7 Hz, CH), 132.9 (d, *J*_C–P_ = 3.6 Hz, CH), 130.7 (d, *J*_C–P_ = 10.9 Hz, CH), 130.1 (d, *J*_C–P_ = 106.3 Hz, C), 129.3 (d, *J*_C–P_ = 6.4 Hz, CH), 129.2 (d, *J*_C–P_ = 14.5 Hz, CH), 126.1 (d, *J*_C–P_ = 11.8 Hz, CH), 40.2 (d, *J*_C–P_ = 71.8 Hz, CH_2_). ^31^P{^1^H} NMR (202 MHz, CDCl_3_): δ 32.55 (s). GC–MS (EI) *m*/*z*: 278 (14), 277 (7), 276 (49) (M)^+^, 235 (14), 234 (14), 233 (57). HRMS (ESI/Q-TOF) *m*/*z*: calcd for C_14_H_10_ClO_2_P [M + H]^+^, 277.0180; found, 277.0180.

#### 5-Methoxybenzophospholan-3-one Oxide (**3d**)

Compound **2d** (0.609 g, 2.0 mmol) was reacted according to general procedure B to afford **3d** as a brown oil (59%, 0.32 g, 1.18 mmol). *R*_*f*_ = 0.72 (2:1 CHCl_3_/acetone). ^1^H NMR (500 MHz, CDCl_3_): δ 7.74–7.78 (m, 1H), 7.52–7.59 (m, 3H), 7.44–7.47 (m, 3H), 7.32–7.35 (m, 1H), 3.93 (m, 3H), 3.11–3.32 (m, 2H). ^13^C{^1^H} NMR (125 MHz, CDCl_3_): δ 194.3 (d, *J*_C–P_ = 12.7 Hz, C), 164.2 (d, *J*_C–P_ = 2.7 Hz, C), 144.0 (d, *J*_C–P_ = 14.5 Hz, C), 133.2 (d, *J*_C–P_ = 97.2 Hz, C), 132.4 (d, *J*_C–P_ = 3.6 Hz, CH), 133.2 (d, *J*_C–P_ = 107.2 Hz, C), 130.7 (d, *J*_C–P_ = 10.7 Hz, 2CH), 130.6 (d, *J*_C–P_ = 7.4 Hz, CH), 129.0 (d, *J*_C–P_ = 12.9 Hz, 2CH), 124.7 (d, *J*_C–P_ = 11.7 Hz, CH), 106.5 (d, *J*_C–P_ = 12.6 Hz, CH), 55.9 (s, OCH_3_), 40.8 (d, *J*_C–P_ = 71.8 Hz, CH_2_). ^31^P{^1^H} NMR (202 MHz, CDCl_3_): δ 28.92 (s). GC–MS (EI) *m*/*z*: 273 (24), 272 (100) (M)^+^, 271 (17), 256 (12), 244 (21), 229 (49), 210 (12), 208 (15), 195 (18), 193 (19), 183 (10), 179 (31), 165 (21), 153 (12), 152 (29), 148 (13), 139 (21), 137 (11), 120 (43), 107 (24), 91 (36), 89 (14). HRMS (ESI/Q-TOF) *m*/*z*: calcd for C_15_H_13_O_3_P [M + H]^+^, 273.0675; found, 273.0681.

### Synthesis of Benzophospholan-3-one Sulfide (**4a**, General Procedure C)

To a Schlenk tube (100 mL) equipped with a magnetic stirrer and an argon inlet was added phosphine oxide **3a** (0.528 g, 2.18 mmol) in anhydrous toluene (20 mL). Then, HSiCl_3_ (2.2 mL, 21.8 mmol) was added, and the mixture was stirred at rt for 18 h. The crude reaction mixture was checked using the ^31^P{^1^H} NMR technique. Then, sulfur (21.8 mmol) was added, and the reaction mixture was stirred at rt for 8 h. Then, the reaction mixture was quenched by the addition of saturated NH_4_Cl solution (5 mL) and extracted with CHCl_3_ (5 × 10 mL). The collected organic phases were dried over Na_2_SO_4_, the solid was filtered off, and the filtrate was evaporated under reduced pressure. The crude product was purified by column chromatography on silica gel using hexane/*i*-PrOH (10:1 v/v) as an eluent.

#### Benzophospholan-3-one Sulfide (**4a**)

Compound **3a** (0.48 g, 2.0 mmol) was reacted according to general procedure C to afford **4** as a brownish oil (67%, 0.346 g, 1.34 mmol). *R*_*f*_ = 0.44 (10:1 hexane/*i*-PrOH). ^1^H NMR (500 MHz, CDCl_3_): δ 8.03–8.07 (m, 1H), 7.83–7.91 (m, 2H), 7.74–7.78 (m, 1H), 7.65–7.70 (m, 2H), 7.52–7.53 (m, 1H), 7.44–7.48 (m, 2H), 3.35–3.47 (m, 2H). ^13^C{^1^H} NMR (125 MHz, CDCl_3_): δ 195.3 (d, *J*_C–P_ = 9.1 Hz, C), 143.4 (d, *J*_C–P_ = 76.3 Hz, C), 139.6 (d, *J*_C–P_ = 10.9 Hz, C), 136.4 (d, *J*_C–P_ = 11.8 Hz, CH), 132.9 (d, *J*_C–P_ = 2.7 Hz, CH), 132.1 (d, *J*_C–P_ = 3.6 Hz, CH), 131.2 (d, *J*_C–P_ = 81.8 Hz, C), 130.8 (d, *J*_C–P_ = 11.8 Hz, 2CH), 129.4 (d, *J*_C–P_ = 7.3 Hz, CH), 128.9 (d, *J*_C–P_ = 12.7 Hz, 2CH), 128.3 (d, *J*_C–P_ = 19.9 Hz, C), 124.8 (d, *J*_C–P_ = 10.9 Hz, CH), 45.8 (d, *J*_C–P_ = 57.2 Hz, CH_2_). ^31^P{^1^H} NMR (202 MHz, CDCl_3_): δ 32.02 (s). GC–MS (EI) *m*/*z*: 260 (7), 259 (58) (M)^+^, 258 (100), 226 (15), 184 (10), 185 (33), 165 (24), 153 (11), 152 (31), 151 (7), 150 (12), 149 (98), 139 (21), 136 (10), 134 (12), 121 (36), 120 (12), 118 (11), 109 (44), 108 (24), 107 (67), 95 (13), 91 (29), 90 (15), 89 (20), 81 (19), 78 (20). Anal. Calcd for C_14_H_11_OPS: C, 65.10; H, 4.29. Found: C, 64.90; H, 4.10.

### Synthesis of Triflates **5** and **6a** (General Procedure D)

To a Schlenk tube (50 mL) equipped with a magnetic stirrer and an argon inlet was added phosphine oxide **3** (0.3 mmol) or sulfide **4a** (0.3 mmol) in anhydrous DCM (8 mL), and the mixture was cooled to −78 °C with a dry ice/acetone bath. DIPEA (78.4 μL, 0.45 mmol) was added dropwise. Then, Tf_2_O (61 μL, 0.36 mmol) was added dropwise with vigorous stirring. The reaction mixture was stirred at −78 °C for 30 min. The crude reaction mixture was checked using the ^31^P{^1^H} NMR technique. Then, the reaction mixture was quenched by the addition of 1 M HCl solution (5 mL) and extracted with CHCl_3_ (5 × 10 mL). The collected organic phases were dried over Na_2_SO_4_, the solid was filtered off, and the filtrate was evaporated under reduced pressure. The crude product was purified by column chromatography on silica gel using CHCl_3_/acetone (2:1 v/v) as an eluent.

#### 1-Oxido-1-phenyl-1*H*-phosphindol-3-yl Trifluoromethanesulfonate (**5a**)

Compound **3a** (0.073 g, 0.3 mmol) was reacted according to general procedure D to afford **5a** as an orange solid (75%, 0.084 g, 0.225 mmol). Mp: 120.1–121.1 °C. *R*_*f*_ = 0.76 (2:1 CHCl_3_/acetone). ^1^H NMR (500 MHz, CDCl_3_): δ 7.68–7.75 (m, 3H), 7.62–7.66 (m, 1H), 7.56–7.61 (m, 1H), 7.52–7.57 (m, 2H), 7.46–7.50 (m, 2H), 6.31 (d, *J*_H–P_ = 14.82 Hz, 1H). ^13^C{^1^H} NMR (125 MHz, CDCl_3_): δ 156.7 (d, *J*_C–P_ = 34.5 Hz, C), 135.8 (d, *J*_C–P_ = 19.1 Hz, C), 133.4 (d, *J*_C–P_ = 1.8 Hz, CH), 133.1 (d, *J*_C–P_ = 2.7 Hz, CH), 132.2 (d, *J*_C–P_ = 104.5 Hz, C), 131.5 (d, *J*_C–P_ = 10.9 Hz, CH), 130.8 (d, *J*_C–P_ = 10.9 Hz, 2CH), 129.5 (d, *J*_C–P_ = 8.2 Hz, CH), 129.1 (d, *J*_C–P_ = 12.7 Hz, 2CH), 127.5 (d, *J*_C–P_ = 108.1 Hz, C), 121.0 (d, *J*_C–P_ = 10.9 Hz, CH), 118.5 (q, *J*_C–P_ = 321.5 Hz, CF_3_), 108.9 (d, *J*_C–P_ = 95.4 Hz, CH). ^31^P{^1^H} NMR (202 MHz, CDCl_3_): δ 32.12 (s). ^19^F NMR (470 MHz, CDCl_3_): δ −72.96 (s). GC–MS (EI) *m*/*z*: 374 (5) (M)^+^, 240 (23), 194 (44), 183 (11), 179 (6), 178 (31), 177 (6), 166 (16), 165 (44), 152 (11), 151 (7), 107 (15), 89 (12), 77 (17), 69 (100). HRMS (ESI/Q-TOF) *m*/*z*: calcd for C_15_H_10_F_3_O_4_PS [M + H]^+^, 375.0062; found, 375.0053.

#### 1-Oxido-1-phenyl-1*H*-7-methylphosphindol-3-yl Trifluoromethanesulfonate (**5b**)

Compound **3b** (0.077 g, 0.3 mmol) was reacted according to general procedure D to afford **5b** as a yellow solid (55%, 0.064 g, 0.165 mmol). Mp: 125–126.5 °C. *R*_*f*_ = 0.68 (2:1 CHCl_3_/acetone). ^1^H NMR (500 MHz, CDCl_3_): δ 7.73–7.77 (m, 2H), 7.58–7.61 (m, 1H), 7.47–7.53 (m, 3H), 7.35–7.37 (m, 1H), 7.27–7.29 (m, 2H), 6.25 (d, *J*_H–P_ = 15.13 Hz, 1H), 2.37 (s, 3H). ^13^C{^1^H} NMR (125 MHz, CDCl_3_): δ 156.8 (d, *J*_C–P_ = 34.5 Hz, C), 142.2 (d, *J*_C–P_ = 8.2 Hz, C), 135.8 (d, *J*_C–P_ = 19.1 Hz, C), 133.5 (d, *J*_C–P_ = 2.7 Hz, CH), 133.1 (d, *J*_C–P_ = 10.0 Hz, CH), 132.9 (d, *J*_C–P_ = 2.7 Hz, CH), 130.8 (d, *J*_C–P_ = 10.9 Hz, 2CH), 130.3 (d, *J*_C–P_ = 103.5 Hz, C), 129.2 (d, *J*_C–P_ = 13.6 Hz, 2CH), 127.3 (d, *J*_C–P_ = 106.3 Hz, C), 118.5 (q, *J*_C–P_ = 321.5 Hz, CF_3_), 118.48 (d, *J*_C–P_ = 10.9 Hz, CH), 108.9 (d, *J*_C–P_ = 95.4 Hz, *C*H), 19.4 (s, CH_3_). ^31^P{^1^H} NMR (202 MHz, CDCl_3_): δ 32.47 (s). ^19^F NMR (470 MHz, CDCl_3_): δ −72.98 (s). GC–MS (EI) *m*/*z*: 388 (9) (M)^+^, 255 (9), 208 (33), 192 (18), 178 (16), 165 (26), 89 (13), 77 (19). HRMS (ESI/Q-TOF) *m*/*z*: calcd for C_16_H_12_O_4_F_3_PS [M + H]^+^, 389.0219; found, 389.0213.

#### 1-Oxido-1-phenyl-1*H*-6-chlorophosphindol-3-yl Trifluoromethanesulfonate (**5c**)

Compound **3c** (0.083 g, 0.3 mmol) was reacted according to general procedure D to afford **5c** as an orange oil (60%, 0.074 g, 0.18 mmol). *R*_*f*_ = 0.75 (2:1 CHCl_3_/acetone). ^1^H NMR (500 MHz, CDCl_3_): δ 7.71–7.75 (m, 2H), 7.58–7.65 (m, 3H), 7.45–7.52 (m, 3H), 6.31 (d, *J*_H–P_ = 15.29 Hz, 1H). ^13^C{^1^H} NMR (125 MHz, CDCl_3_): δ 156.2 (d, *J*_C–P_ = 33.6 Hz, C), 138.4 (d, *J*_C–P_ = 14.3 Hz, C), 134.6 (d, *J*_C–P_ = 100.8 Hz, C), 133.9 (d, *J*_C–P_ = 19.1 Hz, C), 133.4 (d, *J*_C–P_ = 2.7 Hz, CH), 133.3 (d, *J*_C–P_ = 1.8 Hz, CH), 130.8 (d, *J*_C–P_ = 10.9 Hz, 2CH), 128.9 (d, *J*_C–P_ = 10.0 Hz, CH), 128.4 (d, *J*_C–P_ = 13.6 Hz, 2CH), 126.7 (d, *J*_C–P_ = 108.9 Hz, C), 122.2 (d, *J*_C–P_ = 11.8 Hz, *C*H), 118.5 (q, *J*_C–P_ = 320.6 Hz, CF_3_), 109.9 (d, *J*_C–P_ = 98.6 Hz, CH). ^31^P{^1^H} NMR (202 MHz, CDCl_3_): δ 31.06 (s). ^19^F NMR (470 MHz, CDCl_3_): δ −72.83 (s). GC–MS (EI) *m*/*z*: 411 (3) 410 (10), 409 (5), 408 (28) (M)^+^, 277 (16), 276 (16), 275 (51), 259 (22), 232 (11), 229 (30), 229 (20), 228 (100), 214 (30), 213 (14), 212 (92), 211 (11), 199 (22), 196 (22), 193 (36), 183 (13), 176 (16), 166 (16), 165 (75), 164 (14), 151 (24), 140 (11), 107 (21), 105 (28), 89 (12). HRMS (ESI/Q-TOF) *m*/*z*: calcd for C_15_H_9_ClF_3_O_4_PS [M + H]^+^, 408.9673; found, 408.9681.

#### 1-Oxido-1-phenyl-1*H*-5-methoxyphosphindol-3-yl Trifluoromethanesulfonate (**5d**)

Compound **3c** (0.082 g, 0.3 mmol) was reacted according to general procedure D to afford **5d** as an orange oil (58%, 0.07 g, 0.174 mmol). *R*_*f*_ = 0.65 (2:1 CHCl_3_/acetone). ^1^H NMR (500 MHz, CDCl_3_): δ 7.68–7.72 (m, 2H), 7.55–7.62 (m, 2H), 7.44–7.48 (m, 2H), 7.03–7.04 (m, 1H), 6.97–7.00 (m, 1H), 6.30 (d, *J*_H–P_ = 14.66 Hz, 1H), 3.90 (s, 3H). ^13^C{^1^H} NMR (125 MHz, CDCl_3_): δ 164.0 (*J*_P–C_ = 1.8 Hz, C), 155.9 (d, *J*_P–C_ = 33.6 Hz, C), 138.2 (d, *J*_P–C_ = 20.9 Hz, C), 132.9 (d, *J*_P–C_ = 2.7 Hz, CH), 131.1 (d, *J*_P–C_ = 10.0 Hz, CH), 130.8 (d, *J*_P–C_ = 11.8 Hz, 2CH), 129.1 (d, *J*_P–C_ = 13.6 Hz, 2CH), 129.7 (d, *J*_P–C_ = 108.9 Hz, C), 122.7 (d, *J*_P–C_ = 110.8 Hz, C), 118.6 (q, *J*_P–C_ = 320.6 Hz, CF_3_), 115.5 (d, *J*_P–C_ = 12.7 Hz, CH), 110.5 (d, *J*_P–C_ = 94.5 Hz, CH), 108.0 (d, *J*_P–C_ = 11.8 Hz, CH), 55.8 (s, CH_3_). ^31^P{^1^H} NMR (202 MHz, CDCl_3_): δ 30.95 (s). ^19^F NMR (470 MHz, CDCl_3_): δ - 72.89 (s). GC–MS (EI) *m*/*z*: 405 (2), 404 (11) (M)^+^, 282 (13), 281 (33), 273 (11), 272 (62) (M-SO_2_CF_3_), 271 (57), 256 (72), 240 (20), 229 (24), 224 (71) 223 (18), 209 (38), 208 (94), 207 (100), 196 (15), 195 (25), 179 (49), 165 (29), 152 (34), 151 (18), 148 (18), 139 (24), 137 (18), 120 (36), 106 (24), 91 (47), 89 (19). HRMS (ESI/Q-TOF) *m*/*z*: calcd for C_16_H_12_O_5_F_3_PS [M + H]^+^, 405.0168; found, 405.0165.

#### 1-Sulfido-1-phenyl-1*H*-phosphindol-3-yl Trifluoromethanesulfonate (**6a**)

Compound **4a** (0.077 g, 0.3 mmol) was reacted according to general procedure D to afford **6** as a yellowish solid (50%, 0.058 g, 0.15 mmol). Mp: 109–110 °C. *R*_*f*_ = 0.59 (10:1 hexane/*i*-PrOH). ^1^H NMR (500 MHz, CDCl_3_): δ 7.78–7.82 (m, 2H), 7.68–7.72 (m, 2H), 7.54–7.59 (m, 3H), 7.44–7.48 (m, 2H), 6.40 (d, *J*_H–P_ = 18.60 Hz, 1H). ^13^C{^1^H} NMR (125 MHz, CDCl_3_): δ 154.7 (d, *J*_P–C_ = 28.9 Hz, C), 135.59 (d, *J*_P–C_ = 87.9 Hz, C), 135.58 (d, *J*_P–C_ = 16.2 Hz, C), 132.8 (d, *J*_P–C_ = 3.1 Hz, CH), 132.7 (d, *J*_P–C_ = 1.8 Hz, CH), 133.2 (d, *J*_P–C_ = 11.7 Hz, CH), 130.8 (d, *J*_P–C_ = 12.7 Hz, CH), 129.1 (d, *J*_P–C_ = 12.9 Hz, CH), 128.9 (d, *J*_P–C_ = 9.2 Hz, CH), 127.4 (d, *J*_P–C_ = 84.4 Hz, C), 121.3 (d, *J*_P–C_ = 10.4 Hz, CH), 118.5 (q, *J*_P–C_ = 321.4 Hz, CF_3_), 112.4 (d, *J*_P–C_ = 78.8 Hz, CH). ^31^P{^1^H} NMR (202 MHz, CDCl_3_): δ 41.0 (s). ^19^F NMR (470 MHz, CDCl_3_): δ −72.92 (s). GC–MS (EI) *m*/*z*: 391 (7), 390 (34), 258 (22), 257 (100), 239 (14), 229 (31), 225 (21), 210 (17), 197 (27), 196 (47), 195 (15), 194 (37), 193 (17), 182 (26), 178 (49), 166 (25), 165 (82), 152 (39). HRMS (ESI/Q-TOF) *m*/*z*: calcd for C_15_H_10_O_3_F_3_PS_2_ [M + H]^+^, 390.9834; found, 390.9834.

### Reaction of **5** or **6** with Aryl Grignard Reagents (General Procedure E)

To a Schlenk tube (25 mL) equipped with a magnetic stirrer and an argon inlet was added phosphine oxide **5** or **6** (0.13 mmol) in anhydrous THF (2 mL), and the mixture was cooled to 0 °C. Then, aryl Grignard reagent (0.195 mmol) was added. Then, the reaction mixture was stirred at 0 °C for 30–60 min. Then, the reaction mixture was quenched by the addition of NH_4_Cl solution (5 mL) and extracted with CHCl_3_ (5 × 5 mL). The collected organic phases were dried over Na_2_SO_4_, the solid was filtered off, and the was filtrate evaporated under reduced pressure. The crude reaction mixture was checked using the NMR technique. The residue was purified by column chromatography on silica gel using CHCl_3_/EtOAc/MeOH (30:5:1 v/v) or CHCl_3_/MTBE (30:1 v/v) as an eluent.

#### (2-Ethynylphenyl)phenyl(*p*-tolyl)phosphine Oxide (**8a**)

Compound **5a** (0.0486 g, 0.13 mmol) was reacted with *p*-TolMgBr (0.195 mL, 0.195 mmol, 1 M solution in THF) according to general procedure E for 30 min to afford **8a** (0.0403 g, 0.127 mmol, 98% yield) as a yellowish solid. Mp: 173–174 °C. *R*_*f*_ = 0.45 (30:5:1 CHCl_3_/AcOEt/MeOH). ^1^H NMR (500 MHz, CDCl_3_): δ 7.71–7.75 (m, 2H), 7.59–7.68 (m, 4H), 7.40–7.56 (m, 5H), 7.26–7.28 (m, 2H), 3.00 (s, 1H), 2.41 (s, 3H). ^13^C{^1^H} NMR (125 MHz, CDCl_3_): δ 142.3 (d, *J*_P–C_ = 2.7 Hz, C), 134.9 (d, *J*_P–C_ = 100.8 Hz, C), 134.9 (d, *J*_P–C_ = 9.1 Hz, CH), 133.8 (d, *J*_P–C_ = 9.1 Hz, CH), 132.5 (d, *J*_P–C_ = 106.3 Hz, C), 132.3 (d, *J*_P–C_ = 10.9 Hz, 2CH), 132.2 (d, *J*_P–C_ = 10.0 Hz, 2CH), 131.7 (d, *J*_P–C_ = 2.7 Hz, CH), 131.6 (d, *J*_P–C_ = 1.8 Hz, CH), 129.1 (d, *J*_P–C_ = 12.7 Hz, 2CH), 128.7 (d, *J*_P–C_ = 108.9 Hz, C), 128.4 (d, *J*_P–C_ = 11.8 Hz, CH), 128.3 (d, *J*_P–C_ = 11.8 Hz, CH), 125.3 (d, *J*_P–C_ = 7.3 Hz, C), 85.0 (s, C), 81.5 (d, *J*_P–C_ = 5.5 Hz, CH), 21.6 (s, CH_3_). ^31^P{^1^H} NMR (202 MHz, CDCl_3_): δ 28.70 (s). GC–MS (EI) *m*/*z*: 317 (21), 316 (93) (M)^+^, 315 (100), 297 (15), 269 (13), 268 (15), 254 (18), 253 (18), 252 (15), 239 (34), 223 (17), 215 (11), 210 (12), 197 (11), 196 (19), 191 (11), 189 (11), 182 (15), 174 (17), 165 (32), 152 (21), 91 (24), 89 (12), 78 (15). HRMS (ESI/Q-TOF) *m*/*z*: calcd for C_21_H_17_OP [M + H]^+^, 317.1090; found, 317.1088.

#### (2-Ethynylphenyl)phenyl(*m*-tolyl)phosphine Oxide (**8b**)

Compound **5a** (0.0486 g, 0.13 mmol) was reacted with *m*-TolMgBr (0.195 mL, 0.195 mmol, 1 M solution in THF) according to general procedure E for 30 min to afford **8b** (0.0345 g, 0.109 mmol, 84% yield) as a yellowish oil. *R*_*f*_ = 0.65 (2:1 CHCl_3_/acetone). ^1^H NMR (500 MHz, CDCl_3_): δ 7.71–7.75 (m, 2H), 7.60–7.68 (m, 3H), 7.39–7.58 (m, 6H), 7.33–7.36 (m, 2H), 3.00 (s, 1H), 2.37 (s, 3H). ^13^C{^1^H} NMR (125 MHz, CDCl_3_): δ 138.2 (d, *J*_P–C_ = 11.8 Hz, C), 134.9 (d, *J*_P–C_ = 8.2 Hz, CH), 134.7 (d, *J*_P–C_ = 100.8 Hz, C), 133.8 (d, *J*_P–C_ = 9.1 Hz, CH), 132.6 (d, *J*_P–C_ = 1.8 Hz, CH), 132.59 (d, *J*_P–C_ = 10.0 Hz, CH), 132.4 (d, *J*_P–C_ = 105.4 Hz, C), 132.15 (d, *J*_P–C_ = 10.0 Hz, 2CH), 134.7 (d, *J*_P–C_ = 100.8 Hz, C), 131.8 (d, *J*_P–C_ = 105.4 Hz, C), 131.7 (d, *J*_P–C_ = 2.7 Hz, CH), 131.6 (d, *J*_P–C_ = 1.8 Hz, CH), 129.3 (d, *J*_P–C_ = 10.9 Hz, CH), 128.4 (d, *J*_P–C_ = 11.8 Hz, CH), 128.3 (d, *J*_P–C_ = 12.7 Hz, 2CH), 128.1 (d, *J*_P–C_ = 13.6 Hz, CH), 125.3 (d, *J*_P–C_ = 6.4 Hz, C), 84.9 (s, C), 81.6 (d, *J*_P–C_ = 6.4 Hz, CH), 21.5 (s, CH_3_). ^31^P{^1^H} NMR (202 MHz, CDCl_3_): δ 28.56 (s). GC–MS (EI) *m*/*z*: 317 (22), 316 (100) (M)^+^, 315 (97), 268 (20), 267 (13), 254 (11), 253 (24), 252 (17), 239 (28), 210 (15), 207 (11), 197 (14), 196 (35), 195 (11), 194 (10), 189 (12), 183 (14), 178 (26), 176 (19), 166 (11), 165 (48), 153 (12), 152 (42), 151 (16), 150 (12), 107 (16), 102 (12), 101 (12), 92 (35), 91 (67), 89 (40). HRMS (ESI/Q-TOF) *m*/*z*: calcd for C_21_H_17_OP [M + Na]^+^, 339.0909; found, 339.0912.

#### (2-Ethynylphenyl)phenyl(*o*-tolyl)phosphine Oxide (**8c**)

Compound **5a** (0.0486 g, 0.13 mmol) was reacted with *o*-TolMgCl (0.195 mL, 0.195 mmol, 1 M solution in THF) according to general procedure E for 30 min to afford **8c** (0.0156 g, 0.0494 mmol, 38% yield) as a yellowish oil. *R*_*f*_ = 0.15 (30:1 CHCl_3_/MTBE). ^1^H NMR (500 MHz, CDCl_3_): δ 7.75–7.82 (m, 3H), 7.61–7.63 (m, 1H), 7.52–7.57 (m, 2H), 7.41–7.49 (m, 5H), 7.18–7.20 (m, 1H), 7.13–7.16 (m, 1H), 2.94 (s, 1H), 2.47 (s, 3H). ^13^C{^1^H} NMR (125 MHz, CDCl_3_): δ 143.2 (d, *J*_P–C_ = 8.2 Hz, C), 135.0 (d, *J*_P–C_ = 99.9 Hz, C), 134.9 (d, *J*_P–C_ = 9.1 Hz, CH), 133.7 (d, *J*_P–C_ = 12.7 Hz, CH), 133.4 (d, *J*_P–C_ = 9.1 Hz, CH), 132.4 (d, *J*_P–C_ = 10.0 Hz, CH), 131.9 (d, *J*_P–C_ = 2.7 Hz, CH), 131.8 (d, *J*_P–C_ = 2.7 Hz, CH), 131.55 (d, *J*_P–C_ = 10.9 Hz, CH), 131.51 (d, *J*_P–C_ = 7.3 Hz, CH), 130.4 (d, *J*_P–C_ = 105.4 Hz, C), 128.7 (d, *J*_P–C_ = 11.8 Hz, CH), 128.3 (d, *J*_P–C_ = 12.7 Hz, CH), 124.9 (d, *J*_P–C_ = 12.7 Hz, CH), 124.9 (d, *J*_P–C_ = 7.3 Hz, C), 84.7 (s, C), 81.4 (d, *J*_P–C_ = 5.5 Hz, CH), 21.7 (s, CH_3_). ^31^P NMR (202 MHz, CDCl_3_): δ 30.71 (s). GC–MS (EI) *m*/*z*: 317 (8), 316 (48) (M)^+^, 315 (100), 237 (17), 220 (11), 191 (16), 189 (14), 178 (11), 165 (21), 77 (11). HRMS (ESI/Q-TOF) *m*/*z*: calcd for C_21_H_17_OP [2M + Na]^+^, 655.1926; found, 655.1929.

#### [2-(Ethynyl)phenyl]diphenylphosphine Oxide (**8d**)

Compound **5a** (0.0486 g, 0.13 mmol) was reacted with PhMgBr (0.195 mL, 0.195 mmol, 1 M solution in THF) according to general procedure E for 30 min to afford **8d** (0.0373 g, 0.124 mmol, 95% yield) as a yellowish solid. Mp: 181.9–182.9 °C. *R*_*f*_ = 0.59 (2:1 CHCl_3_/acetone). ^1^H NMR (500 MHz, CDCl_3_): δ 7.72–7.77 (m, 4H), 7.65–7.69 (m, 1H), 7.61–7.62 (m, 1H), 7.49–7.59 (m, 3H), 7.41–7.48 (m, 5H), 2.99 (s, 1H). ^13^C{^1^H} NMR (125 MHz, CDCl_3_): δ 134.9 (d, *J*_P–C_ = 9.1 Hz, CH), 134.7 (d, *J*_P–C_ = 100.8 Hz, C), 133.8 (d, *J*_P–C_ = 10.0 Hz, CH), 132.2 (d, *J*_P–C_ = 10.0 Hz, CH), 132.1 (d, *J*_P–C_ = 106.2 Hz, C), 131.8 (d, *J*_P–C_ = 2.7 Hz, CH), 131.6 (d, *J*_P–C_ = 2.7 Hz, CH), 128.5 (d, *J*_P–C_ = 11.8 Hz, CH), 128.3 (d, *J*_P–C_ = 12.7 Hz, CH), 125.3 (d, *J*_P–C_ = 6.4 Hz, C), 85.0 (s, C), 81.5 (d, *J*_P–C_ = 5.5 Hz, CH). ^31^P NMR (202 MHz, CDCl_3_): δ 28.49 (s). GC–MS (EI) *m*/*z*: 303 (12), 302 (50) (M)^+^, 301 (46), 254 (12), 253 (12), 252 (12), 225 (17), 196 (24), 183 (16), 178 (17), 176 (14), 165 (31), 152 (27), 151 (15), 107 (13), 101 (11), 78 (44), 77 (93), 76 (15), 75 (33), 74 (17). HRMS (ESI/Q-TOF) *m*/*z*: calcd for C_20_H_15_OP [M + Na]^+^, 325.0753; found, 325.0754.

#### *p*-Anisyl(2-ethynylphenyl)phenylphosphine Oxide (**8e**)

Compound **5a** (0.0486 g, 0.13 mmol) was reacted with *p*-AnMgBr (0.39 mL, 0.195 mmol, 0.5 M solution in THF) according to general procedure E for 30 min to afford **8e** (0.0419 g, 0.127 mmol, 98% yield) as a yellowish solid. Mp: 149.5–150.5 °C. *R*_*f*_ = 0.58 (2:1 CHCl_3_/acetone). ^1^H NMR (500 MHz, CDCl_3_): δ 7.65–7.76 (m, 5H), 7.61–7.62 (m, 1H), 7.49–7.51 (m, 2H), 7.41–7.48 (m, 3H), 6.97–6.99 (m, 2H), 3.89 (s, 3H), 3.01 (s, 1H). ^13^C{^1^H} NMR (125 MHz, CDCl_3_): δ 162.5 (d, *J*_P–C_ = 2.7 Hz, C), 134.9 (d, *J*_P–C_ = 9.1 Hz, CH), 134.7 (d, *J*_P–C_ = 100.8 Hz, C), 134.2 (d, *J*_P–C_ = 10.9 Hz, 2CH), 133.8 (d, *J*_P–C_ = 9.1 Hz, CH), 132.3 (d, *J*_P–C_ = 107.2 Hz, C), 132.2 (d, *J*_P–C_ = 10.0 Hz, 2CH), 131.8 (d, *J*_P–C_ = 2.7 Hz, CH), 131.7 (d, *J*_P–C_ = 2.7 Hz, CH), 128.5 (d, *J*_P–C_ = 10.9 Hz, CH), 128.3 (d, *J*_P–C_ = 11.8 Hz, 2CH), 125.2 (d, *J*_P–C_ = 7.3 Hz, C), 122.8 (d, *J*_P–C_ = 112.6 Hz, C), 113.9 (d, *J*_P–C_ = 13.6 Hz, 2CH), 85.0 (s, C), 81.6 (d, *J*_P–C_ = 5.5 Hz, CH), 55.3 (s, CH_3_). ^31^P{^1^H} NMR (202 MHz, CDCl_3_): δ 28.15 (s). GC–MS (EI) *m*/*z*: 333 (22), 332 (100) (M)^+^, 331 (72), 317 (19), 284 (14), 270 (15), 255 (24), 253 (14), 252 (12), 241 (11), 240 (10), 239 (20), 231 (10), 226 (15), 196 (15), 194 (15), 184 (11), 183 (36), 178 (13), 176 (17), 170 (16), 169 (10), 165 (52), 163 (12), 157 (10), 152 (30), 151 (12), 141 (12), 139 (18), 125 (25), 120 (15), 15 (15), 108 (11), 107 (19), 101 (10), 95 (17), 92 (41), 78 (34), 77 (34), 76 (18). HRMS (ESI/Q-TOF) *m*/*z*: calcd for C_21_H_17_O_2_P [M + Na]^+^, 355.0856; found, 355.0856.

#### *m*-Anisyl(2-ethynylphenyl)phenylphosphine Oxide (**8f**)

Compound **5a** (0.0486 g, 0.13 mmol) was reacted with *m*-AnMgBr (0.195 mL, 0.195 mmol, 1 M solution in THF) according to general procedure E for 30 min to afford **8f** (0.0385 g, 0.116 mmol, 89% yield) as a yellow solid. Mp: 129–130 °C. *R*_*f*_ = 0.69 (2:1 CHCl_3_/acetone). ^1^H NMR (500 MHz, CDCl_3_): δ 7.71–7.77 (m, 2H), 7.60–7.64 (m, 2H), 7.50–7.57 (m, 2H), 7.44–7.49 (m, 3H), 7.39–7.43 (m, 2H), 7.23–7.27 (m, 1H), 7.07–7.09 (m, 1H), 3.80 (s, 3H), 3.02 (s, 1H). ^13^C{^1^H} NMR (125 MHz, CDCl_3_): δ 159.4 (d, *J*_P–C_ = 14.5 Hz, C), 134.9 (d, *J*_P–C_ = 9.1 Hz, CH), 133.8 (d, *J*_P–C_ = 9.9 Hz, CH), 133.3 (d, *J*_P–C_ = 105.4 Hz, C), 132.1 (d, *J*_P–C_ = 9.9 Hz, 2CH), 132.0 (d, *J*_P–C_ = 103.5 Hz, C), 131.8 (d, *J*_P–C_ = 3.6 Hz, CH), 131.7 (d, *J*_P–C_ = 1.8 Hz, CH), 129.5 (d, *J*_P–C_ = 15.4 Hz, CH), 128.4 (d, *J*_P–C_ = 9.9 Hz, CH), 128.3 (d, *J*_P–C_ = 12.7 Hz, 2CH), 125.3 (d, *J*_P–C_ = 6.4 Hz, C), 124.5 (d, *J*_P–C_ = 10.0 Hz, CH), 118.1 (d, *J*_P–C_ = 2.7 Hz, CH), 116.9 (d, *J*_P–C_ = 10.9 Hz, CH), 85.1 (s, C), 81.5 (d, *J*_P–C_ = 6.4 Hz, CH), 55.4 (s, OCH_3_). ^31^P{^1^H} NMR (202 MHz, CDCl_3_): δ 28.82 (s). GC–MS (EI) *m*/*z*: 334 (5), 333 (19), 332 (82) (M)^+^, 331 (83), 317 (21), 316 (11), 285 (11), 284 (16), 281 (17), 271 (10), 270 (20), 269 (13), 254 (16), 253 (24), 251 (16), 240 (12), 238 (29), 226 (18), 225 (18), 223 (16), 208 (11), 207 (40), 199 (10), 196 (21), 195 (13). HRMS (ESI/Q-TOF) *m*/*z*: calcd for C_21_H_17_O_2_P [M + Na]^+^, 355.0858; found, 355.0860.

#### *o*-Anisyl(2-ethynylphenyl)phenylphosphine Oxide (**8g**)

Compound **5a** (0.0486 g, 0.13 mmol) was reacted with *o*-AnMgBr (0.195 mL, 0.195 mmol, 1 M solution in THF) according to general procedure E for 30 min to afford **8g** (0.0397 g, 0.119 mmol, 92% yield) as a yellow solid. Mp: 156.7–157.7 °C. *R*_*f*_ = 0.54 (2:1 CHCl_3_/acetone). ^1^H NMR (500 MHz, CDCl_3_): δ 7.84–7.88 (m, 2H), 7.70–7.74 (m, 1H), 7.61–7.66 (m, 1H), 7.56–7.58 (m, 1H), 7.48–7.54 (m, 2H), 7.42–7.46 (m, 3H), 7.36–7.39 (m, 1H), 7.03–7.06 (m, 1H), 6.89–6.92 (m, 1H), 3.54 (s, 3H), 2.89 (s, 1H). ^13^C{^1^H} NMR (125 MHz, CDCl_3_): δ 161.1 (d, *J*_P–C_ = 3.6 Hz, C), 134.9 (d, *J*_P–C_ = 7.3 Hz, CH), 134.4 (d, *J*_P–C_ = 9.1 Hz, CH), 134.1 (d, *J*_P–C_ = 1.9 Hz, CH), 132.9 (d, *J*_P–C_ = 10.0 Hz, CH), 132.2 (d, *J*_P–C_ = 10.0 Hz, 2CH), 131.4 (d, *J*_P–C_ = 1.8 Hz, CH), 130.9 (d, *J*_P–C_ = 1.9 Hz, CH), 128.2 (d, *J*_P–C_ = 11.8 Hz, CH), 127.9 (d, *J*_P–C_ = 12.7 Hz, 2CH), 124.5 (d, *J*_P–C_ = 6.4 Hz, C), 120.8 (d, *J*_P–C_ = 11.8 Hz, CH), 111.7 (d, *J*_P–C_ = 6.4 Hz, CH), 84.0 (s, C), 81.4 (d, *J*_P–C_ = 6.4 Hz, CH), 55.3 (s, CH_3_). ^31^P{^1^H} NMR (202 MHz, CDCl_3_): δ 26.19 (s). GC–MS (EI) *m*/*z*: 333 (10), 332 (42) (M)^+^, 331 (55), 254 (12), 253 (12), 252 (12), 225 (17), 196 (24), 183 (16), 178 (17), 176 (14), 165 (31), 152 (27), 151 (15), 107 (13), 101 (11), 78 (44), 77 (93), 76 (15), 75 (33), 74 (17). HRMS (ESI/Q-TOF) *m*/*z*: calcd for C_21_H_17_O_2_P [M + Na]^+^, 355.0858; found, 355.0851.

#### 2-Ethynylphenyl(9-phenanthryl)phenylphosphine Oxide (**8h**)

Compound **5a** (0.0486 g, 0.13 mmol) was reacted with 9-phenanthryl magnesium bromide (0.39 mL, 0.195 mmol, 0.5 M solution in THF) according to general procedure E for 30 min to afford **8h** (0.0366 g, 0.091 mmol, 70% yield) as a yellow solid. Mp: 192–193 °C (dec.). *R*_*f*_ = 0.25 (100:1 CHCl_3_/MTBE). ^1^H NMR (500 MHz, CDCl_3_): δ 8.86–8.75 (m, 3H), 7.79–7.89 (m, 3H), 7.73–7.78 (m, 3H), 7.65–7.68 (m, 1H), 7.62–7.64 (m, 1H), 7.57–7.60 (m, 2H), 7.42–7.55 (m, 5H), 2.84 (s, 1H). ^13^C{^1^H} NMR (125 MHz, CDCl_3_): δ 137.0 (d, *J*_P–C_ = 11.8 Hz, CH), 135.0 (d, *J*_P–C_ = 9.1 Hz, CH), 134.8 (d, *J*_P–C_ = 99.0 Hz, C), 133.7 (d, *J*_P–C_ = 10.0 Hz, CH), 132.6 (d, *J*_P–C_ = 10.0 Hz, CH), 132.1 (d, *J*_P–C_ = 2.7 Hz, C), 132.0 (d, *J*_P–C_ = 2.7 Hz, CH), 131.9 (d, *J*_P–C_ = 106.3 Hz, C), 131.7 (d, *J*_P–C_ = 1.8 Hz, CH), 131.2 (d, *J*_P–C_ = 9.1 Hz, C), 130.5 (d, *J*_P–C_ = 9.1 Hz, C), 130.1 (s, CH), 129.7 (d, *J*_P–C_ = 14.5 Hz, C), 129.0 (s, CH), 128.6 (d, *J*_P–C_ = 11.8 Hz, CH), 128.5 (d, *J*_P–C_ = 2.7 Hz, CH), 128.4 (d, *J*_P–C_ = 12.7 Hz, CH), 127.2 (d, *J*_P–C_ = 106.3 Hz, C), 127.1 (d, *J*_P–C_ = 15.4 Hz, CH), 126.9 (s, CH), 125.4 (d, *J*_P–C_ = 6.4 Hz, C), 123.0 (s, CH), 122.7 (s, CH), 84.7 (s, C), 81.5 (d, *J*_P–C_ = 5.5 Hz, CH). ^31^P{^1^H} NMR (202 MHz, CDCl_3_): δ 31.94 (s). HRMS (ESI/Q-TOF) *m*/*z*: calcd for C_28_H_19_OP [2M + Na]^+^, 827.2239; found, 827.2228.

#### *p*-Chlorophenyl(2-ethynylphenyl)phenylphosphine Oxide (**8i**)

Compound **5a** (0.0486 g, 0.13 mmol) was reacted with *p*-Cl–C_6_H_4_MgBr (0.195 mL, 0.195 mmol, 1 M solution in THF) according to general procedure E for 30 min to afford **8i** (0.0366 g, 0.107 mmol, 82% yield) as a yellow solid. Mp: 117.5–118 °C. *R*_*f*_ = 0.55 (2:1 CHCl_3_/acetone). ^1^H NMR (500 MHz, CDCl_3_): δ 7.67–7.75 (m, 5H), 7.62–7.65 (m, 1H), 7.51–7.59 (m, 2H), 7.42–7.49 (m, 5H), 3.02 (s, 1H). ^13^C{^1^H} NMR (125 MHz, CDCl_3_): δ 138.4 (d, *J*_P–C_ = 3.6 Hz, C), 135.0 (d, *J*_P–C_ = 9.1 Hz, CH), 134.1 (d, *J*_P–C_ = 102.6 Hz, C), 133.8 (d, *J*_P–C_ = 9.1 Hz, CH), 133.6 (d, *J*_P–C_ = 10.9 Hz, CH), 132.15 (d, *J*_P–C_ = 10.0 Hz, CH), 132.12 (d, *J*_P–C_ = 3.1 Hz, CH), 131.9 (d, *J*_P–C_ = 2.5 Hz, CH), 131.0 (d, *J*_P–C_ = 106.3 Hz, C), 128.6 (d, *J*_P–C_ = 12.7 Hz, CH), 128.5 (d, *J*_P–C_ = 11.8 Hz, CH), 128.4 (d, *J*_P–C_ = 11.8 Hz, CH), 125.2 (d, *J*_P–C_ = 6.4 Hz, C), 85.3 (s, C), 81.5 (d, *J*_P–C_ = 6.4 Hz, CH). ^31^P{^1^H} NMR (202 MHz, CDCl_3_): δ 27.61 (s). GC–MS (EI) *m*/*z*: 338 (17), 337 (32), 336 (61) (M)^+^, 335 (60), 250 (21), 253 (19), 252 (23), 225 (14), 212 (10), 199 (12), 196 (31), 194 (13), 183 (16), 178 (18), 176 (28), 169 (13), 165 (30), 152 (37), 150 (25), 119 (12). HRMS (ESI/Q-TOF) *m*/*z*: calcd for C_20_H_14_ClOP [M + H]^+^, 337.0544; found, 337.0537.

#### 2-Ethynylphenyl(*p*-fluorophenyl)phenylphosphine Oxide (**8j**)

Compound **5a** (0.0486 g, 0.13 mmol) was reacted with *p*–F-C_6_H_4_MgBr (0.195 mL, 0.195 mmol, 1 M solution in THF) according to general procedure E for 30 min to afford **8j** (0.034 g, 0.107 mmol, 82% yield) as a yellow oil. *R*_*f*_ = 0.52 (30:5:1 CHCl_3_/EtOAc/MeOH). ^1^H NMR (500 MHz, CDCl_3_): δ 7.68–7.77 (m, 5H), 7.61–7.64 (m, 1H), 7.51–7.58 (m, 2H), 7.42–7.49 (m, 3H), 7.13–7.17 (m, 2H), 3.01 (s, 1H). ^13^C{^1^H} NMR (125 MHz, CDCl_3_): δ 165.1 (dd, *J*_P–C_ = 3.6 Hz, *J*_F–C_= 237.0 Hz, CF), 134.9 (d, *J*_P–C_ = 8.2 Hz, CH), 134.7 (dd, *J*_P–C_ = 9.1 Hz, *J*_F–C_= 11.8 Hz, 2CH), 134.2 (d, *J*_P–C_ = 100.8 Hz, C), 133.7 (d, *J*_P–C_ = 9.1 Hz, CH), 132.1 (d, *J*_P–C_ = 10.0 Hz, 2CH), 132.0 (d, *J*_P–C_ = 2.7 Hz, CH), 131.9 (d, *J*_P–C_ = 2.7 Hz, CH), 131.8 (d, *J*_P–C_ = 108.1 Hz, C), 128.6 (d, *J*_P–C_ = 11.8 Hz, CH), 128.4 (d, *J*_P–C_ = 11.8 Hz, 2CH), 128.1 (dd, *J*_F–C_= 2.7 Hz, *J*_P–C_ = 108.9 Hz, C), 125.2 (d, *J*_P–C_ = 6.4 Hz, C), 115.7 (dd, *J* = 20.9 Hz, *J*_P–C_ = 13.6 Hz, CH), 85.2 (s, C), 81.5 (d, *J*_P–C_ = 5.5 Hz, CH). ^31^P{^1^H} NMR (202 MHz, CDCl_3_): δ 27.61 (s). GC–MS (EI) *m*/*z*: 321 (14), 320 (58) (M)^+^, 319 (68), 300 (16), 273 (15), 272 (20), 271 (19), 270 (15), 252 (11), 242 (18), 245 (18), 214 (23), 201 (20), 196 (28), 194 (20), 183 (20), 178 (11), 176 (17), 170 (19), 165 (26), 152 (26), 151 (13), 150 (16), 126 (11), 107 (17). HRMS (ESI/Q-TOF) *m*/*z*: calcd for C_20_H_14_FOP [M + H]^+^, 321.0839; found, 321.0841.

#### (2-Ethynylphenyl)phenyl[*p*-(*N*,*N-*dimethylamino)phenyl]phosphine Oxide (**8k**)

Compound **5a** (0.0486 g, 0.13 mmol) was reacted with *p*-Me_2_N–C_6_H_4_MgBr (0.39 mL, 0.195 mmol, 0.5 M solution in THF) according to general procedure E for 30 min to afford **8k** (0.0269 g, 0.078 mmol, 60% yield) as a yellow solid. Mp: 194.9–195.7 °C. *R*_*f*_ = 0.42 (30:5:1 CHCl_3_/EtOAc/MeOH). ^1^H NMR (500 MHz, CDCl_3_): δ 7.68–7.74 (m, 3H), 7.58–7.61 (m, 1H), 7.53–7.58 (m, 2H), 7.48–7.52 (m, 1H), 7.38–7.47 (m, 4H), 6.70–6.72 (m, 2H), 3.02 (s, 6H), 3.02 (s, 1H). ^13^C{^1^H} NMR (125 MHz, CDCl_3_): δ 152.3 (d, *J*_P–C_ = 2.7 Hz, C), 135.8 (d, *J*_P–C_ = 100.8 Hz, C), 134.8 (d, *J*_P–C_ = 9.1 Hz, CH), 133.8 (d, *J*_P–C_ = 9.1 Hz, CH), 133.6 (d, *J*_P–C_ = 10.9 Hz, 2CH), 133.3 (d, *J*_P–C_ = 106.3 Hz, C), 132.1 (d, *J*_P–C_ = 10.0 Hz, 2CH), 131.3 (d, *J*_P–C_ = 2.7 Hz, CH), 131.2 (d, *J*_P–C_ = 1.8 Hz, CH), 128.3 (d, *J*_P–C_ = 11.8 Hz, CH), 128.1 (d, *J*_P–C_ = 11.8 Hz, 2CH), 125.2 (d, *J*_P–C_ = 6.4 Hz, C), 116.3 (d, *J*_P–C_ = 118.1 Hz, C), 111.1 (d, *J*_P–C_ = 12.7 Hz, 2CH), 84.6 (s, C), 81.8 (d, *J*_P–C_ = 5.5 Hz, CH), 39.9 (s, 2CH_3_). ^31^P{^1^H} NMR (202 MHz, CDCl_3_): δ 28.92 (s). GC–MS (EI) *m*/*z*: 346 (18), 345 (100) (M)^+^, 344 (32), 330 (15), 329 (11), 252 (16), 183 (10), 152 (12), 136 (13), 77 (12), 51 (10). HRMS (ESI/Q-TOF) *m*/*z*: calcd for C_22_H_20_NOP [M + H]^+^, 346.1355; found, 346.1360.

#### (2-Ethynyl-6-methylphenyl)phenyl(*p*-tolyl)phosphine Oxide (**10a**)

Compound **5b** (0.0505 g, 0.13 mmol) was reacted with *p*-TolMgBr (0.195 mL, 0.195 mmol, 1 M solution in THF) according to general procedure E for 1 h to afford **10a** (0.0283 g, 0.086 mmol, 66% yield) as a yellowish oil. *R*_*f*_ = 0.15 (200:1 CHCl_3_/MTBE). ^1^H NMR (500 MHz, CDCl_3_): δ 7.68–7.72 (m, 2H), 7.59–7.63 (m, 2H), 7.50–7.54 (m, 1H), 7.39–7.43 (m, 3H), 7.33–7.36 (m, 1H), 7.24–7.27 (m, 3H), 2.61 (s, 1H), 2.59 (s, 3H), 2.41 (s, 3H). ^13^C{^1^H} NMR (125 MHz, CDCl_3_): δ 145.5 (d, *J*_P–C_ = 7.3 Hz, C), 142.3 (d, *J*_P–C_ = 3.6 Hz, C), 134.4 (d, *J*_P–C_ = 105.4 Hz, C), 133.6 (d, *J*_P–C_ = 9.1 Hz, CH), 133.1 (d, *J*_P–C_ = 10.0 Hz, CH), 132.1 (d, *J*_P–C_ = 10.0 Hz, 4CH), 131.6 (d, *J*_P–C_ = 2.7 Hz, CH), 131.1 (d, *J*_P–C_ = 2.7 Hz, CH), 130.5 (d, *J*_P–C_ = 107.2 Hz, C), 129.1 (d, *J*_P–C_ = 12.7 Hz, 2CH), 129.1 (d, *J*_P–C_ = 11.8 Hz, 2CH), 125.7 (d, *J*_P–C_ = 9.1 Hz, C), 84.6 (s, C), 82.1 (d, *J*_P–C_ = 6.4 Hz, CH), 23.3 (d, *J*_P–C_ = 4.5 Hz, CH), 21.6 (s, CH_3_). ^31^P{^1^H} NMR (202 MHz, CDCl_3_): δ 30.67 (s). GC–MS (EI) *m*/*z*: 330 (1) (M)^+^, 329 (1), 305 (19), 304 (100), 303 (55), 302 (18), 289 (14), 288 (22), 273 (18), 258 (11), 257 (36), 256 (16), 242 (29), 241 (24), 240 (14), 239 (22), 227 (21), 213 (19), 197 (18), 196 (18), 194 (17), 183 (49), 178 (11), 170 (12), 166 (15), 165 (67), 164 (11), 163 (14), 152 (34), 139 (17), 91 (42), 89 (25), 78 (23), 77 (70). HRMS (ESI/Q-TOF) *m*/*z*: calcd for C_22_H_19_OP [M + H]^+^, 331.1246; found, 331.1236.

#### (2-Ethynyl-6-methylphenyl]diphenylphosphine Oxide (**10d**)

Compound **5b** (0.0505 g, 0.13 mmol) was reacted with PhMgBr (0.195 mL, 0.195 mmol, 1 M solution in THF) according to general procedure E for 1 h to afford **10d** (0.0374 g, 0.118 mmol, 91% yield) as a yellowish oil. *R*_*f*_ = 0.15 (200:1 CHCl_3_/MTBE). ^1^H NMR (500 MHz, CDCl_3_): δ 7.70–7.74 (m, 4H), 7.52–7.55 (m, 2H), 7.40–7.46 (m, 5H), 7.33–7.38 (m, 1H), 7.27–7.30 (m, 1H), 2.61 (s, 1H), 2.60 (s, 3H). ^13^C{^1^H} NMR (125 MHz, CDCl_3_): δ 145.6 (d, *J*_P–C_ = 7.3 Hz, C), 134.4 (d, *J*_P–C_ = 1.8 Hz, C), 133.7 (d, *J*_P–C_ = 9.1 Hz, CH), 133.1 (d, *J*_P–C_ = 9.1 Hz, CH), 132.1 (d, *J*_P–C_ = 10.9 Hz, CH), 131.8 (d, *J*_P–C_ = 2.7 Hz, CH), 131.1 (d, *J*_P–C_ = 1.8 Hz, CH), 128.3 (d, *J*_P–C_ = 12.7 Hz, 2CH), 125.7 (d, *J*_P–C_ = 8.2 Hz, C), 84.6 (s, C), 82.0 (d, *J*_P–C_ = 6.4 Hz, CH), 23.3 (d, *J*_P–C_ = 3.6 Hz, CH_3_). ^31^P{^1^H} NMR (202 MHz, CDCl_3_): δ 30.62 (s). GC–MS (EI) *m*/*z*: 317 (5) (M)^+^, 315 (4), 291 (11), 290 (95), 289 (53), 274 (17), 259 (11), 243 (28), 243 (13), 241 (13), 239 (13), 228 (24), 212 (34), 197 (11), 194 (17), 183 (45), 165 (51), 139 (18), 77 (100). HRMS (ESI/Q-TOF) *m*/*z*: calcd for C_21_H_17_OP [M + H]^+^, 317.1090; found, 317.1082.

#### (*p*-Anisyl)(2-ethynyl-6-methylphenyl]phenylphosphine Oxide (**10e**)

Compound **5b** (0.0505 g, 0.13 mmol) was reacted with *p*-AnMgBr (0.39 mL, 0.195 mmol, 0.5 M solution in THF) according to general procedure E for 1 h to afford **10e** (0.0266 g, 0.0767 mmol, 59% yield) as a yellowish oil. *R*_*f*_ = 0.15 (200:1 CHCl_3_/MTBE). ^1^H NMR (500 MHz, CDCl_3_): δ 7.62–7.72 (m, 4H), 7.50–7.53 (m, 1H), 7.40–7.44 (m, 3H), 7.33–7.38 (m, 1H), 7.25–7.27 (m, 1H), 6.94–6.96 (m, 2H), 3.85 (m, 3H), 2.66 (s, 1H), 2.59 (s, 3H). ^13^C{^1^H} NMR (125 MHz, CDCl_3_): δ 162.5 (d, *J*_P–C_ = 3.6 Hz, C), 145.5 (d, *J*_P–C_ = 7.3 Hz, C), 134.7 (d, *J*_P–C_ = 108.1 Hz, C), 134.1 (d, *J*_P–C_ = 10.9 Hz, 2CH), 133.7 (d, *J*_P–C_ = 9.1 Hz, CH), 133.0 (d, *J*_P–C_ = 10.0 Hz, CH), 132.5 (d, *J*_P–C_ = 99.9 Hz, C), 131.9 (d, *J*_P–C_ = 10.0 Hz, 2CH), 131.6 (d, *J*_P–C_ = 2.7 Hz, CH), 130.9 (d, *J*_P–C_ = 1.8 Hz, CH), 128.3 (d, *J*_P–C_ = 11.8 Hz, 2CH), 125.6 (d, *J*_P–C_ = 6.4 Hz, C), 113.2 (d, *J*_P–C_ = 13.6 Hz, 2CH), 84.4 (s, C), 82.2 (d, *J*_P–C_ = 6.4 Hz, CH), 56.3 (s, CH_3_), 23.3 (d, *J*_P–C_ = 3.6 Hz, CH_3_). ^31^P{^1^H} NMR (202 MHz, CDCl_3_): δ 30.21 (s). HRMS (ESI/Q-TOF) *m*/*z*: calcd for C_22_H_19_O_2_P [M + H]^+^, 347.1195; found, 347.1205.

#### (2-Ethynyl-6-methylphenyl)(4-chlorophenyl)phenylphosphine Oxide (**10i**)

Compound **5b** (0.0505 g, 0.13 mmol) was reacted with *p*-Cl–C_6_H_4_MgBr (0.195 mL, 0.195 mmol, 1 M solution in THF) according to general procedure E for 1 h to afford **10i** (0.0296 g, 0.0845 mmol, 65% yield) as a yellowish oil. *R*_*f*_ = 0.4 (30:5:1 CHCl_3_/AcOEt/MeOH). ^1^H NMR (500 MHz, CDCl_3_): δ 7.69–7.73 (m, 2H), 7.63–7.67 (m, 2H), 7.54–7.57 (m, 1H), 7.40–7.47 (m, 5H), 7.36–7.38 (m, 1H), 7.27–7.29 (m, 1H), 2.67 (m, 1H), 2.59 (s, 3H). ^13^C{^1^H} NMR (125 MHz, CDCl_3_): δ 145.6 (d, *J*_P–C_ = 8.2 Hz, C), 138.3 (d, *J*_P–C_ = 3.6 Hz, C), 133.7 (d, *J*_P–C_ = 9.1 Hz, CH), 133.6 (d, *J*_P–C_ = 102.6 Hz, C), 133.5 (d, *J*_P–C_ = 10.9 Hz, 2CH), 133.2 (d, *J*_P–C_ = 10.0 Hz, CH), 132.9 (d, *J*_P–C_ = 106.3 Hz, C), 131.9 (d, *J*_P–C_ = 2.7 Hz, CH), 131.8 (d, *J*_P–C_ = 10.0 Hz, 2CH), 131.3 (d, *J*_P–C_ = 1.8 Hz, CH), 128.6 (d, *J*_P–C_ = 12.7 Hz, 2CH), 128.5 (d, *J*_P–C_ = 12.7 Hz, 2CH), 125.6 (d, *J*_P–C_ = 8.2 Hz, C), 85.1 (s, C), 82.1 (d, *J*_P–C_ = 6.4 Hz, CH), 23.3 (d, *J*_P–C_ = 3.6 Hz, CH_3_). ^31^P{^1^H} NMR (202 MHz, CDCl_3_): δ 29.69 (s). HRMS (ESI/Q-TOF) *m*/*z*: calcd for C_21_H_16_ClOP [M + H]^+^, 351.0700; found, 351.0694.

#### (2-Ethynyl-6-methylphenyl)[*p*-(*N*,*N-*dimethylamino)phenyl]phenylphosphine Oxide (**10k**)

Compound **5b** (0.0505 g, 0.13 mmol) was reacted with *p*-Me_2_N–C_6_H_4_MgBr (0.39 mL, 0.195 mmol, 0.5 M solution in THF) according to general procedure E for 1 h to afford **10k** (0.0327 g, 0.091 mmol, 70% yield) as a brownish waxy solid. Mp: 140.5–144.5 °C. *R*_*f*_ = 0.21 (10:1 CHCl_3_/MTBE). ^1^H NMR (500 MHz, CDCl_3_): δ 7.66–7.70 (m, 2H), 7.51–7.55 (m, 2H), 7.47–7.50 (m, 1H), 7.38–7.41 (m, 3H), 7.30–7.33 (m, 1H), 7.23–7.30 (m, 1H), 6.69–6.71 (m, 2H), 3.01 (s, 6H), 2.67 (s, 1H), 2.60 (m, 3H). ^13^C{^1^H} NMR (125 MHz, CDCl_3_): δ 152.5 (d, *J*_P–C_ = 2.7 Hz, C), 145.3 (d, *J*_P–C_ = 7.3 Hz, C), 135.6 (d, *J*_P–C_ = 105.4 Hz, C), 133.6 (d, *J*_P–C_ = 9.1 Hz, CH), 133.5 (d, *J*_P–C_ = 11.8 Hz, 2CH), 133.2 (d, *J*_P–C_ = 99.0 Hz, C), 132.9 (d, *J*_P–C_ = 10.0 Hz, CH), 131.9 (d, *J*_P–C_ = 10.0 Hz, 2CH), 131.3 (d, *J*_P–C_ = 3.6 Hz, CH), 130.6 (d, *J*_P–C_ = 1.8 Hz, CH), 128.6 (d, *J*_P–C_ = 101.7 Hz, C), 128.1 (d, *J*_P–C_ = 12.7 Hz, 2CH), 123.1 (d, *J*_P–C_ = 8.2 Hz, C), 111.2 (d, *J*_P–C_ = 12.7 Hz, 2CH), 84.1 (s, C), 82.3 (d, *J*_P–C_ = 6.4 Hz, CH), 39.9 (s, 2CH_3_), 23.3 (d, *J*_P–C_ = 4.5 Hz, CH_3_). ^31^P{^1^H} NMR (202 MHz, CDCl_3_): δ 31.06 (s). HRMS (ESI/Q-TOF) *m*/*z*: calcd for C_23_H_22_NOP [M + H]^+^, 360.1512; found, 360.1515.

#### (2-Ethynyl-5-chlorophenyl)phenyl(*p*-tolyl)phosphine Oxide (**11a**)

Compound **5c** (0.053 g, 0.13 mmol) was reacted with *p*-TolMgBr (0.195 mL, 0.195 mmol, 1 M solution in THF) according to general procedure E for 1 h to afford **11a** (0.0301 g, 0.086 mmol, 66% yield) as an orange solid. Mp: 160.4–161.8 °C. *R*_*f*_ = 0.45 (30:5:1 CHCl_3_/AcOEt/MeOH). ^1^H NMR (500 MHz, CDCl_3_): δ 7.71–7.75 (m, 3H), 7.61–7.66 (m, 2H), 7.52–7.56 (m, 2H), 7.45–7.49 (m, 3H), 7.27–7.30 (m, 2H), 3.02 (m, 1H), 2.43 (m, 3H). ^13^C{^1^H} NMR (125 MHz, CDCl_3_): δ 142.8 (d, *J*_P–C_ = 2.7 Hz, C), 137.3 (d, *J*_P–C_ = 97.2 Hz, C), 136.1 (d, *J*_P–C_ = 10.0 Hz, CH), 136.1 (d, *J*_P–C_ = 14.5 Hz, C), 133.7 (d, *J*_P–C_ = 10.0 Hz, CH), 132.3 (d, *J*_P–C_ = 10.0 Hz, 2CH), 132.2 (d, *J*_P–C_ = 10.0 Hz, 2CH), 132.1 (d, *J*_P–C_ = 2.7 Hz, CH), 131.9 (d, *J*_P–C_ = 108.9 Hz, C), 131.8 (d, *J*_P–C_ = 2.7 Hz, CH), 129.3 (d, *J*_P–C_ = 12.7 Hz, 2CH), 128.4 (d, *J*_P–C_ = 12.7 Hz, 2CH), 128.1 (d, *J*_P–C_ = 109.9 Hz, C), 123.5 (d, *J*_P–C_ = 6.4 Hz, C), 85.9 (s, C), 80.8 (d, *J*_P–C_ = 5.5 Hz, CH). ^31^P{^1^H} NMR (202 MHz, CDCl_3_): δ 27.47 (s). GC–MS (EI) *m*/*z*: 353 (8), 352 (31), 351 (52), 350 (95) (M)^+^, 349 (91), 331 (8), 302 (12), 273 (27), 268 (25), 258 (13), 256 (12), 252 (23), 244 (17), 230 (14), 215 (22), 212 (14), 210 (13), 199 (13), 196 (15), 194 (22), 189 (29), 176 (25), 165 (31). HRMS (ESI/Q-TOF) *m*/*z*: calcd for C_21_H_16_ClOP [M + H]^+^, 351.0700; found, 351.0693.

#### (2-Ethynyl-5-chlorophenyl]diphenylphosphine Oxide (**11d**)

Compound **5c** (0.053 g, 0.13 mmol) was reacted with PhMgBr (0.195 mL, 0.195 mmol, 1 M solution in THF) according to general procedure E for 1 h to afford **11d** (0.032 g, 0.095 mmol, 73% yield) as an yellow oil. *R*_*f*_ = 0.48 (30:5:1 CHCl_3_/AcOEt/MeOH). ^1^H NMR (500 MHz, CDCl_3_): δ 7.73–7.77 (m, 5H), 7.53–7.59 (m, 3H), 7.46–7.50 (m, 5H), 3.01 (s, 1H). ^13^C{^1^H} NMR (125 MHz, CDCl_3_): δ 136.9 (d, *J*_P–C_ = 98.1 Hz, C), 136.1 (d, *J*_P–C_ = 10.0 Hz, CH), 135.2 (d, *J*_P–C_ = 14.5 Hz, C), 133.7 (d, *J*_P–C_ = 9.1 Hz, CH), 132.2 (d, *J*_P–C_ = 10.0 Hz, 4CH), 132.17 (d, *J*_P–C_ = 4.5 Hz, 2CH), 131.9 (d, *J*_P–C_ = 2.7 Hz, CH), 131.4 (d, *J*_P–C_ = 107.2 Hz, 2C), 128.5 (d, *J*_P–C_ = 12.7 Hz, 4CH), 123.5 (d, *J*_P–C_ = 6.4 Hz, C), 84.9 (s, C), 80.7 (d, *J*_P–C_ = 5.5 Hz, CH). ^31^P{^1^H} NMR (202 MHz, CDCl_3_): δ 27.46 (s). GC–MS (EI) *m*/*z*: 338 (14), 337 (33), 336 (46) (M)^+^, 335 (41), 259 (13), 254 (15), 252 (12), 230 (11), 207 (17), 199 (13), 196 (14), 194 (12), 176 (12), 154 (17), 152 (14), 135 (14), 100 (11), 99 (37), 98 (10), 78 (59), 77 (100), 76 (15), 75 (22), 74 (23), 51 (89). HRMS (ESI/Q-TOF) *m*/*z*: calcd for C_20_H_14_ClOP [M + H]^+^, 337.0544; found, 337.0543.

#### (*p*-Anisyl)(2-ethynyl-5-chlorophenyl]phenylphosphine Oxide (**11e**)

Compound **5c** (0.053 g, 0.13 mmol) was reacted with *p*-AnMgBr (0.39 mL, 0.195 mmol, 0.5 M solution in THF) according to general procedure E for 1 h to afford **11e** (0.0276 g, 0.0754 mmol, 58% yield) as an orange oil. *R*_*f*_ = 0.54 (30:5:1 CHCl_3_/AcOEt/MeOH). ^1^H NMR (500 MHz, CDCl_3_): δ 7.65–7.77 (m, 5H), 7.52–7.56 (m, 2H), 7.45–7.48 (m, 3H), 6.69–6.99 (m, 2H), 3.86 (s, 3H), 3.03 (s, 1H). ^13^C{^1^H} NMR (125 MHz, CDCl_3_): δ 162.7 (d, *J*_P–C_ = 3.6 Hz, C), 137.5 (d, *J*_P–C_ = 98.1 Hz, C), 136.2 (d, *J*_P–C_ = 10.0 Hz, CH), 135.2 (d, *J*_P–C_ = 14.5 Hz, C), 134.2 (d, *J*_P–C_ = 11.8 Hz, 2CH), 133.7 (d, *J*_P–C_ = 10.0 Hz, CH), 132.2 (d, *J*_P–C_ = 10.9 Hz, 2CH), 132.0 (d, *J*_P–C_ = 3.6 Hz, CH), 131.9 (d, *J*_P–C_ = 108.1 Hz, C), 131.8 (d, *J*_P–C_ = 2.7 Hz, CH), 128.4 (d, *J*_P–C_ = 12.7 Hz, 2CH), 123.4 (d, *J*_P–C_ = 6.4 Hz, C), 122.4 (d, *J*_P–C_ = 113.5 Hz, C), 114.1 (d, *J*_P–C_ = 13.6 Hz, 2CH), 85.6 (s, C), 80.8 (d, *J*_P–C_ = 4.5 Hz, CH), 55.4 (s, OCH_3_). ^31^P{^1^H} NMR (202 MHz, CDCl_3_): δ 27.26 (s). GC–MS (EI) *m*/*z*: 368 (27), 367 (48), 366 (100) (M)^+^, 365 (76), 353 (13), 351 (23), 289 (24), 283 (11), 252 (10), 245 (12), 239 (15), 217 (12), 199 (25), 182 (11), 176 (19), 170 (10), 152 (11), 151 (10), 141 (14), 139 (11), 138 (10), 123 (37), 110 (11), 108 (24), 107 (13). HRMS (ESI/Q-TOF) *m*/*z*: calcd for C_21_H_16_ClO_2_P [M + H]^+^, 367.0649; found, 367.0642.

#### (4-Chlorophenyl)(2-ethynyl-5-chlorophenyl]phenylphosphine Oxide (**11i**)

Compound **5c** (0.053 g, 0.13 mmol) was reacted with *p*-Cl–C_6_H_4_MgBr (0.195 mL, 0.195 mmol, 1 M solution in THF) according to general procedure E for 1 h to afford **11i** (0.0289 g, 0.078 mmol, 60% yield) as an yellow oil. *R*_*f*_ = 0.47 (30:5:1 CHCl_3_/AcOEt/MeOH). ^1^H NMR (500 MHz, CDCl_3_): δ 7.67–7.77 (m, 5H), 7.54–7.60 (m, 2H), 7.45–7.51 (m, 5H), 3.04 (s, 1H). ^13^C{^1^H} NMR (125 MHz, CDCl_3_): δ 138.7 (d, *J*_P–C_ = 3.6 Hz, C), 136.3 (d, *J*_P–C_ = 10.0 Hz, 2CH), 135.4 (d, *J*_P–C_ = 15.4 Hz, C), 133.7 (d, *J*_P–C_ = 10.0 Hz, CH), 133.6 (d, *J*_P–C_ = 10.9 Hz, 2CH), 132.4 (d, *J*_P–C_ = 1.8 Hz, CH), 132.1 (d, *J*_P–C_ = 1.8 Hz, CH), 132.1 (d, *J*_P–C_ = 10.0 Hz, 2CH), 128.8 (d, *J*_P–C_ = 13.6 Hz, 2CH), 128.6 (d, *J*_P–C_ = 12.7 Hz, 2CH), 123.3 (d, *J*_P–C_ = 6.4 Hz, C), 86.2 (s, C), 80.7 (d, *J*_P–C_ = 5.5 Hz, CH). ^31^P{^1^H} NMR (202 MHz, CDCl_3_): δ 26.56 (s). GC–MS (EI) *m*/*z*: 373 (13), 372 (18), 371 (59), 370 (100) (M)^+^, 369 (24), 355 (22), 354 (11), 334 (17), 325 (14), 297 (11), 288 (14), 283 (14), 266 (18), 264 (25), 252 (43), 243 (13), 241 (18), 235 (10), 230 (28), 216 (14), 207 (21), 199 (13), 197 (13), 196 (16), 177 (18), 174 (13), 175 (12), 174 (22), 166 (12), 159 (12), 152 (12), 137 (11), 136 (17), 133 (41), 126 (10), 119 (13), 113 (14), 112 (36), 111 (34), 108 (11), 107 (30), 99 (37), 98 (17), 78 (12), 77 (41), 76 (24), 75 (69), 74 (35), 72 (21). HRMS (ESI/Q-TOF) *m*/*z*: calcd for C_20_H_13_Cl_2_OP [M + H]^+^, 371.0154; found, 371.0145.

#### (2-Ethynyl-5-chlorophenyl)[*p*-(*N*,*N-*dimethylamino)phenyl]phenyl Phosphine Oxide (**11k**)

Compound **5c** (0.053 g, 0.13 mmol) was reacted with *p*-Me_2_N–C_6_H_4_MgBr (0.39 mL, 0.195 mmol, 0.5 M solution in THF) according to general procedure E for 1 h to afford **11k** (0.0296 g, 0.078 mmol, 60% yield) as an orange oil. *R*_*f*_ = 0.21 (10:1 CHCl_3_/MTBE). ^1^H NMR (500 MHz, CDCl_3_): δ 7.77–7.78 (m, 1H), 7.70–7.74 (m, 2H), 7.50–7.58 (m, 4H), 7.43–7.46 (m, 3H), 6.72–6.74 (m, 2H), 3.03 (s, 7H). ^13^C{^1^H} NMR (125 MHz, CDCl_3_): δ 152.4 (d, *J*_P–C_ = 1.8 Hz, C), 138.1 (d, *J*_P–C_ = 92.6 Hz, C), 135.9 (d, *J*_P–C_ = 10.5 Hz, CH), 134.9 (d, *J*_P–C_ = 14.5 Hz, CH), 133.6 (d, *J*_P–C_ = 10.0 Hz, CH), 133.5 (d, *J*_P–C_ = 10.9 Hz, 2CH), 132.6 (d, *J*_P–C_ = 107.2 Hz, C), 132.1 (d, *J*_P–C_ = 10.0 Hz, 2CH), 131.7 (d, *J*_P–C_ = 2.7 Hz, CH), 131.4 (d, *J*_P–C_ = 1.8 Hz, CH), 128.2 (d, *J*_P–C_ = 12.7 Hz, 2CH), 123.1 (d, *J*_P–C_ = 5.5 Hz, C), 115.2 (d, *J*_P–C_ = 118.0 Hz, C), 111.1 (d, *J*_P–C_ = 13.6 Hz, 2C), 85.5 (s, C), 80.9 (d, *J*_P–C_ = 5.5 Hz, CH), 39.9 (s, 2CH_3_).^31^P{^1^H} NMR (202 MHz, CDCl_3_): δ 27.79 (s). GC–MS (EI) *m*/*z*: 379 (22), 378 (4) (M)^+^, 377 (9), 362 (10), 292 (12), 281 (41), 253 (15), 208 (24), 207 (100), 193 (13), 191 (18), 135 (12), 133 (17), 95 (12), 73 (29). HRMS (ESI/Q-TOF) *m*/*z*: calcd for C_22_H_19_ClNOP [M + H]^+^, 380.0966; found, 380.0976.

#### (2-Ethynyl-4-methoxyphenyl]diphenylphosphine Oxide (**12d**)

Compound **5d** (0.0525 g, 0.13 mmol) was reacted with PhMgBr (0.195 mL, 0.195 mmol, 1 M solution in THF) according to general procedure E to afford **12d** (0.035 g, 0.107 mmol, 82% yield) as a yellowish solid. Mp: 156.8–157.8 °C. *R*_*f*_ = 0.15 (10:1 CHCl_3_/MTBE). ^1^H NMR (500 MHz, CDCl_3_): δ 7.72–7.76 (m, 4H), 7.49–7.56 (m, 3H), 7.44–7.48 (m, 4H), 7.13–7.14 (m, 1H), 6.92–6.94 (m, 1H), 3.86 (s, 3H), 2.99 (s, 1H). ^13^C{^1^H} NMR (125 MHz, CDCl_3_): δ 162.0 (d, *J*_P–C_ = 2.7 Hz, C), 135.8 (d, *J*_P–C_ = 10.0 Hz, CH), 132.4 (d, *J*_P–C_ = 107.2 Hz, 2C), 132.2 (d, *J*_P–C_ = 10.0 Hz, 4CH), 131.7 (d, *J*_P–C_ = 2.7 Hz, 2CH), 128.3 (d, *J*_P–C_ = 11.8 Hz, 4CH), 126.9 (d, *J*_P–C_ = 8.2 Hz, C), 125.7 (d, *J*_P–C_ = 107.2 Hz, C), 120.3 (d, *J*_P–C_ = 10.0 Hz, CH), 114.4 (d, *J*_P–C_ = 12.7 Hz, C), 84.7 (s, C), 81.7 (d, *J*_P–C_ = 5.5 Hz, CH), 55.5 (s, CH_3_). ^31^P{^1^H} NMR (202 MHz, CDCl_3_): δ 28.27 (s). GC–MS (EI) *m*/*z*: 333 (22), 332 (100) (M)^+^, 331 (76), 316 (11), 285 (13), 281 (15), 270 (18), 255 (48), 253 (24), 239 (90), 208 (15), 207 (34), 183 (28), 165 (31), 163 (11), 152 (15), 77 (36). HRMS (ESI/Q-TOF) *m*/*z*: calcd for C_21_H_17_O_2_P [M + H]^+^, 333.1039; found, 333.1040.

#### (2-Ethynyl-4-methoxyphenyl)phenyl[*p*-(*N*,*N-*dimethylamino)phenyl]phosphine Oxide (**12k**)

Compound **5d** (0.0525 g, 0.13 mmol) was reacted with *p*-Me_2_N–C_6_H_4_MgBr (0.39 mL, 0.195 mmol, 0.5 M solution in THF) according to general procedure E for 1 h to afford **12k** (0.0311 g, 0.083 mmol, 64% yield) as an orange oil. *R*_*f*_ = 0.57 (30:5:1 CHCl_3_/AcOEt/MeOH). ^1^H NMR (500 MHz, CDCl_3_): δ 7.69–7.73 (m, 2H), 7.52–7.62 (m, 4H), 7.40–7.47 (m, 2H), 7.11–7.12 (m, 1H), 6.90–6.92 (m, 1H), 6.69–6.71 (m, 2H), 3.82 (s, 3H), 3.01 (s, 6H), 2.99 (s, 1H). ^13^C{^1^H} NMR (125 MHz, CDCl_3_): δ 161.7 (d, *J*_P–C_ = 2.7 Hz, C), 152.3 (d, *J*_P–C_ = 2.7 Hz, C), 135.8 (d, *J*_P–C_ = 10.8 Hz, CH), 133.9 (d, *J*_P–C_ = 107.2 Hz, C), 133.6 (d, *J*_P–C_ = 10.9 Hz, 2CH), 132.2 (d, *J*_P–C_ = 10.0 Hz, 2CH), 131.2 (d, *J*_P–C_ = 2.7 Hz, CH), 128.1 (d, *J*_P–C_ = 11.8 Hz, CH), 127.3 (d, *J*_P–C_ = 106.3 Hz, C), 126.7 (d, *J*_P–C_ = 8.2 Hz, C), 120.1 (d, *J*_P–C_ = 10.0 Hz, CH), 117.0 (d, *J*_P–C_ = 118.1 Hz, C), 114.2 (d, *J*_P–C_ = 12.7 Hz, 2CH), 111.2 (d, *J*_P–C_ = 13.6 Hz, CH), 84.3 (s, C), 81.7 (d, *J*_P–C_ = 5.5 Hz, CH), 55.5 (s, CH_3_), 39.9 (s, 2CH_3_). ^31^P{^1^H} NMR (202 MHz, CDCl_3_): δ 28.45 (s). HRMS (ESI/Q-TOF) *m*/*z*: calcd for C_23_H_22_NO_2_P [M + H]^+^, 376.1461; found, 376.1460.

### Reaction of **5a** with *p*-TolMgBr at −78 °C (General Procedure F)

To a Schlenk tube (25 mL) equipped with a magnetic stirrer and an argon inlet was added phosphine oxide **5a** (0.0485 g, 0.13 mmol) in anhydrous THF (2 mL), and the mixture was cooled to −78 °C. Then, *p*-TolMgBr (0.195 mL, 0.195 mmol, 1 M solution in THF) was added. Then, the reaction mixture was stirred at −78 °C for 30 min. Then, the reaction mixture was quenched by the addition of NH_4_Cl solution (5 mL) and extracted with CHCl_3_ (5 × 10 mL). The collected organic phases were dried over Na_2_SO_4_, the solid was filtered off, and the filtrate was evaporated under reduced pressure. The crude reaction mixture was checked using the ^31^P NMR technique. The residue was purified by column chromatography on silica gel using CHCl_3_/EtOAc/MeOH (30:5:1 v/v) to provide **8a** (0.0148 g, 0.047 mmol, 36% yield) and **9**.

#### (2-Ethynylphenyl)(phenyl)((2-(phenyl(*p*-tolyl)phosphoryl)phenyl)ethynyl)phosphine Oxide (**9**):

0.0204 g, 0.038 mmol, 29% yield, white solid (EtOAc). Mp: 233–234 °C (dec). *R*_*f*_ = 0.27 (30:5:1 CHCl_3_/AcOEt/MeOH). ^1^H NMR (500 MHz, CDCl_3_): δ 8.09–8.17 (m, 1H), 7.78–7.82 (m, 1H), 7.60–7.68 (m, 4H), 7.45–7.75 (m, 10H), 7.33–7.43 (m, 4H), 7.15–7.20 (m, 2H), 3.20 (d, *J*_P–H_= 1.89 Hz, 1H), 2.35 (d, *J*_P–H_= 5.04 Hz, 3H). ^13^C{^1^H} NMR (125 MHz, CDCl_3_): δ 142.7 (d, *J*_P–C_ = 2.7 Hz, C), 136.2 (d, *J*_P–C_ = 8.2 Hz, CH), 135.4 (d, *J*_P–C_ = 99.9 Hz, C), 134.4 (dd, *J*_P–C_ = 1.8 Hz, *J*_P–C_ = 11.8 Hz, CH), 133.9 (d, *J*_P–C_ = 10.0 Hz, CH), 133.4 (d, *J*_P–C_ = 10.0 Hz, CH), 132.2 (d, *J*_P–C_ = 3.6 Hz, CH), 132.1 (d, *J*_P–C_ = 10.0 Hz, 2CH), 132.07 (d, *J*_P–C_ = 2.7 Hz, CH), 132.06 (d, *J*_P–C_ = 3.08 Hz, CH), 131.9 (d, *J*_P–C_ = 2.7 Hz, CH), 131.85 (dd, *J*_P–C_ = 4.5 Hz, *J*_P–C_ = 124.4 Hz, C), 131.8 (d, *J*_P–C_ = 2.7 Hz, CH), 131.7 (d, *J*_P–C_ = 1.8 Hz, CH), 131.6 (d, *J*_P–C_ = 11.8 Hz, 2CH), 129.9 (d, *J*_P–C_ = 1.8 Hz, *J*_P–C_ = 11.8 Hz, CH), 129.32 (d, *J*_P–C_ = 13.6 Hz, 2CH), 128.5 (d, *J*_P–C_ = 12.7 Hz, 2CH), 128.1 (d, *J*_P–C_ = 2.7 Hz, CH), 128.1 (d, *J*_P–C_ = 2.7 Hz, CH), 127.4 (d, *J*_P–C_ = 8.2 Hz, C), 123.9 (dm, C), 102.5 (dd, *J*_P–C_ = 4.5 Hz, *J*_P–C_ = 29.1 Hz, C), 89.3 (d, *J*_P–C_ = 172.6 Hz, C), 85.6 (s, C), 80.7 (dd, *J*_P–C_ = 2.7 Hz, *J*_P–C_ = 7.3 Hz, CH), 21.6 (s, CH_3_). ^31^P{^1^H} NMR (202 MHz, CDCl_3_): δ 29.41 (d, *J*_P–P_ = 12.4 Hz), 7.44 (d, *J*_P–P_ = 12.4 Hz). HRMS (ESI/Q-TOF) *m*/*z*: calcd for C_35_H_26_O_2_P_2_ [M + H]^+^, 541.1481; found, 541.1479.

### Reaction of **5a** with *p*-TolMgBr in a Higher Scale (General Procedure G)

To a Schlenk tube (25 mL) equipped with a magnetic stirrer and an argon inlet phosphine was added oxide **5a** (0.24 g, 0.634 mmol) in anhydrous THF (5 mL), and the mixture was cooled to 0 °C. Then, *p*-TolMgBr (0.952 mL, 0.952 mmol, 1 M in THF) was added dropwise. Then, the reaction mixture was stirred at 0 °C for 1 h. Then, the reaction mixture was quenched by the addition of NH_4_Cl solution (5 mL) and extracted with CHCl_3_ (5 × 5 mL). The collected organic phases were dried over Na_2_SO_4_, the solid was filtered off, and the filtrate was evaporated under reduced pressure. The crude reaction mixture was checked using the ^31^P{^1^H} NMR technique. The residue was purified by column chromatography on silica gel using CHCl_3_/EtOAc/MeOH (30:5:1 v/v) as an eluent, yielding (2-ethynylphenyl)phenyl(*p*-tolyl)phosphine oxide (**8a**) in 91% yield (0.182 g, 0.577 mmol).

### Reaction of **5a** with PhLi or EtMgBr (General Procedure H)

To a Schlenk tube (25 mL) equipped with a magnetic stirrer and an argon inlet was added phosphine oxide **5a** (0.0405 g, 0.108 mmol) in anhydrous THF (2 mL), and the mixture was cooled to 0 °C. Then, PhLi or EtMgBr (0.164 mmol) was added. Then, the reaction mixture was stirred at 0 °C for 30 min. Then, the reaction mixture was quenched by the addition of NH_4_Cl solution (5 mL) and extracted with CHCl_3_ (5 × 10 mL). The collected organic phases were dried over Na_2_SO_4_, the solid was filtered off, and the filtrate was evaporated under reduced pressure. The crude reaction mixture was checked using the ^31^P{^1^H} NMR technique. The residue was purified by column chromatography on silica gel using CHCl_3_/MTBE (30:1 v/v).

#### [2-(Ethynyl)phenyl]diphenylphosphine Oxide (**8d**)

Compound **5a** (0.0405 g, 0.108 mmol) was reacted with PhLi(0.086 mL, 0.164 mmol, 1.9 M solution in *n*-Bu_2_O) according to general procedure H to afford **8d** (0.007 g, 0.022 mmol, 21% yield).

#### Ethyl(2-ethynylphenyl)phenylphosphine (**13**)

Compound **5a** (0.0405 g, 0.108 mmol) was reacted with EtMgBr (0.162 mL, 0.162 mmol, 1 M solution in THF) according to general procedure H to afford **13** (0.016 g, 0.063 mmol, 58% yield) as a yellow oil. *R*_*f*_ = 0.35 (30:5:1 CHCl_3_/AcOEt/MeOH). ^1^H NMR (500 MHz, CDCl_3_): δ 8.18–8.24 (m, 1H), 7.71–7.76 (m, 2H), 7.56–7.91 (m, 1H), 7.46–7.55 (m, 3H), 7.40–7.44 (m, 2H), 3.21 (s, 1H), 2.71–2.80 (m, 1H), 2.51–2.61 (m, 1H), 1.20 (dt, *J*_H–H_ = 7.72 Hz, *J*_H–P_= 17.9 Hz, 3H). ^31^P{^1^H} NMR (202 MHz, CDCl_3_): δ 34.50 (s). ^13^C{^1^H} NMR (125 MHz, CDCl_3_): δ 134.7 (d, *J*_C–P_ = 8.62 Hz, CH), 133.9 (d, *J*_C–P_ = 92.6 Hz, C), 133.8 (d, *J*_C–P_ = 6.8 Hz, CH), 133.1 (d *J*_C–P_ = 99.9 Hz, C), 131.6 (d, *J*_C–P_ = 2.5 Hz, CH), 131.5 (d, *J*_C–P_ = 2.5 Hz, CH), 130.9 (d, *J*_C–P_ = 9.99 Hz, 2CH), 128.9 (d, *J*_C–P_ = 10.0 Hz, CH), 128.3 (d, *J*_C–P_ = 12.7 Hz, 2CH), 123.2 (d, *J*_C–P_ = 8.2 Hz, C), 84.2 (s, C), 82.3 (d, *J*_C–P_ = 4.5 Hz, CH), 21.0 (d, *J*_C–P_ = 72.7 Hz, CH_2_), 5.59 (d, *J*_C–P_ = 5.5 Hz, CH_3_). GC–MS (EI) *m*/*z*: (%) 255 (9), 254 (49) (M)^+^, 253 (9), 226 (35), 225 (100), 179 (17), 178 (30), 165 (17), 152 (12), 149 (11), 77 (25). HRMS (ESI/Q-TOF) *m*/*z*: calcd for C_32_H_30_O_2_P_2_Na [2M + Na]^+^, 531.1619; found, 531.1628.

## Supporting Information Available

^1^H NMR, ^13^C{^1^H} NMR, ^31^P{^1^H} NMR, and ^19^F NMR spectra for all products, crystallographic data (PDF)

## Accession Codes

CCDC 2094907 contains the supplementary crystallographic data for this paper. These data can be obtained free of charge via www.ccdc.cam.ac.uk/data_request/cif, or by emailing data_request@ccdc.cam.ac.uk, or by contacting The Cambridge Crystallographic Data Centre, 12 Union Road, Cambridge CB2 1EZ, UK; fax: +44 1223 336033.
