# Supplementary material for: Ring Opening of Triflates Derived from Benzophospholan-3-one Oxides by Aryl Grignard Reagents as a Route to 2-Ethynylphenyl(diaryl)phosphine Oxides
Source: J Org Chem. 2021 Oct 26;86(21):14928–41. doi: 10.1021/acs.joc.1c01629 (PMC8576819; doi:10.1021/acs.joc.1c01629)
Supplement: Supplementary file 1 — jo1c01629_si_001.pdf [file jo1c01629_si_001.pdf]

Supporting information

Ring opening of triflates derived from benzophospholan-3-one oxides by aryl Grignard reagents as a route to 2-ethynylphenyl(diaryl)phosphine oxides

Łukasz Ponikiewski, Sylwia Sowa*

Table of contents:

Copies of ^{1}H NMR ^{13}C NMR and ^{31}P NMR spectra of compounds S3-82
(2-Methoxycarbonylphenyl)(methyl)phenylphosphine oxide (**2a**) S3
(2-Methoxycarbonyl-6-methylphenyl)(phenyl)phosphine oxide (**2b**) S4
(2-Methoxycarbonyl-5-chlorophenyl)(phenyl)phosphine oxide (**2c**) S6
(2-Methoxycarbonyl-4-methoxyphenyl)(phenyl)phosphine oxide (**2d**) S8
Benzophospholan-3-one oxide (**3a**) S10
7-Methylbenzophospholan-3-one oxide (**3b**) S12
6-Chlorobenzophospholan-3-one oxide (**3c**) S14
4-Methoxybenzophospholan-3-one oxide (**3d**) S16
Benzophospholan-3-one sulfide (**4a**) S18
1-Oxido-1-phenyl-1H-phosphindol-3-yl trifluoromethanesulfonate (**5a**) S20
1-Oxido-1-phenyl-1H-7-methylphosphindol-3-yl trifluoromethanesulfonate (**5b**) S23
1-Oxido-1-phenyl-1H-6-chlorophosphindol-3-yl trifluoromethanesulfonate (**5c**) S25
1-Oxido-1-phenyl-1H-5-methoxyphosphindol-3-yl trifluoromethanesulfonate (**5d**) S27
1-Sulfido-1-phenyl-1H-phosphindol-3-yl trifluoromethanesulfonate (**6a**) S30
(2-Ethynylphenyl)phenyl(*p*-tolyl)phosphine oxide (**8a**) S32
(2-Ethynylphenyl)phenyl(*m*-tolyl)phosphine oxide (**8b**) S34
(2-Ethynylphenyl)phenyl(*o*-tolyl)phosphine oxide (**8c**) S36
[2-(Ethynyl)phenyl]diphenylphosphine oxide (**8d**) S38
p-Anisyl(2-ethynylphenyl)phenylphosphine oxide (**8e**) S40
m-Anisyl(2-ethynylphenyl)phenylphosphine oxide (**8f**) S42
o-Anisyl(2-ethynylphenyl)phenylphosphine oxide (**8g**) S44
2-Ethynylphenyl(9-phenanthryl)phenylphosphine oxide (**8h**) S46
p-Chlorophenyl(2-ethynylphenyl)phenylphosphine oxide (**8i**) S48
2-Ethynylphenyl(*p*-fluorophenyl)phenylphosphine oxide (**8j**) S50
(2-Ethynylphenyl)phenyl[*p*-(*N*,*N*-dimethylamino)phenyl]phosphine oxide (**8k**) S52
(2-Ethynyl-6-methylphenyl)phenyl(*p*-tolyl)phosphine oxide (**10a**) S54
(2-Ethynyl-6-methylphenyl]diphenylphosphine oxide (**10d**) S56
(*p*-Anisyl)(2-ethynyl-6-methylphenyl]phenylphosphine oxide (**10e**) S58
(2-Ethynyl-6-methylphenyl)(4-chlorophenyl)phenylphosphine oxide (**10i**) S60
(2-Ethynyl-6-methylphenyl)[*p*-(*N*,*N*-dimethylamino)phenyl]phenylphosphine oxide (**10k**) S62
(2-Ethynyl-5-chlorophenyl)phenyl(*p*-tolyl)phosphine oxide (**11a**) S64
(2-Ethynyl-5-chlorophenyl]diphenylphosphine oxide (**11d**) S66
(*p*-Anisyl)(2-ethynyl-5-chlorophenyl]phenylphosphine oxide (**11e**) S68
(4-Chlorophenyl)(2-ethynyl-5-chlorophenyl]phenylphosphine oxide (**11i**) S70
(2-Ethynyl-5-chlorophenyl)[*p*-(*N*,*N*-dimethylamino)phenyl]phenyl phosphine oxide (**11k**) S72
(2-Ethynyl-4-methoxyphenyl]diphenylphosphine oxide (**12d**) S74
(2-Ethynyl-4-methoxyphenyl)phenyl[*p*-(*N*,*N*-dimethylamino)phenyl]phosphine oxide (**12k**) S76
(2-ethynylphenyl)(phenyl)((2-(phenyl(*p*-tolyl)phosphoryl)phenyl)ethynyl)phosphine oxide (**9**) S78
Ethyl(2-ethynylphenyl)phenylphosphine oxide (**13**) S80
Table S1. Crystallographic data for **9**. S83
References S83

Copies of ^{1}H NMR ^{13}C NMR and ^{31}P NMR spectra of compounds

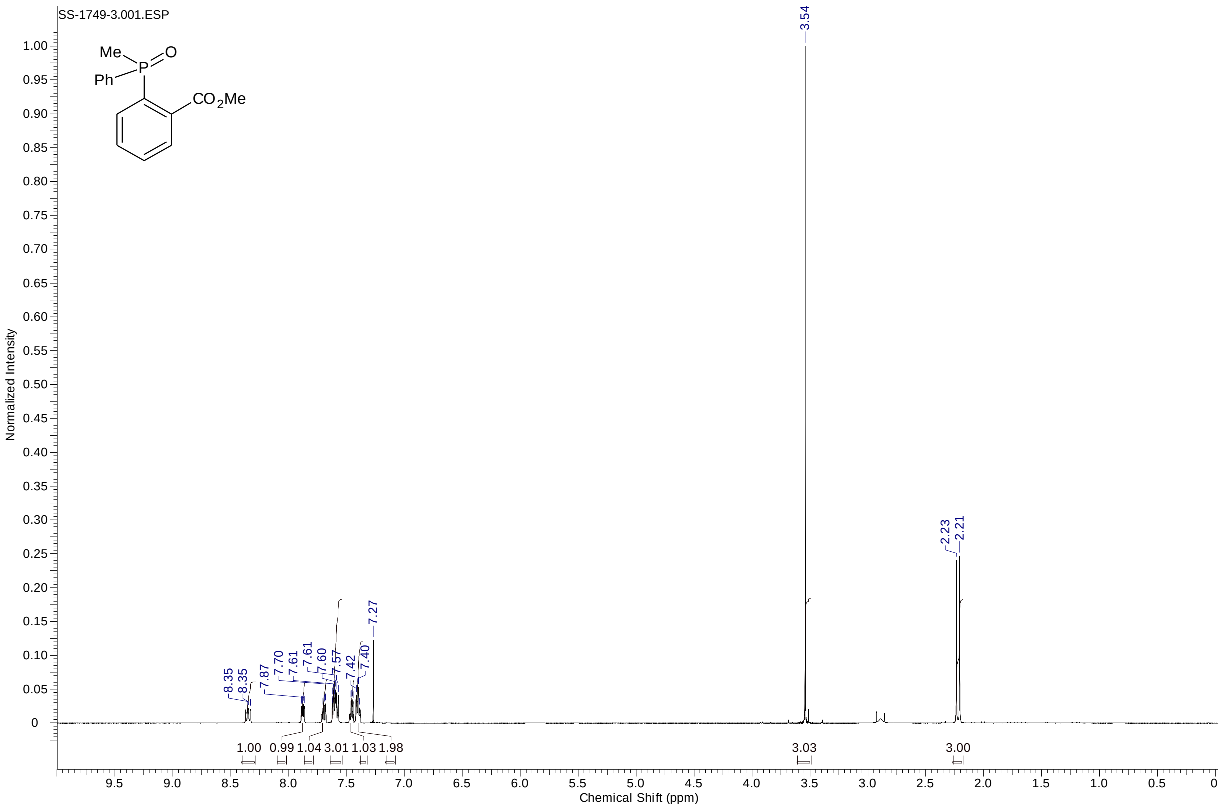

^{1}H NMR spectrum of (2-methoxycarbonylphenyl)(methyl)phenylphosphine oxide (**2a**) (500 MHz, $CDCl_3$)[1]

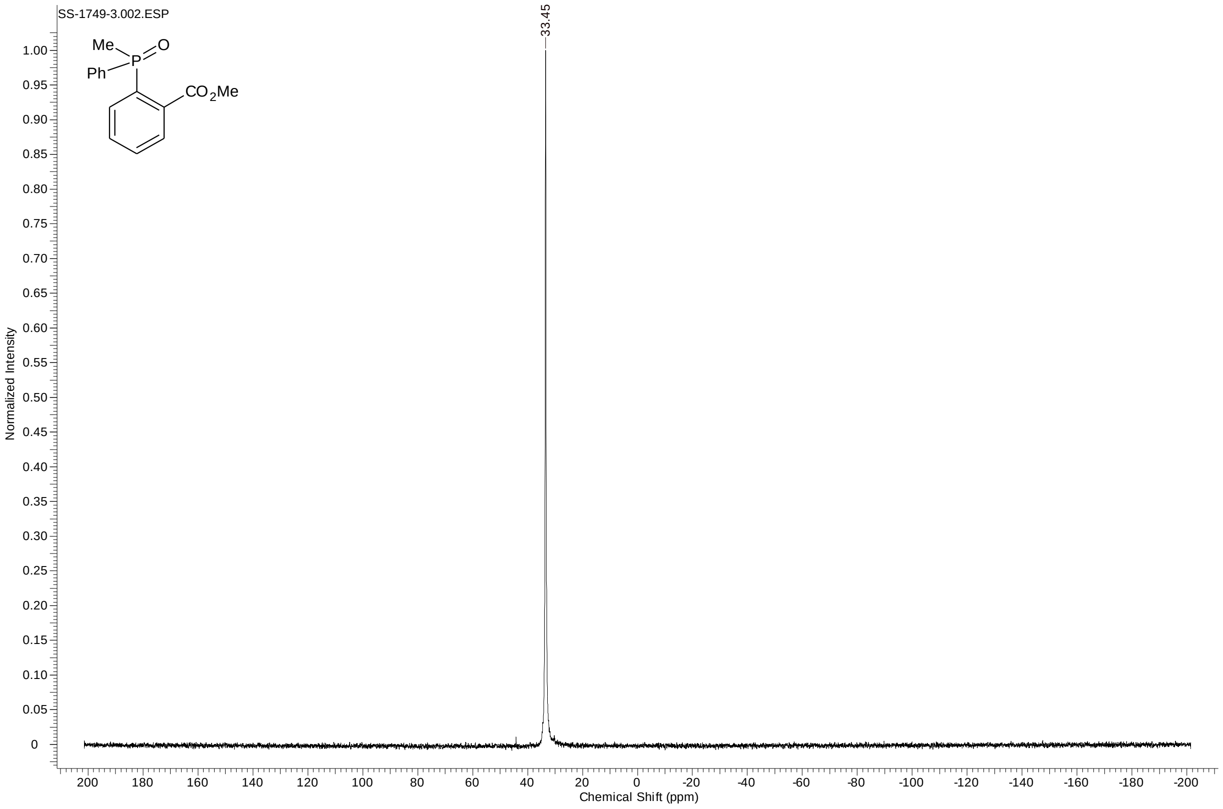

^{31}P{^{1}H} NMR spectrum of (2-methoxycarbonylphenyl)(methyl)phenylphosphine oxide (**2a**) (202 MHz, $CDCl_3$)[1]

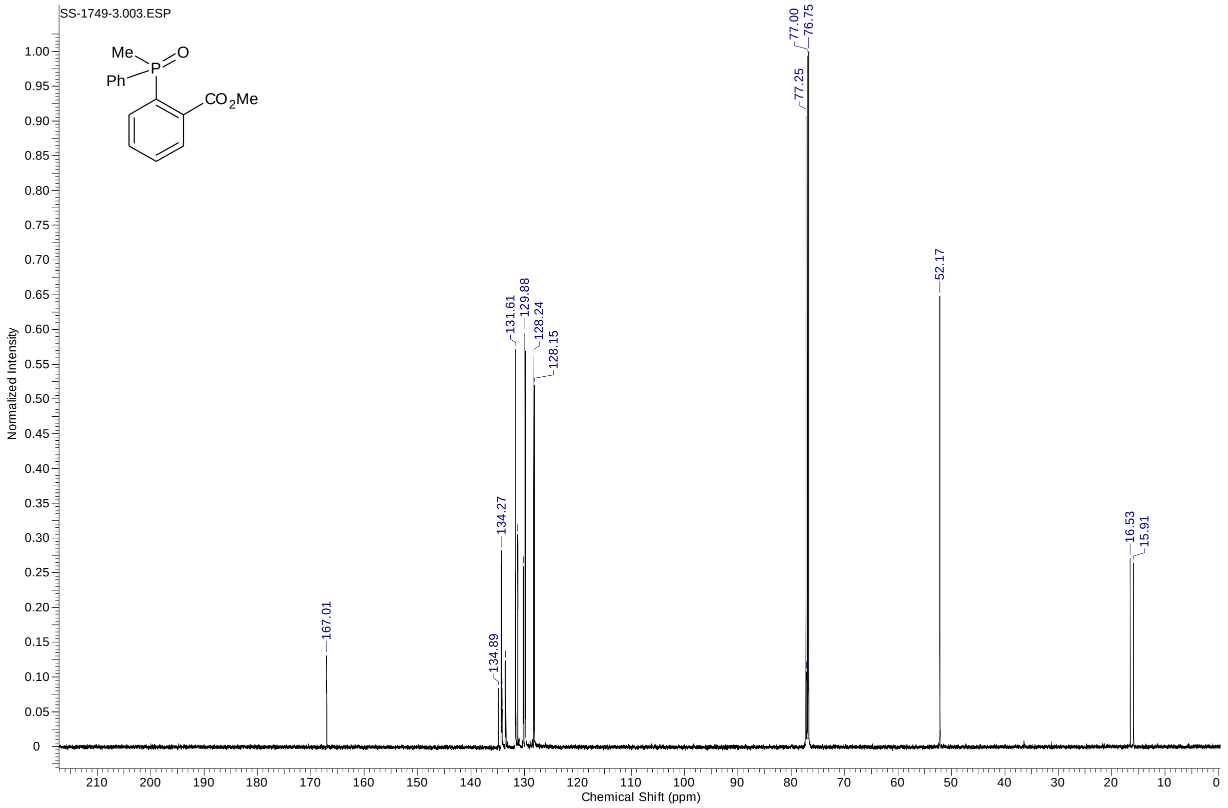

$^{13}C\{^{1}H\}$ NMR spectrum of (2-methoxycarbonylphenyl)(methyl)phenylphosphine oxide (**2a**) (125 MHz, $CDCl_3$)[1]

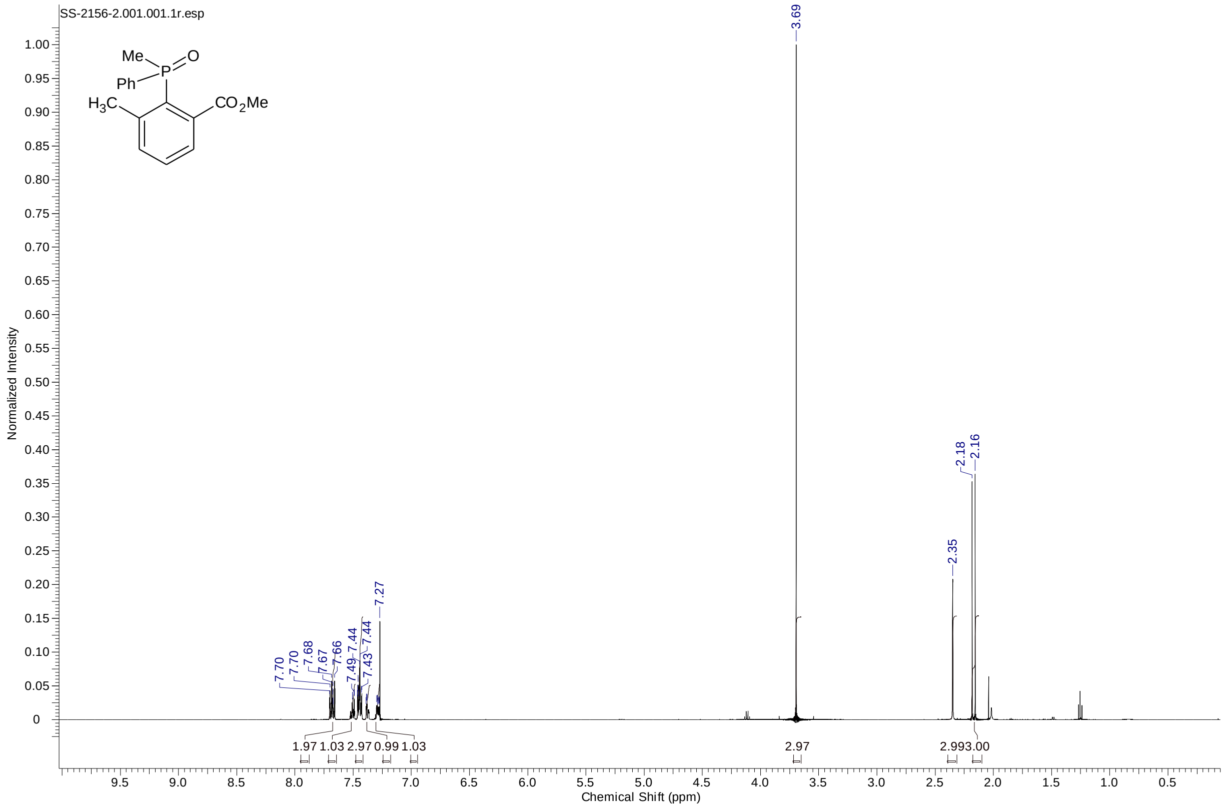

^{1}H NMR spectrum of (2-methoxycarbonyl-6-methylphenyl)(phenyl)phosphine oxide (**2b**) (500 MHz, $CDCl_3$)

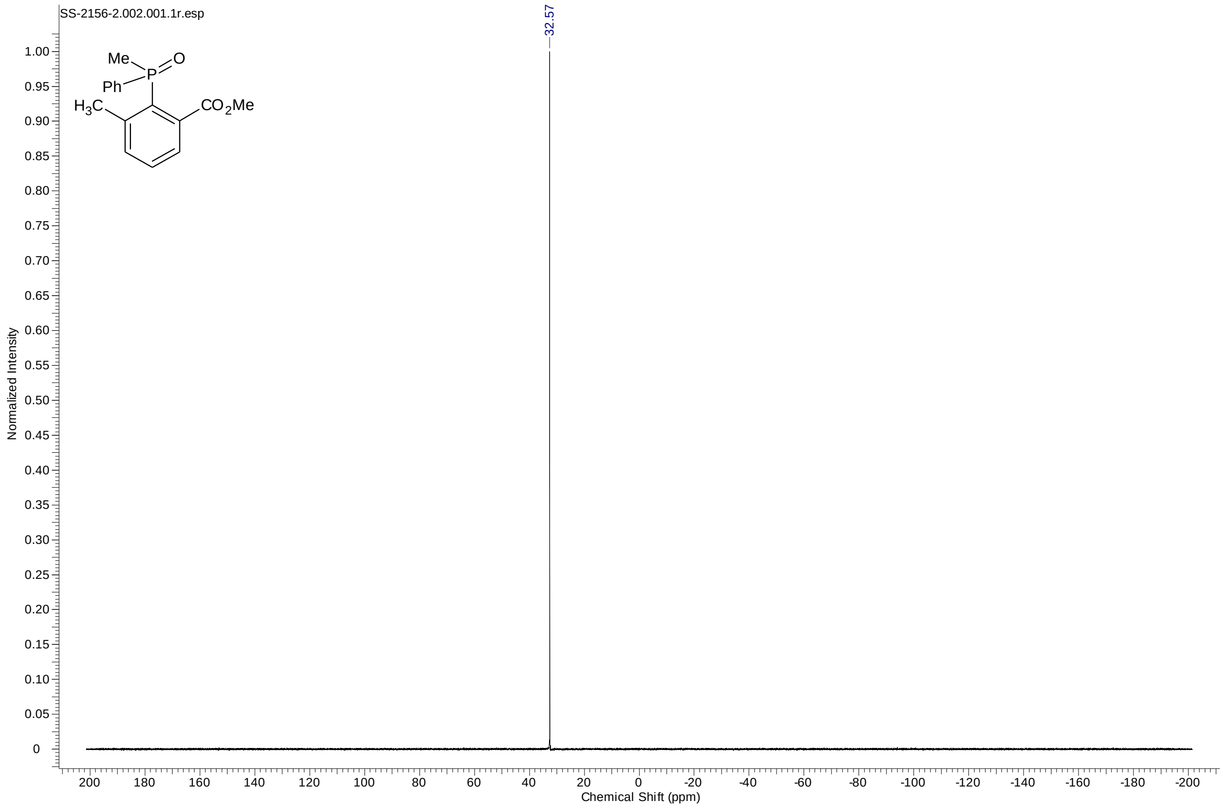

^{31}P NMR spectrum of (2-methoxycarbonyl-6-methylphenyl)(phenyl)phosphine oxide (**2b**) (202 MHz, $CDCl_3$)

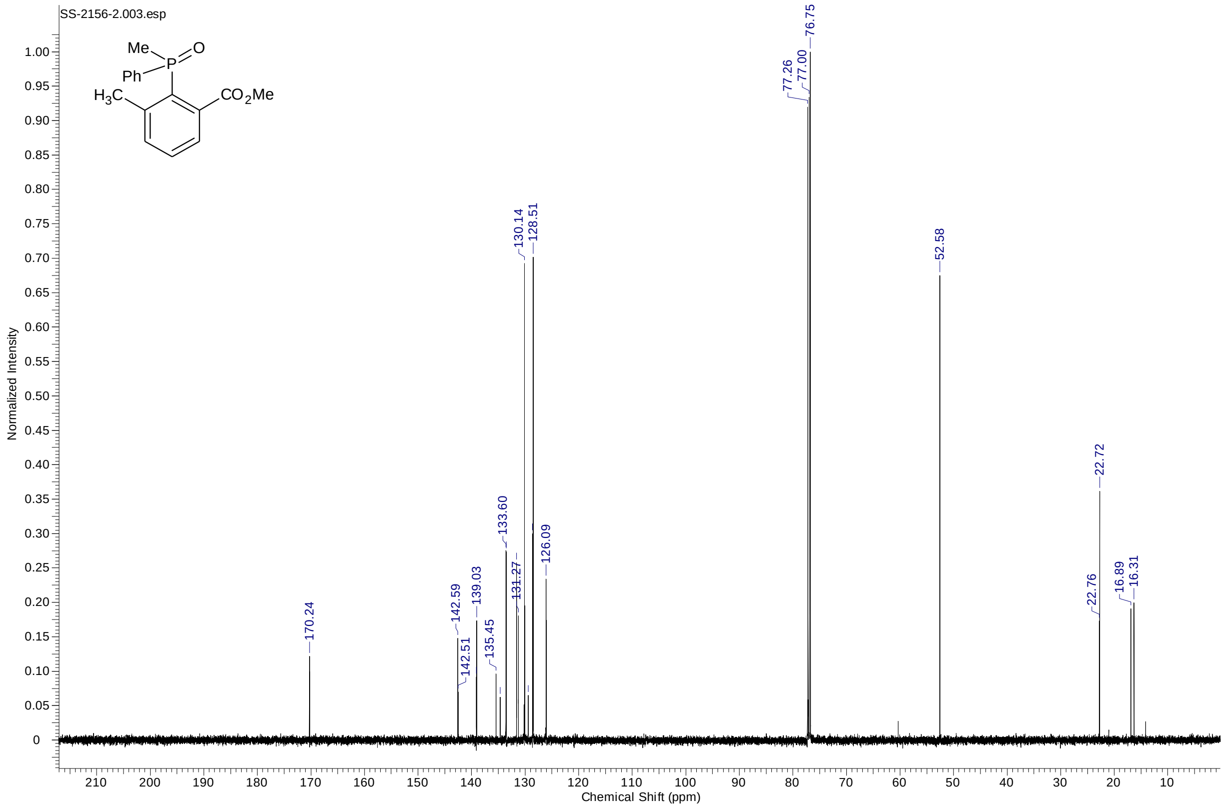

$^{13}C\{^1H\}$ NMR spectrum of (2-methoxycarbonyl-6-methylphenyl)(phenyl)phosphine oxide (**2b**) (125 MHz, $CDCl_3$)

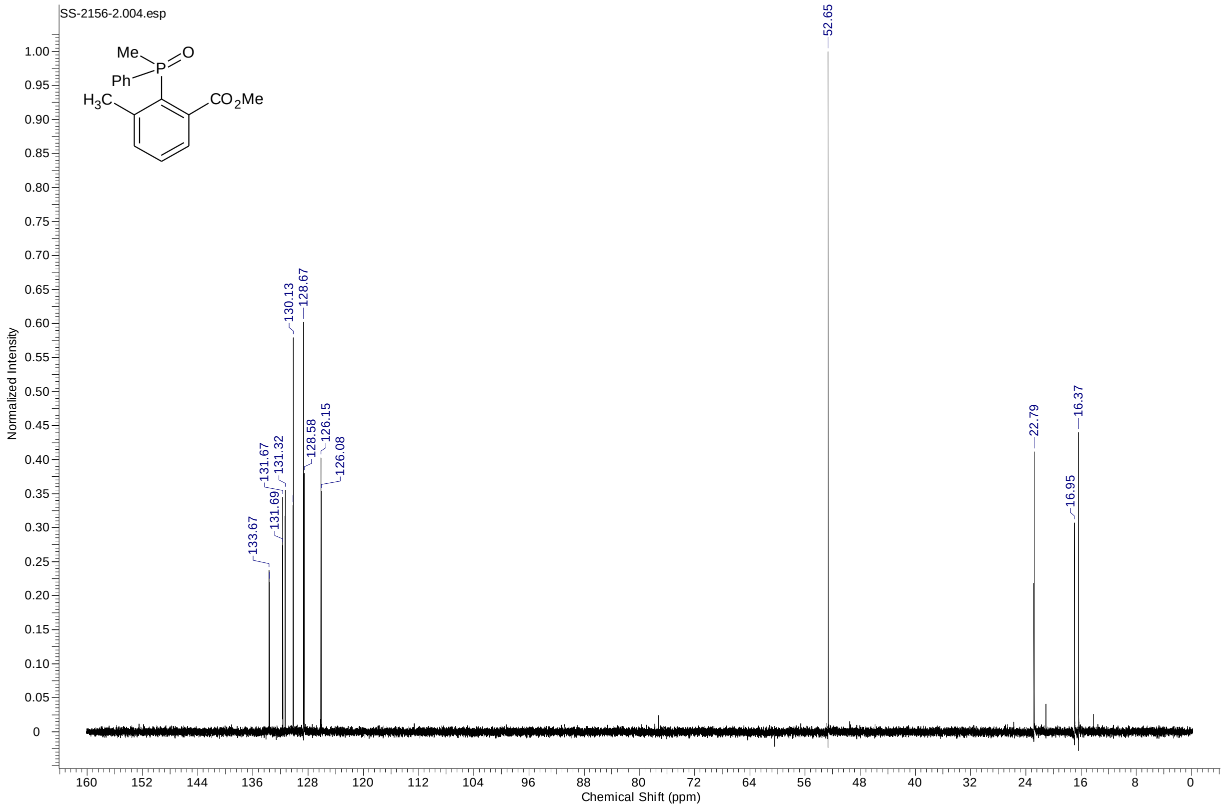

DEPT 135 NMR spectrum of (2-methoxycarbonyl-6-methylphenyl)(phenyl)phosphine oxide (**2b**) (125 MHz, $CDCl_3$)

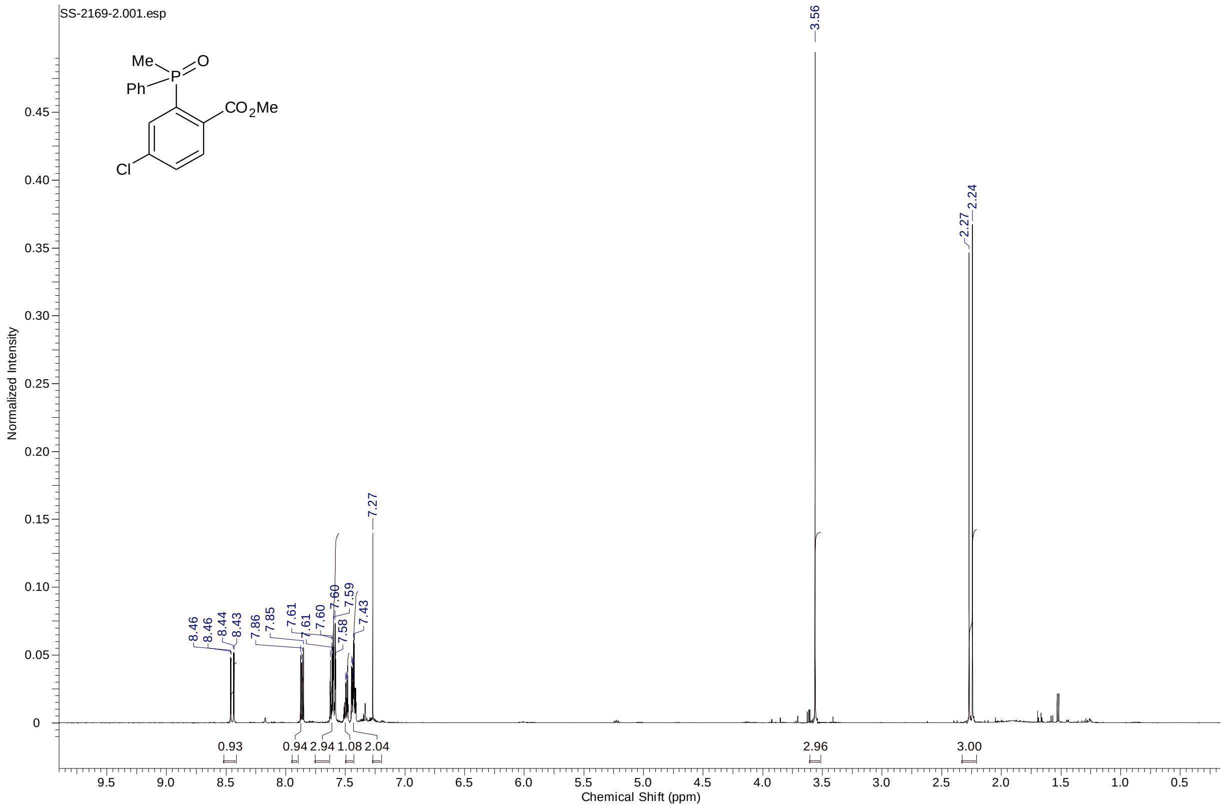

^1H NMR spectrum of (2-methoxycarbonyl-5-chlorophenyl)(phenyl)phosphine oxide (**2c**) (500 MHz, $CDCl_3$)

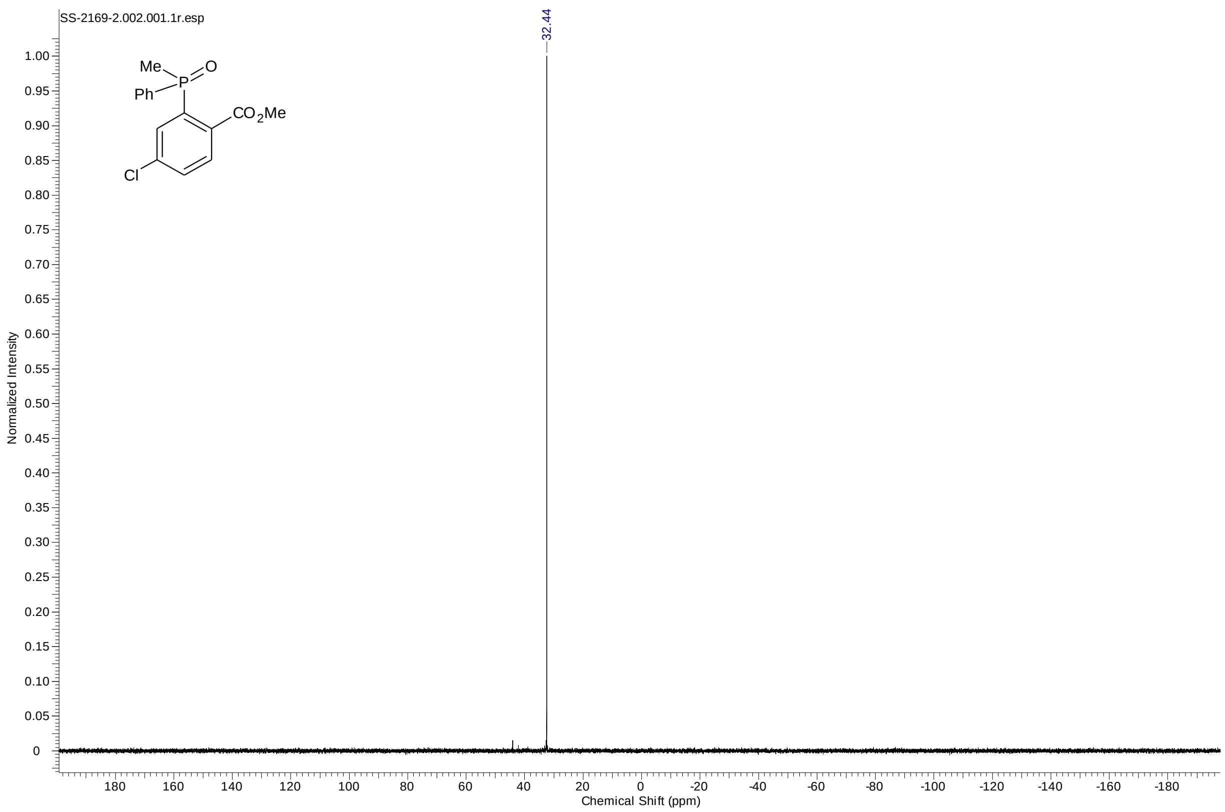

^{31}P NMR spectrum of (2-methoxycarbonyl-5-chlorophenyl)(phenyl)phosphine oxide (**2c**) (202 MHz, $CDCl_3$)

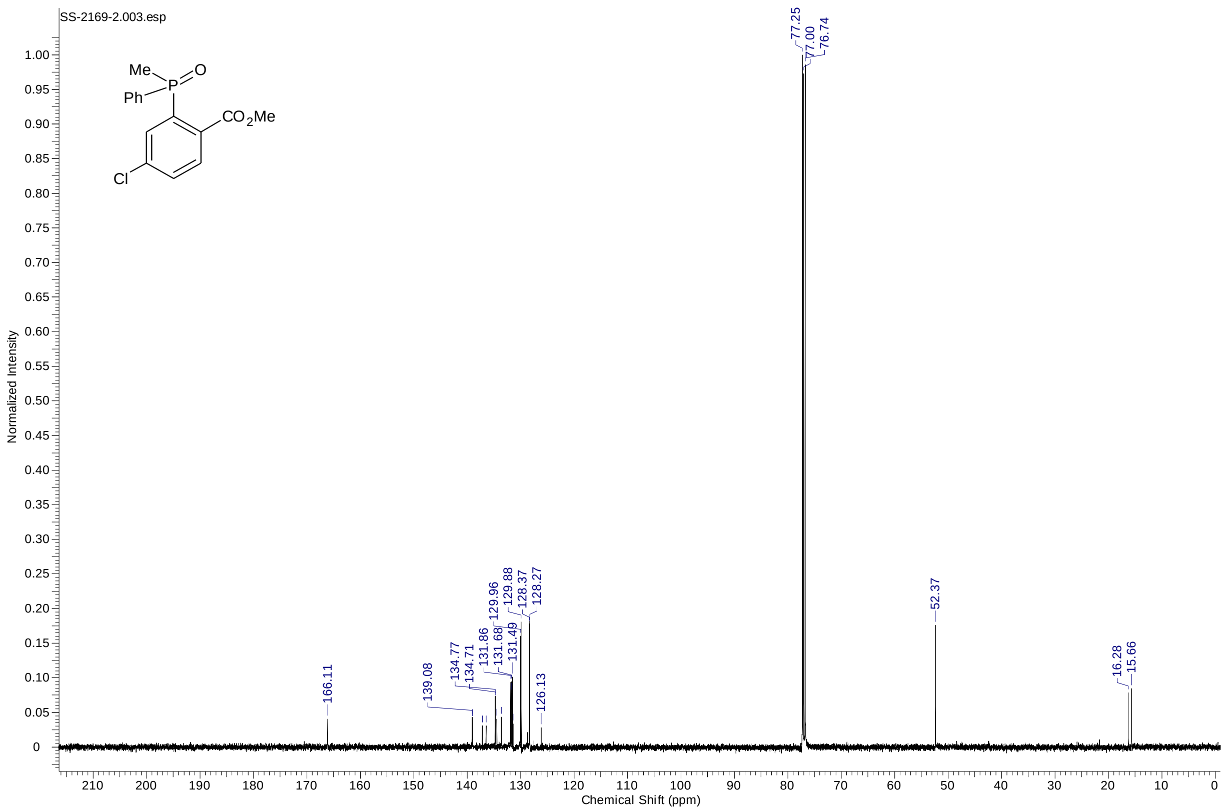

^{13}C{^{1}H} NMR spectrum of (2-methoxycarbonyl-5-chlorophenyl)(phenyl)phosphine oxide (**2c**) (125 MHz, $CDCl_3$)

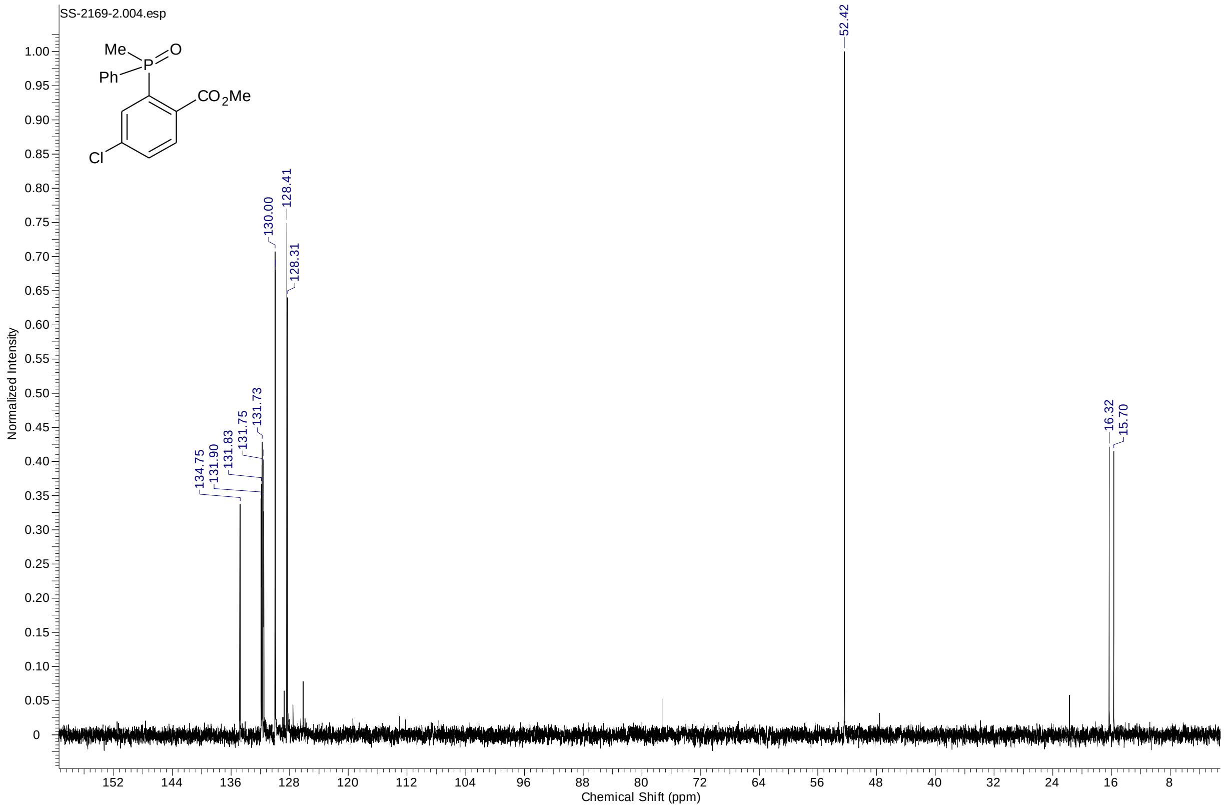

DEPT 135 NMR spectrum of (2-methoxycarbonyl-5-chlorophenyl)(phenyl)phosphine oxide (**2c**) (125 MHz, $CDCl_3$)

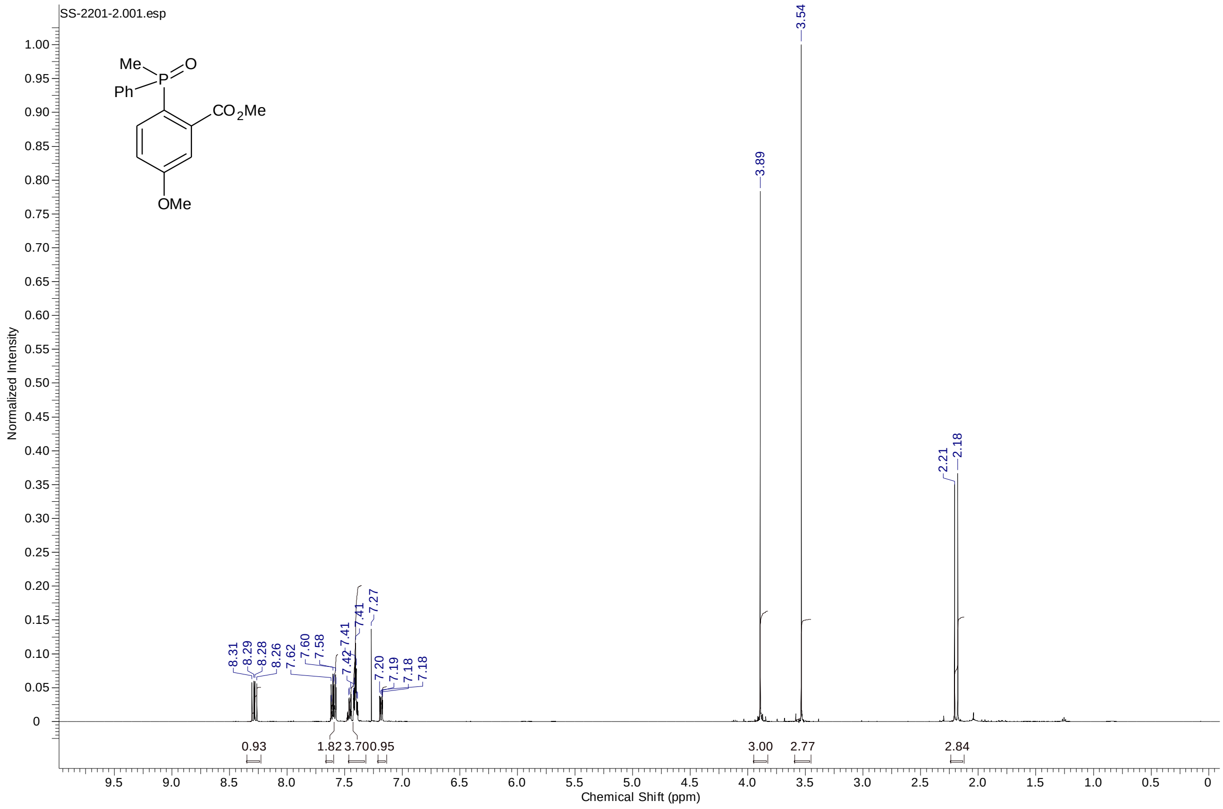

^{1}H NMR spectrum of (2-methoxycarbonyl-4-methoxyphenyl)(phenyl)phosphine oxide (**2d**) (500 MHz, $CDCl_3$)

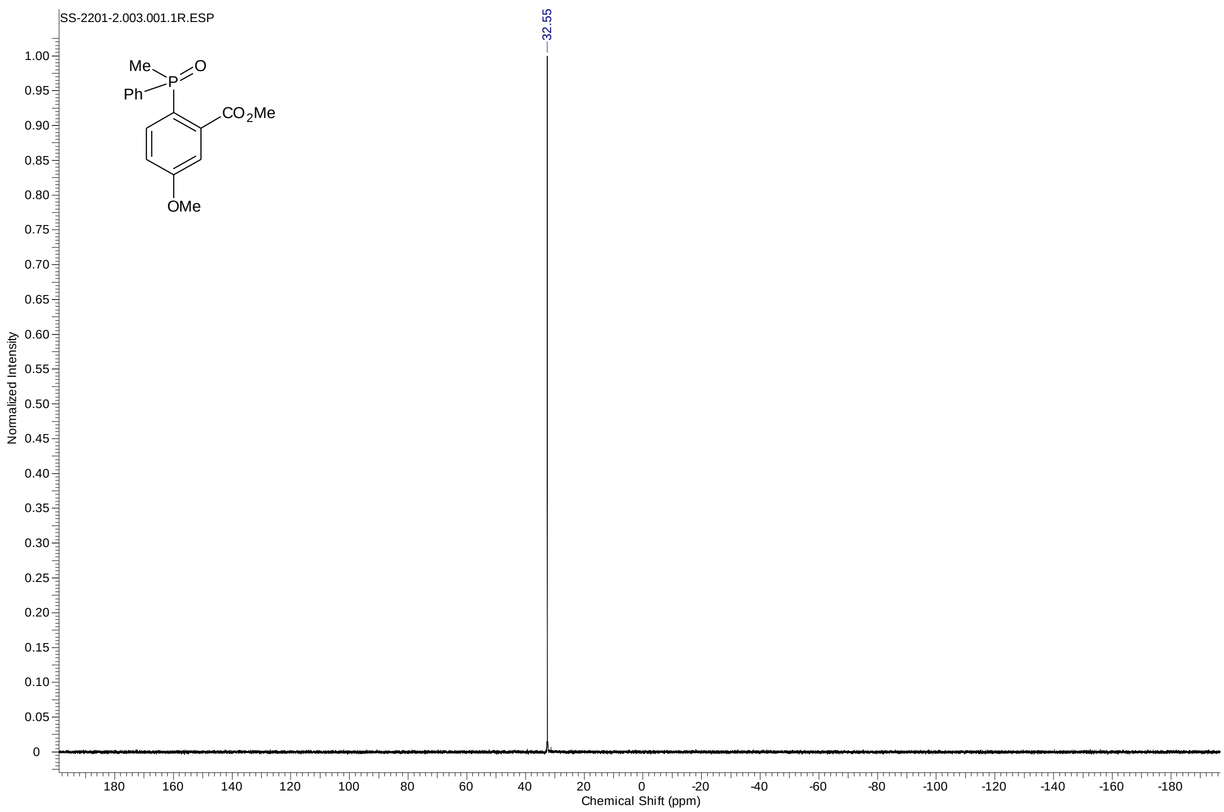

^{31}P NMR spectrum of (2-methoxycarbonyl-4-methoxyphenyl)(phenyl)phosphine oxide (**2d**) (202 MHz, $CDCl_3$)

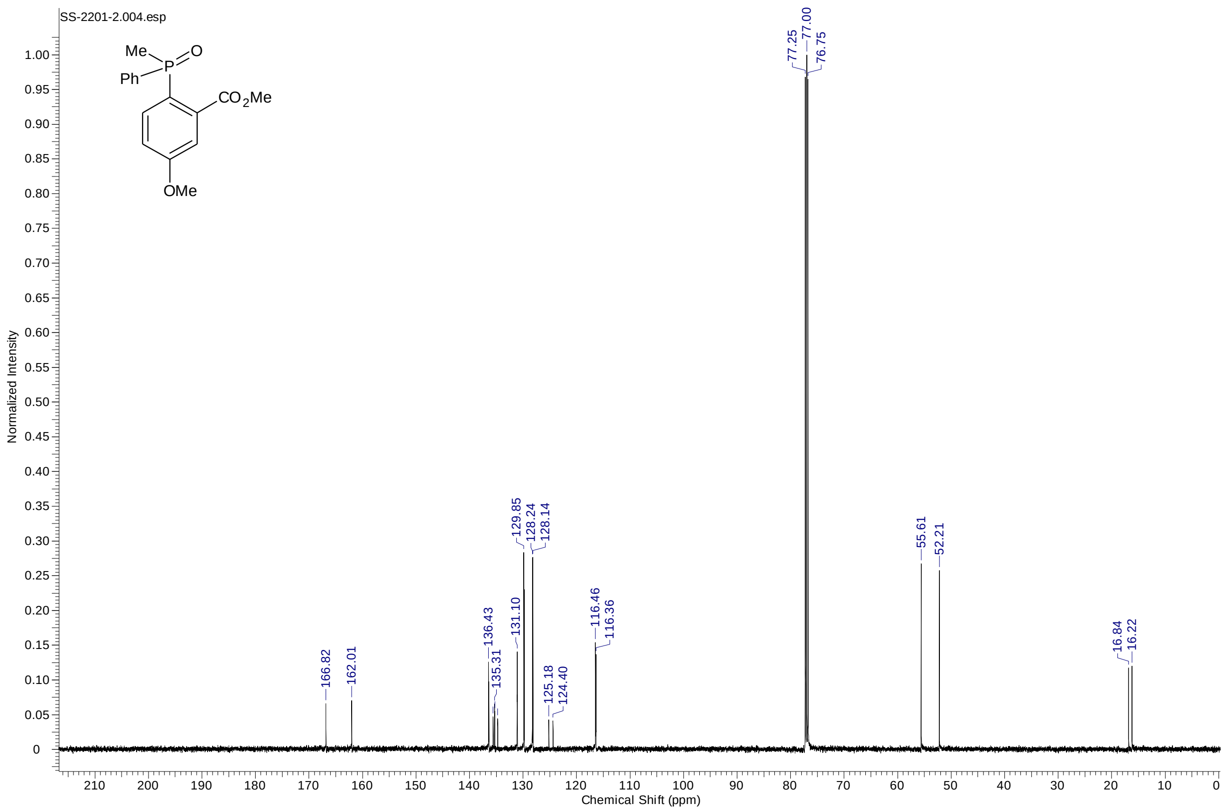

$^{13}C\{^{1}H\}$ NMR spectrum of (2-methoxycarbonyl-4-methoxyphenyl)(phenyl)phosphine oxide (**2d**) (125 MHz, $CDCl_3$)

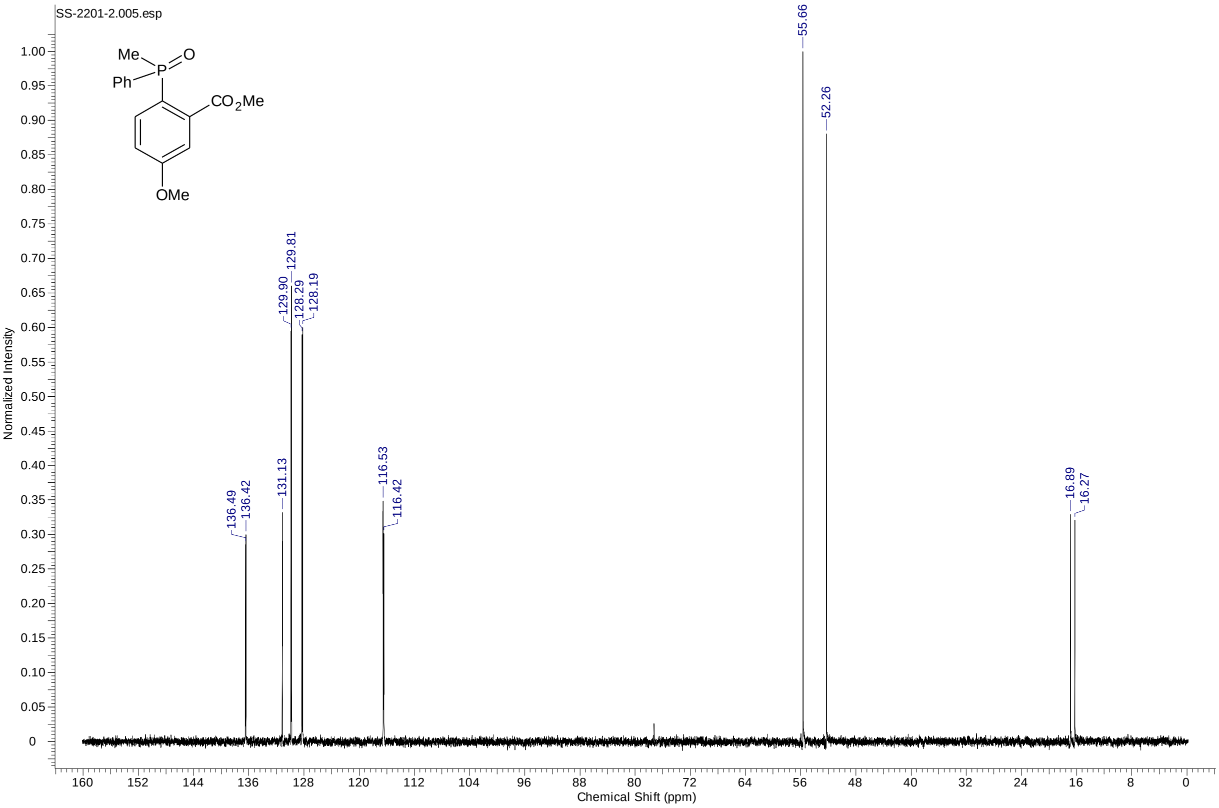

DEPT 135 NMR spectrum of (2-methoxycarbonyl-4-methoxyphenyl)(phenyl)phosphine oxide (**2d**) (125 MHz, $CDCl_3$)

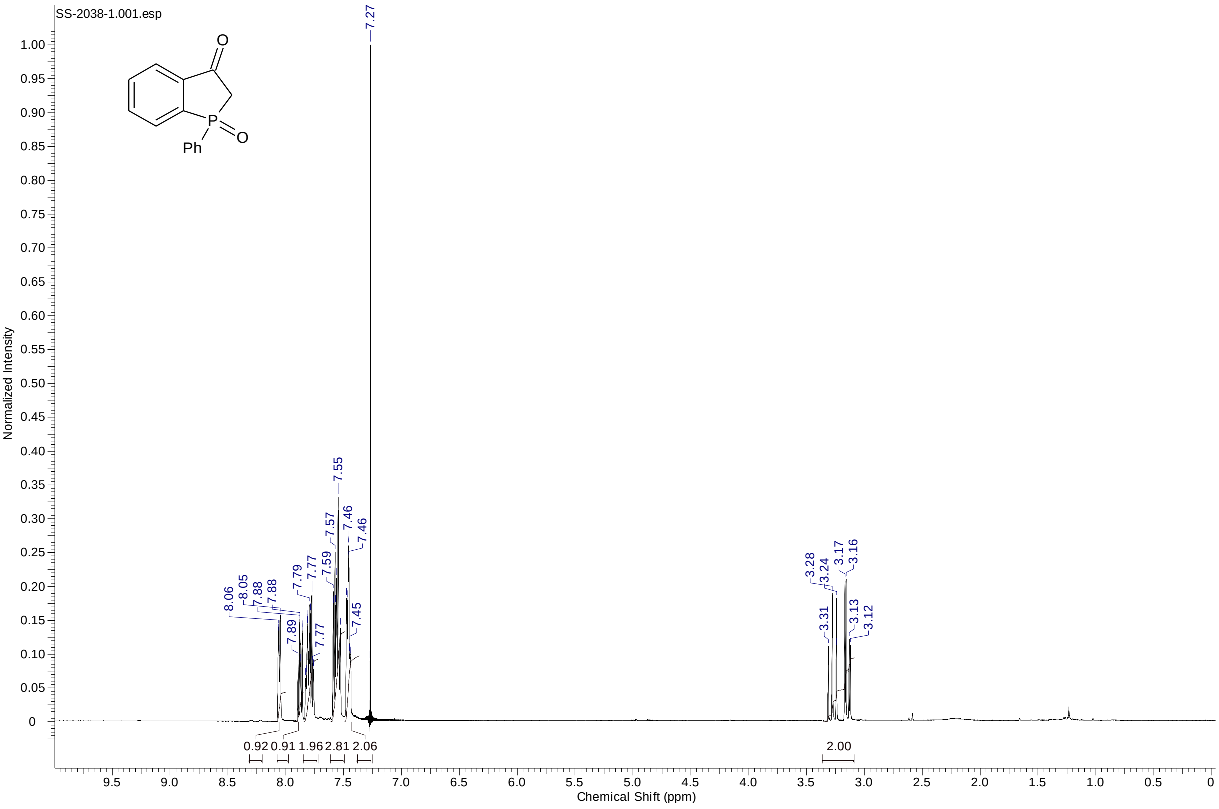

^1H NMR spectrum of benzophospholan-3-one oxide (**3a**) (500 MHz, $CDCl_3$)[2]

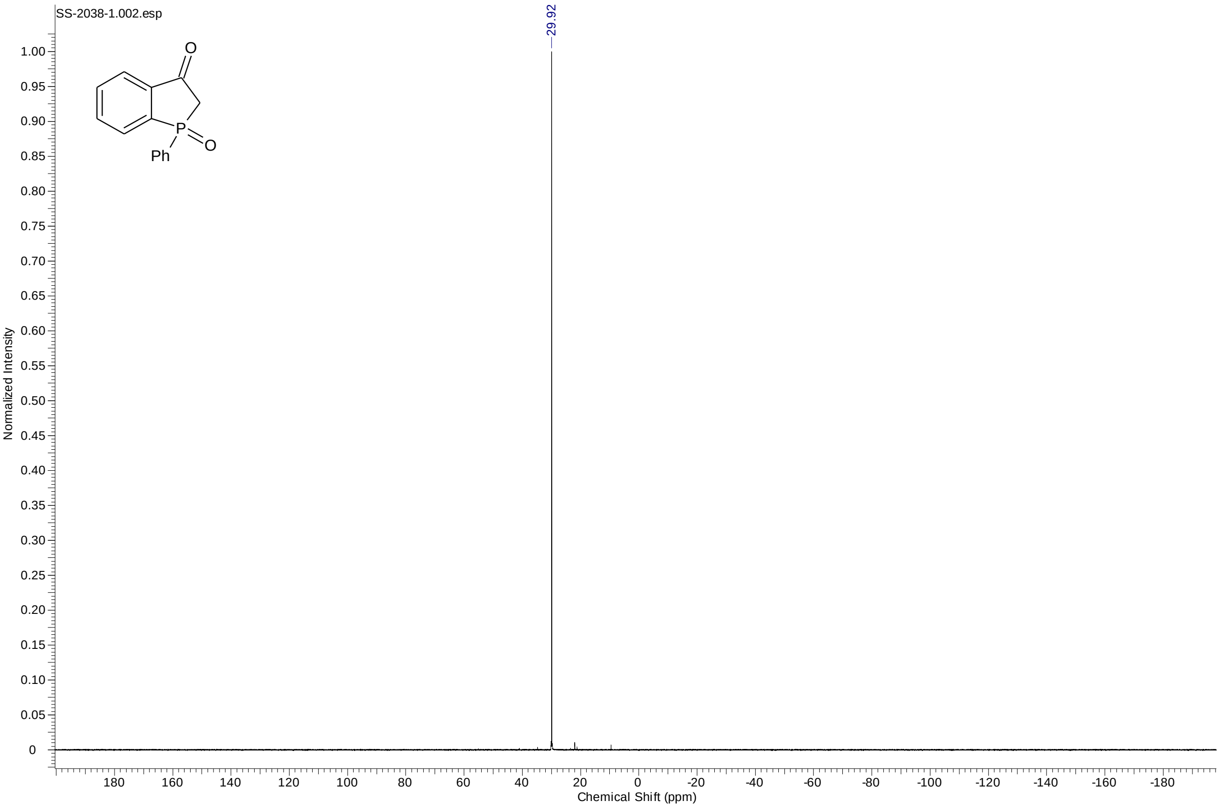

^{31}P NMR spectrum of benzophospholan-3-one oxide (**3a**) (202 MHz, $CDCl_3$)[9]

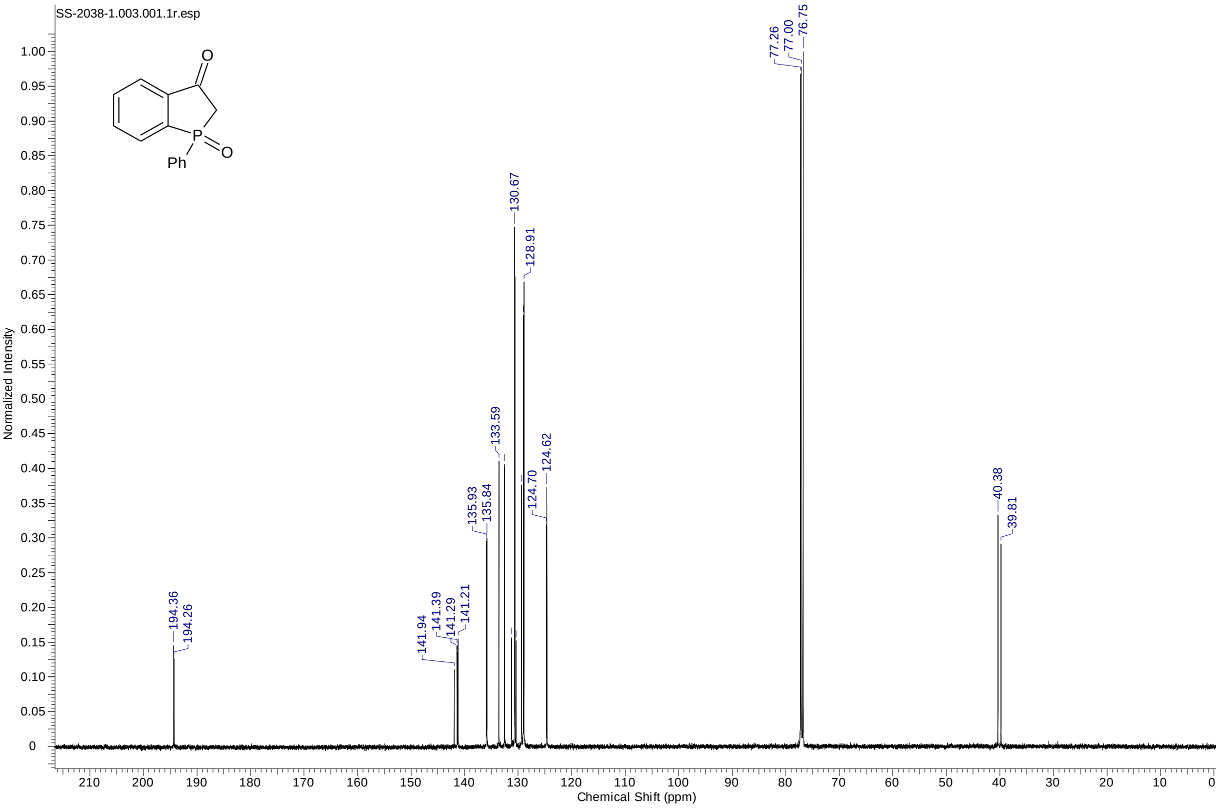

$^{13}C\{^1H\}$ NMR spectrum of benzophospholan-3-one oxide (**3a**) (202 MHz, $CDCl_3$)[9]

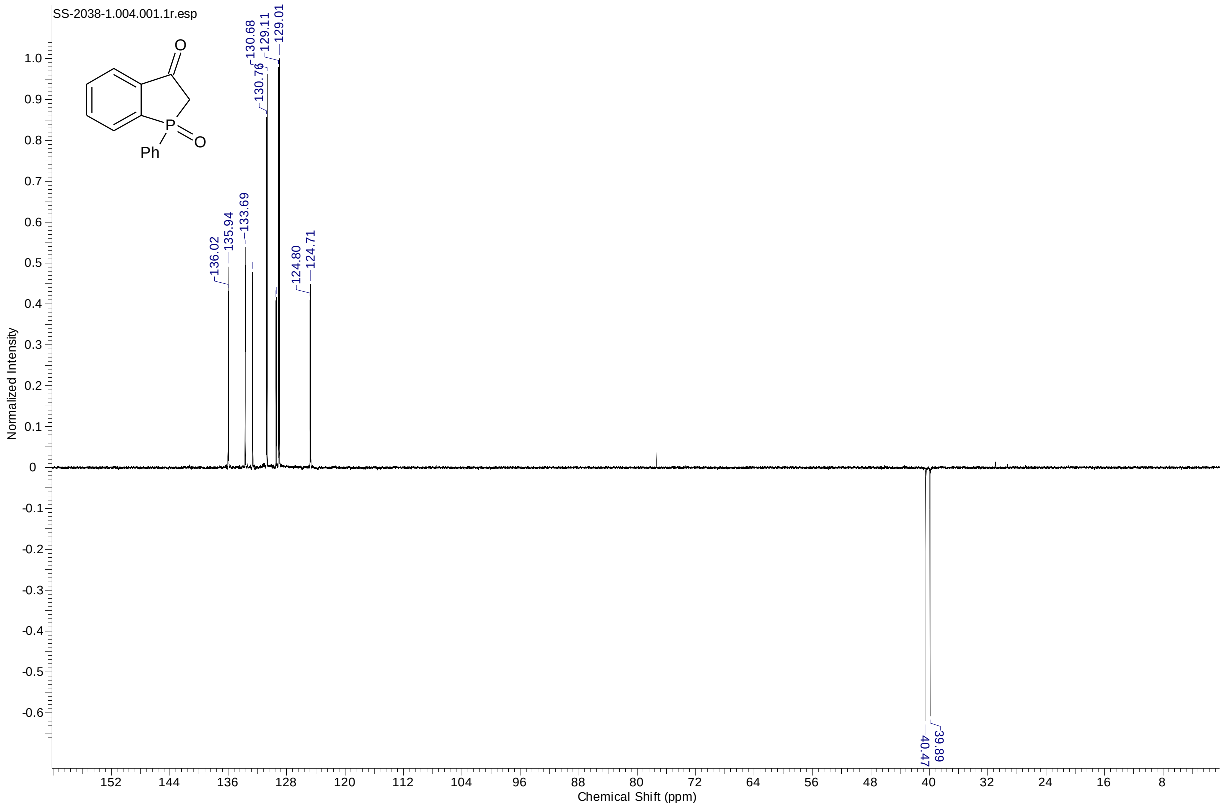

DEPT 135 NMR spectrum of benzophospholan-3-one oxide (**3a**) (202 MHz, $CDCl_3$)[9]

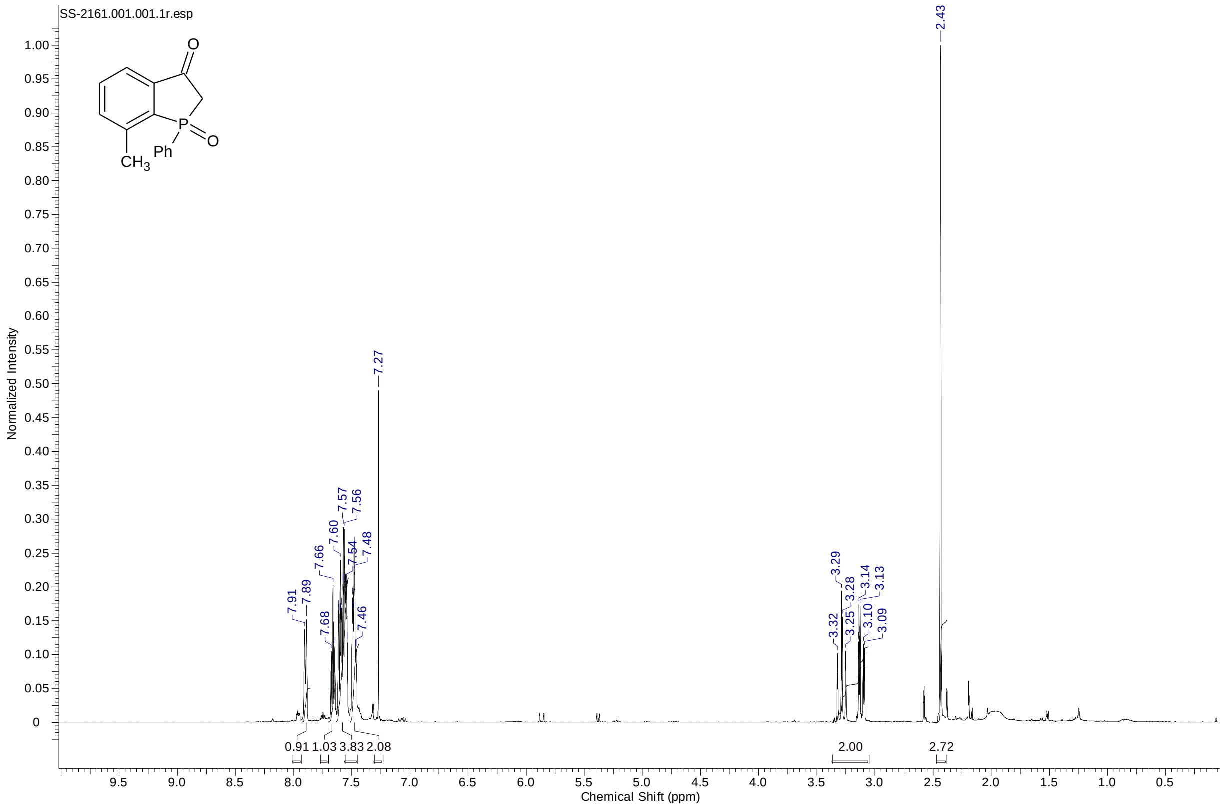

^1H NMR spectrum of 7-methylbenzophospholan-3-one oxide (**3b**) (500 MHz, $CDCl_3$)

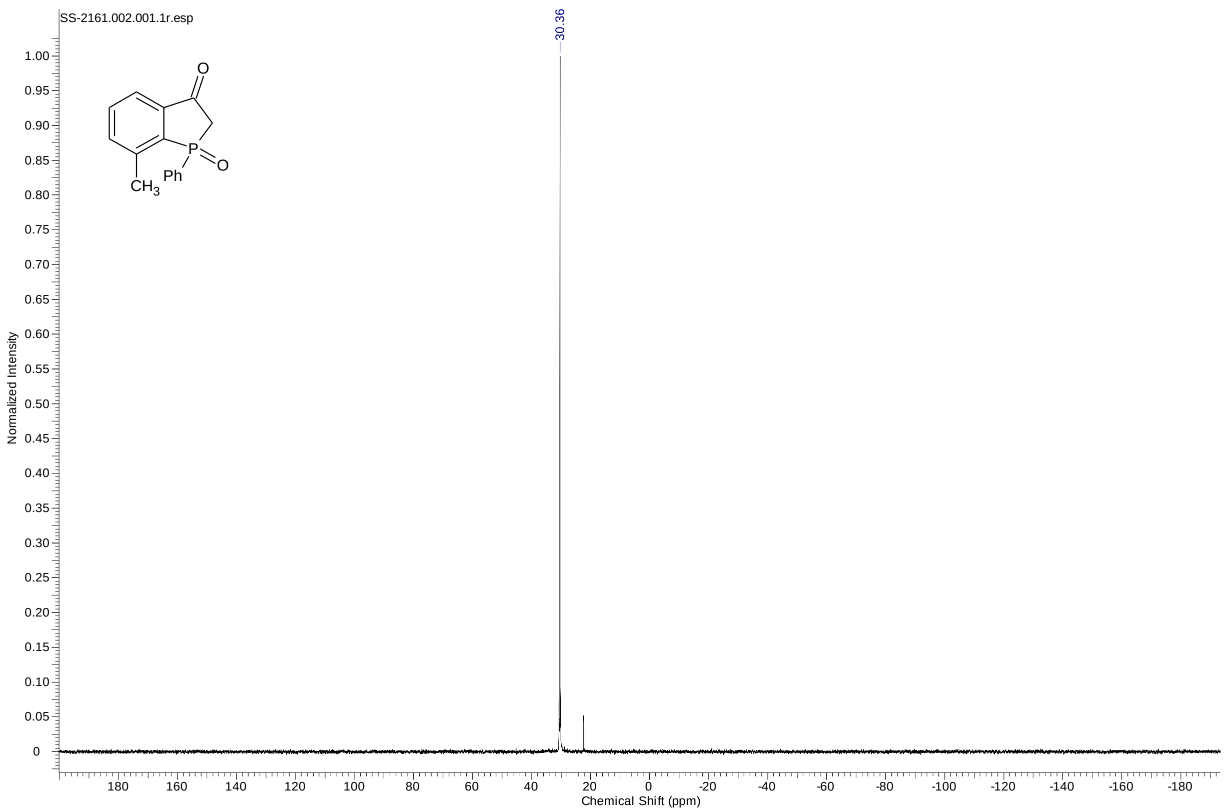

^{31}P NMR spectrum of 7-methylbenzophospholan-3-one oxide (**3b**) (202 MHz, $CDCl_3$)

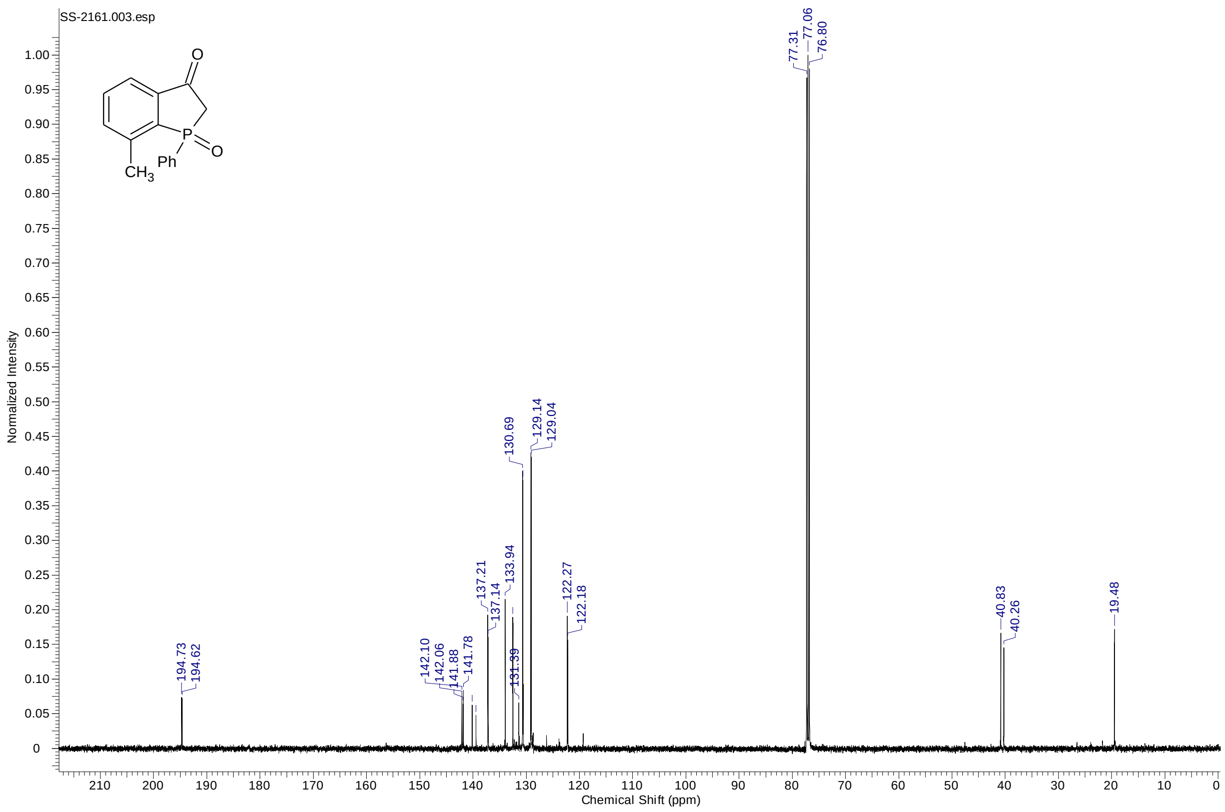

$^{13}C\{^1H\}$ NMR spectrum of 7-methylbenzophospholan-3-one oxide (**3b**) (125 MHz, $CDCl_3$)

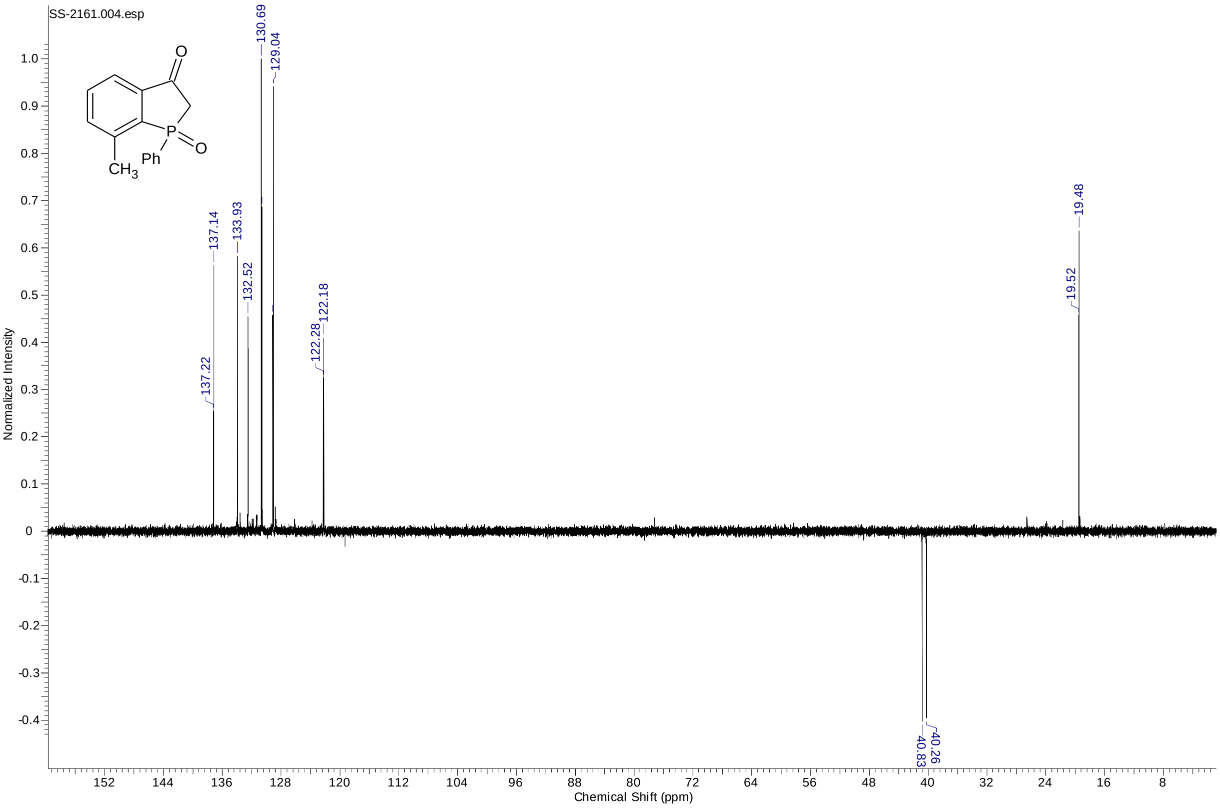

DEPT 135 NMR spectrum of 7-methylbenzophospholan-3-one oxide (**3b**) (125 MHz, $CDCl_3$)

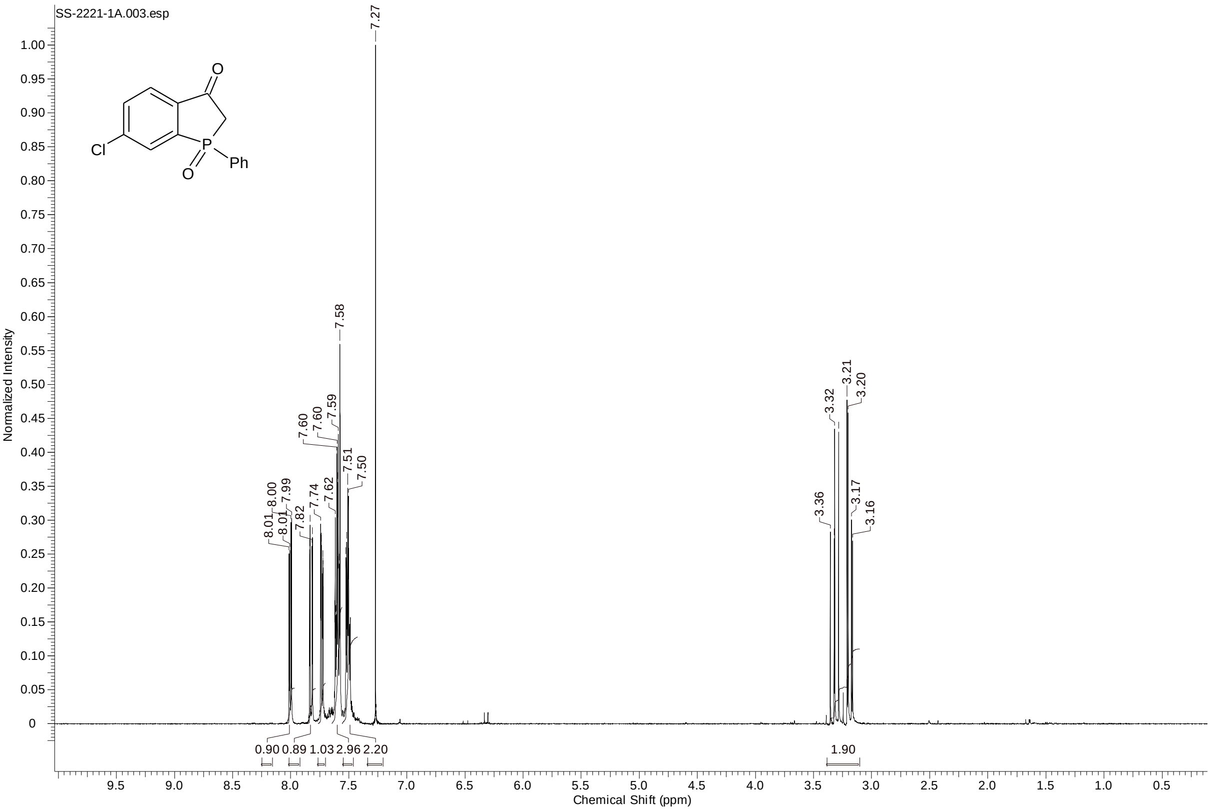

^{1}H NMR spectrum of 6-chlorobenzophospholan-3-one oxide (**3c**) (500 MHz, $CDCl_3$)

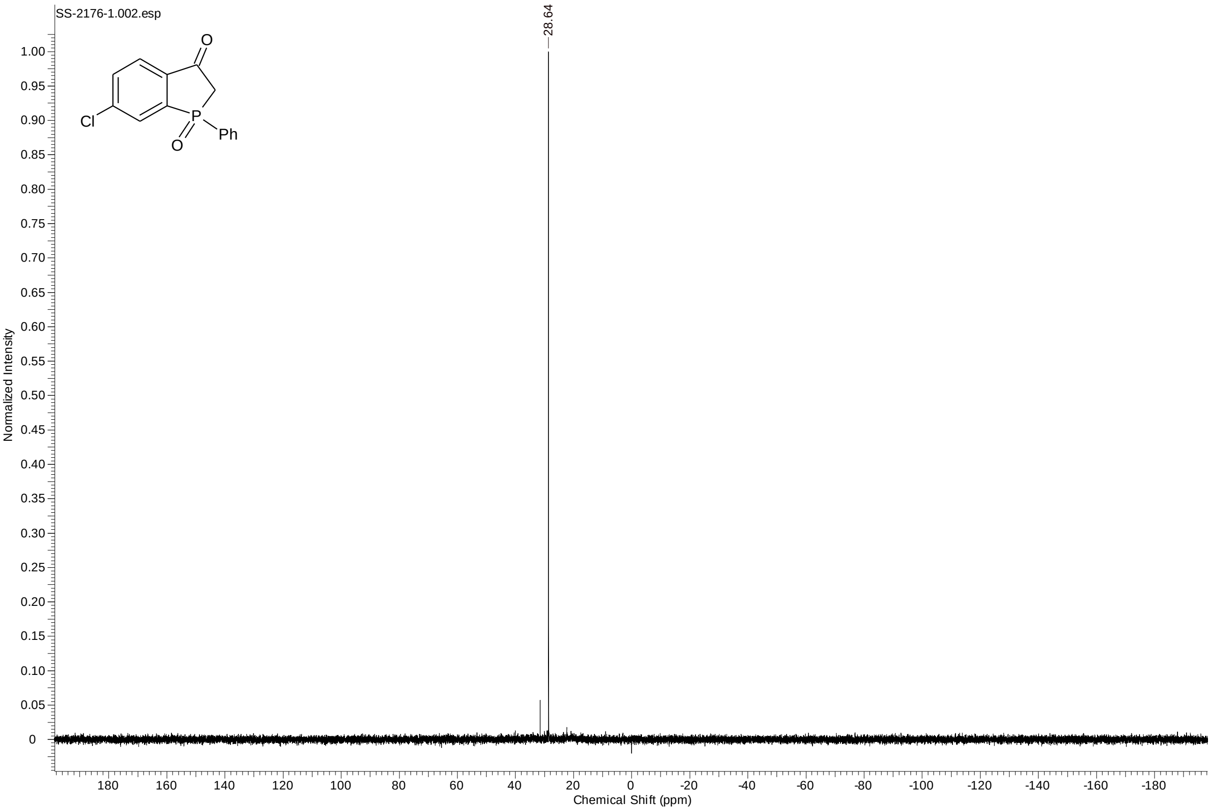

^{31}P NMR spectrum of 6-chlorobenzophospholan-3-one oxide (**3c**) (202 MHz, $CDCl_3$)

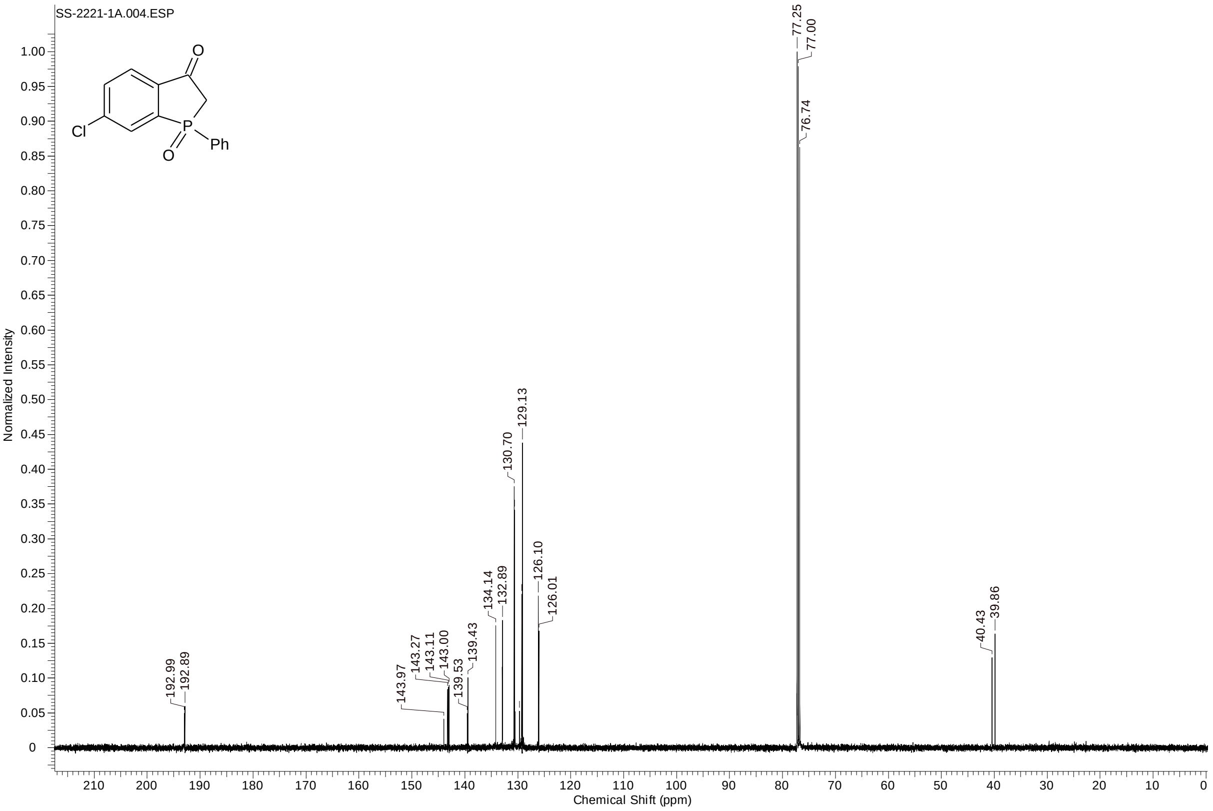

^{13}C{^{1}H} NMR spectrum of 6-chlorobenzophospholan-3-one oxide (**3c**) (125 MHz, $CDCl_3$)

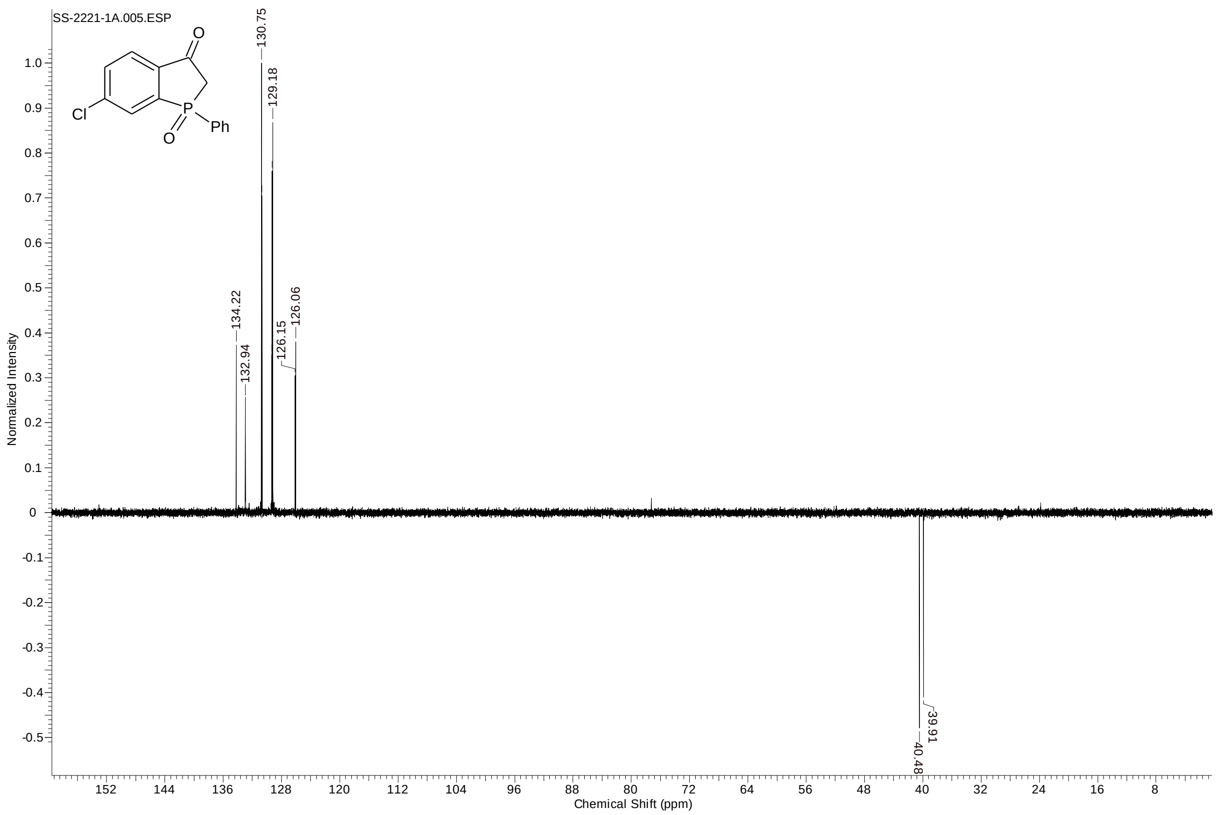

DEPT 135 NMR spectrum of 6-chlorobenzophospholan-3-one oxide (**3c**) (125 MHz, $CDCl_3$)

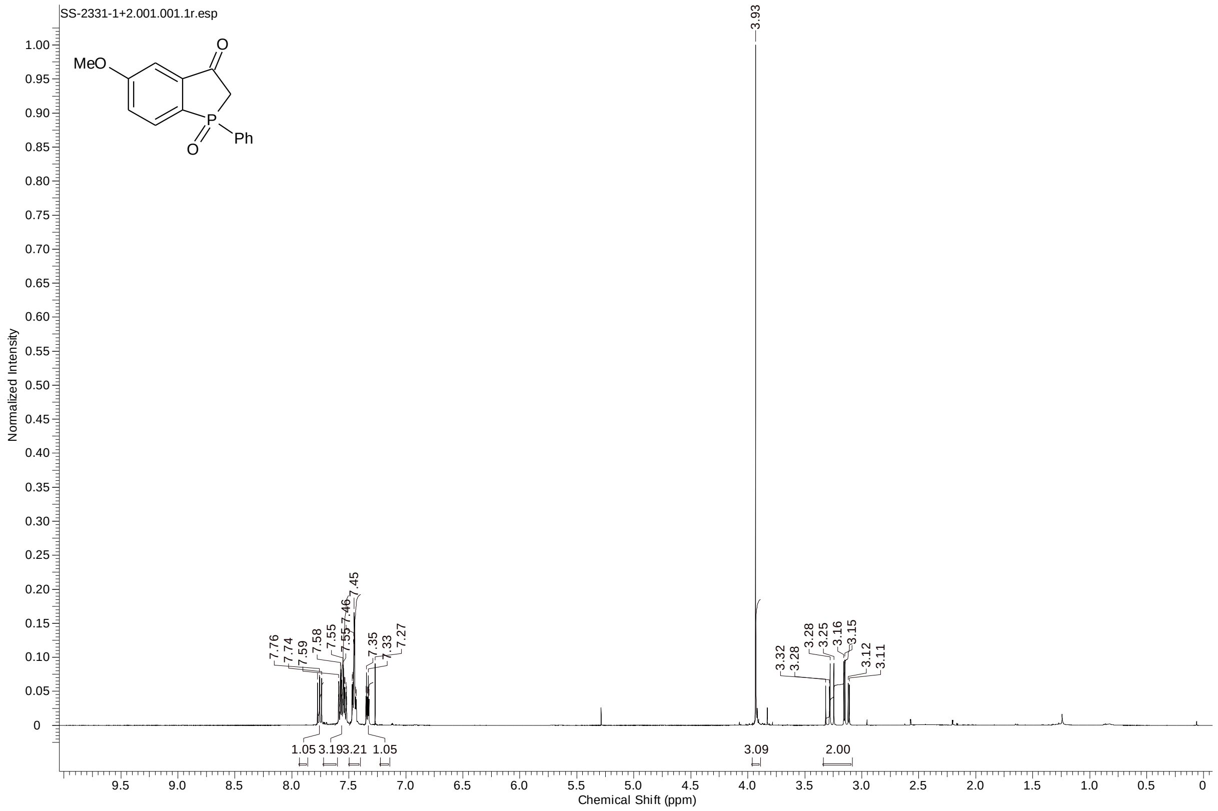

^{1}H NMR spectrum of 5-methoxybenzophospholan-3-one oxide (**3d**) (500 MHz, $CDCl_3$)

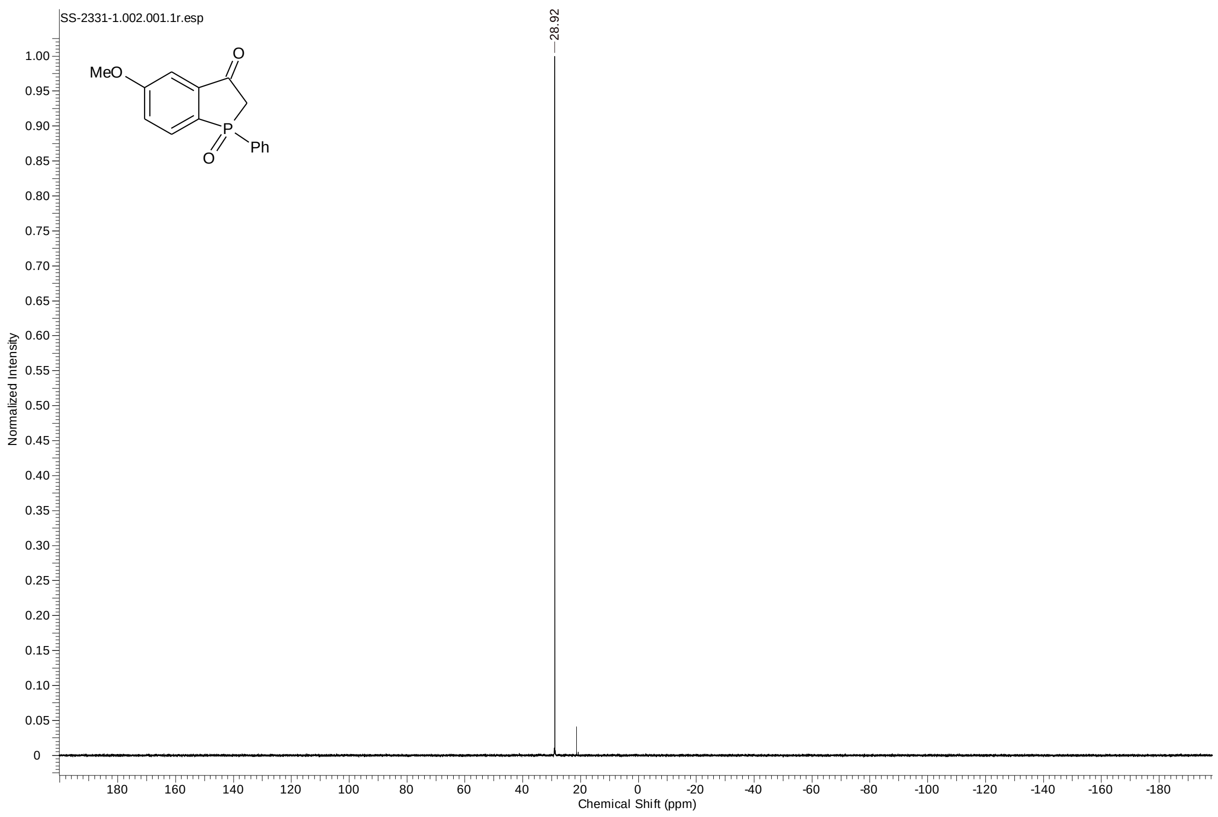

^{31}P NMR spectrum of 5-methoxybenzophospholan-3-one oxide (**3d**) (202 MHz, $CDCl_3$)

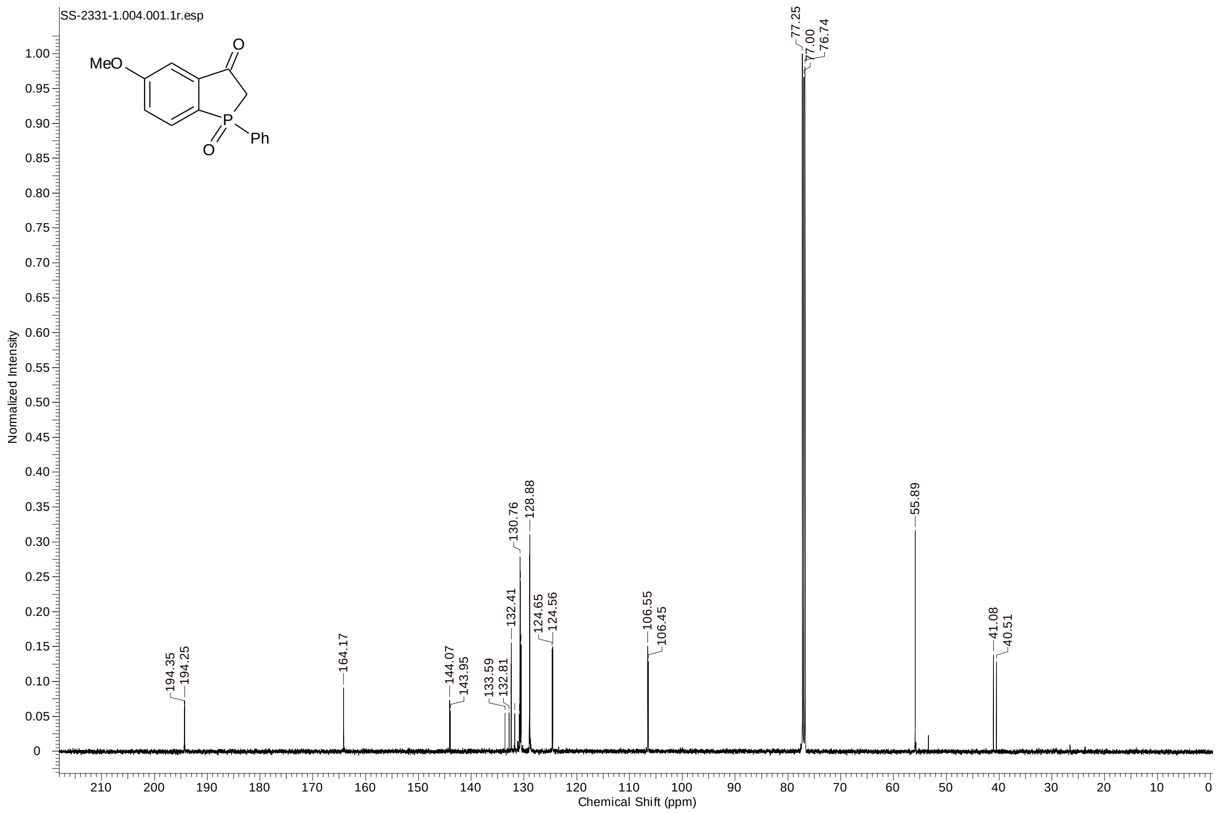

$^{13}C\{^1H\}$ NMR spectrum of 5-methoxybenzophospholan-3-one oxide (**3d**) (125 MHz, $CDCl_3$)

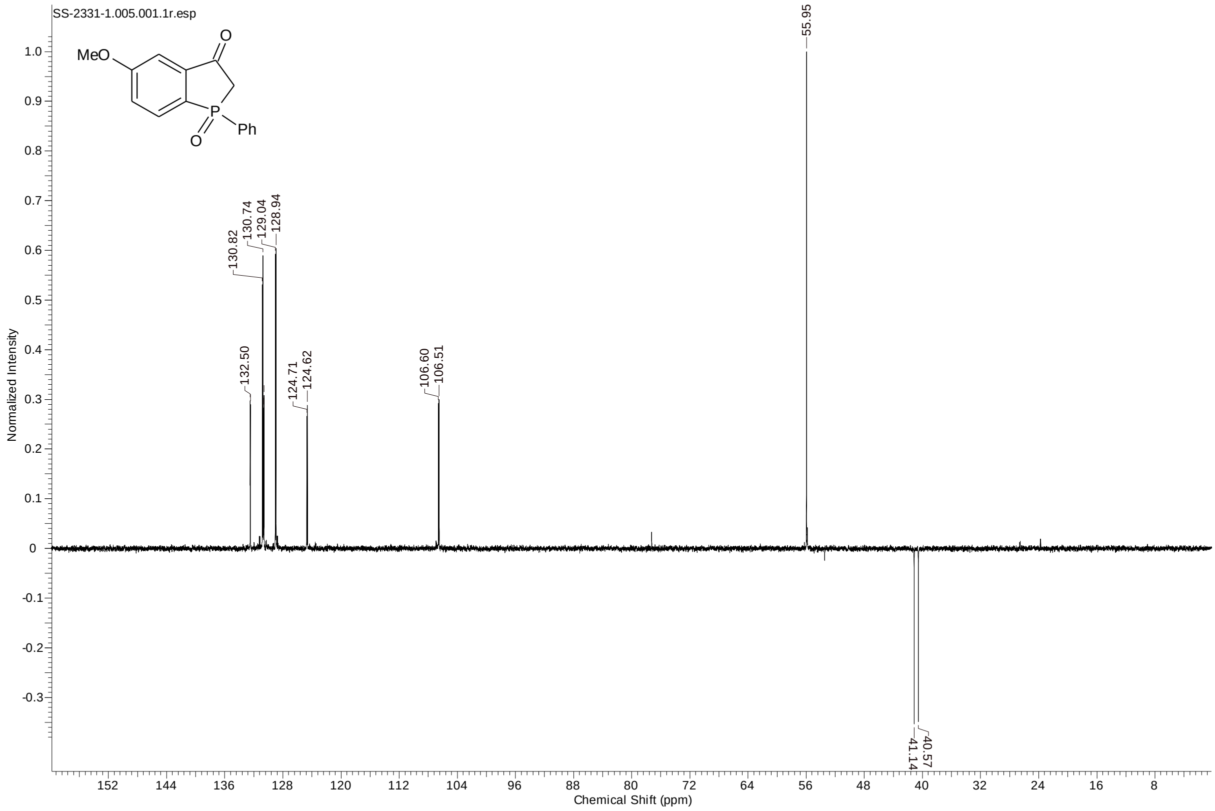

DEPT 135 NMR spectrum of 5-methoxybenzophospholan-3-one oxide (**3d**) (125 MHz, $CDCl_3$)

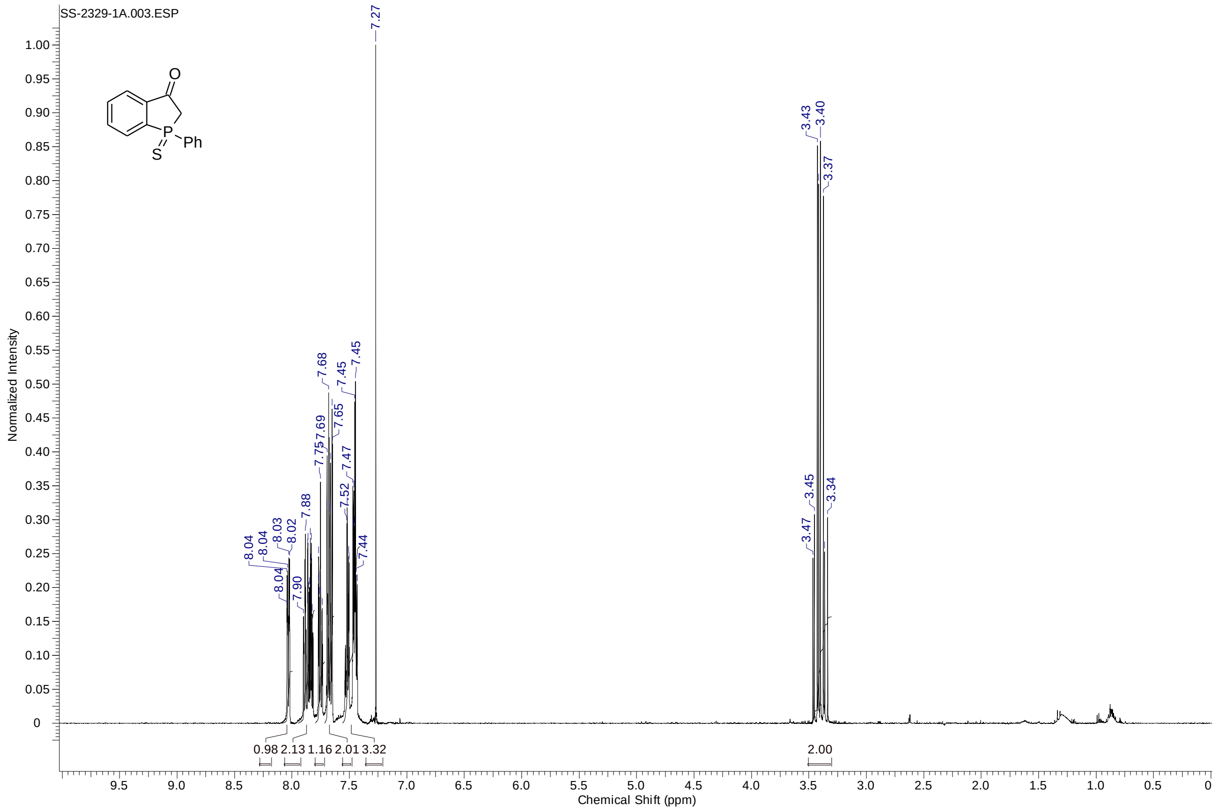

^{1}H NMR spectrum of benzophospholan-3-one sulfide (**4a**) (500 MHz, $CDCl_3$)

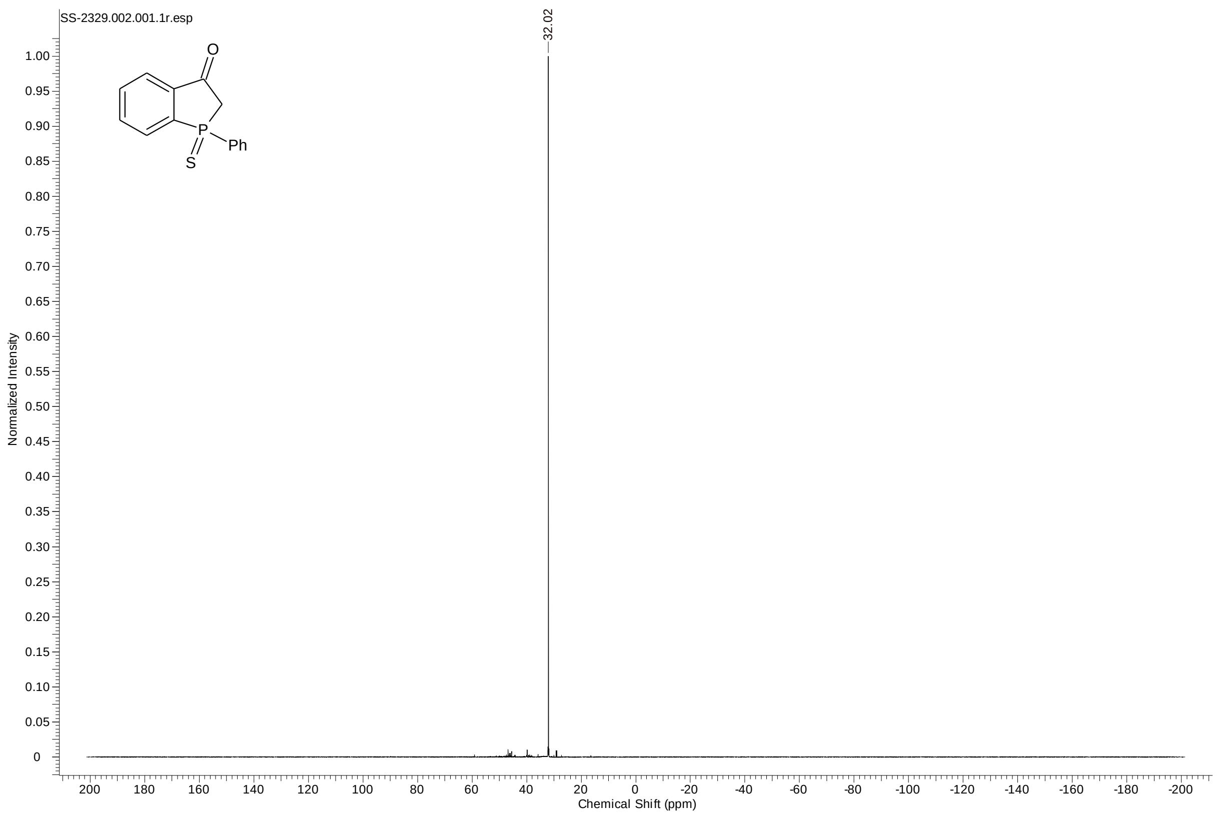

^{31}P NMR spectrum of benzophospholan-3-one sulfide (**4a**) (202 MHz, $CDCl_3$)

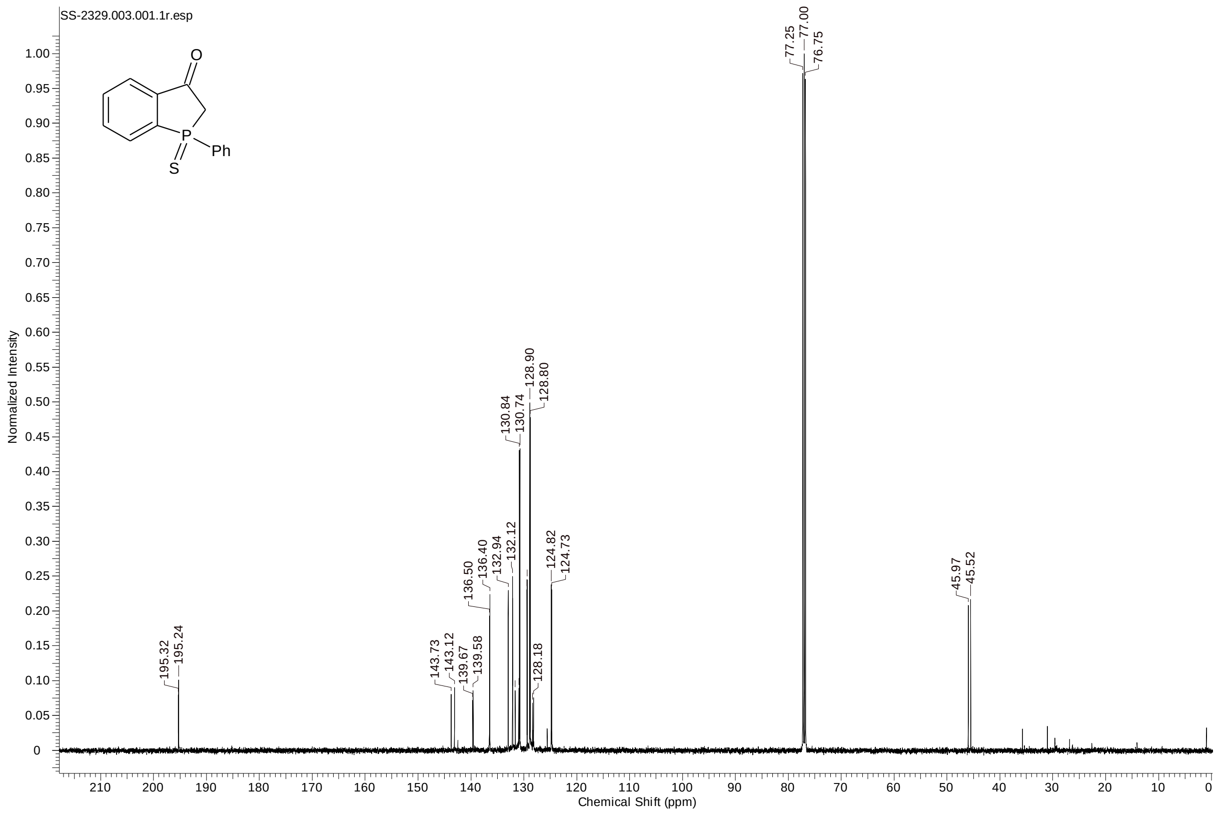

$^{13}C\{^1H\}$ NMR spectrum of benzophospholan-3-one sulfide (**4a**) (125 MHz, $CDCl_3$)

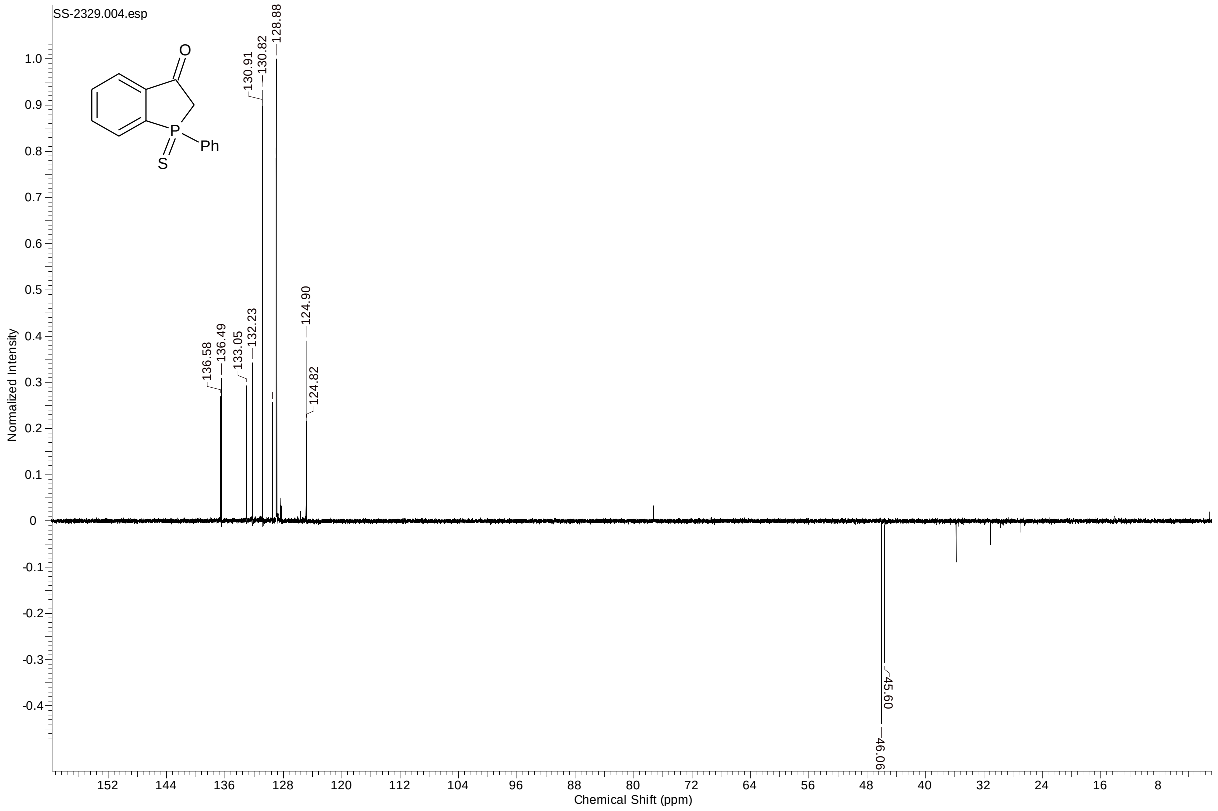

DEPT 135 NMR spectrum of benzophospholan-3-one sulfide (**4a**) (125 MHz, $CDCl_3$)

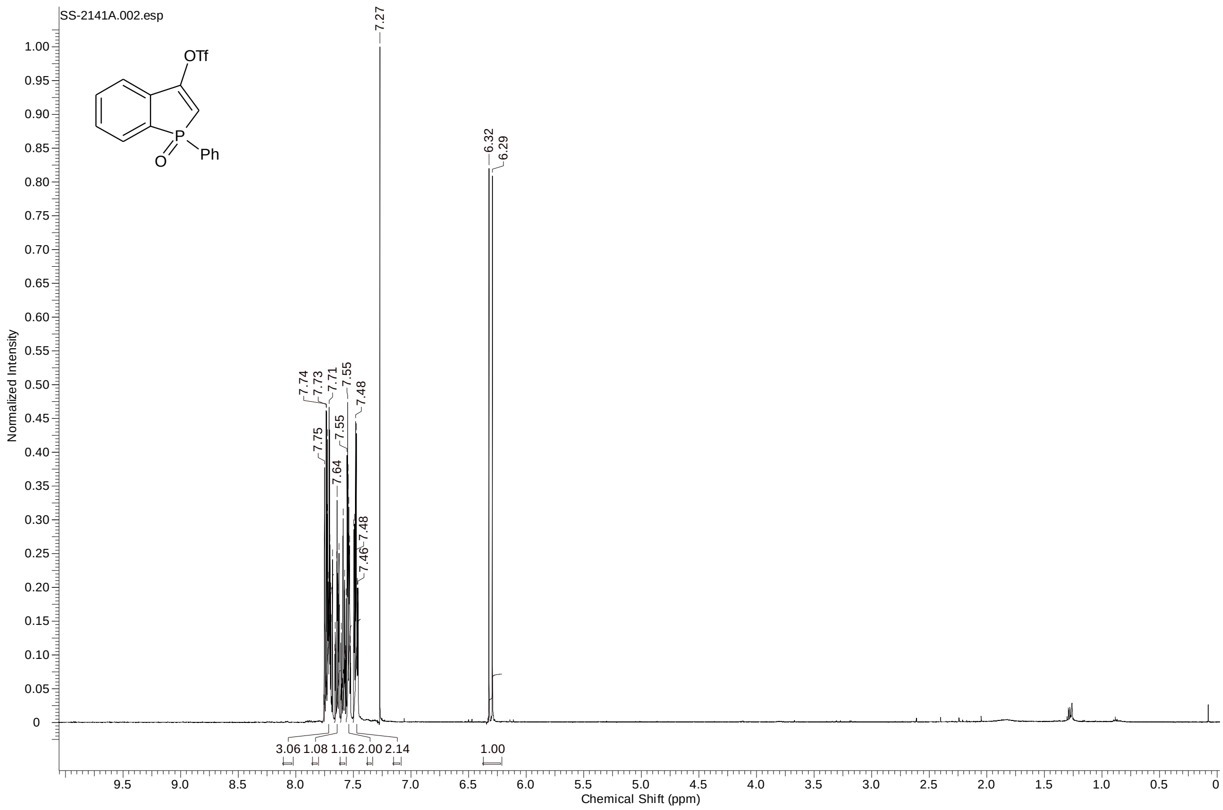

^{1}H NMR spectrum of 1-oxido-1-phenyl-1H-phosphindol-3-yl trifluoromethanesulfonate (**5a**) (500 MHz, $CDCl_3$)

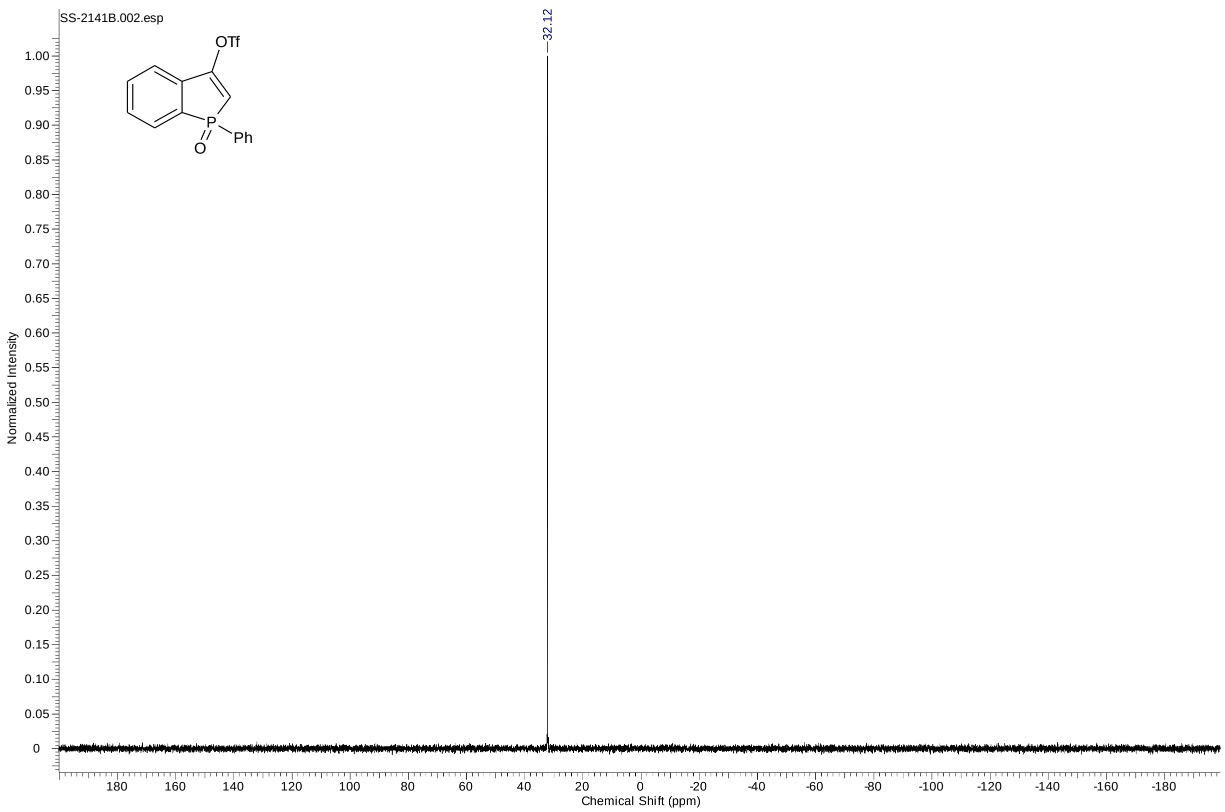

^{31}P NMR spectrum of 1-oxido-1-phenyl-1H-phosphindol-3-yl trifluoromethanesulfonate (**5a**) (202 MHz, $CDCl_3$)

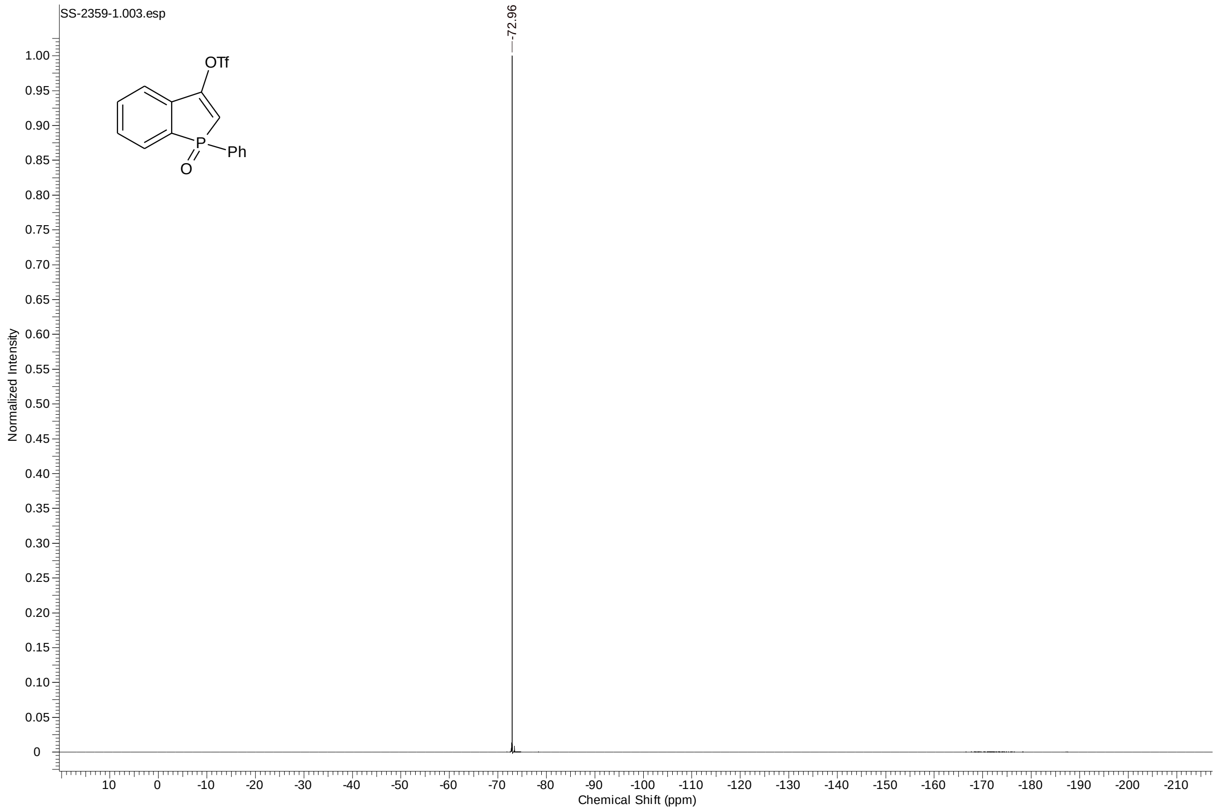

^{19}F NMR spectrum of 1-oxido-1-phenyl-1H-phosphindol-3-yl trifluoromethanesulfonate (**5a**) (471 MHz, $CDCl_3$)

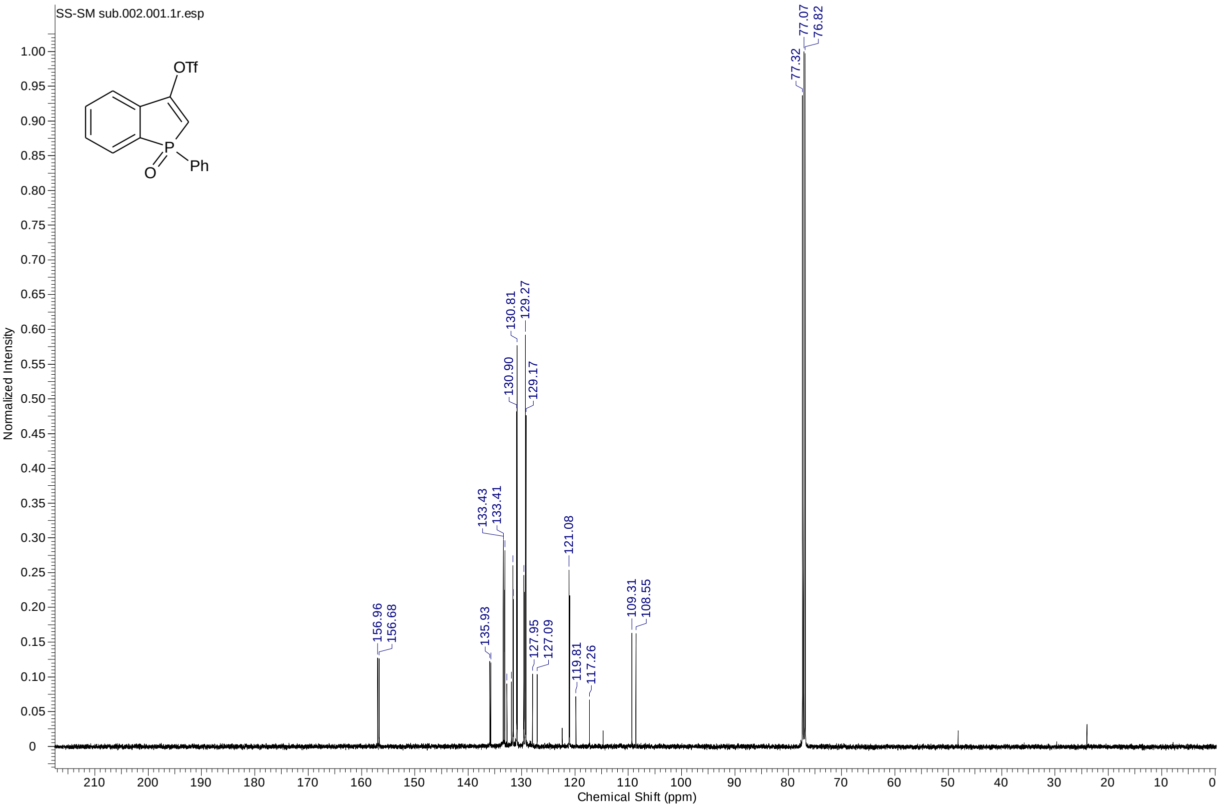

$^{13}C\{^{1}H\}$ NMR spectrum of 1-oxido-1-phenyl-1H-phosphindol-3-yl trifluoromethanesulfonate (**5a**) (125 MHz, $CDCl_3$)

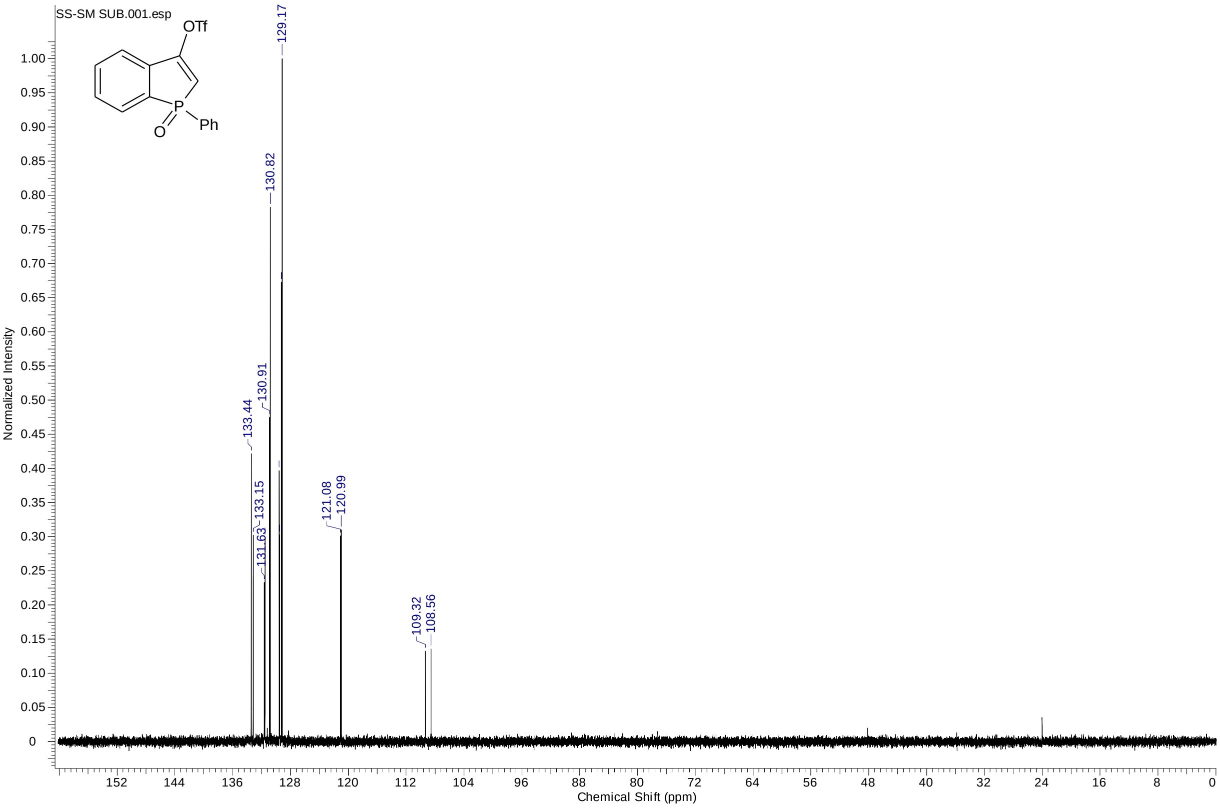

DEPT 135 NMR spectrum of 1-oxido-1-phenyl-1H-phosphindol-3-yl trifluoromethanesulfonate (**5a**) (125 MHz, $CDCl_3$)

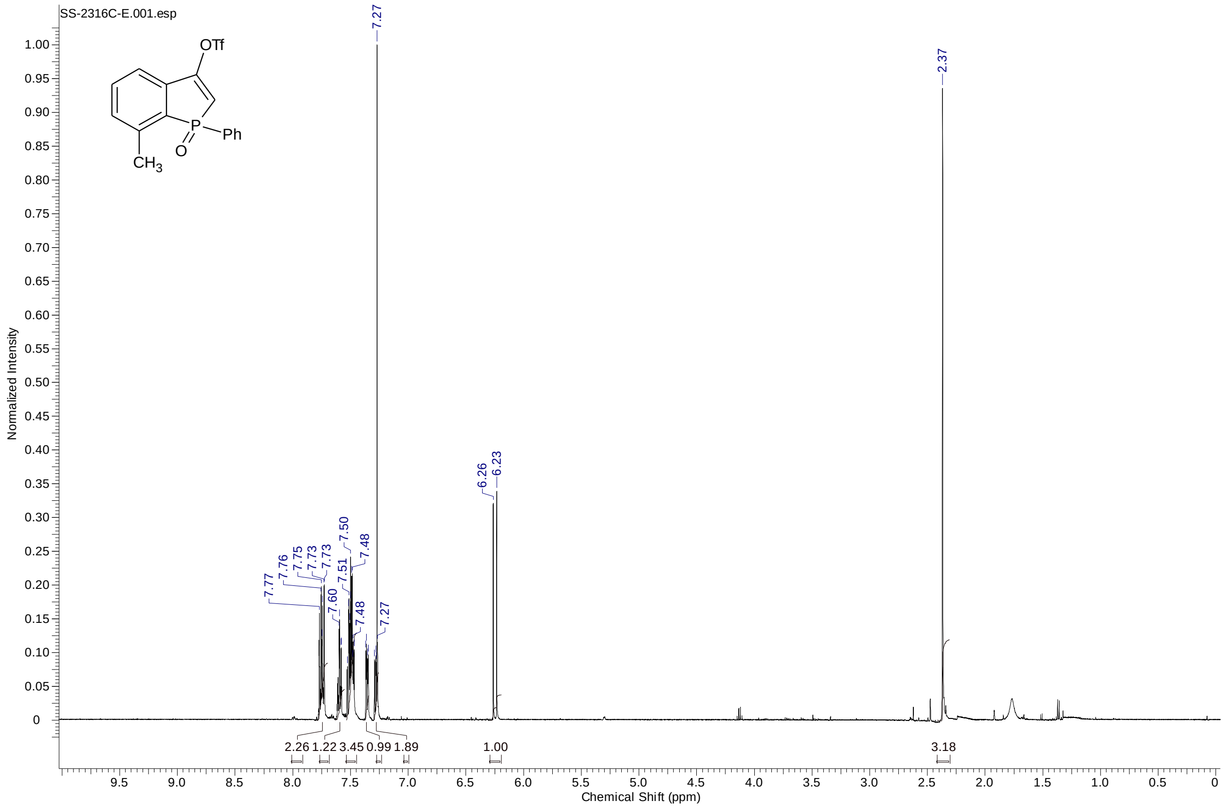

^{1}H NMR spectrum of 1-oxido-1-phenyl-1H-7-methylphosphindol-3-yl trifluoromethanesulfonate (**5b**) (500 MHz, $CDCl_3$)

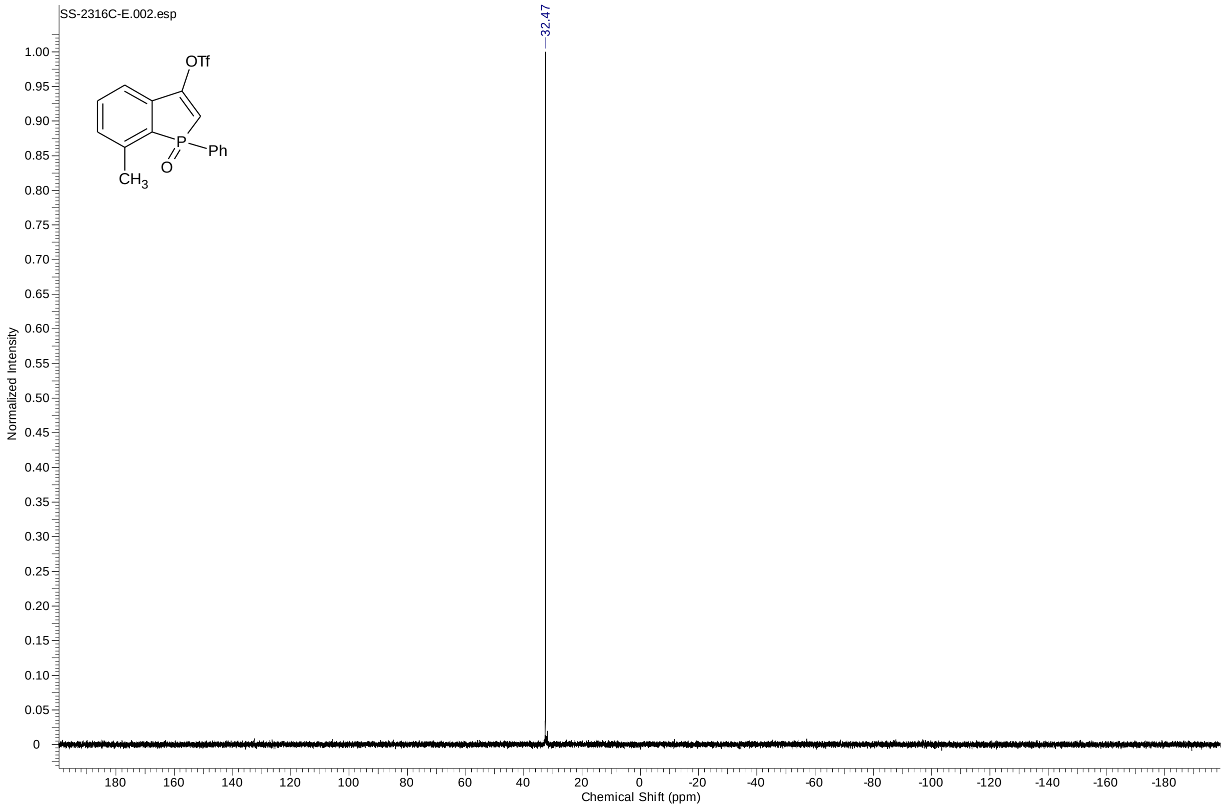

^{31}P NMR spectrum of 1-oxido-1-phenyl-1H-7-methylphosphindol-3-yl trifluoromethanesulfonate (**5b**) (202 MHz, $CDCl_3$)

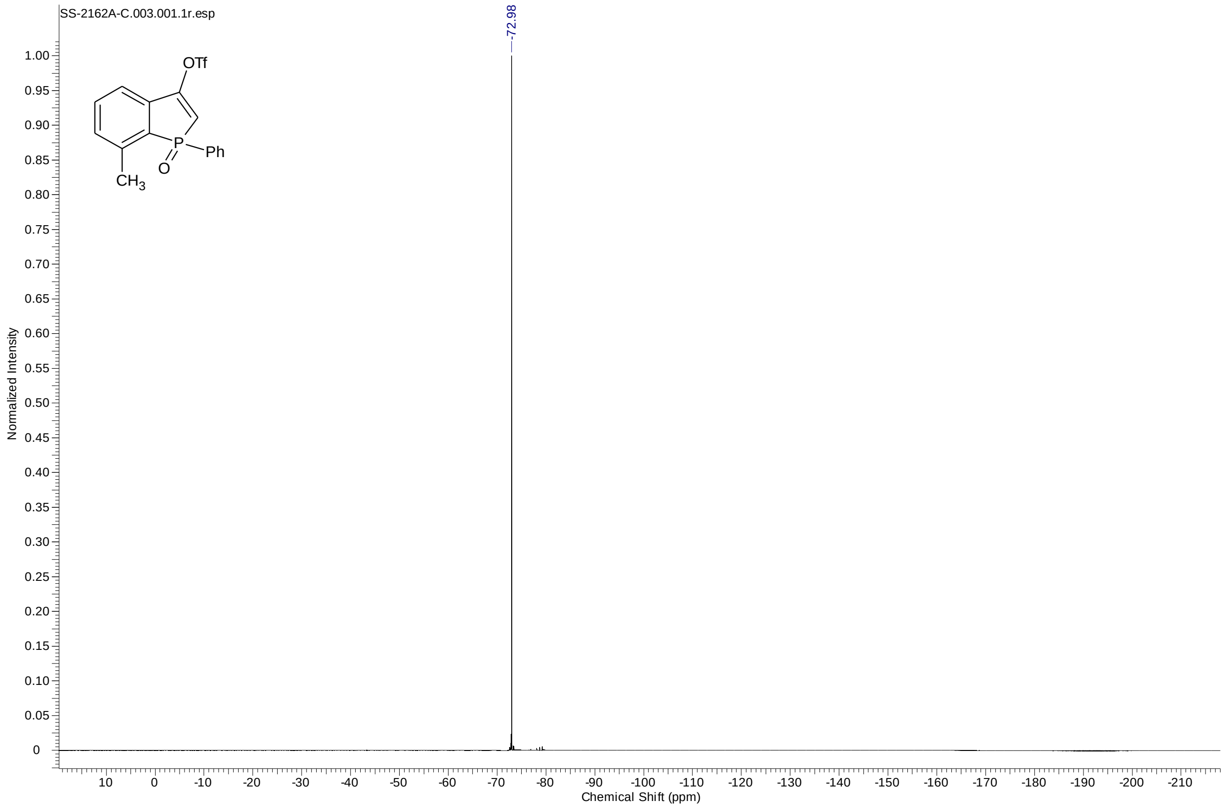

^{19}F NMR spectrum of 1-oxido-1-phenyl-1H-7-methylphosphindol-3-yl trifluoromethanesulfonate (**5b**) (471 MHz, $CDCl_3$)

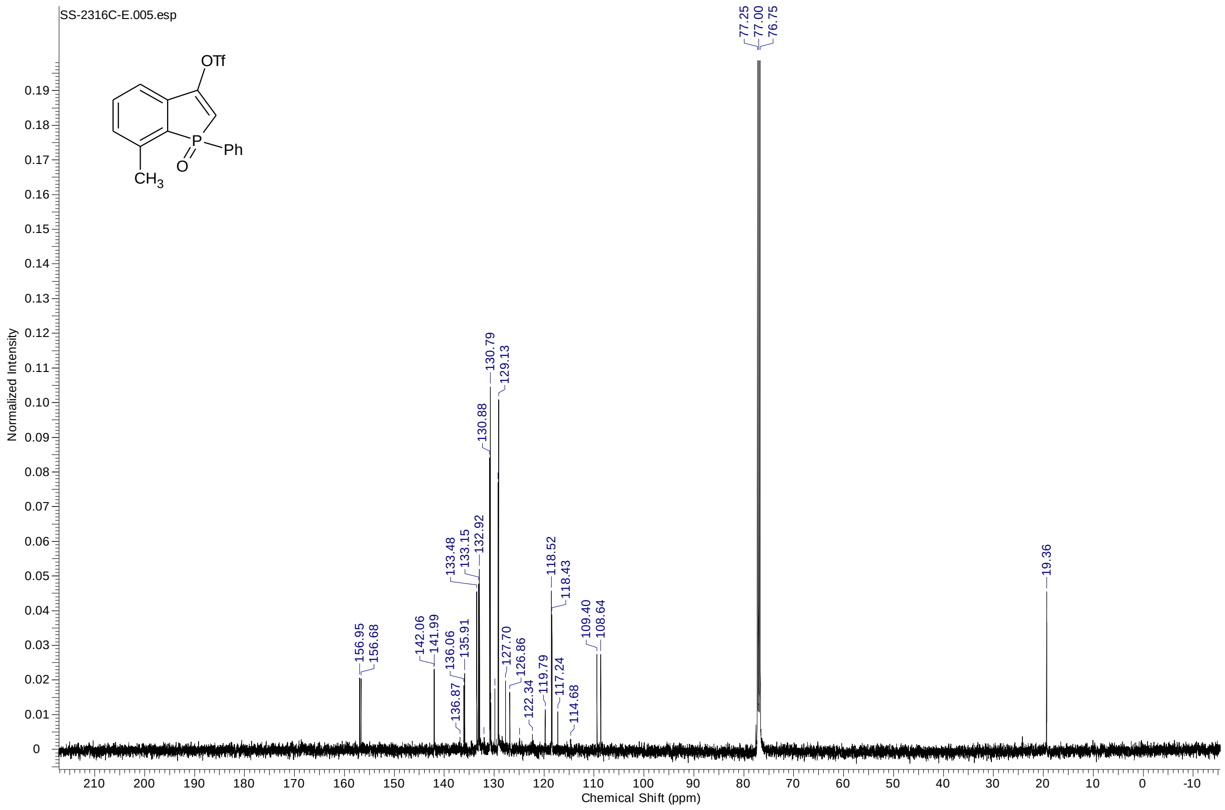

$^{13}C\{^1H\}$ NMR spectrum of 1-oxido-1-phenyl-1H-7-methylphosphindol-3-yl trifluoromethanesulfonate (**5b**) (125 MHz, $CDCl_3$)

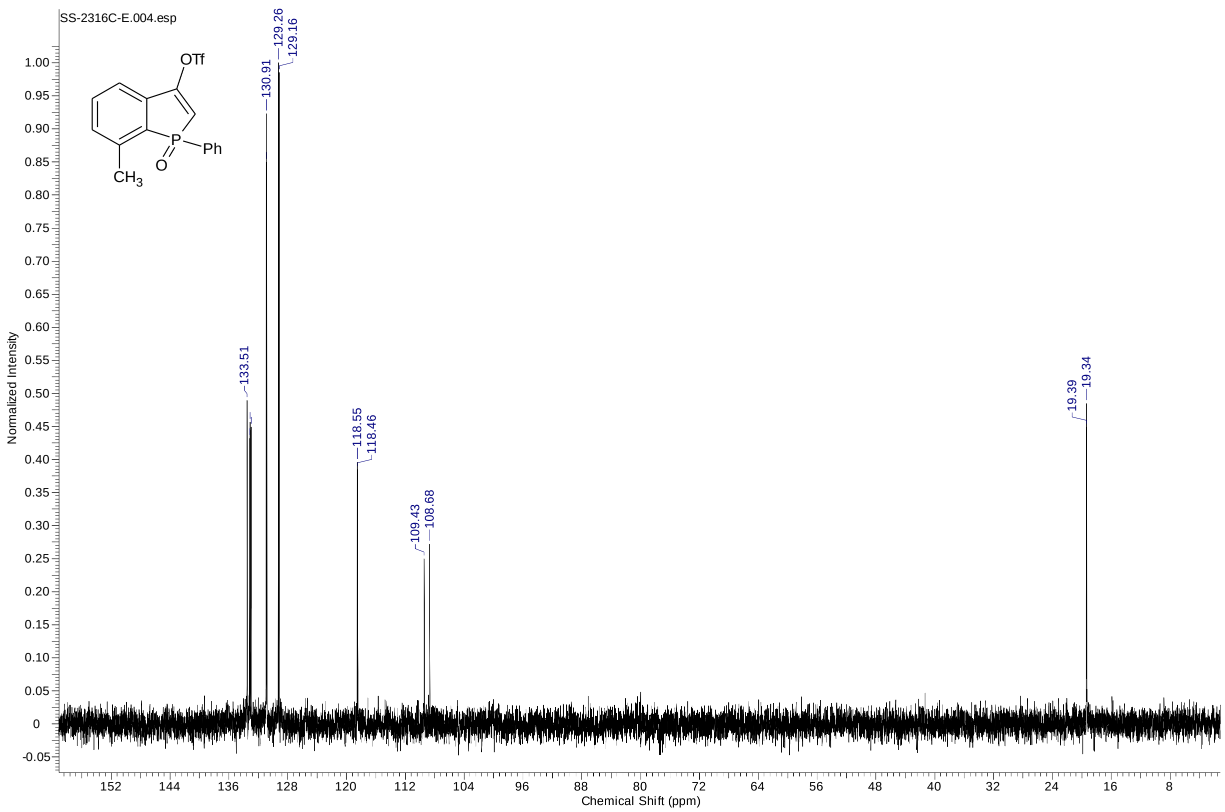

DEPT 135 NMR spectrum of 1-oxido-1-phenyl-1H-7-methylphosphindol-3-yl trifluoromethanesulfonate (**5b**) (125 MHz, $CDCl_3$)

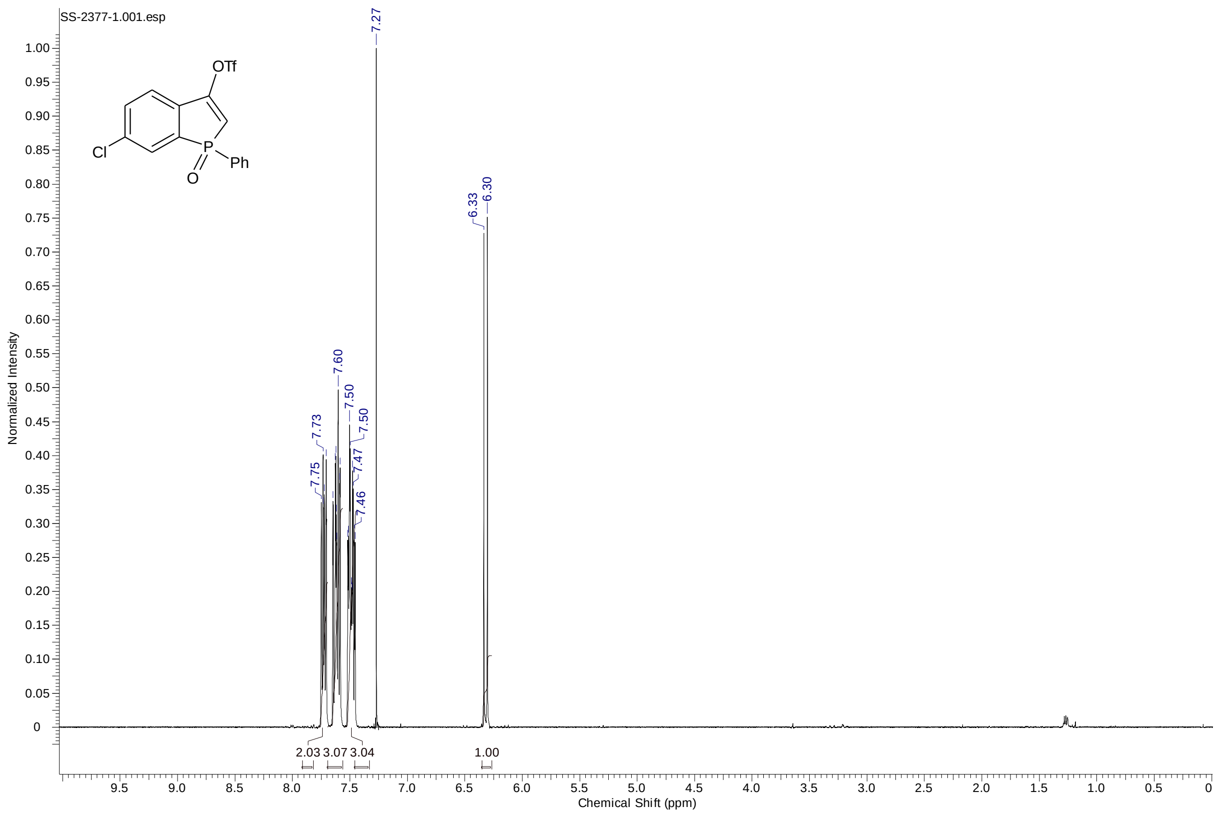

^{1}H NMR spectrum of 1-oxido-1-phenyl-1H-6-chlorophosphindol-3-yl trifluoromethanesulfonate (**5c**) (500 MHz, $CDCl_3$)

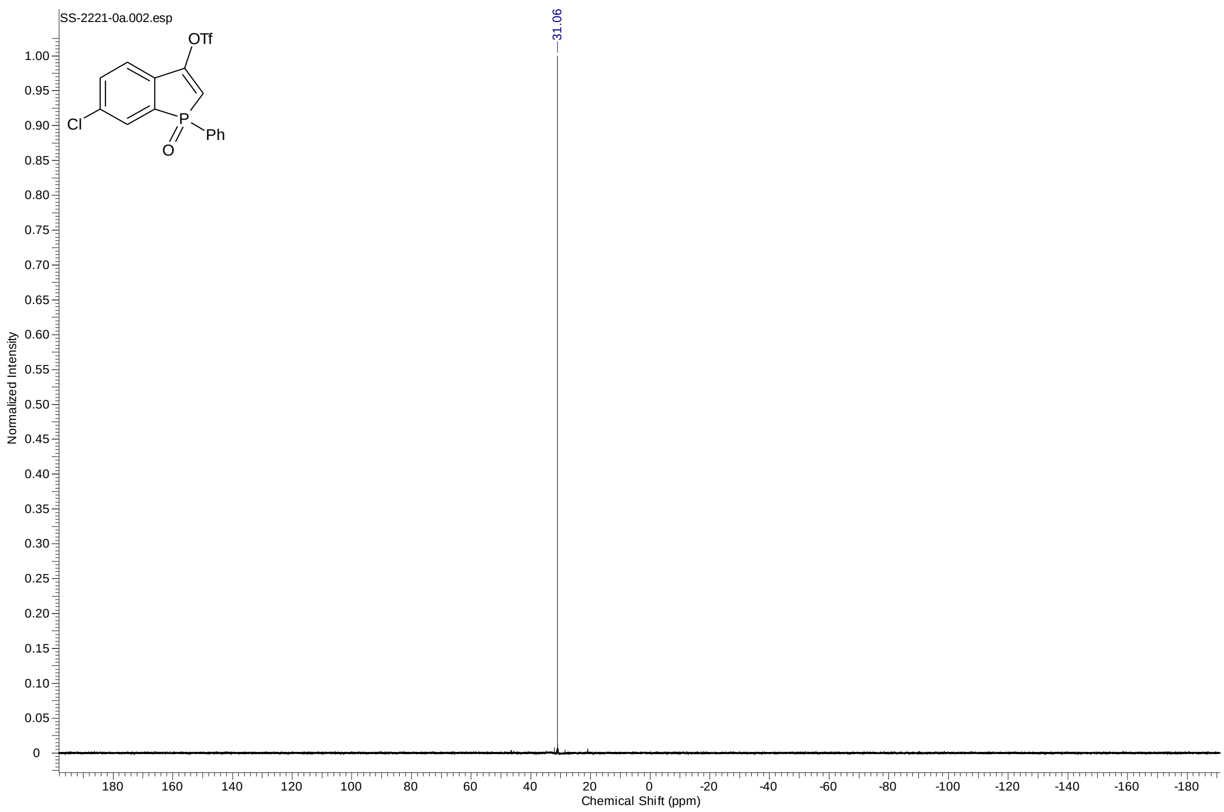

^{31}P NMR spectrum of 1-oxido-1-phenyl-1H-6-chlorophosphindol-3-yl trifluoromethanesulfonate (**5c**) (202 MHz, $CDCl_3$)

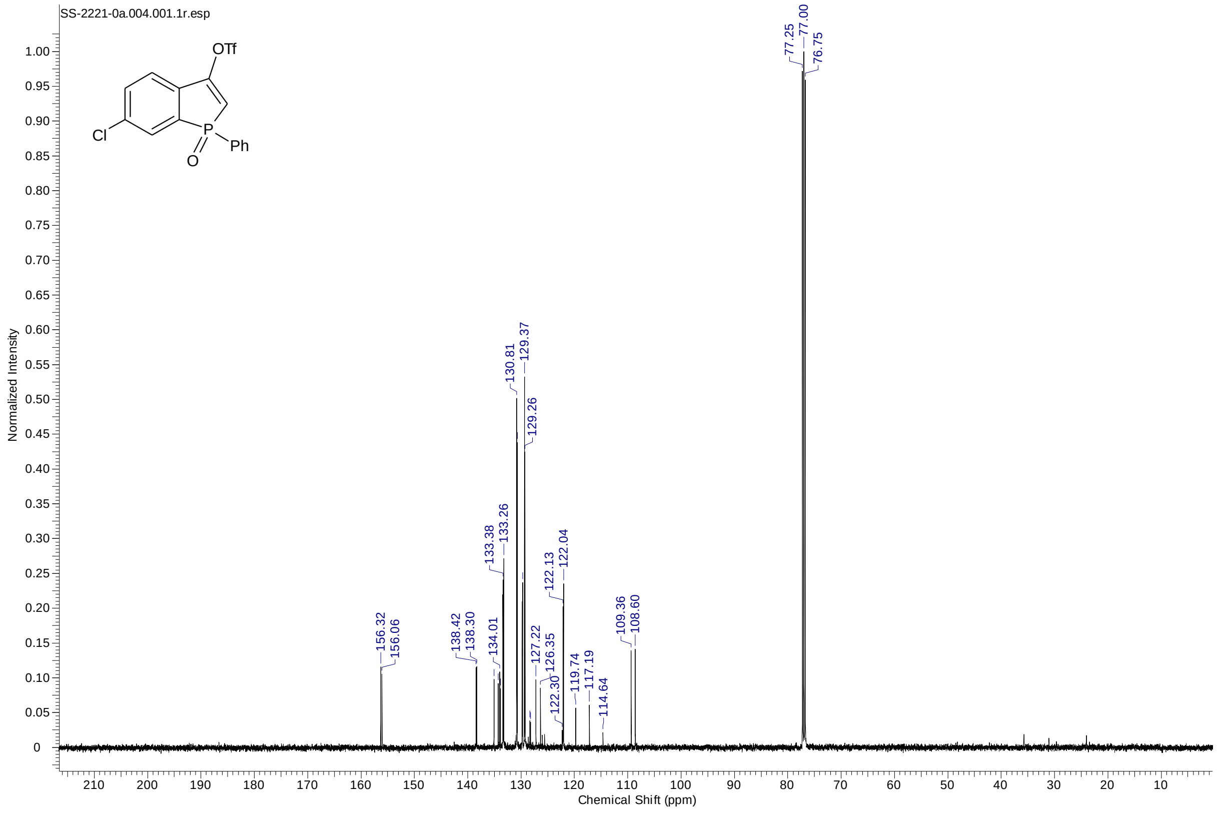

^{13}C{^{1}H} NMR spectrum of 1-oxido-1-phenyl-1H-6-chlorophosphindol-3-yl trifluoromethanesulfonate (**5c**) (125 MHz, $CDCl_3$)

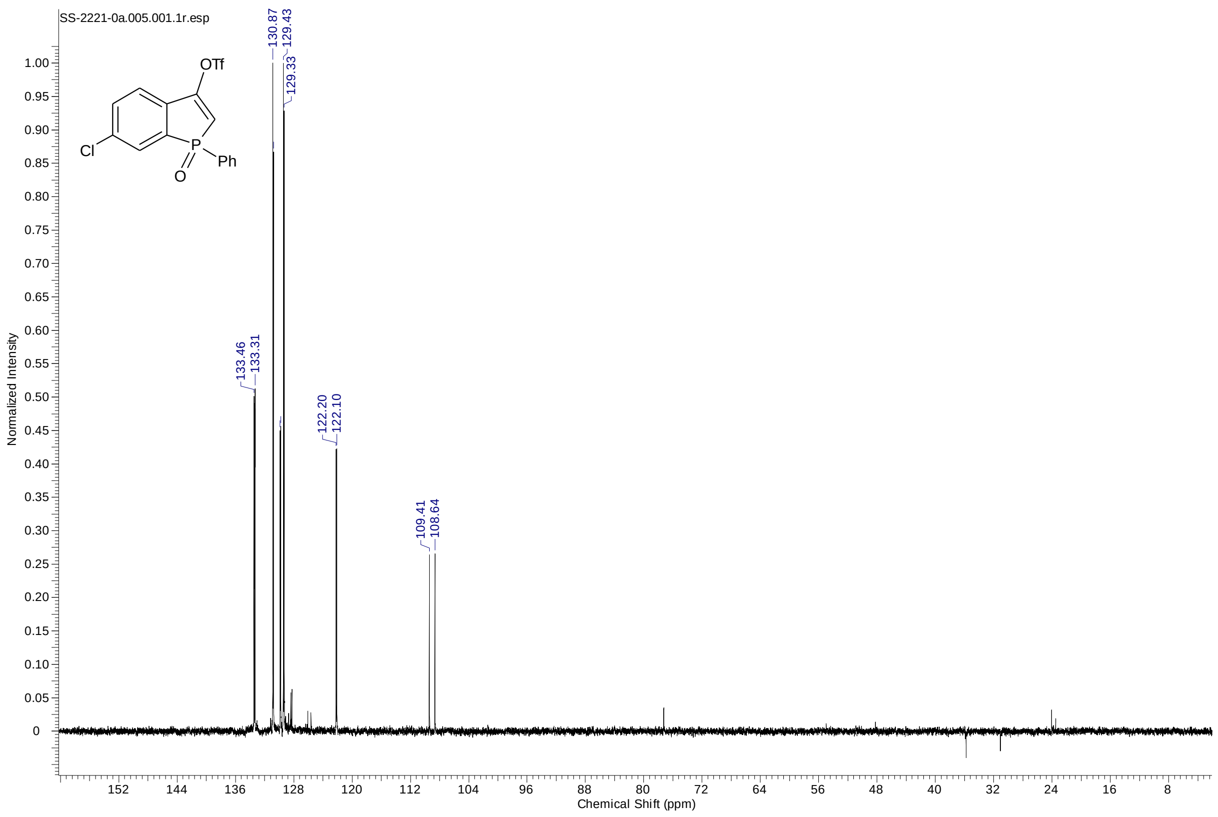

DEPT 135 NMR spectrum of 1-oxido-1-phenyl-1H-6-chlorophosphindol-3-yl trifluoromethanesulfonate (**5c**) (125 MHz, $CDCl_3$)

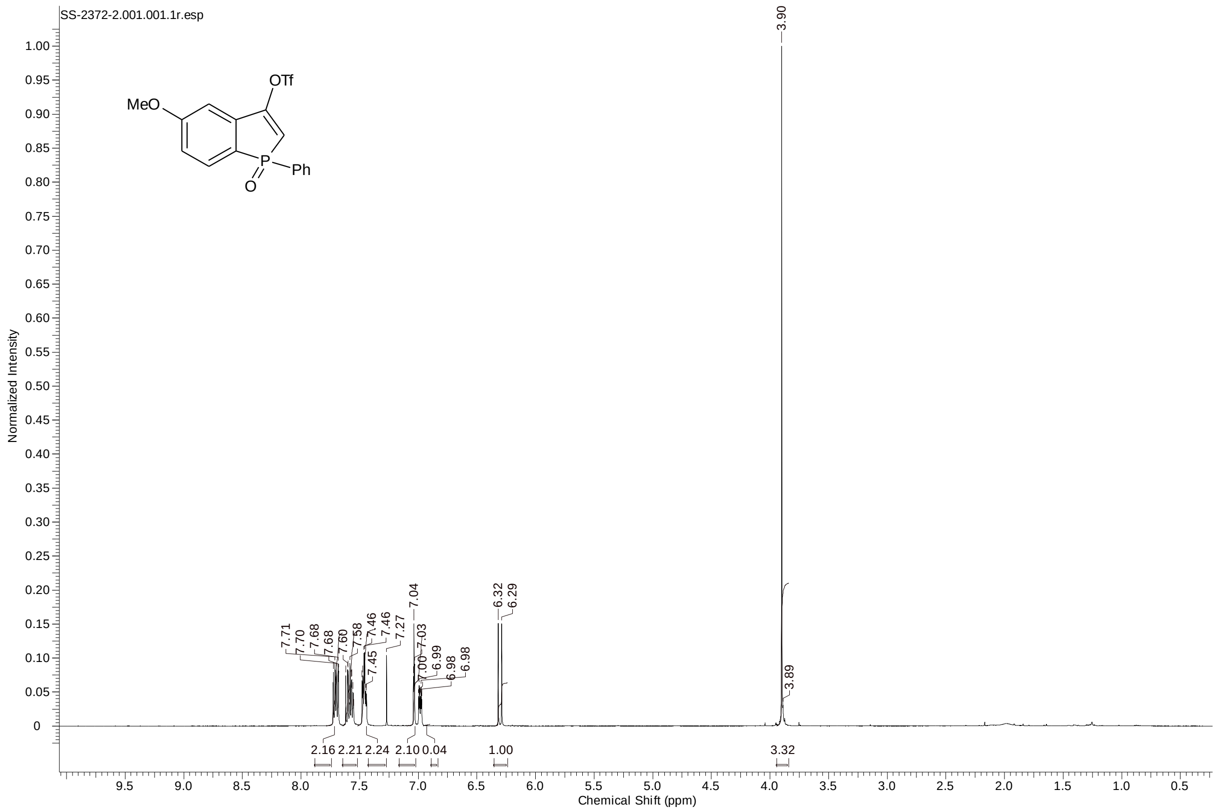

^1H NMR spectrum of 1-oxido-1-phenyl-1H-5-methoxyphosphindol-3-yl trifluoromethanesulfonate (**5d**) (500 MHz, $CDCl_3$)

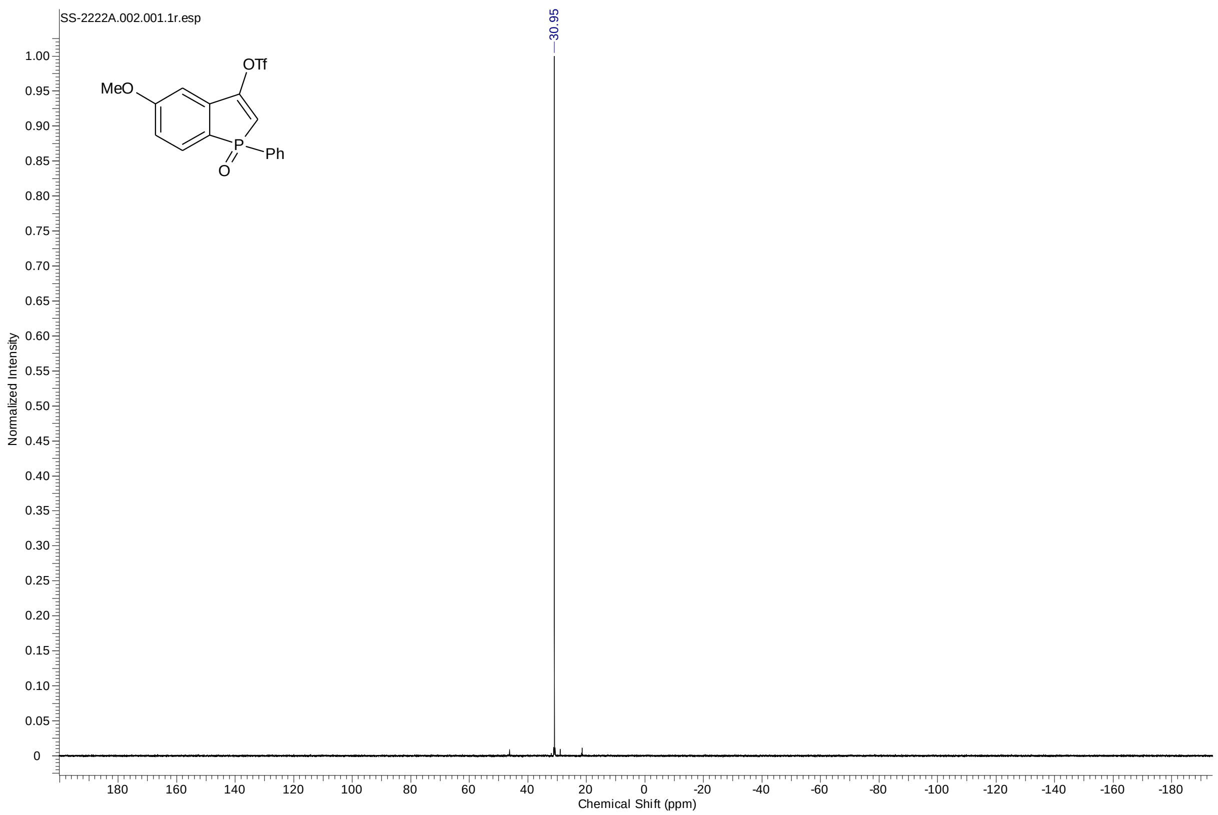

^{31}P NMR spectrum of 1-oxido-1-phenyl-1H-5-methoxyphosphindol-3-yl trifluoromethanesulfonate (**5d**) (202 MHz, $CDCl_3$)

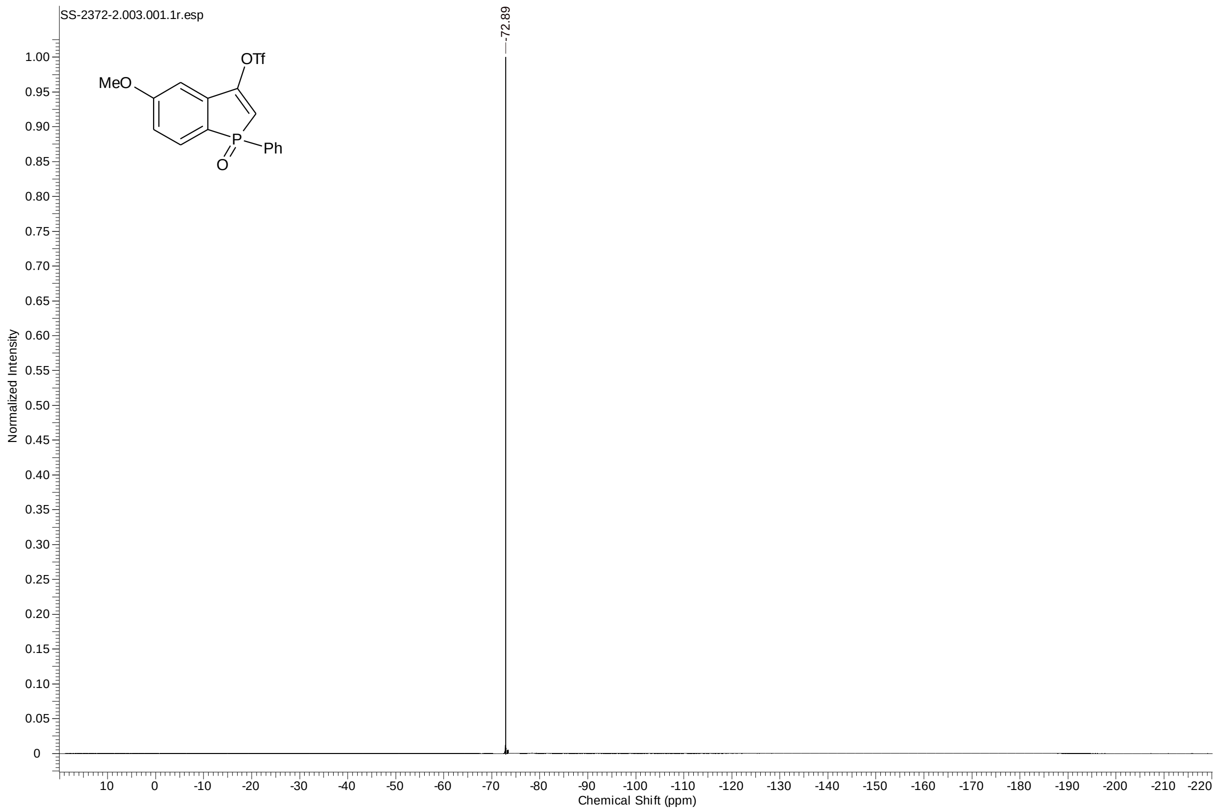

^{19}F NMR spectrum of 1-oxido-1-phenyl-1H-5-methoxyphosphindol-3-yl trifluoromethanesulfonate (**5d**) (471 MHz, $CDCl_3$)

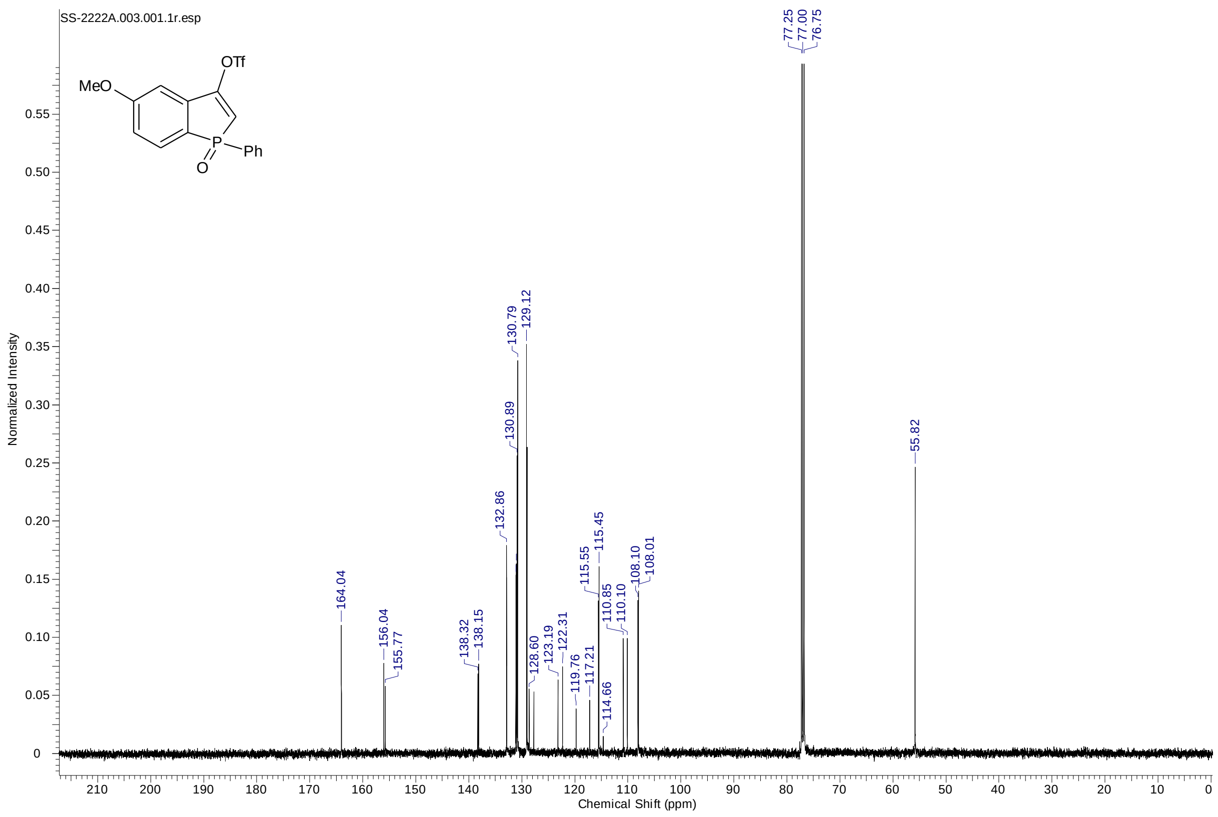

$^{13}C\{^{1}H\}$ NMR spectrum of 1-oxido-1-phenyl-1H-5-methoxyphosphindol-3-yl trifluoromethanesulfonate (**5d**) (125 MHz, $CDCl_3$)

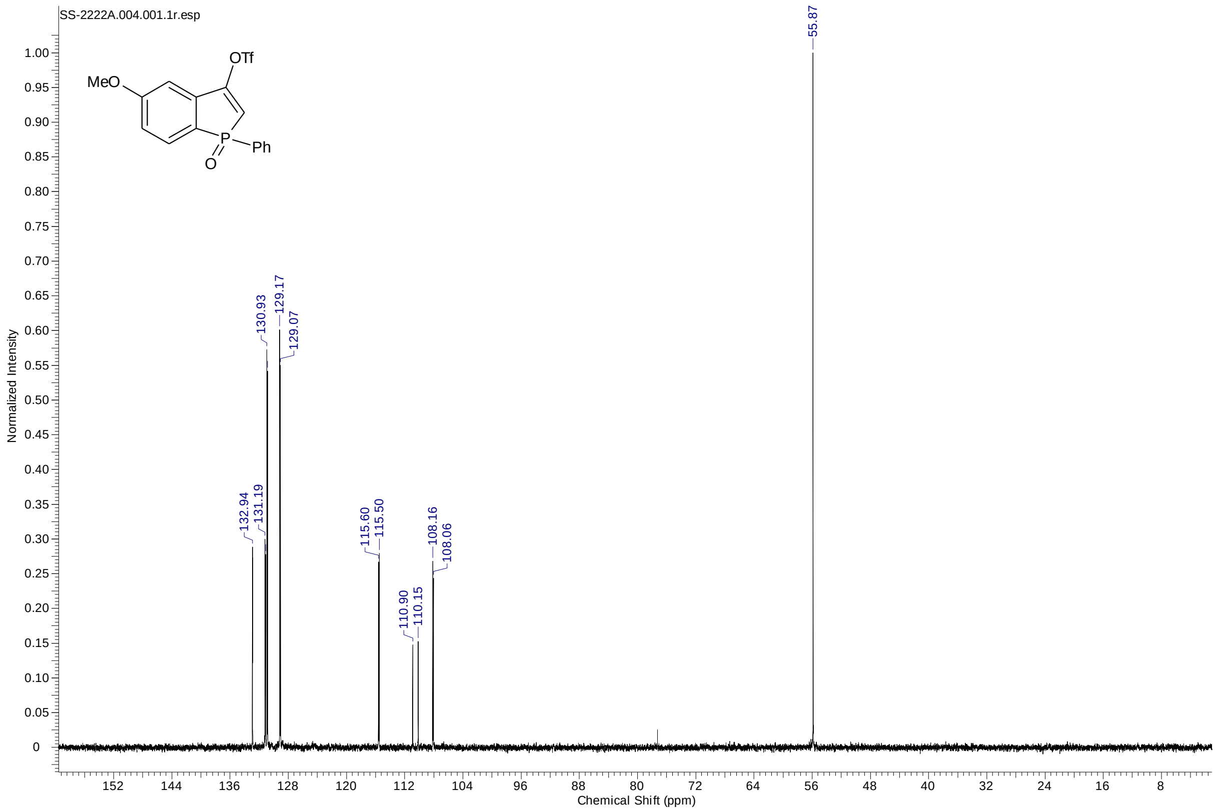

DEPT 135 NMR spectrum of 1-oxido-1-phenyl-1H-5-methoxyphosphindol-3-yl trifluoromethanesulfonate (**5d**) (125 MHz, $CDCl_3$)

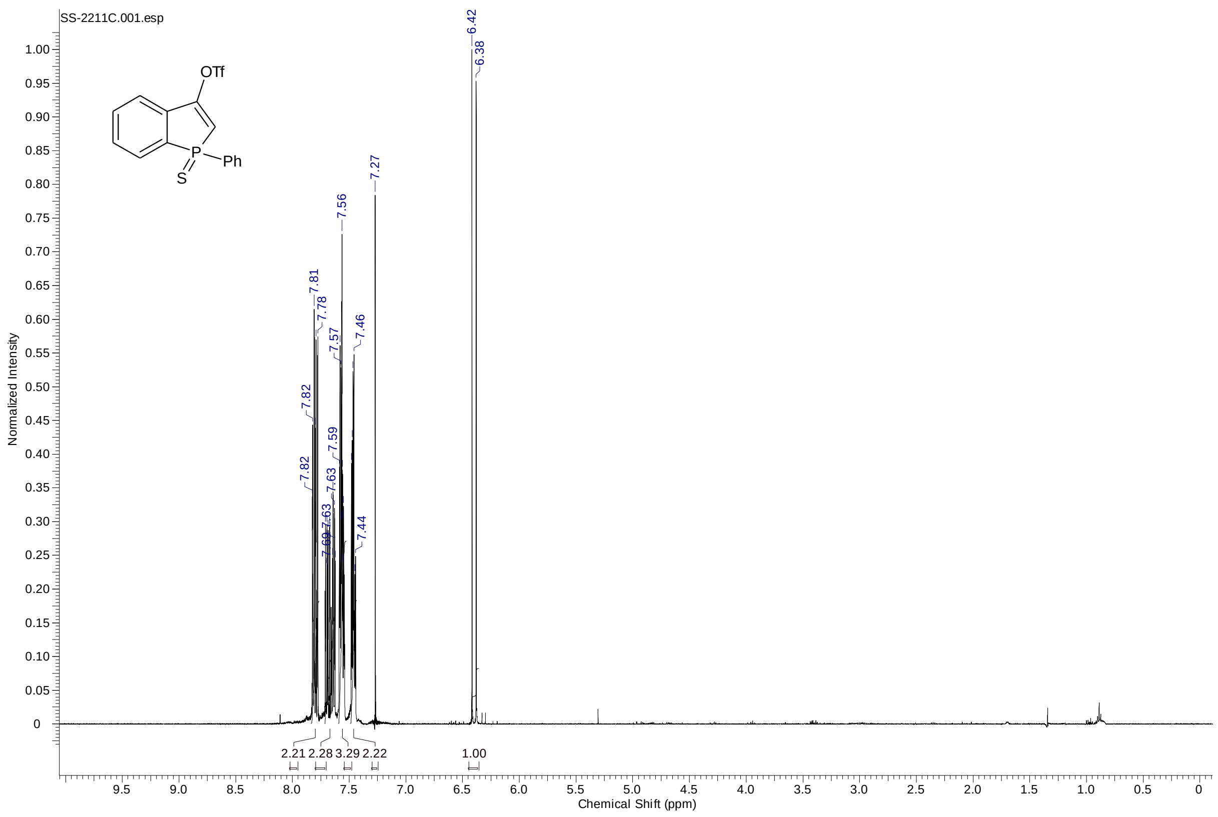

^{1}H NMR spectrum of 1-sulfido-1-phenyl-1H-phosphindol-3-yl trifluoromethanesulfonate (**6a**) (500 MHz, $CDCl_3$)

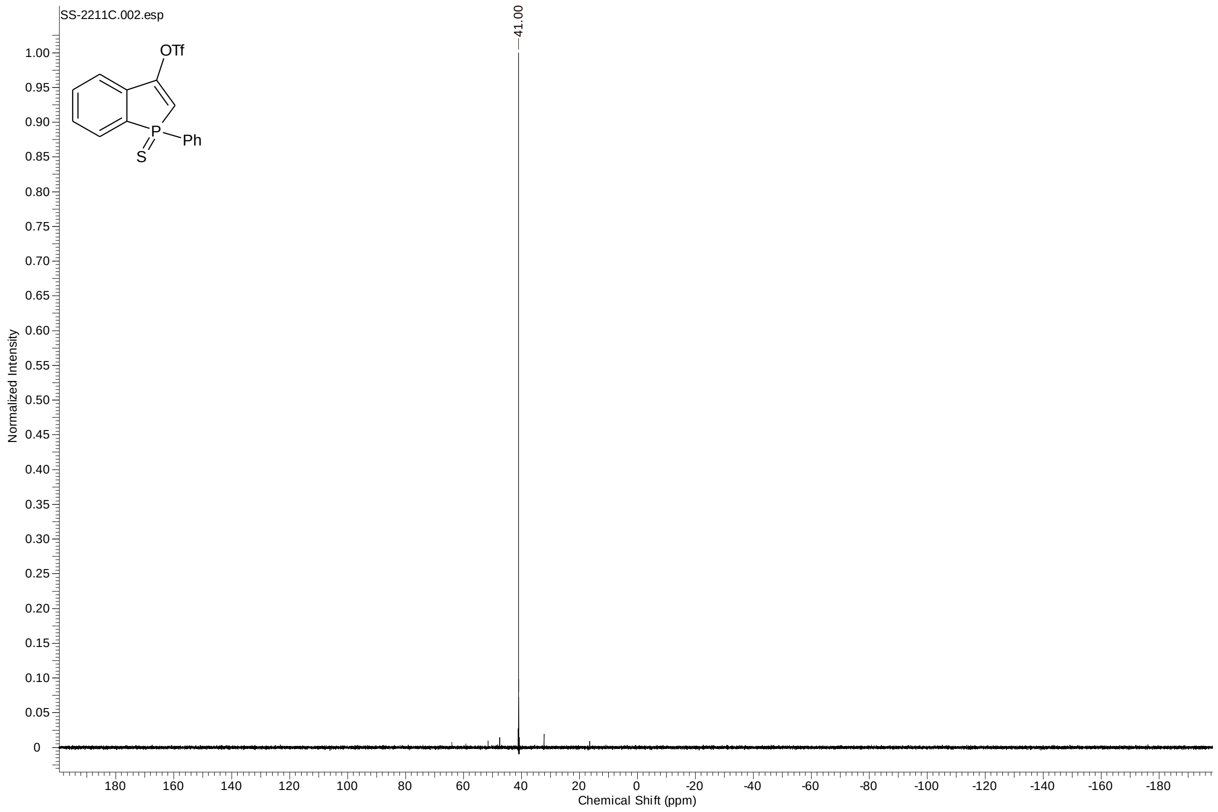

^{31}P NMR spectrum of 1-sulfido-1-phenyl-1H-phosphindol-3-yl trifluoromethanesulfonate (**6a**) (202 MHz, $CDCl_3$)

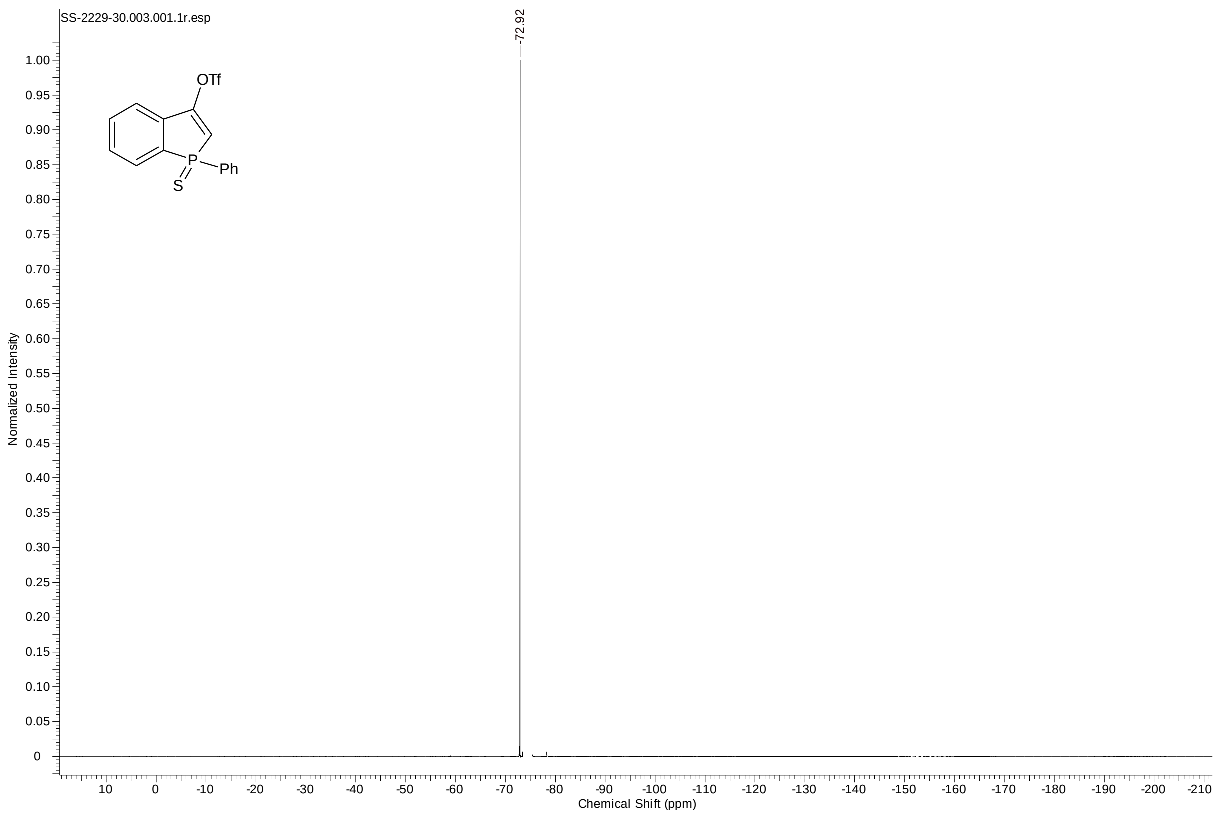

^{19}F NMR spectrum of 1-sulfido-1-phenyl-1H-phosphindol-3-yl trifluoromethanesulfonate (**6a**) (470 MHz, $CDCl_3$)

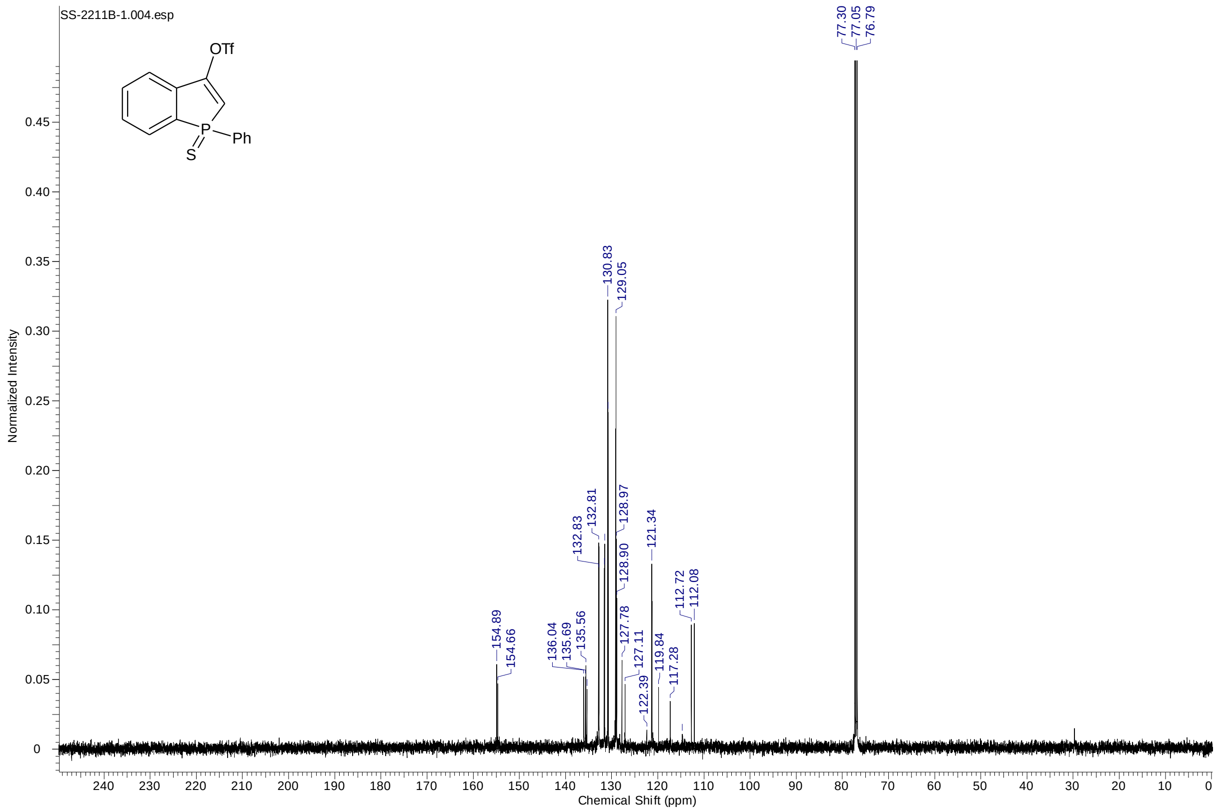

$^{13}C\{^1H\}$ NMR spectrum of 1-sulfido-1-phenyl-1H-phosphindol-3-yl trifluoromethanesulfonate (**6a**) (125 MHz, $CDCl_3$)

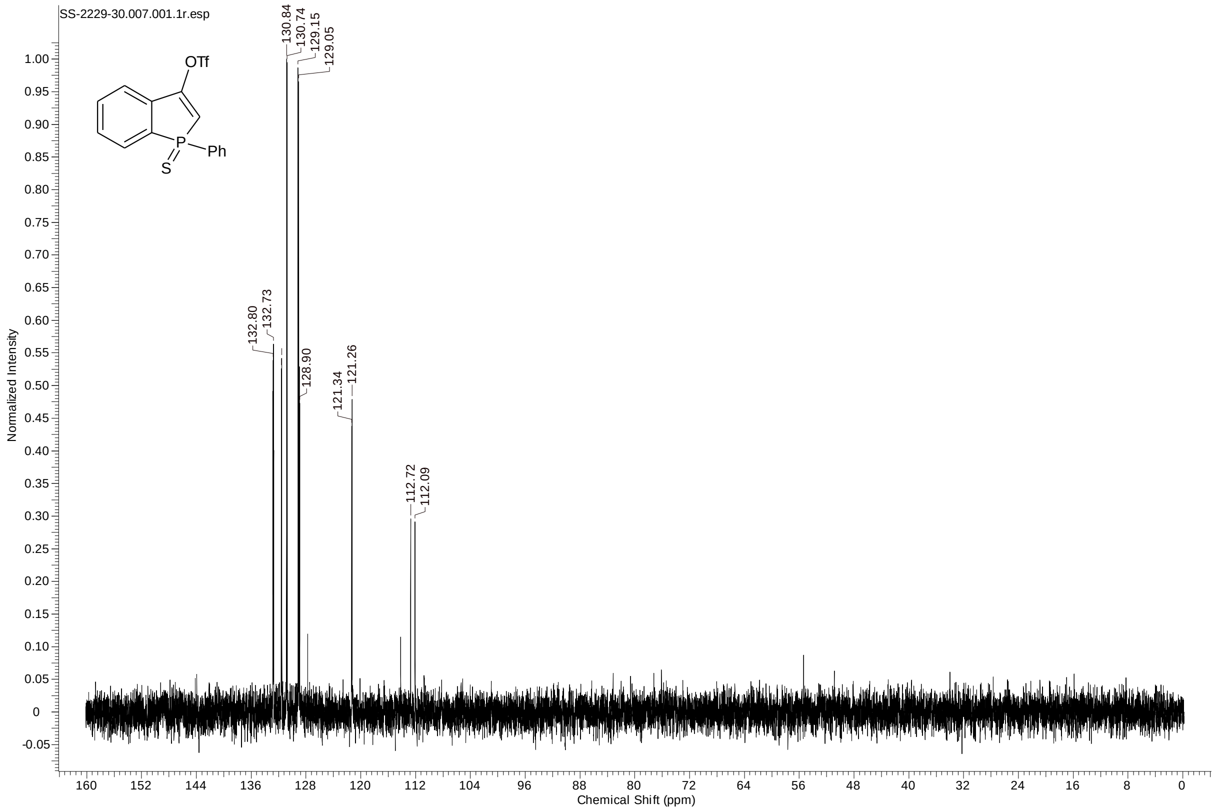

DEPT 135 NMR spectrum of 1-sulfido-1-phenyl-1H-phosphindol-3-yl trifluoromethanesulfonate (**6a**) (125 MHz, $CDCl_3$)

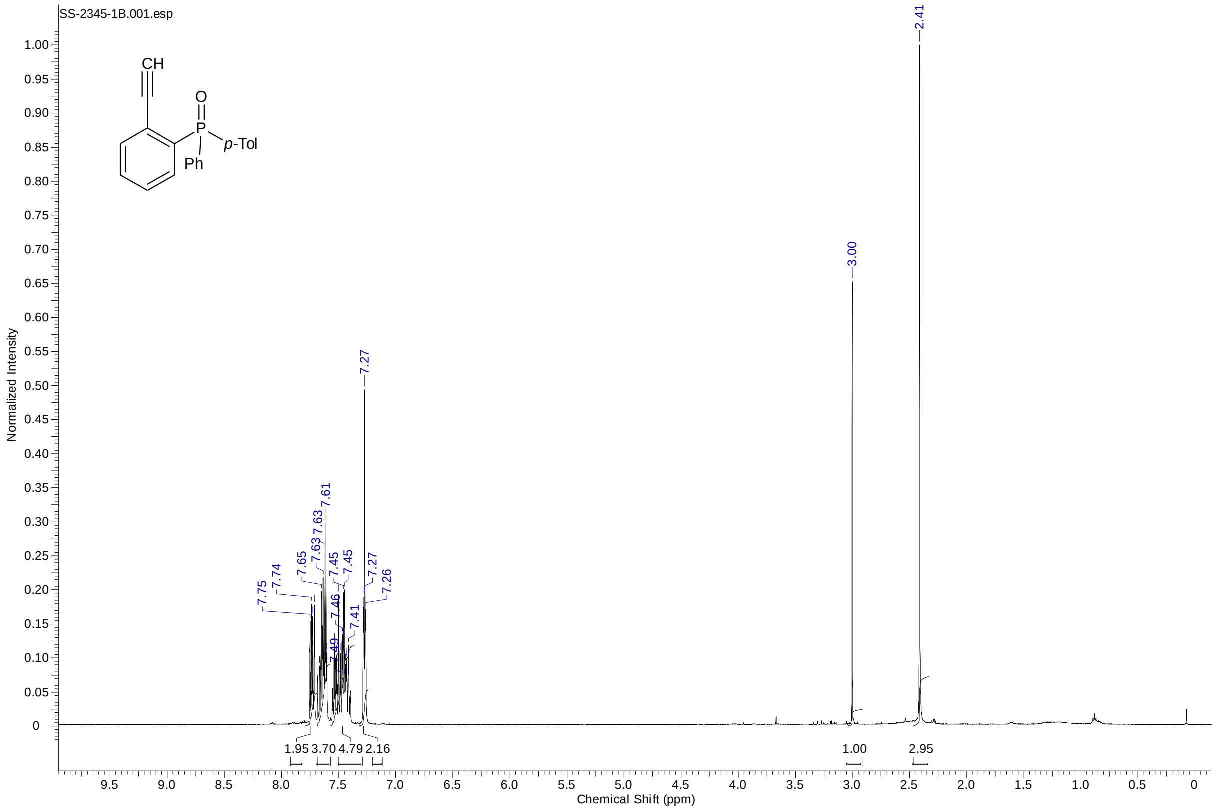

^{1}H NMR spectrum of (2-ethynylphenyl)phenyl(*p*-tolyl)phosphine oxide (**8a**) (500 MHz, $CDCl_3$)

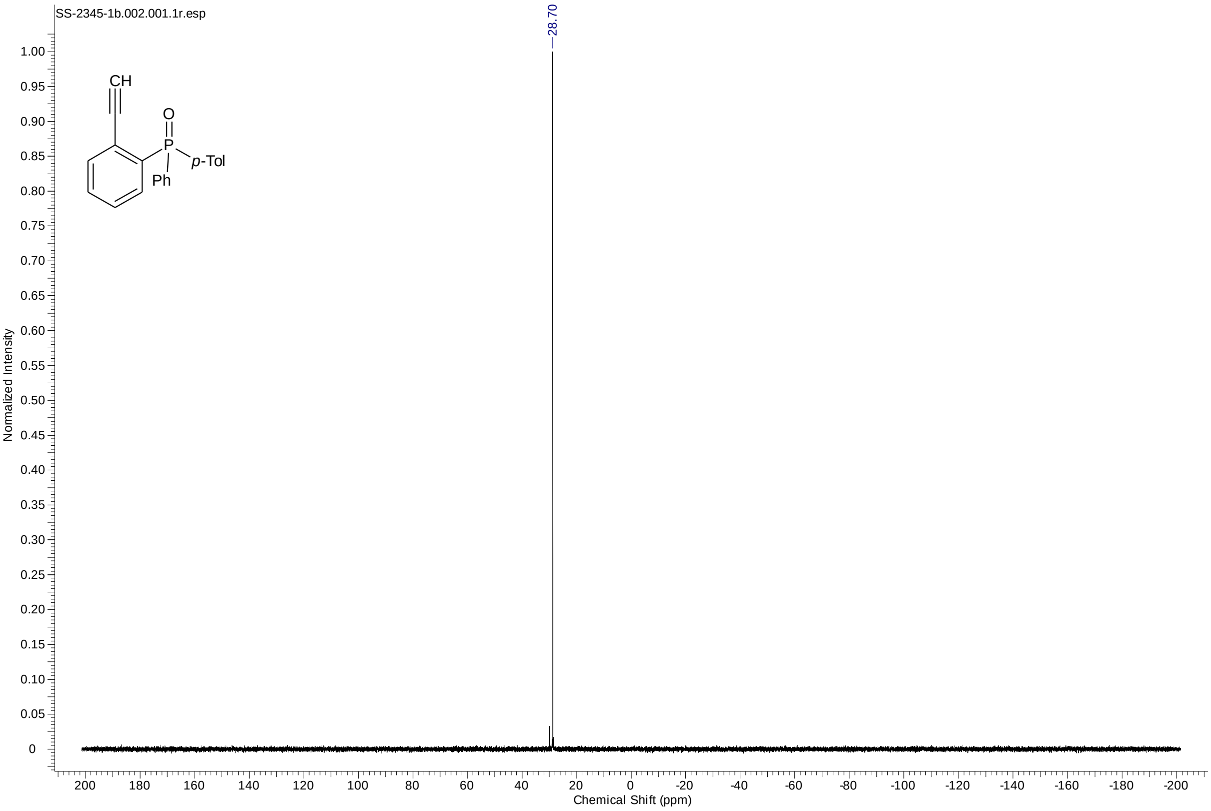

^{31}P NMR spectrum of (2-ethynylphenyl)phenyl(*p*-tolyl)phosphine oxide (**8a**) (202 MHz, $CDCl_3$)

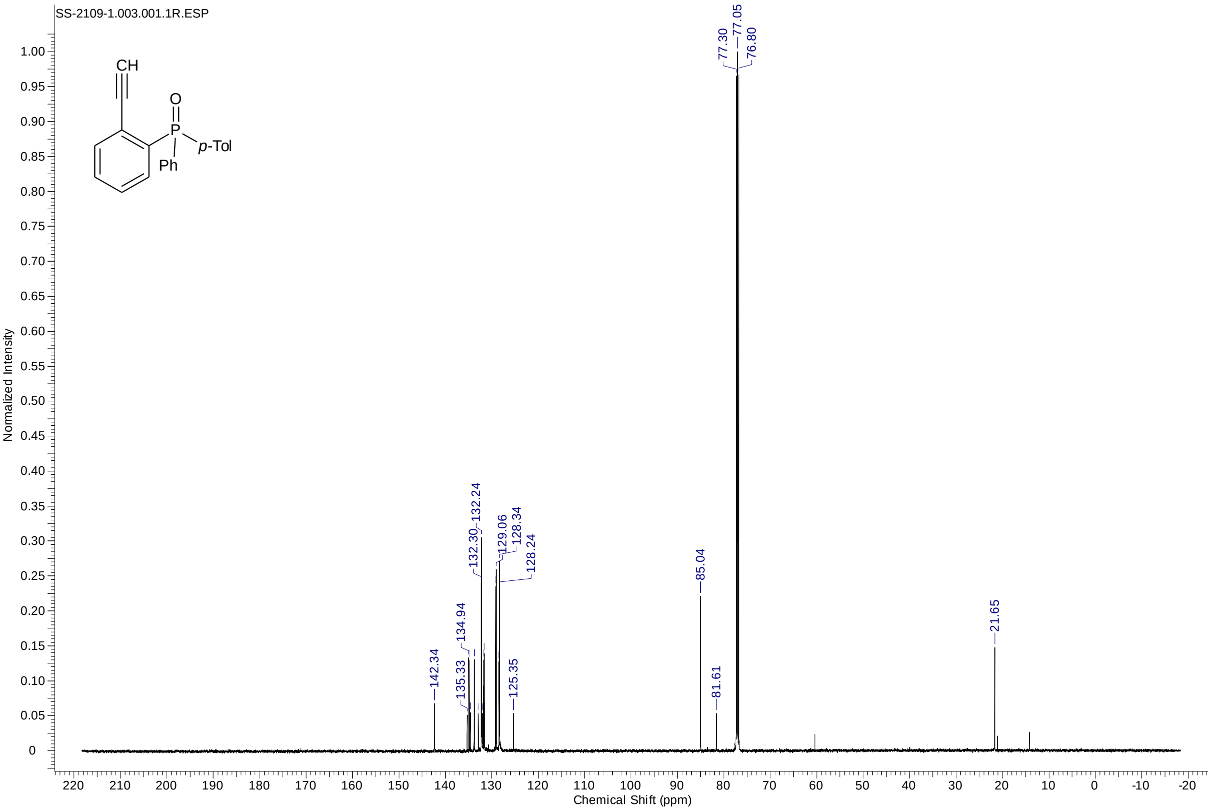

^{13}C{^{1}H} NMR spectrum of (2-ethynylphenyl)phenyl(*p*-tolyl)phosphine oxide (**8a**) (125 MHz, $CDCl_3$)

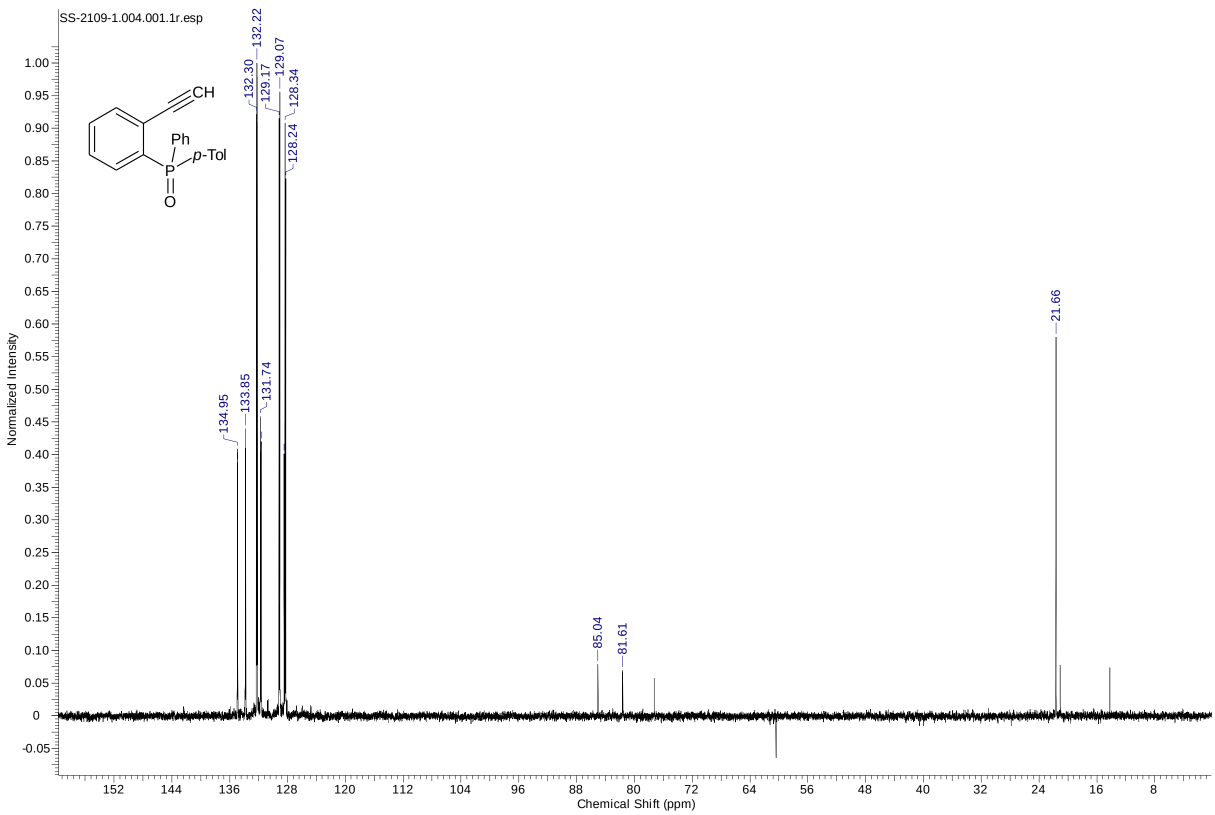

DEPT 135 NMR spectrum of (2-ethynylphenyl)phenyl(*p*-tolyl)phosphine oxide (**8a**) (125 MHz, $CDCl_3$)

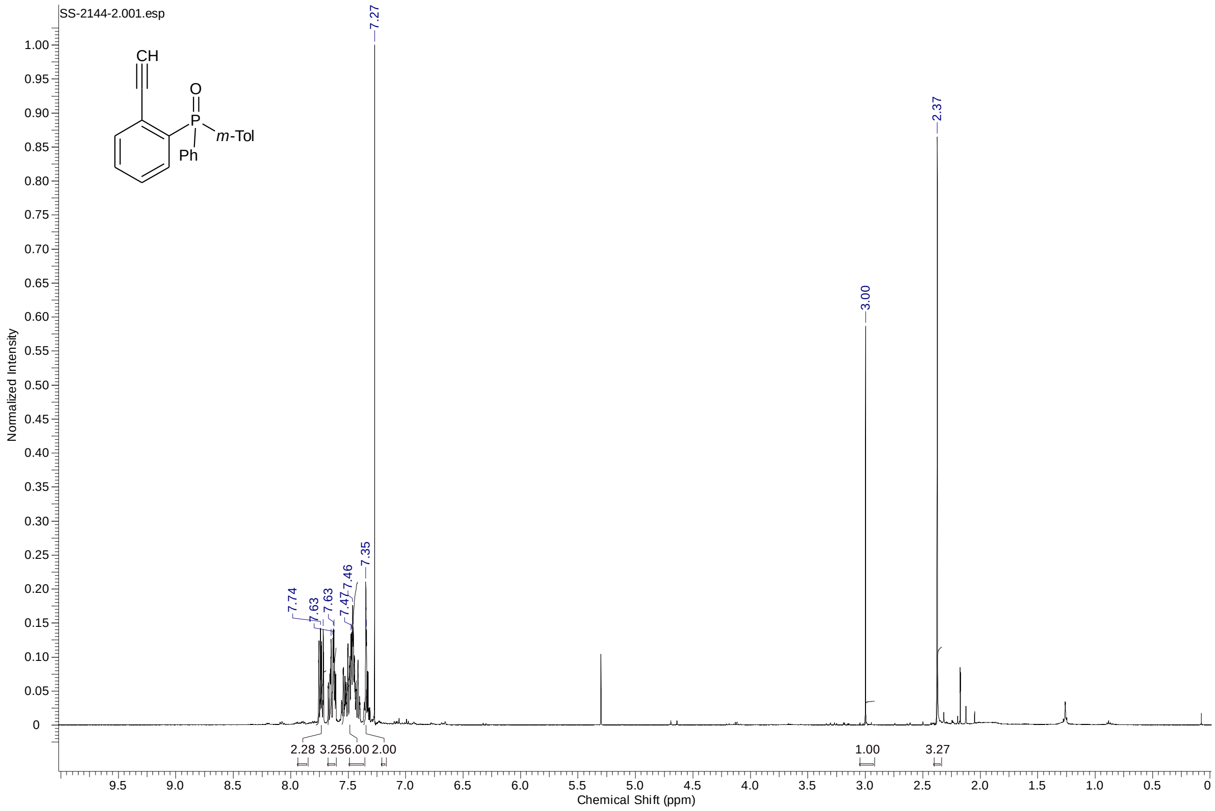

^{1}H NMR spectrum of (2-ethynylphenyl)phenyl(*m*-tolyl)phosphine oxide (**8b**) (500 MHz, $CDCl_3$)

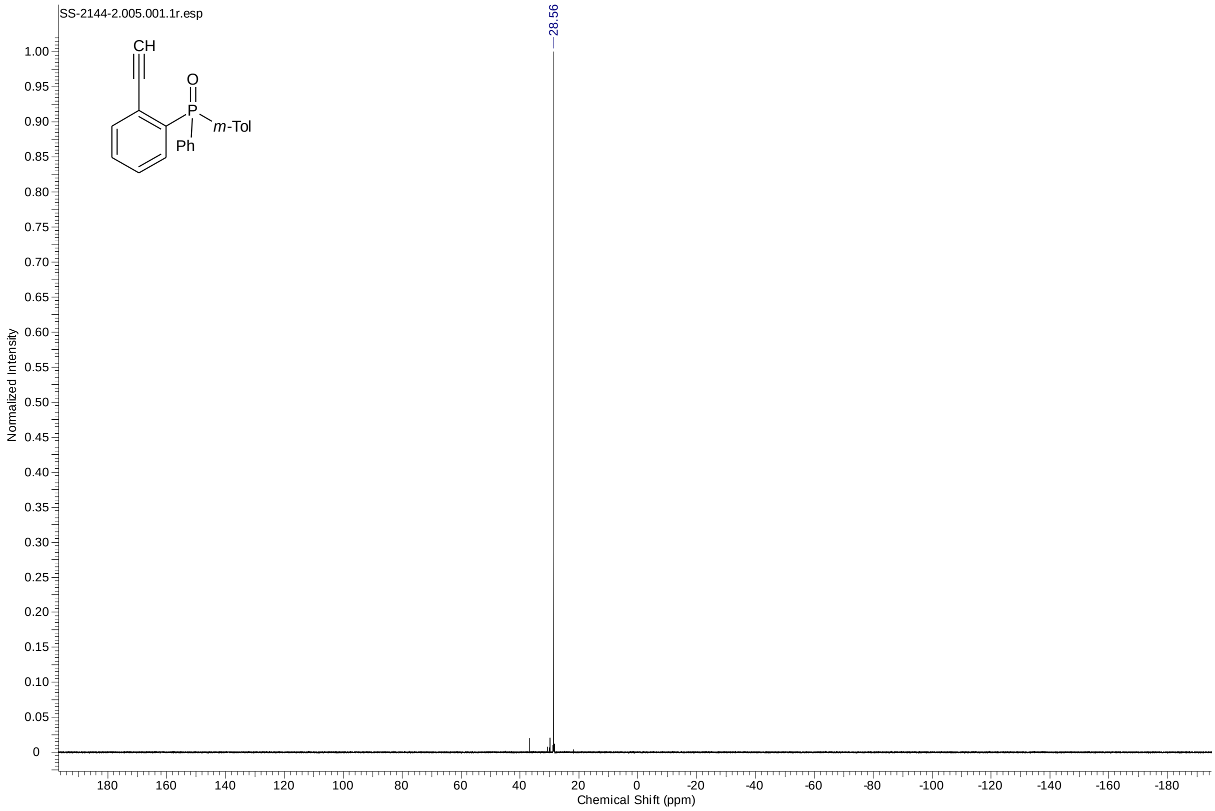

^{31}P NMR spectrum of (2-ethynylphenyl)phenyl(*m*-tolyl)phosphine oxide (**8b**) (202 MHz, $CDCl_3$)

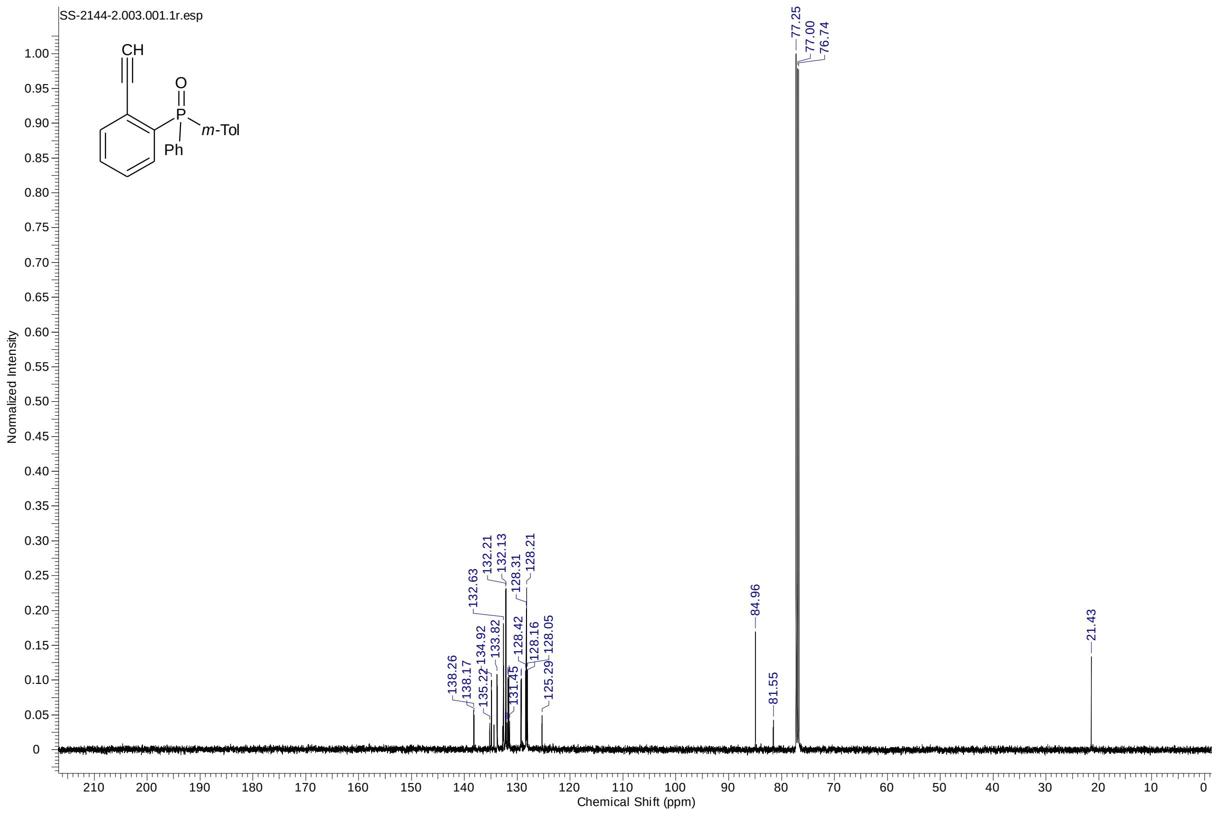

^{13}C{^{1}H} NMR spectrum of (2-ethynylphenyl)phenyl(*m*-tolyl)phosphine oxide (**8b**) (125 MHz, $CDCl_3$)

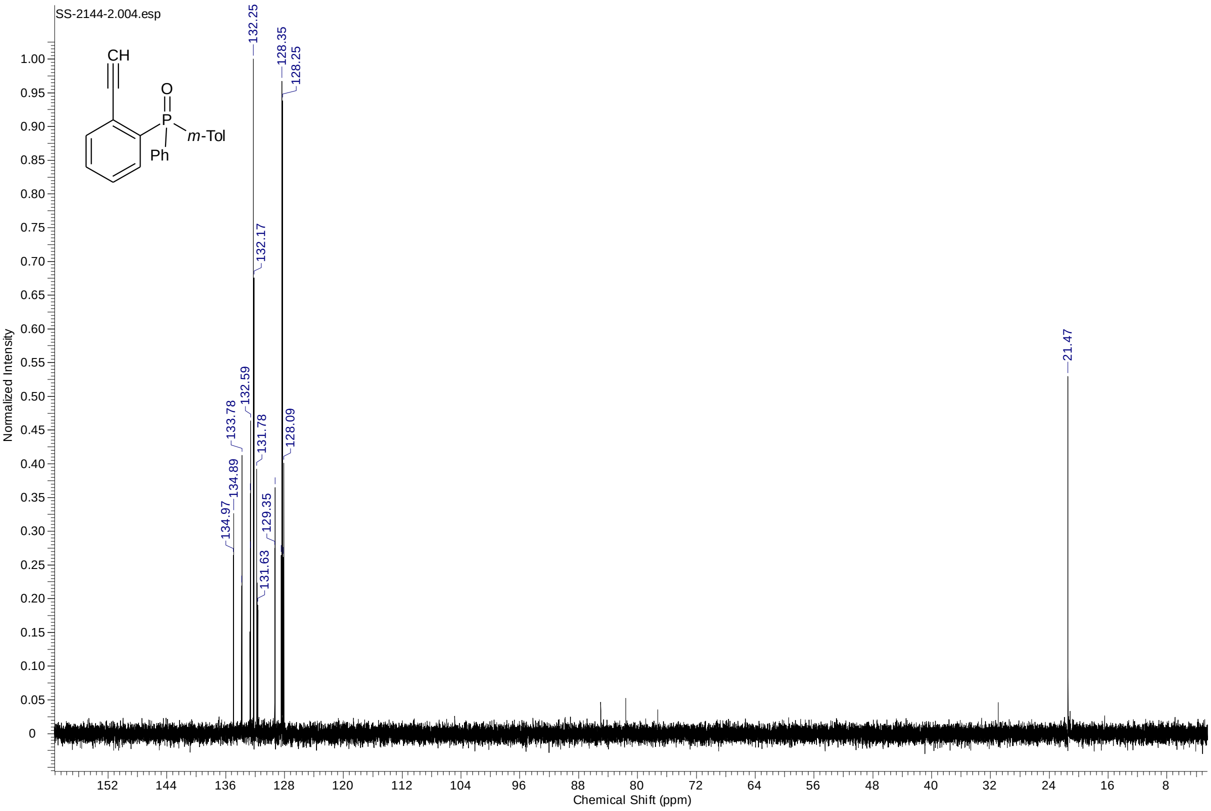

DEPT 135 NMR spectrum of (2-ethynylphenyl)phenyl(*m*-tolyl)phosphine oxide (**8b**) (125 MHz, $CDCl_3$)

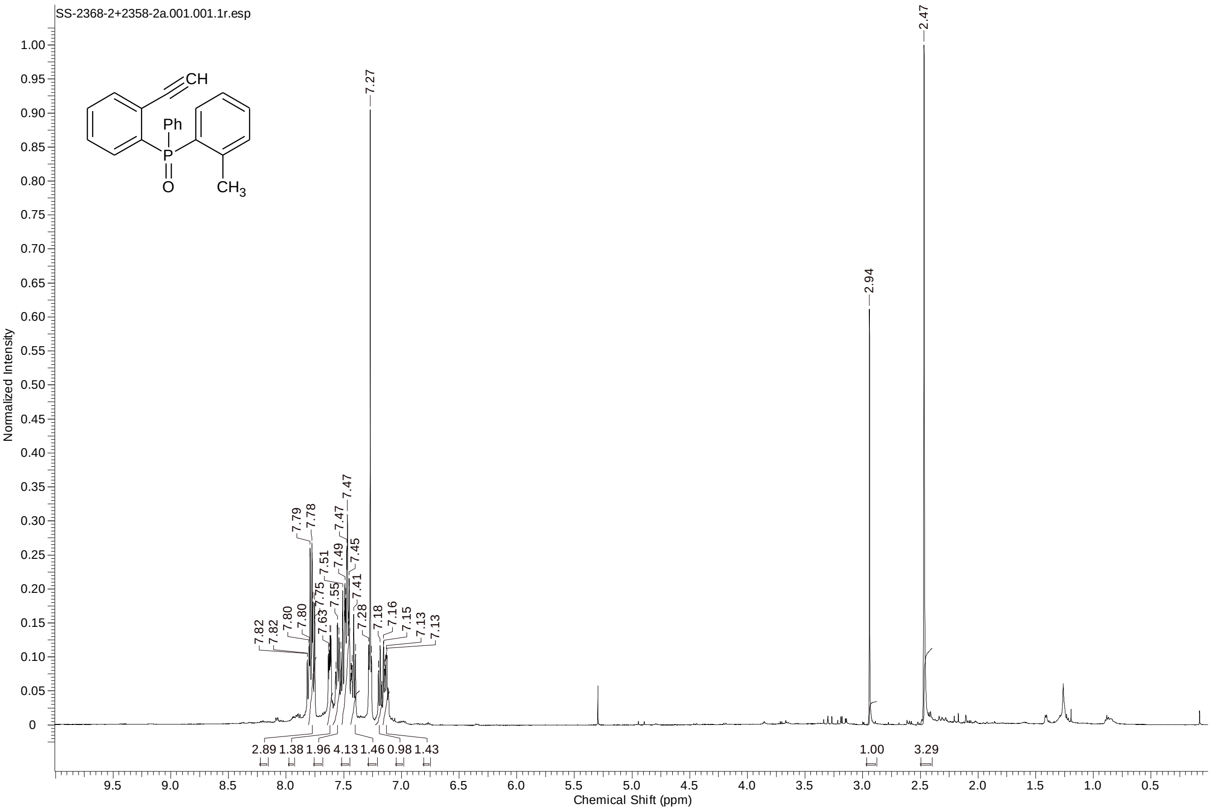

^1H NMR spectrum of (2-ethynylphenyl)phenyl(o-tolyl)phosphine oxide (**8c**) (500 MHz, $CDCl_3$)

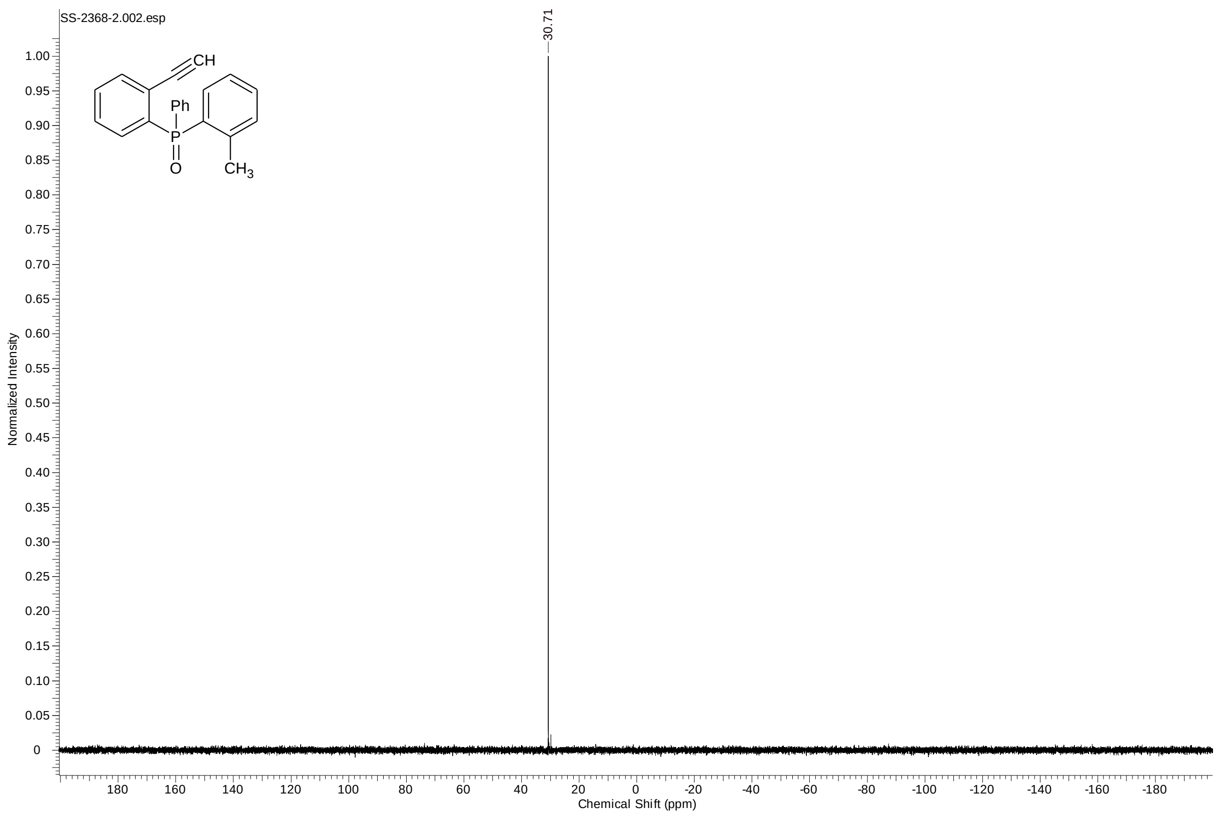

^{31}P NMR spectrum of (2-ethynylphenyl)phenyl(*o*-tolyl)phosphine oxide (**8c**) (202 MHz, $CDCl_3$)

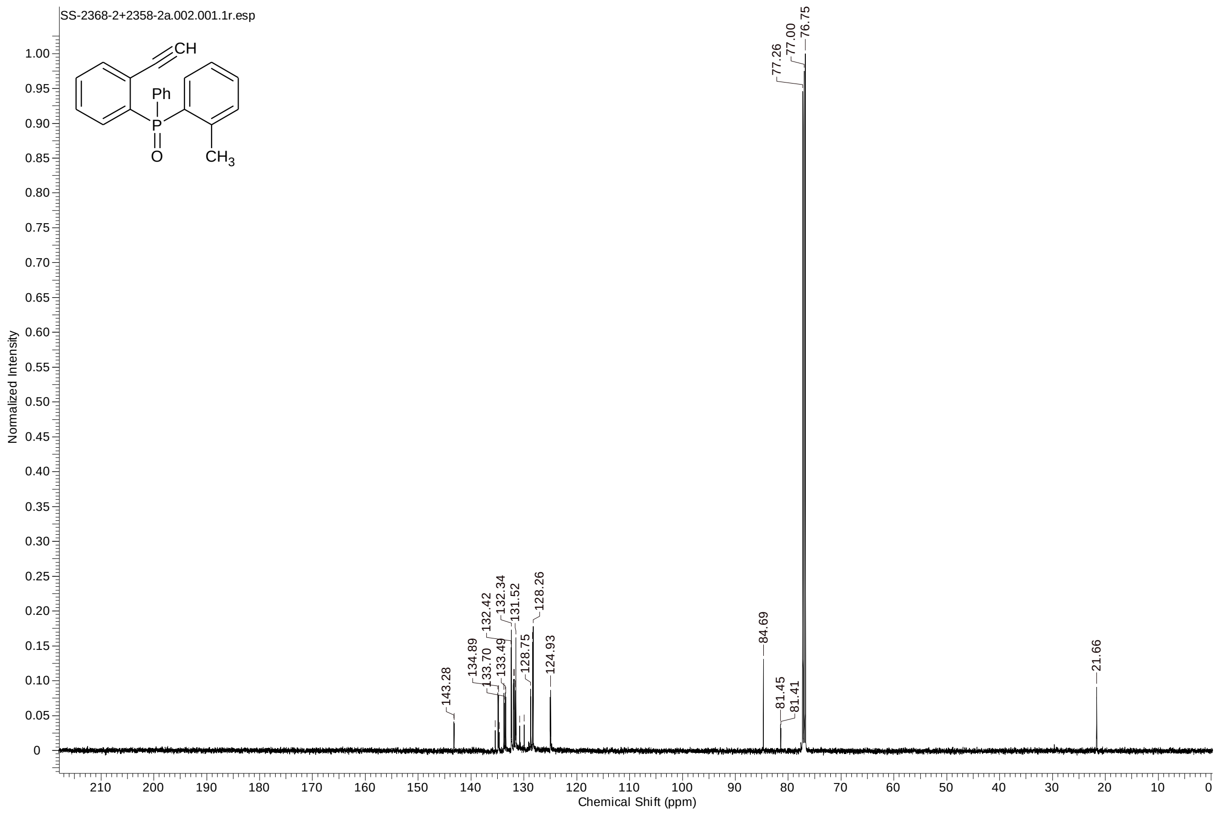

$^{13}C\{^1H\}$ NMR spectrum of (2-ethynylphenyl)phenyl(*o*-tolyl)phosphine oxide (**8c**) (125 MHz, $CDCl_3$)

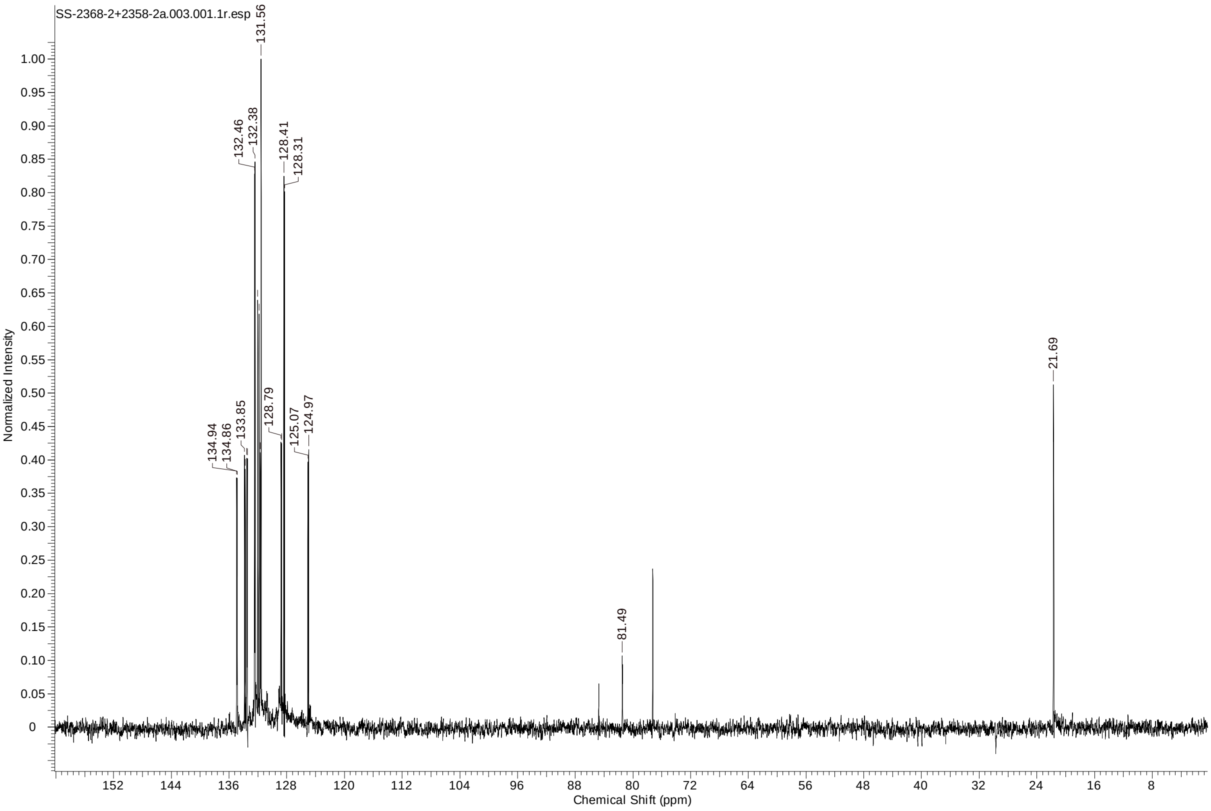

DEPT 135 NMR spectrum of (2-ethynylphenyl)phenyl(*o*-tolyl)phosphine oxide (**8c**) (125 MHz, $CDCl_3$)

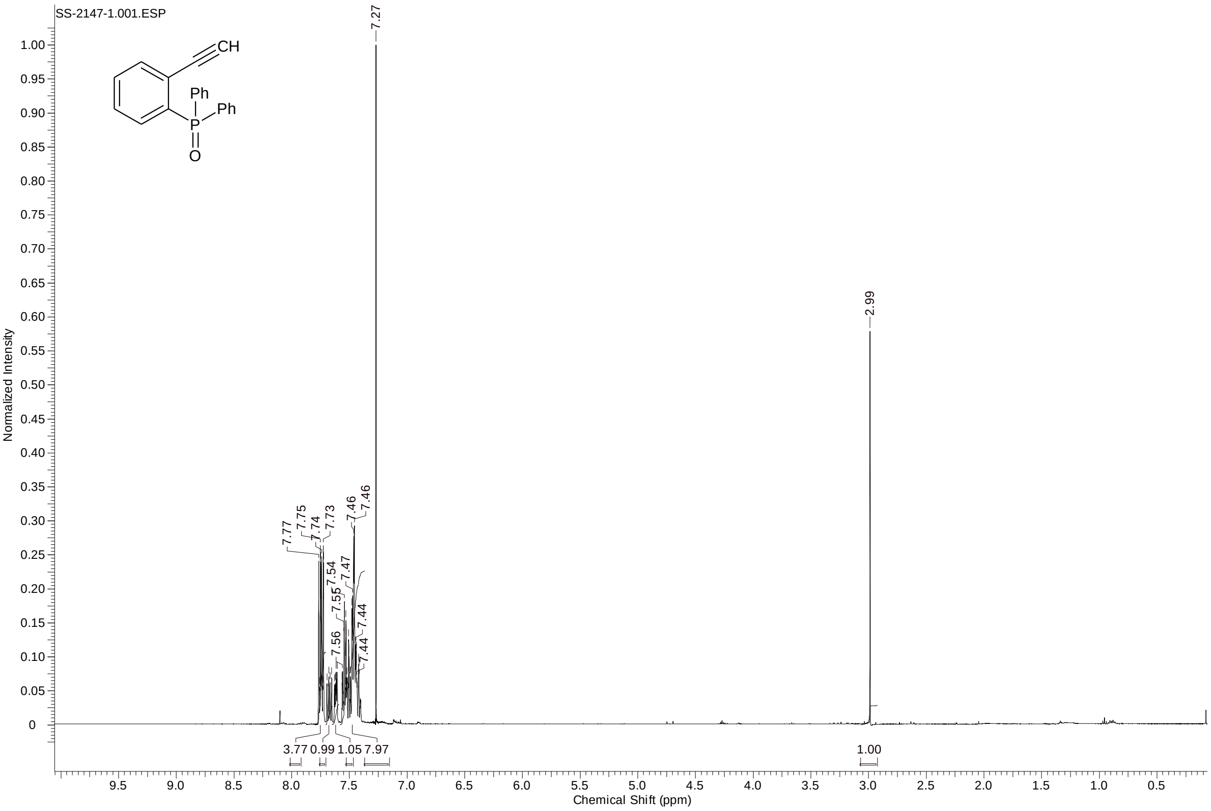

^{1}H NMR spectrum of [2-(ethynyl)phenyl]diphenylphosphine oxide (**8d**) (500 MHz, $CDCl_3$)

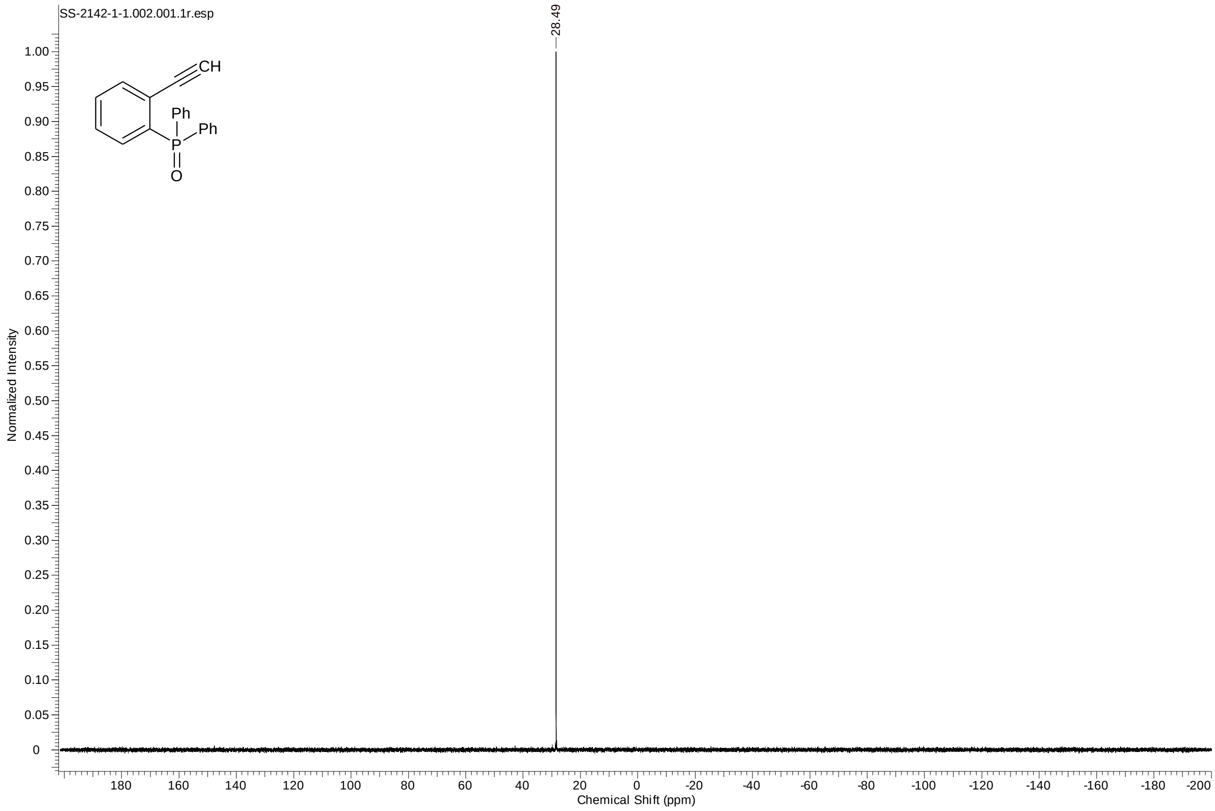

^{31}P NMR spectrum of [2-(ethynyl)phenyl]diphenylphosphine oxide (**8d**) (202 MHz, $CDCl_3$)

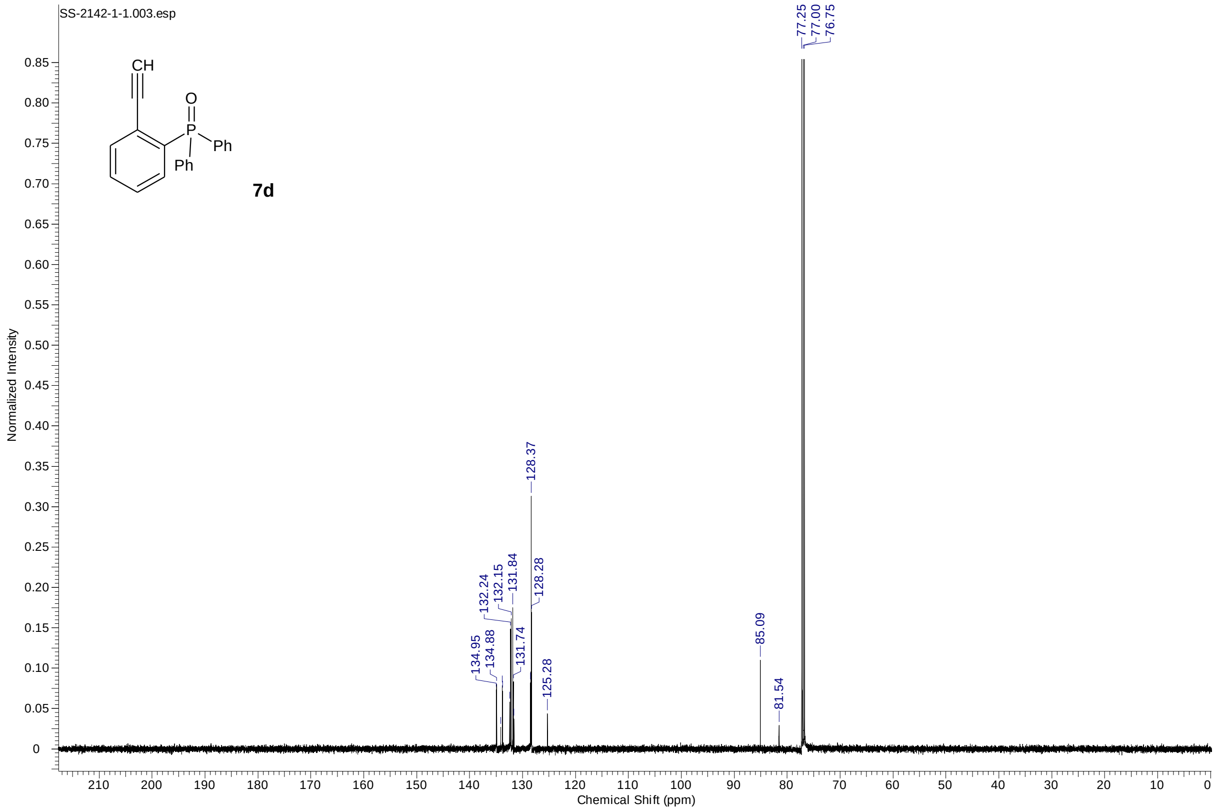

$^{13}C\{^1H\}$ NMR spectrum of [2-(ethynyl)phenyl]diphenylphosphine oxide (**8d**) (125 MHz, $CDCl_3$)

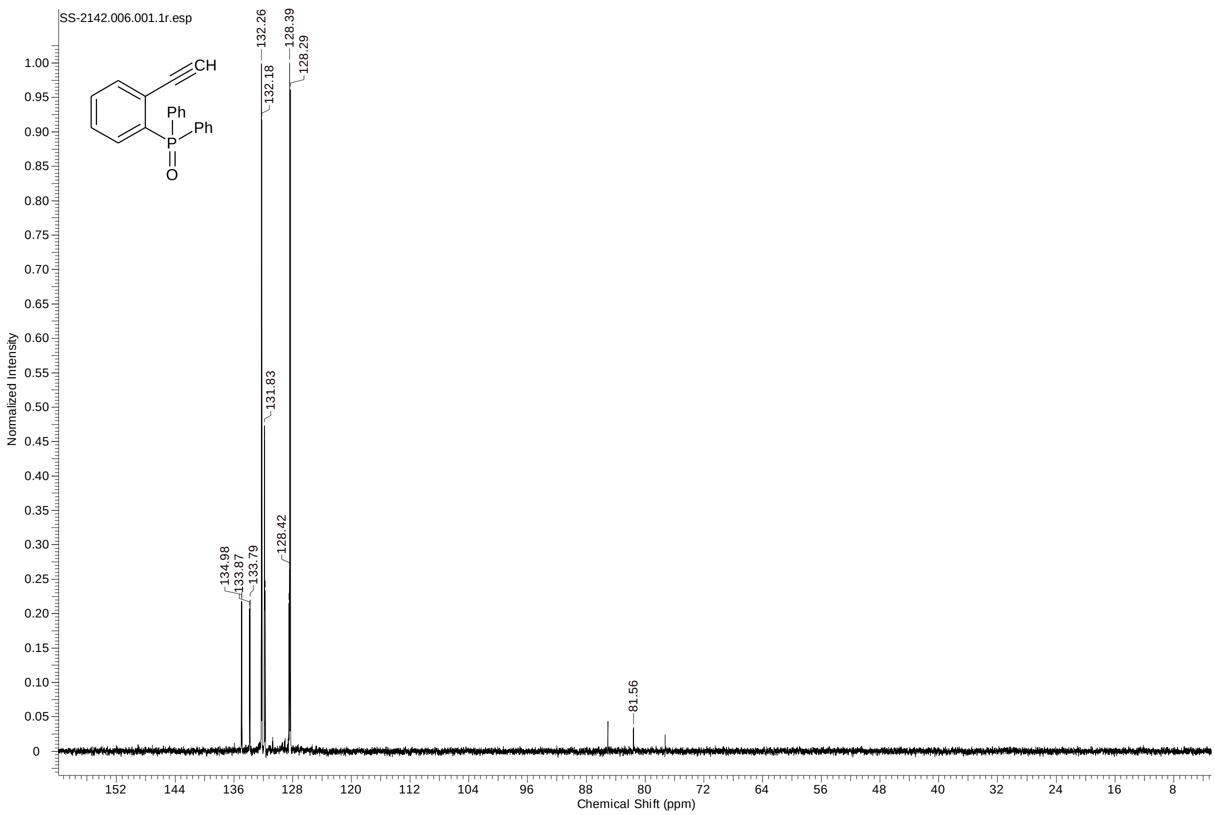

DEPT 135 NMR spectrum of [2-(ethynyl)phenyl]diphenylphosphine oxide (**8d**) (125 MHz, $CDCl_3$)

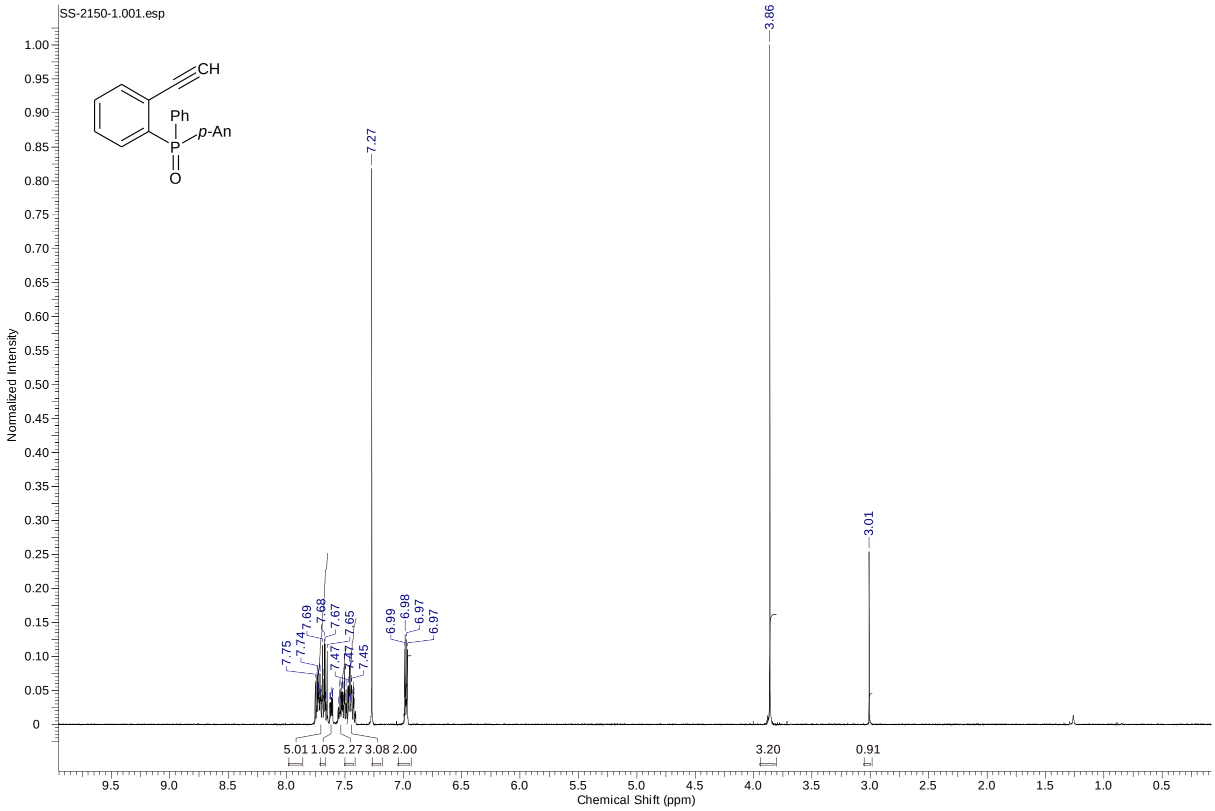

^{1}H NMR spectrum of (2-ethynylphenyl)phenyl(*p*-anisyl)phosphine oxide (**8e**) (500 MHz, $CDCl_3$)

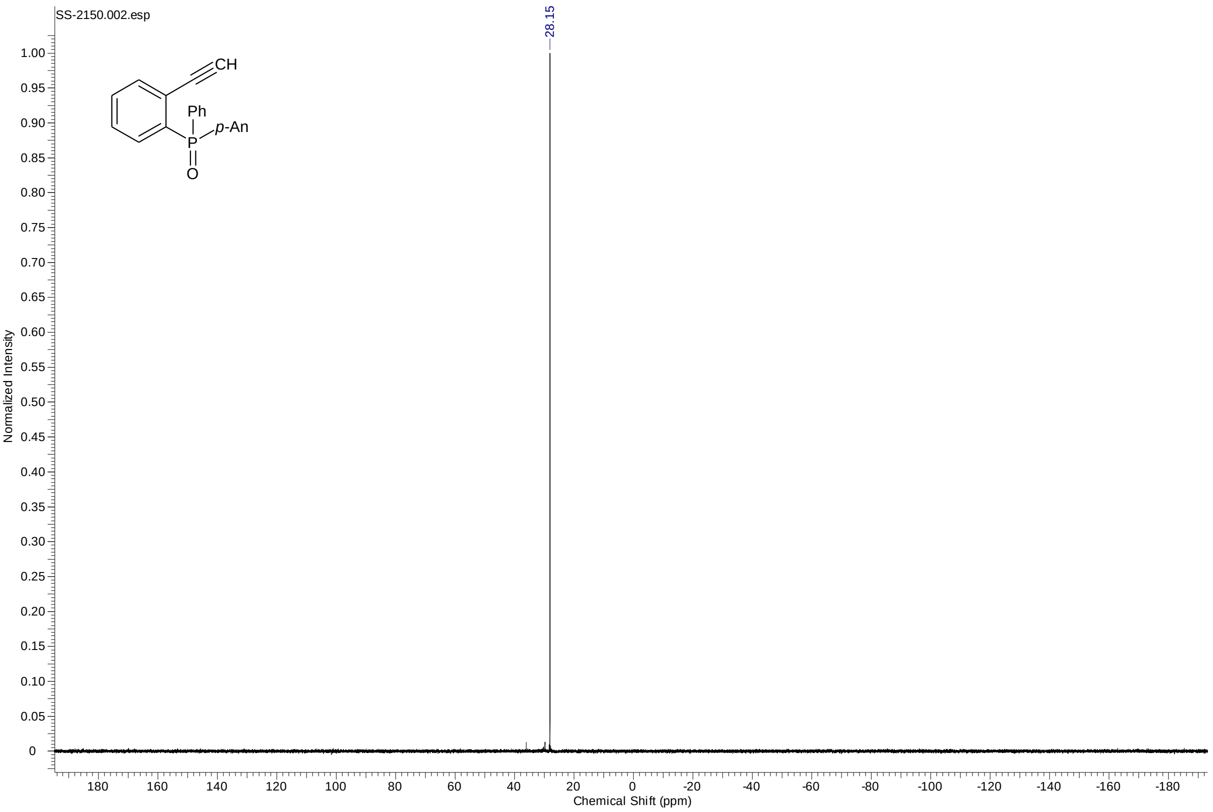

^{31}P NMR spectrum of (2-ethynylphenyl)phenyl(*p*-anisyl)phosphine oxide (**8e**) (202 MHz, $CDCl_3$)

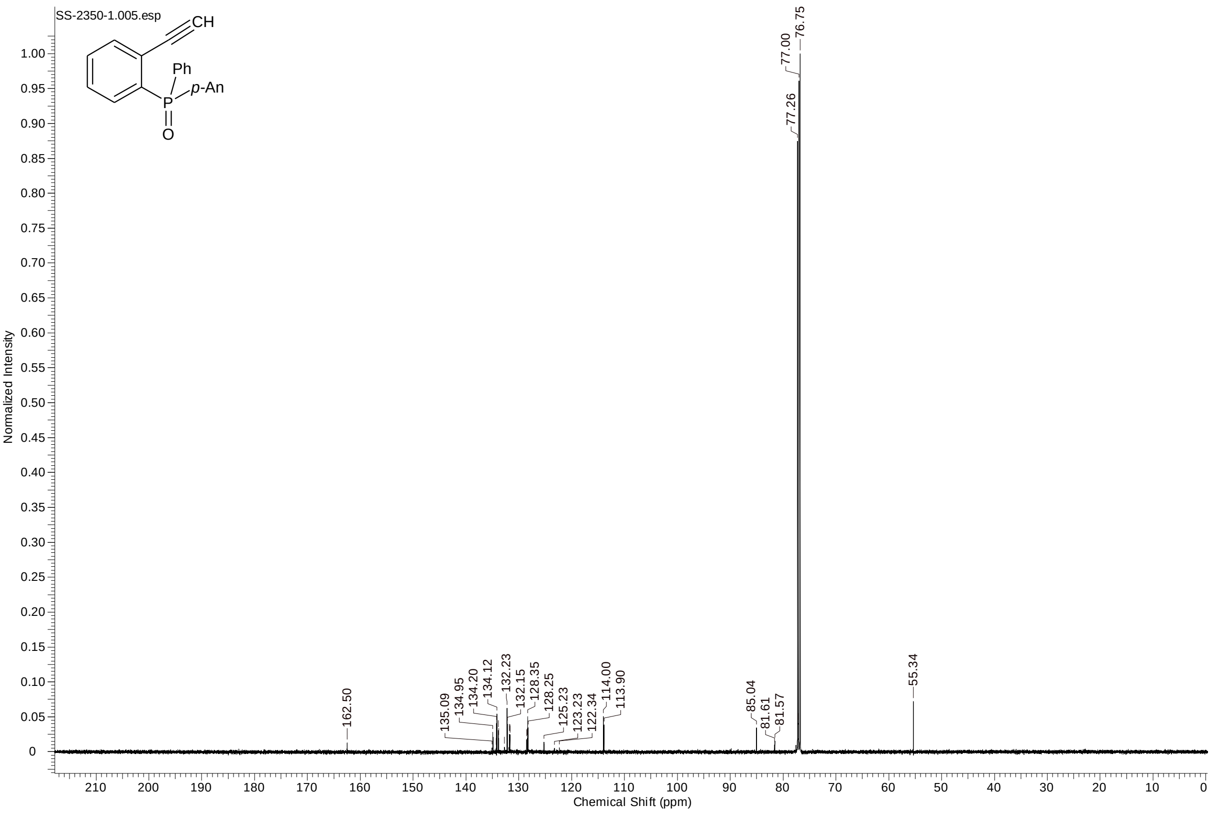

$^{13}C\{^1H\}$ NMR spectrum of (2-ethynylphenyl)phenyl(*p*-anisyl)phosphine oxide (**8e**) (125 MHz, $CDCl_3$)

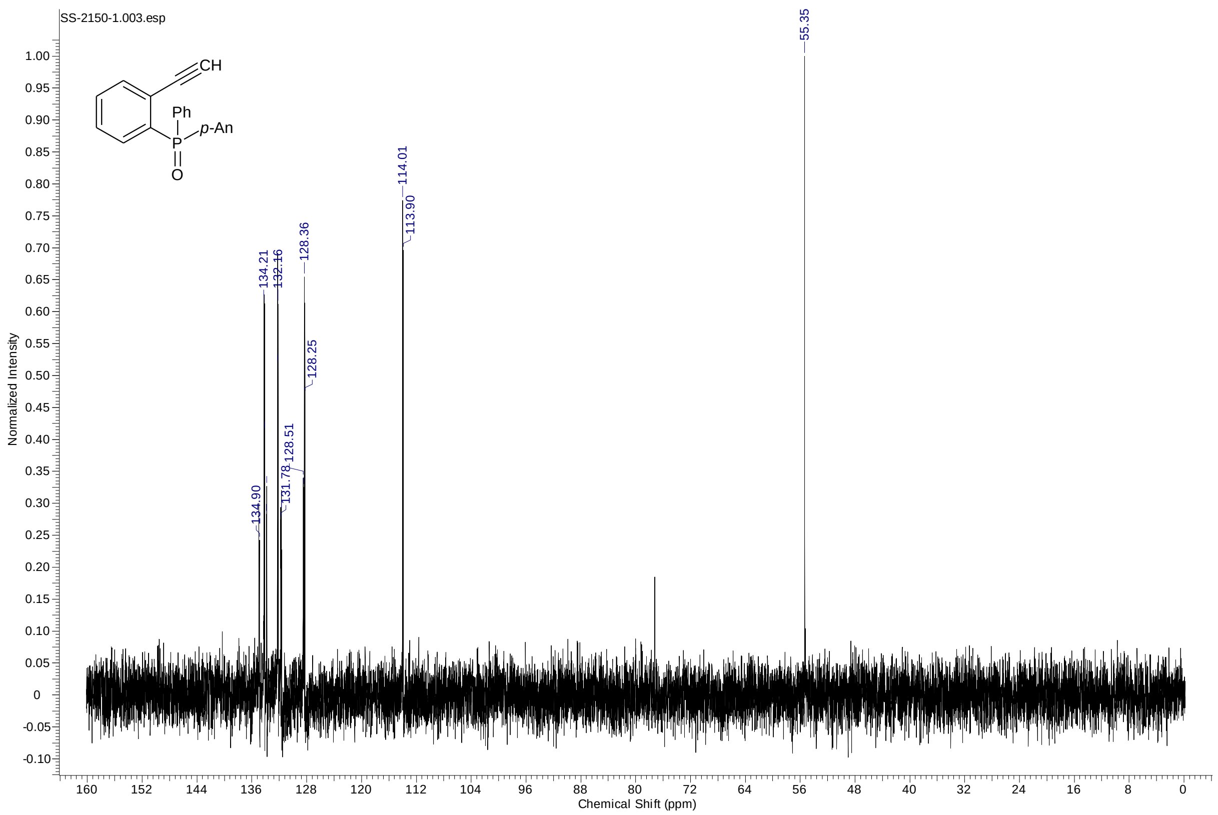

DEPT 135 NMR spectrum of (2-ethynylphenyl)phenyl(*p*-anisyl)phosphine oxide (**8e**) (125 MHz, $CDCl_3$)

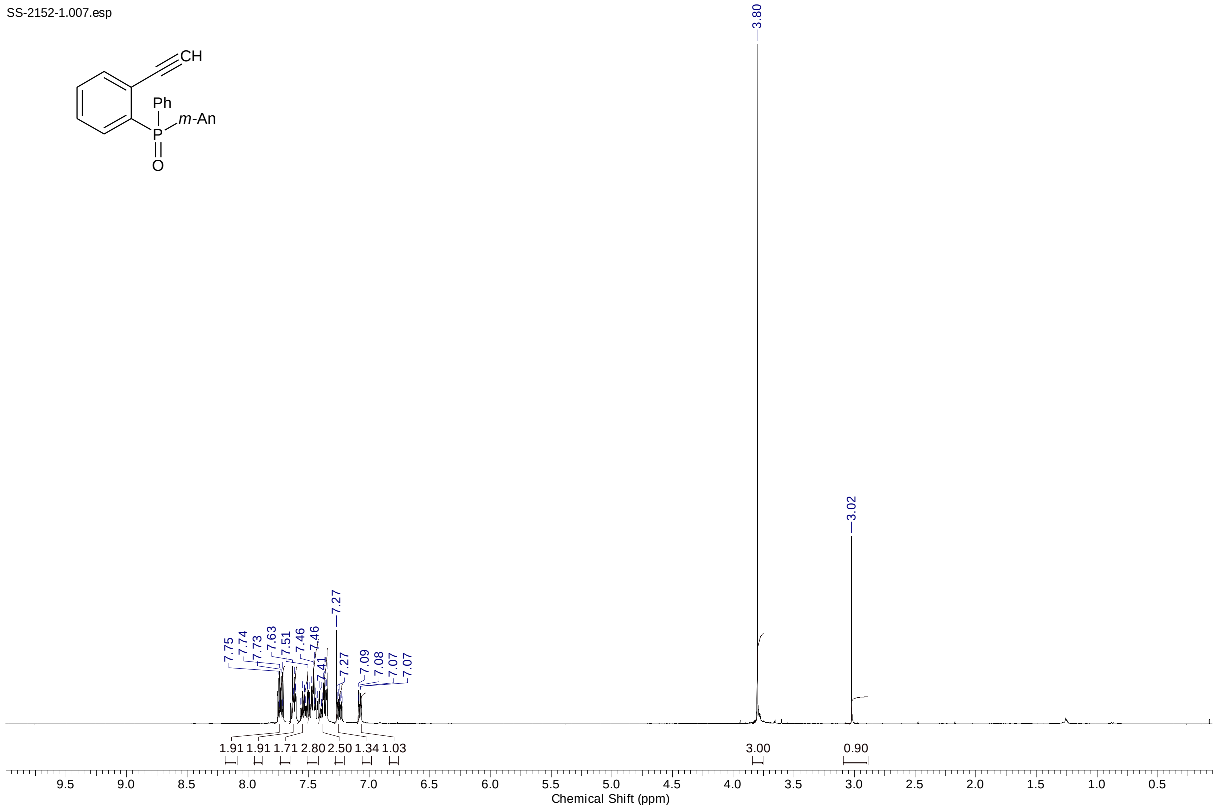

^{1}H NMR spectrum of (2-ethynylphenyl)phenyl(*m*-anisyl)phosphine oxide (**8f**) (500 MHz, $CDCl_3$)

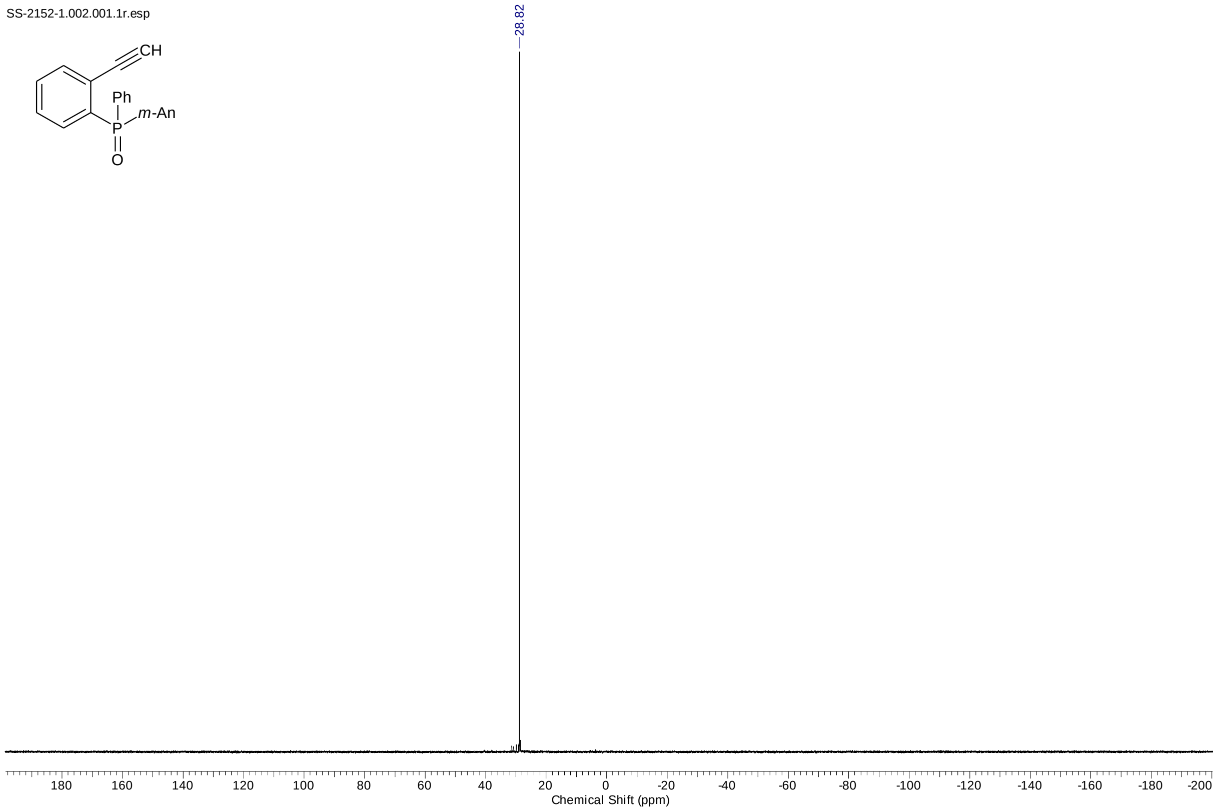

^{31}P NMR spectrum of (2-ethynylphenyl)phenyl(*m*-anisyl)phosphine oxide (**8f**) (202 MHz, $CDCl_3$)

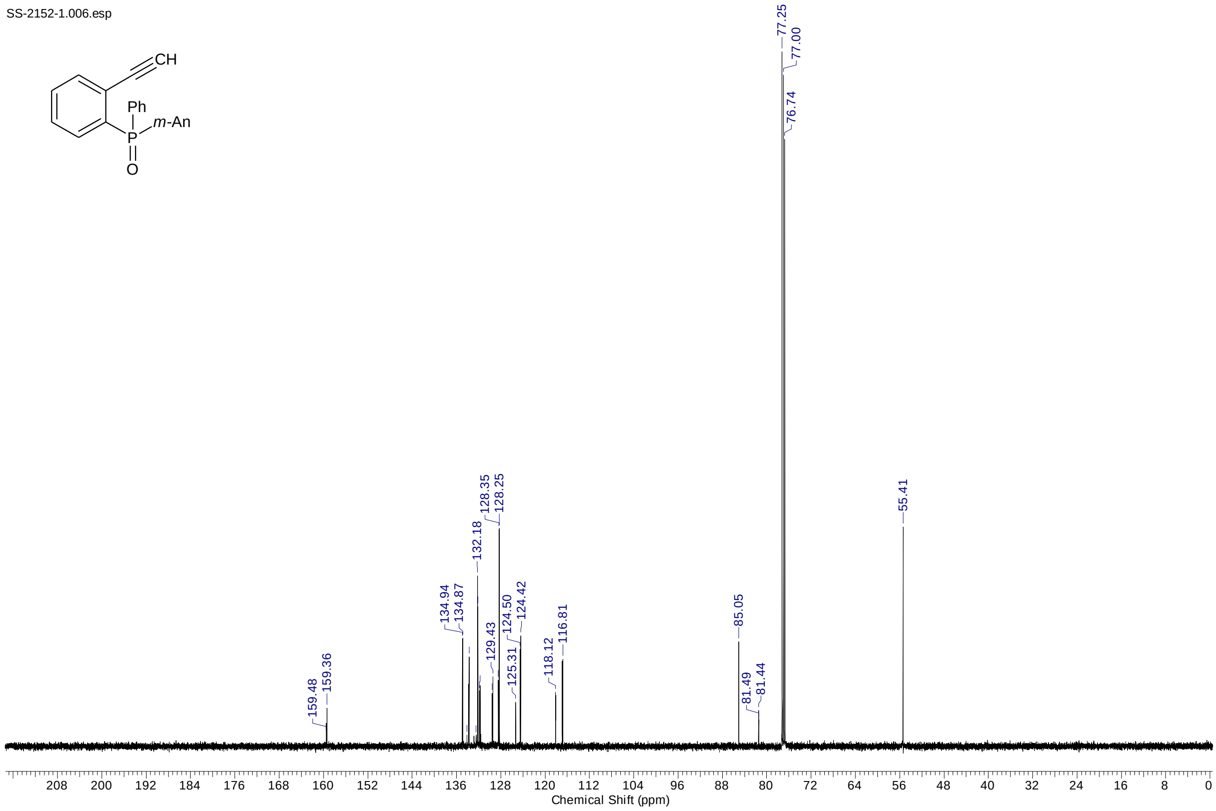

$^{13}C\{^1H\}$ NMR spectrum of (2-ethynylphenyl)phenyl(*m*-anisyl)phosphine oxide (**8f**) (125 MHz, $CDCl_3$)

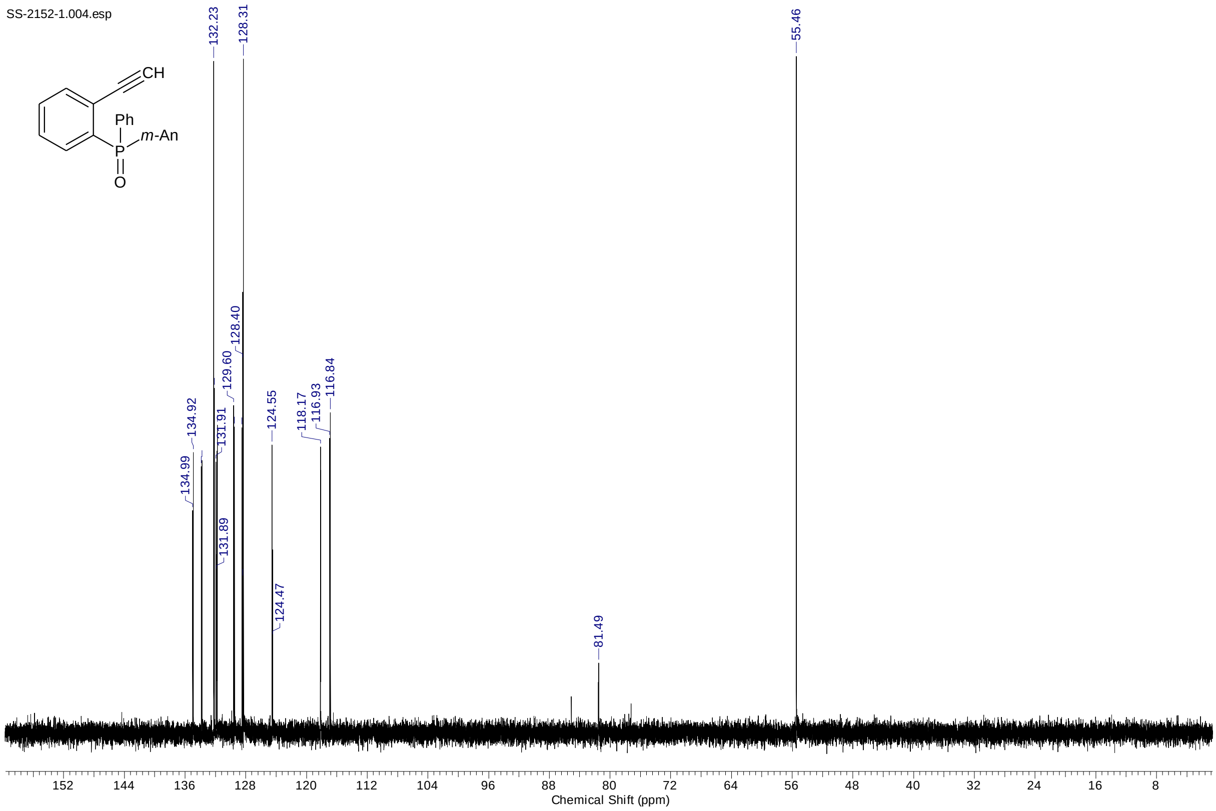

DEPT 135 NMR spectrum of (2-ethynylphenyl)phenyl(*m*-anisyl)phosphine oxide (**8f**) (125 MHz, $CDCl_3$)

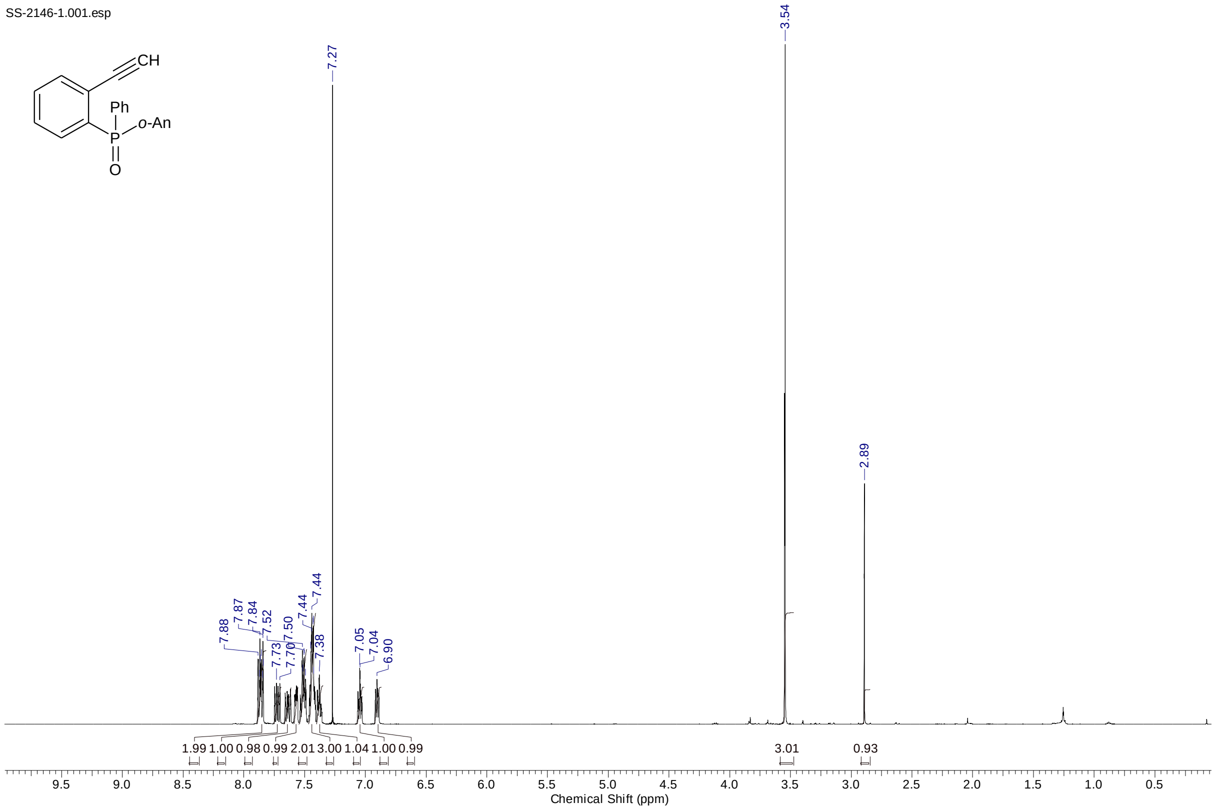

^{1}H NMR spectrum of (2-ethynylphenyl)phenyl(*o*-anisyl)phosphine oxide (**8g**) (500 MHz, $CDCl_3$)

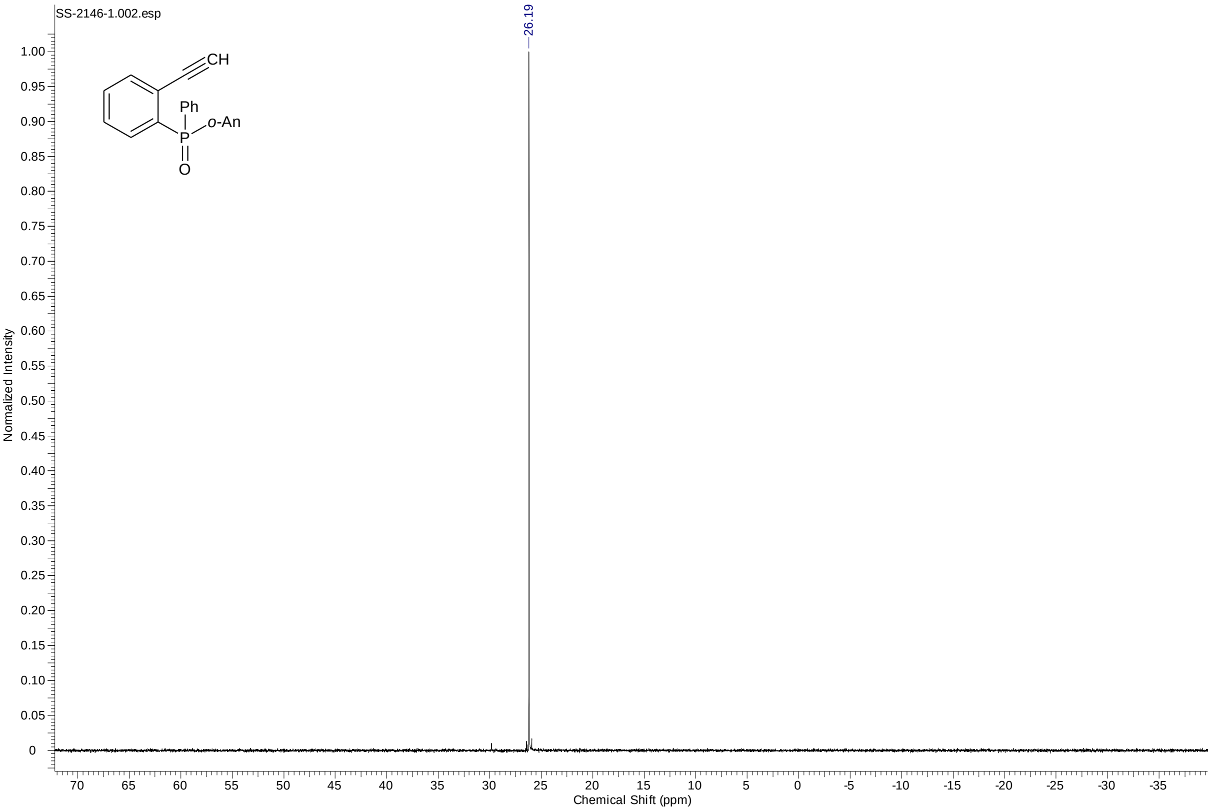

^{31}P NMR spectrum of (2-ethynylphenyl)phenyl(*o*-anisyl)phosphine oxide (**8g**) (202 MHz, $CDCl_3$)

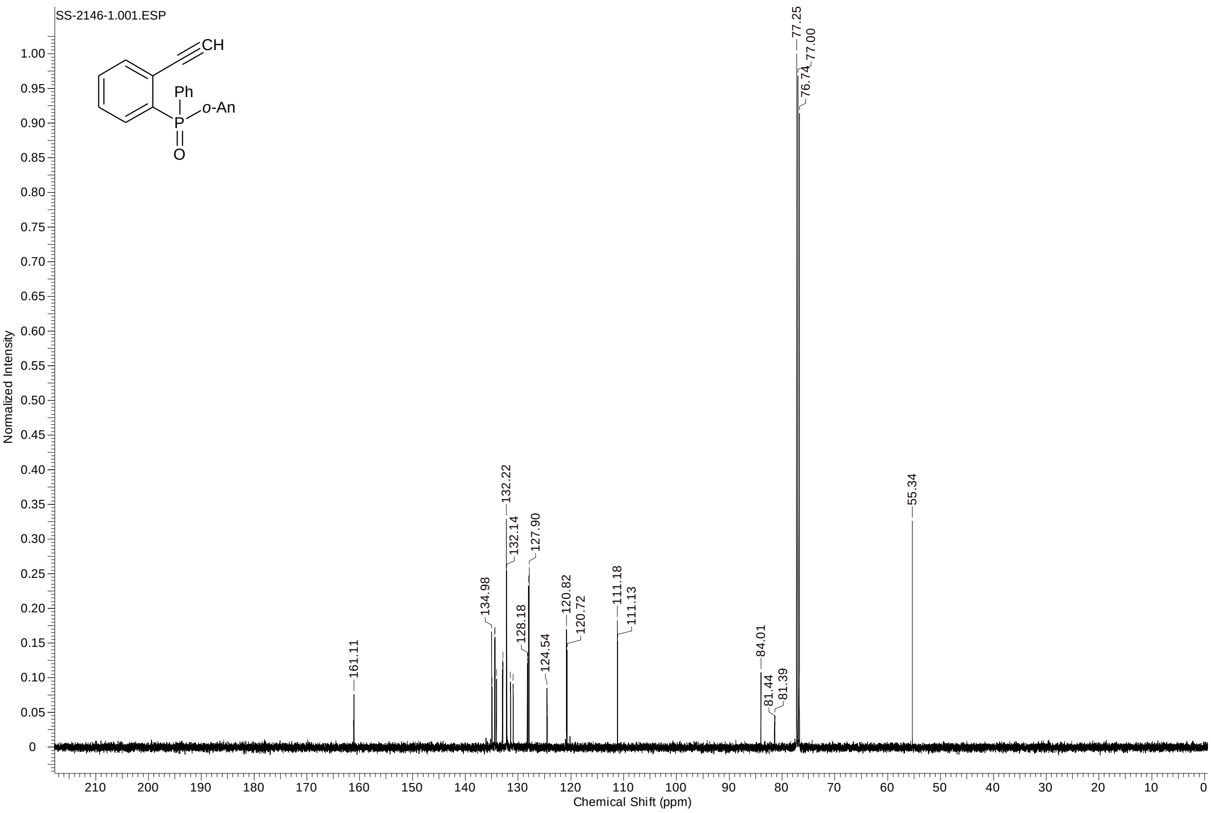

^{13}C{^{1}H} NMR spectrum of (2-ethynylphenyl)phenyl(*o*-anisyl)phosphine oxide (**8g**) (125 MHz, $CDCl_3$)

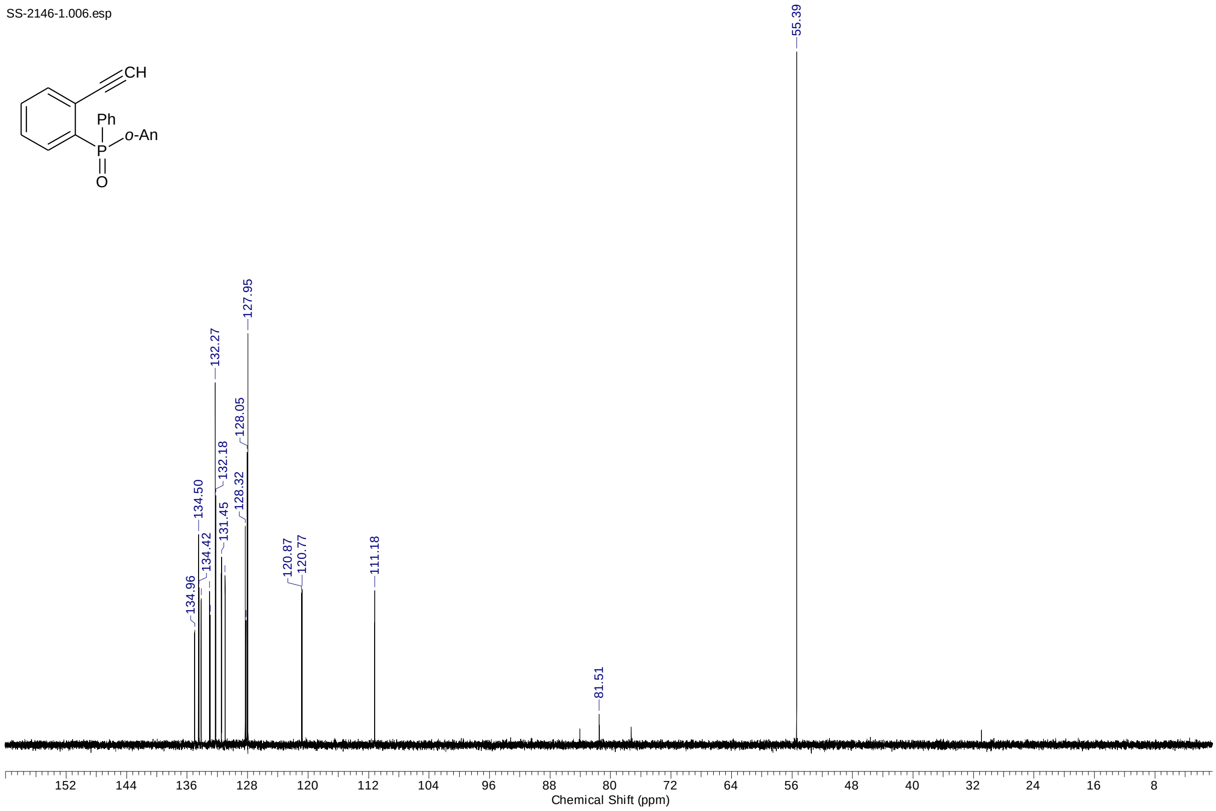

DEPT 135 NMR spectrum of (2-ethynylphenyl)phenyl(*o*-anisyl)phosphine oxide (**8g**) (125 MHz, $CDCl_3$)

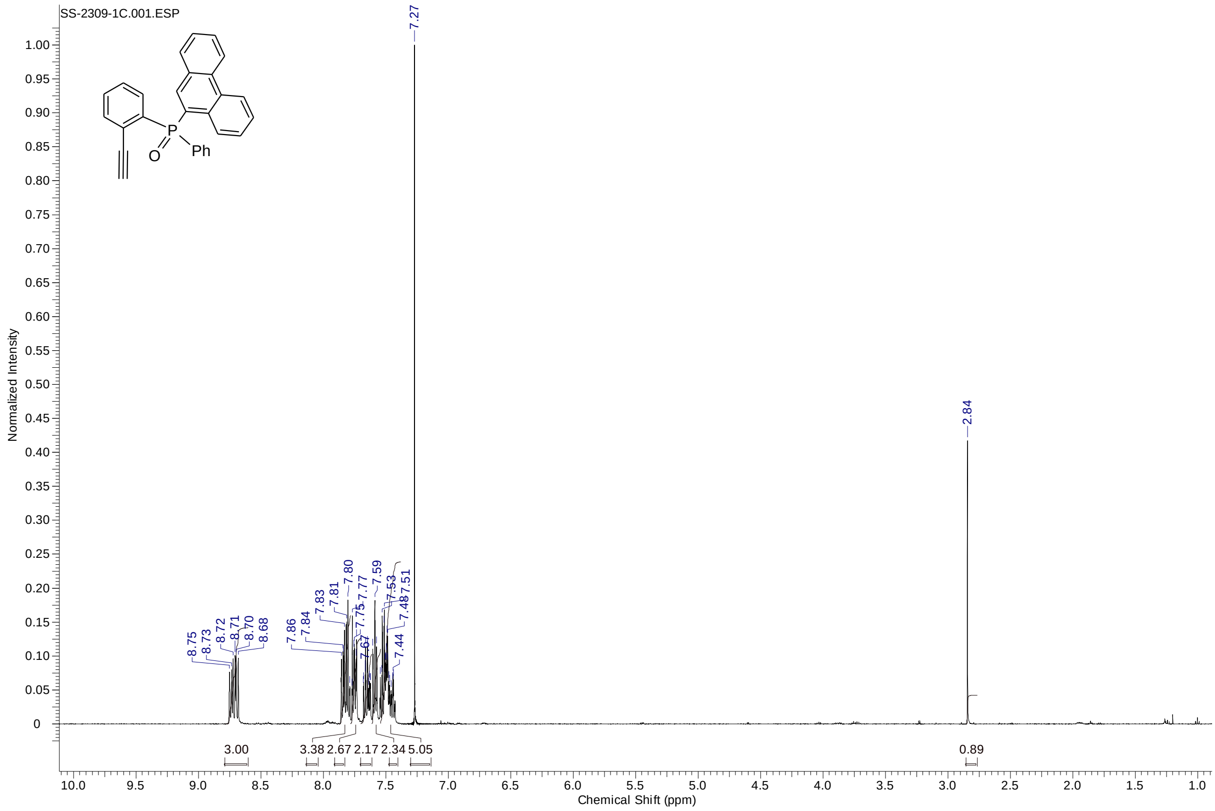

^1H NMR spectrum of (2-ethynylphenyl)phenyl(9-phenanthryl)phosphine oxide (**8h**) (500 MHz, $CDCl_3$)

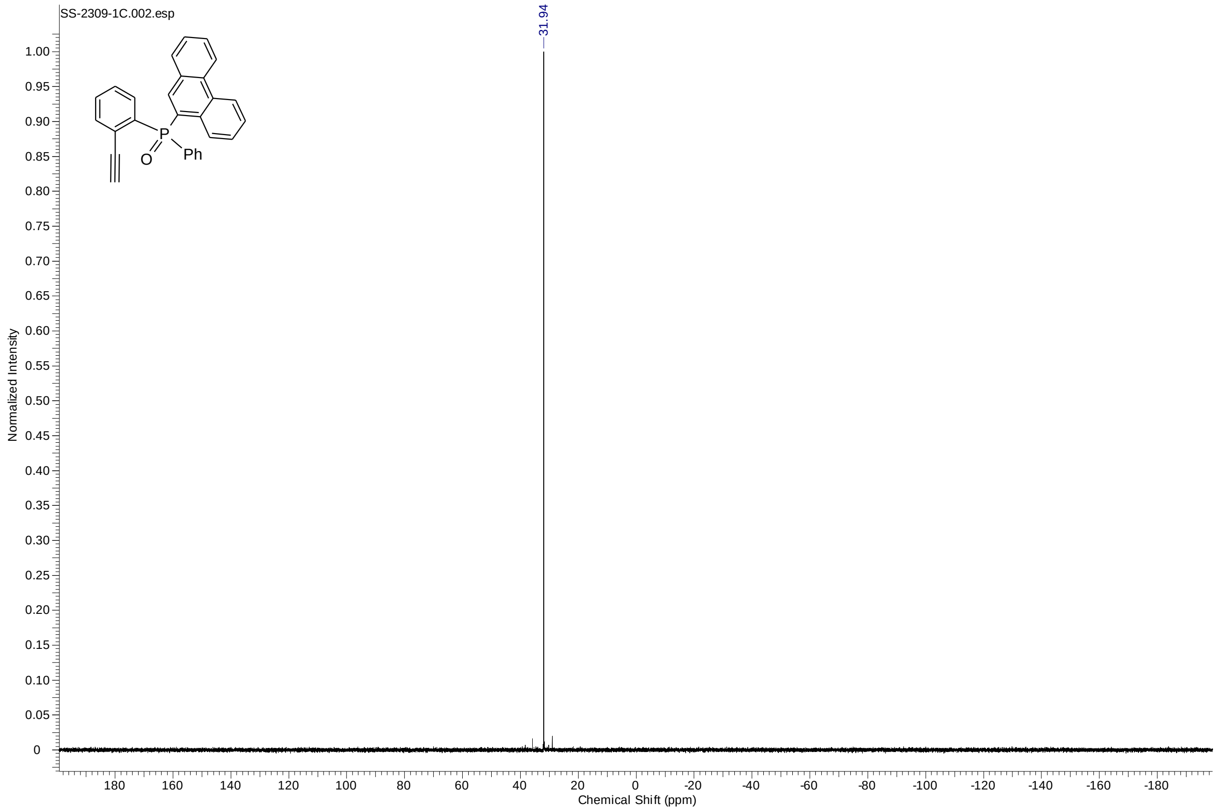

^{31}P NMR spectrum of (2-ethynylphenyl)phenyl(9-phenanthryl)phosphine oxide (**8h**) (202 MHz, $CDCl_3$)

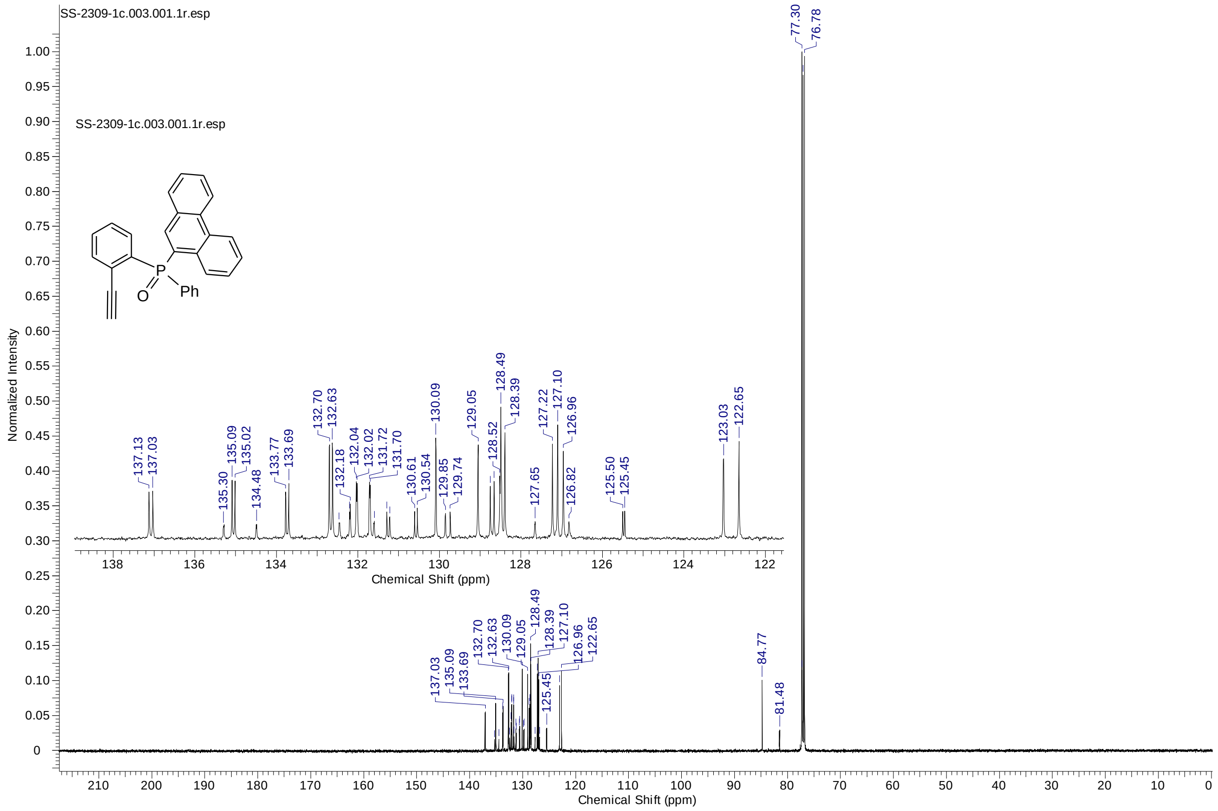

^{13}C{^{1}H} NMR spectrum of (2-ethynylphenyl)phenyl(9-phenanthryl)phosphine oxide (**8h**) (125 MHz, $CDCl_3$)

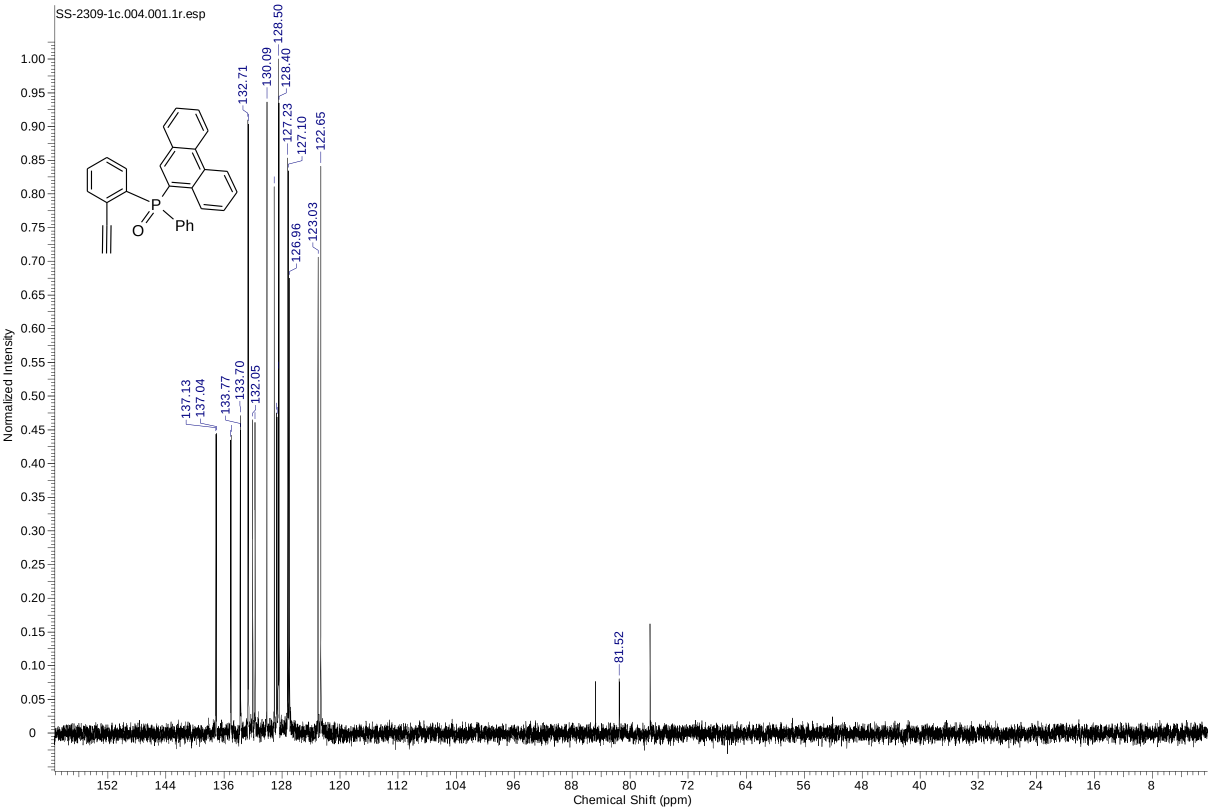

DEPT 135 NMR spectrum of (2-ethynylphenyl)phenyl(9-phenanthryl)phosphine oxide (**8h**) (125 MHz, $CDCl_3$)

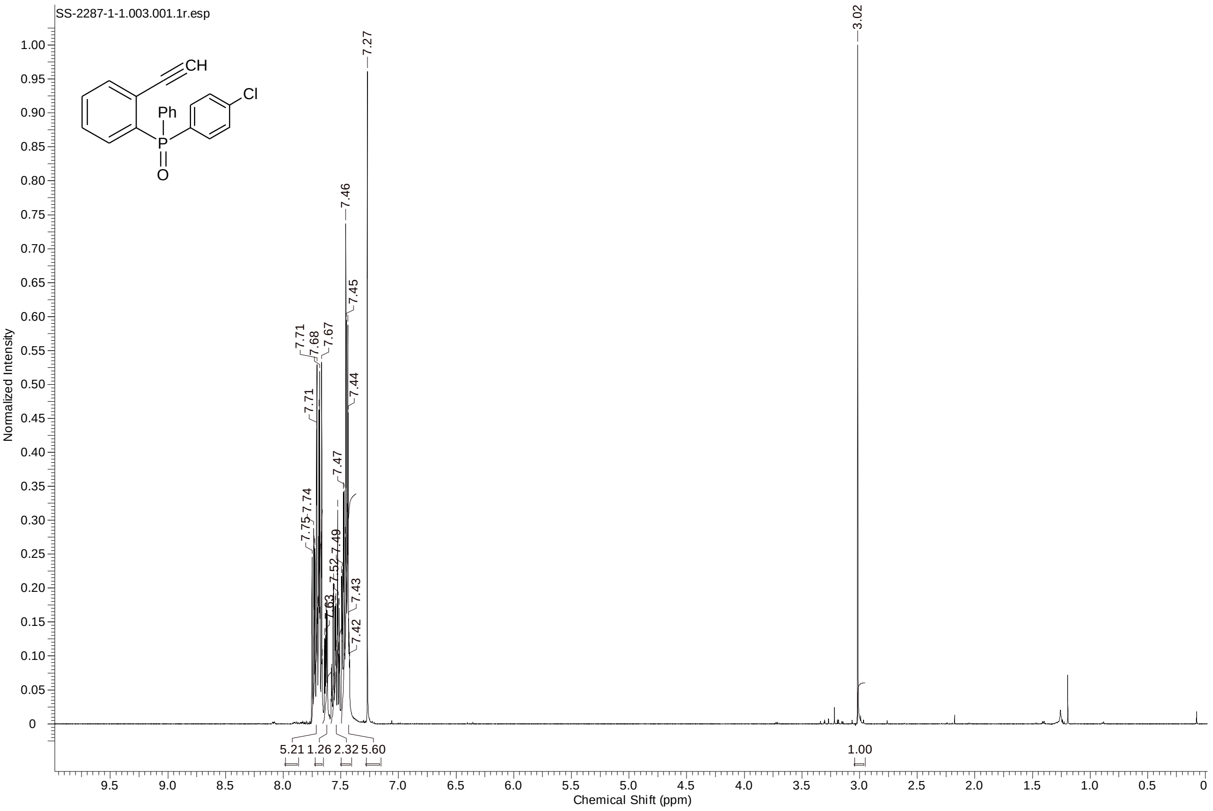

^{1}H NMR spectrum of (2-ethynylphenyl)phenyl(*p*-chlorophenyl)phosphine oxide (**8i**) (500 MHz, $CDCl_3$)

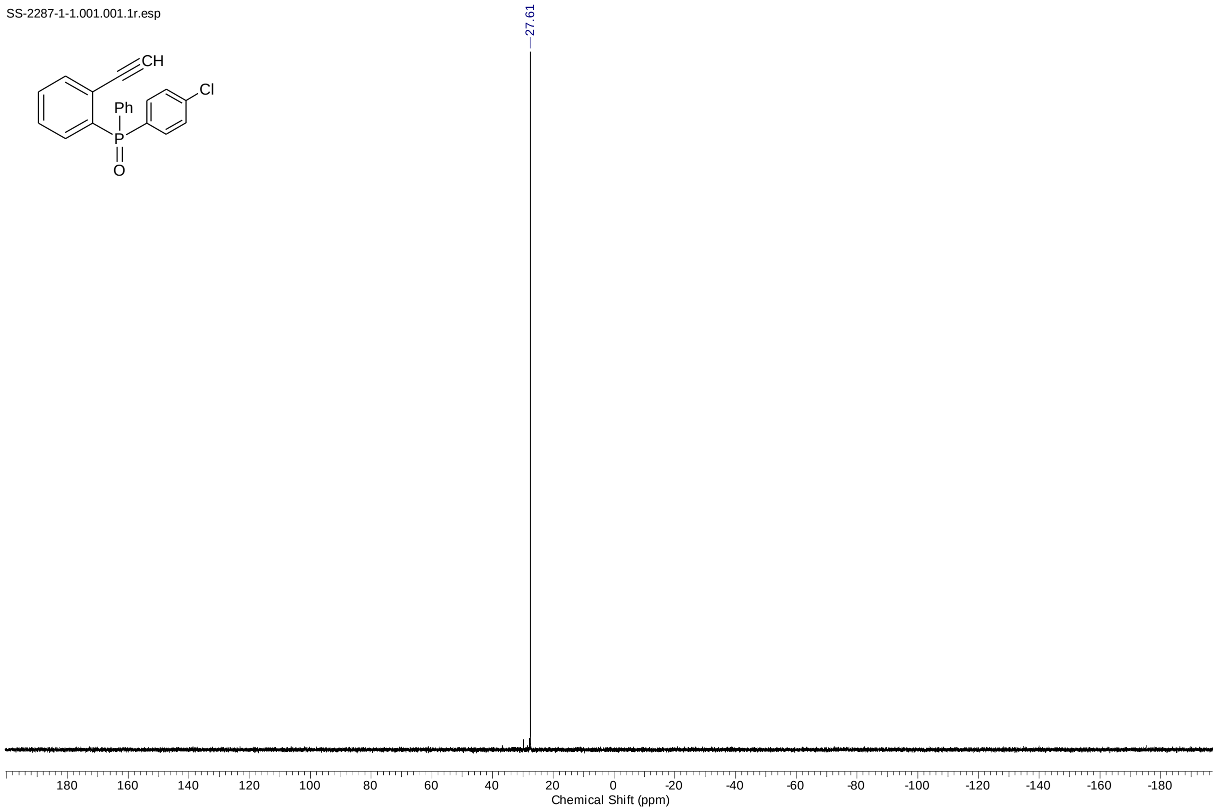

^{31}P NMR spectrum of (2-ethynylphenyl)phenyl(*p*-chlorophenyl)phosphine oxide (**8i**) (202 MHz, $CDCl_3$)

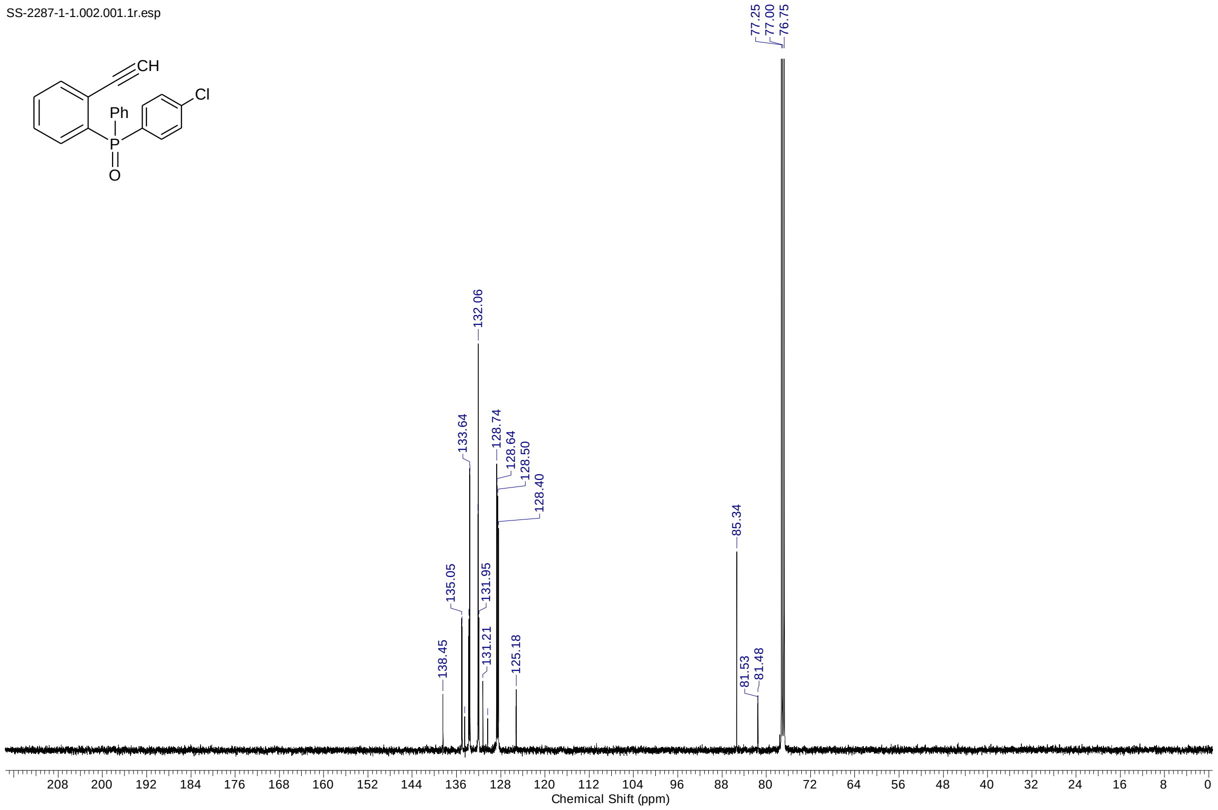

$^{13}C\{^1H\}$ NMR spectrum of (2-ethynylphenyl)phenyl(*p*-chlorophenyl)phosphine oxide (**8i**) (125 MHz, $CDCl_3$)

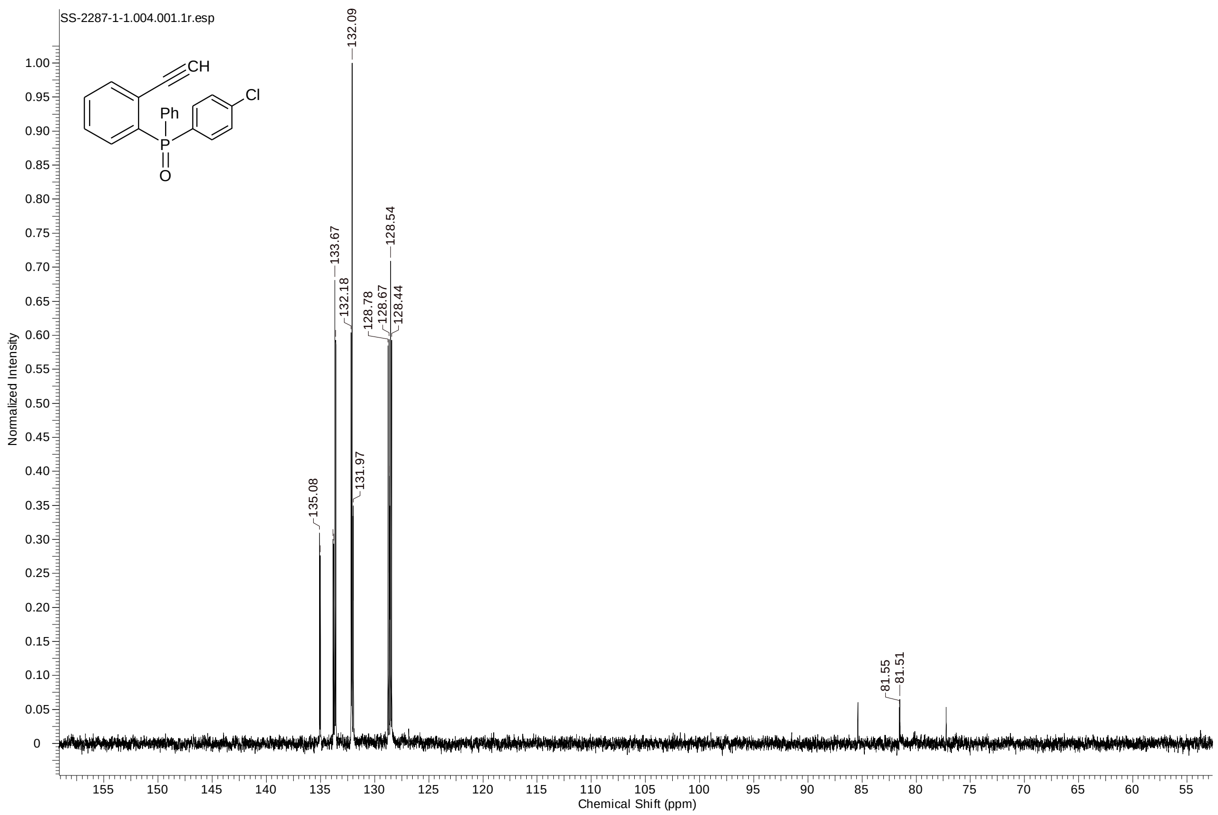

DEPT 135 NMR spectrum of (2-ethynylphenyl)phenyl(*p*-chlorophenyl)phosphine oxide (**8i**) (125 MHz, $CDCl_3$)

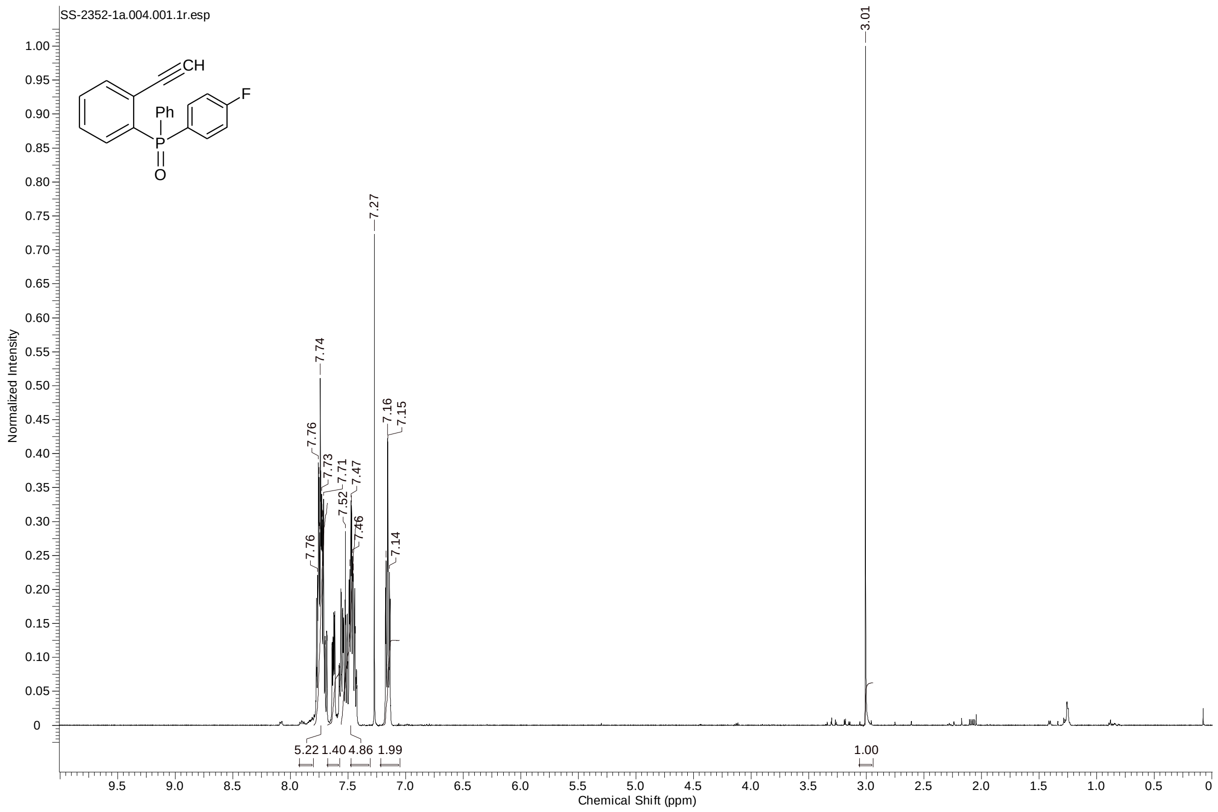

^{1}H NMR spectrum of (2-ethynylphenyl)phenyl(*p*-fluorophenyl)phosphine oxide (**8j**) (500 MHz, $CDCl_3$)

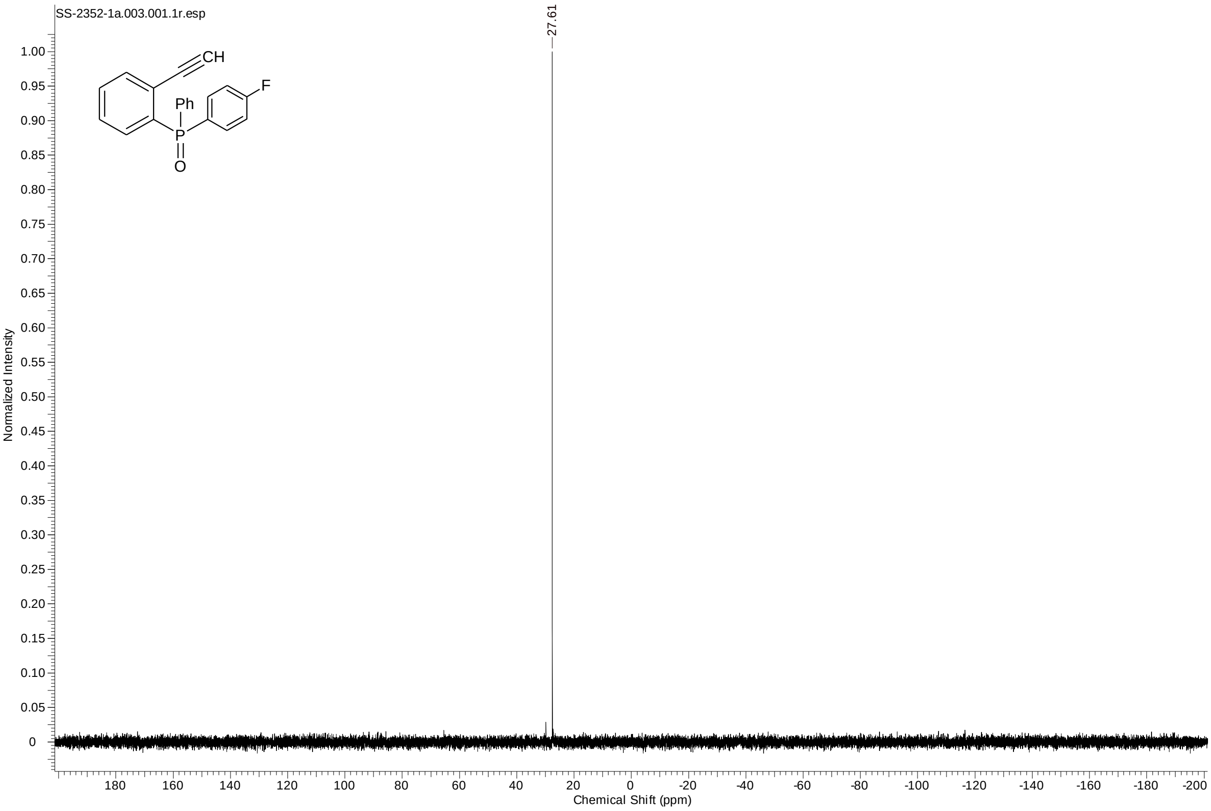

^{31}P NMR spectrum of (2-ethynylphenyl)phenyl(*p*-fluorophenyl)phosphine oxide (**8j**) (202 MHz, $CDCl_3$)

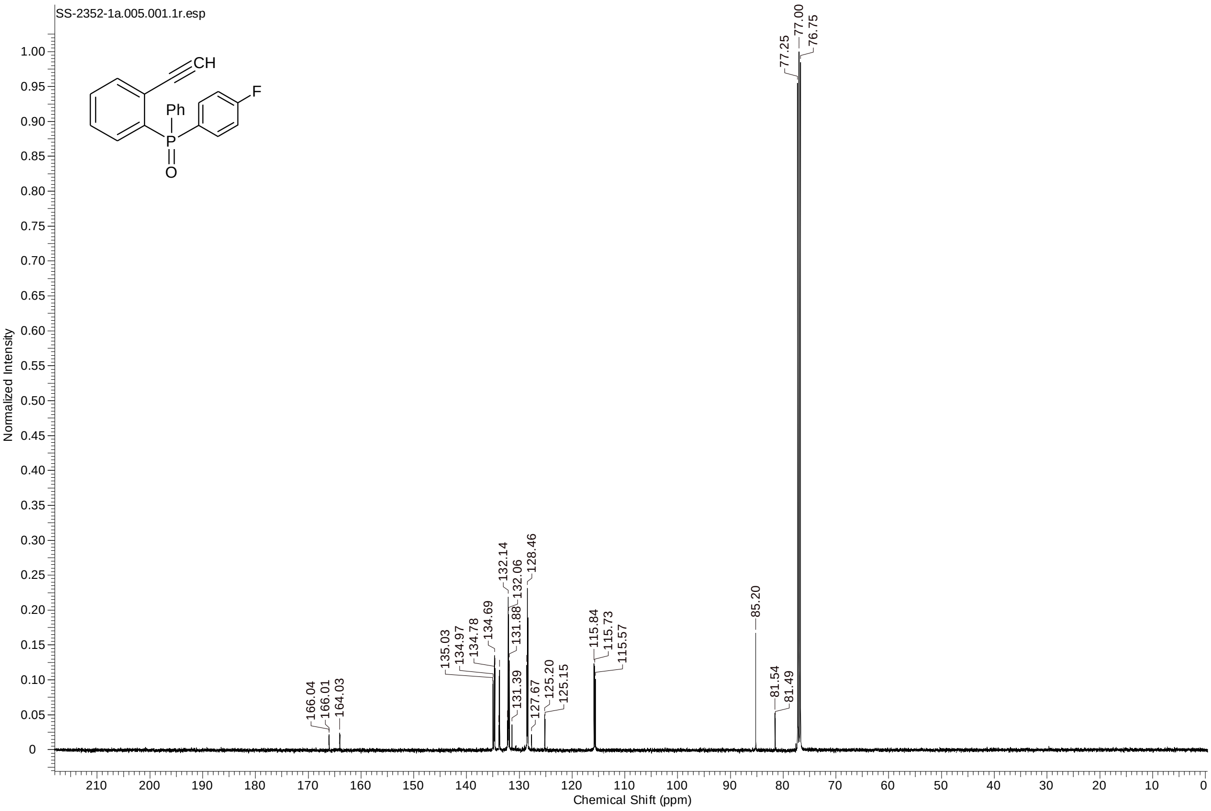

$^{13}C\{^1H\}$ NMR spectrum of (2-ethynylphenyl)phenyl(*p*-fluorophenyl)phosphine oxide (**8j**) (125 MHz, $CDCl_3$)

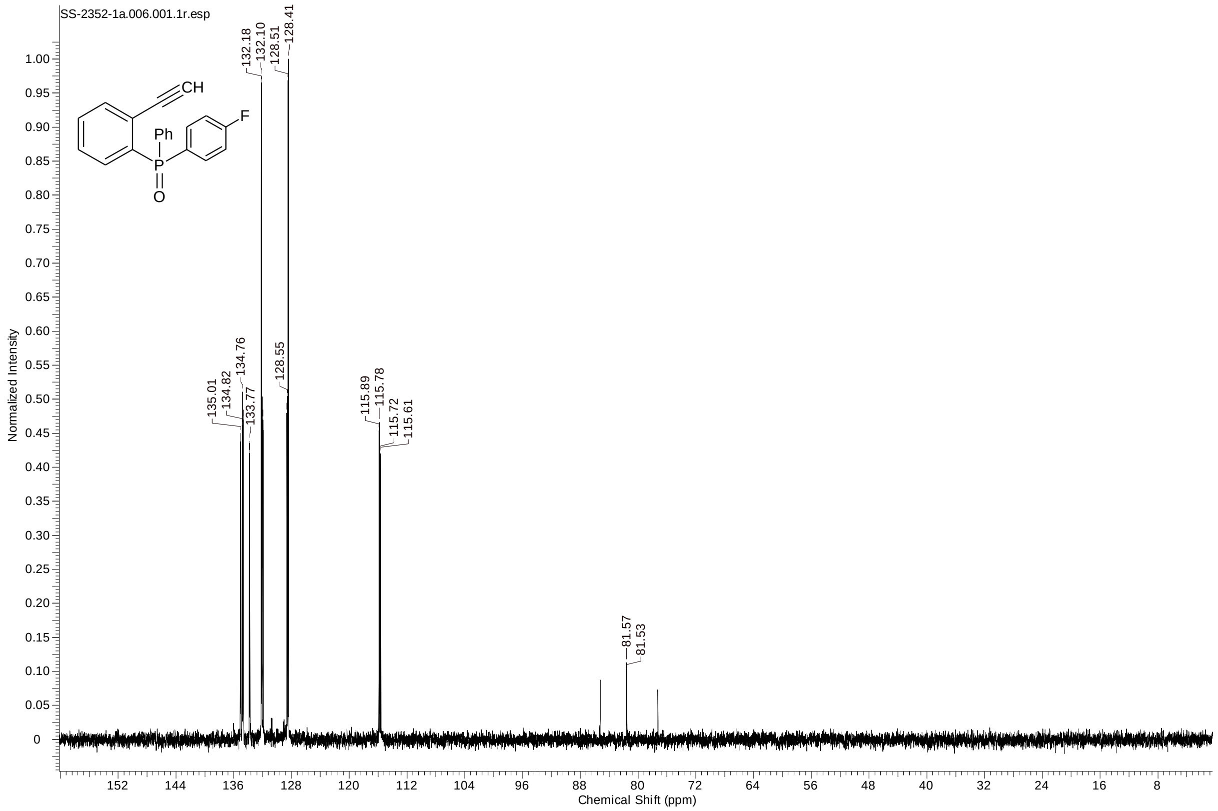

DEPT 135 NMR spectrum of (2-ethynylphenyl)phenyl(*p*-fluorophenyl)phosphine oxide (**8j**) (125 MHz, $CDCl_3$)

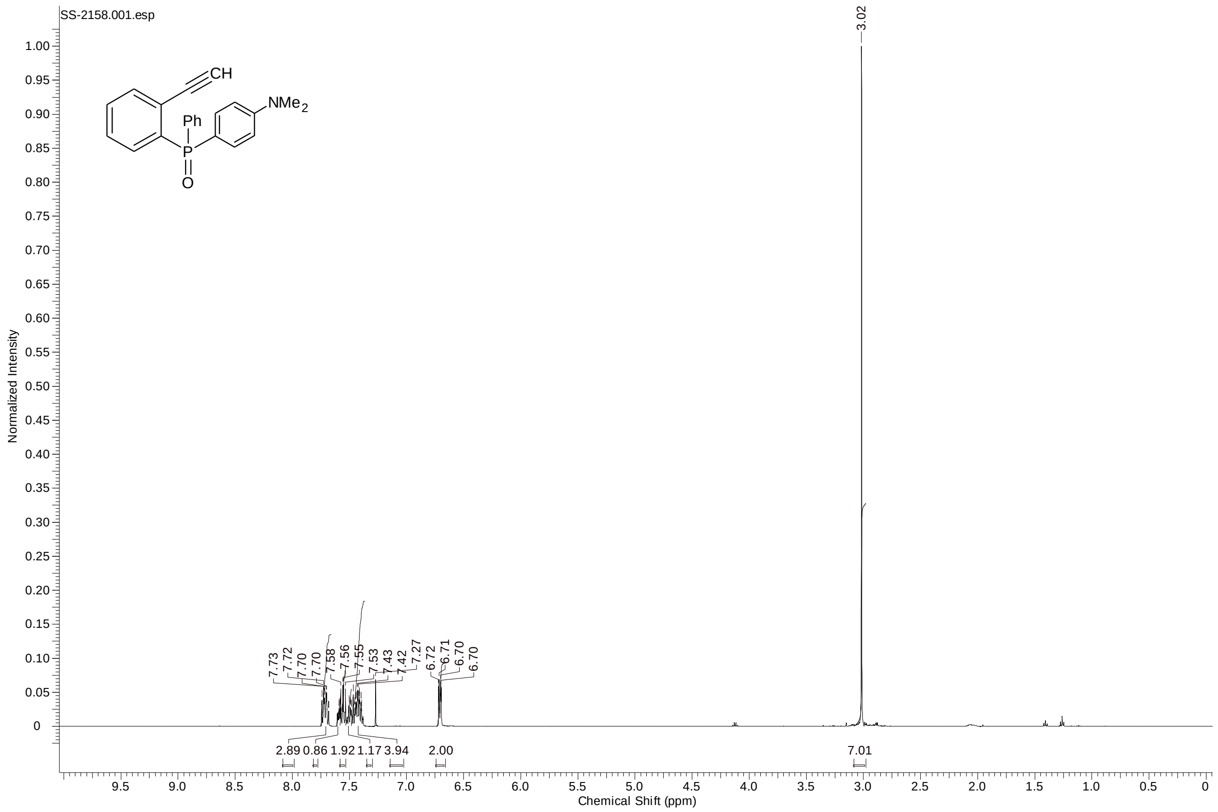

^{1}H NMR spectrum of (2-ethynylphenyl)phenyl(*p-N,N*-dimethylaminophenyl)phosphine oxide (**8k**) (500 MHz, $CDCl_3$)

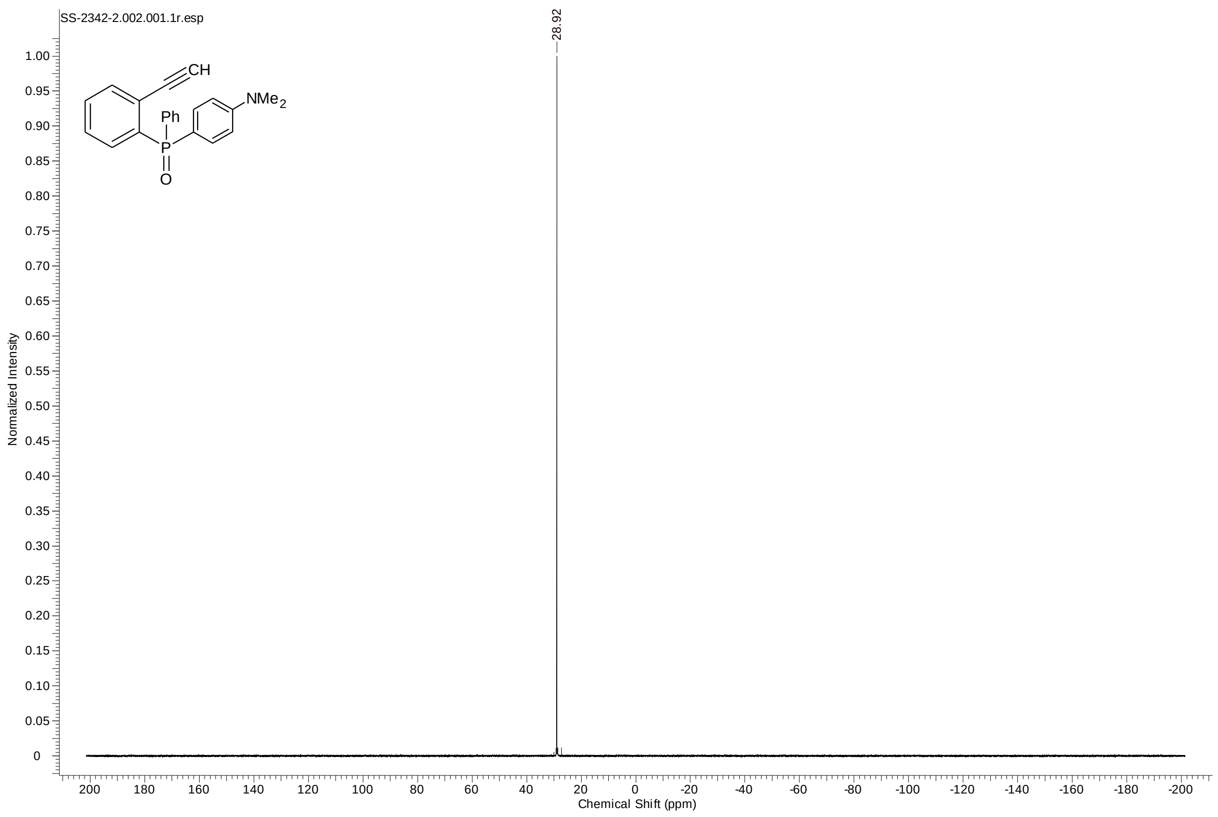

^{31}P NMR spectrum of (2-ethynylphenyl)phenyl(*p-N,N*-dimethylaminophenyl)phosphine oxide (**8k**) (202 MHz, $CDCl_3$)

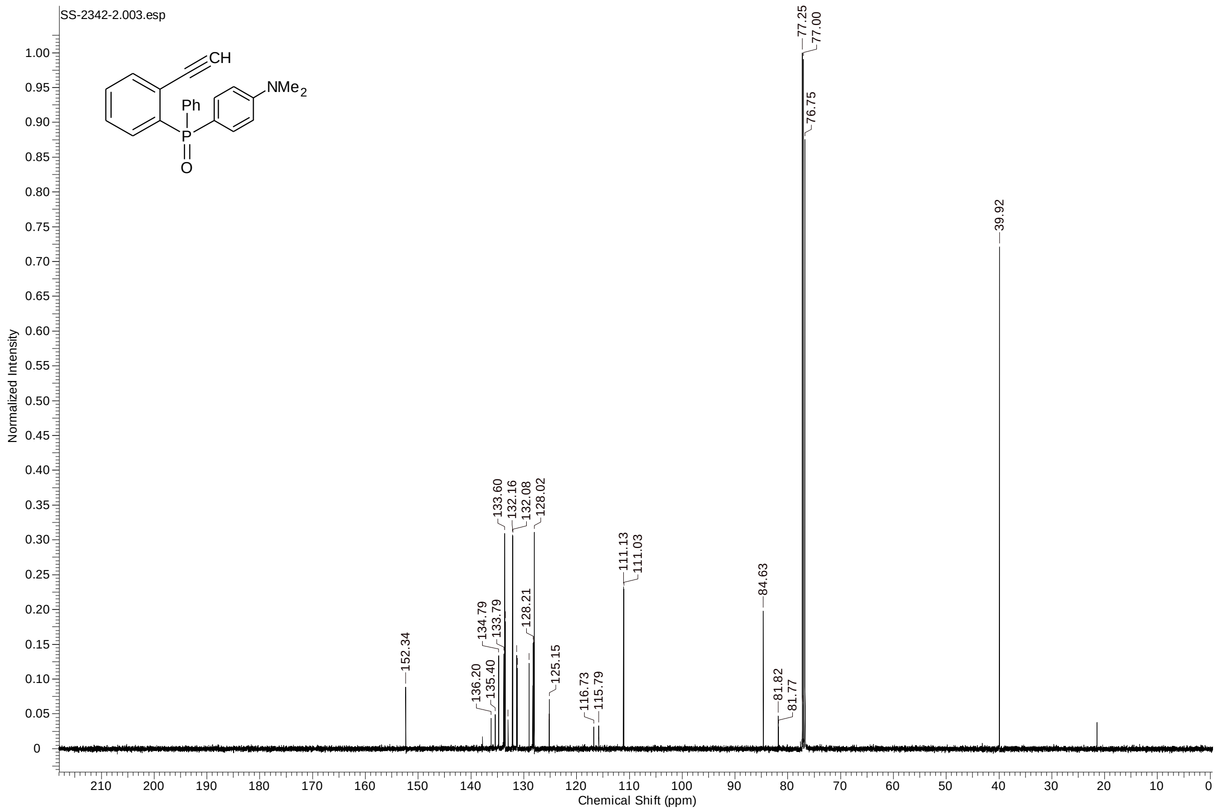

$^{13}C\{^1H\}$ NMR spectrum of (2-ethynylphenyl)phenyl(*p-N,N*-dimethylaminophenyl)phosphine oxide (**8k**) (125 MHz, $CDCl_3$)

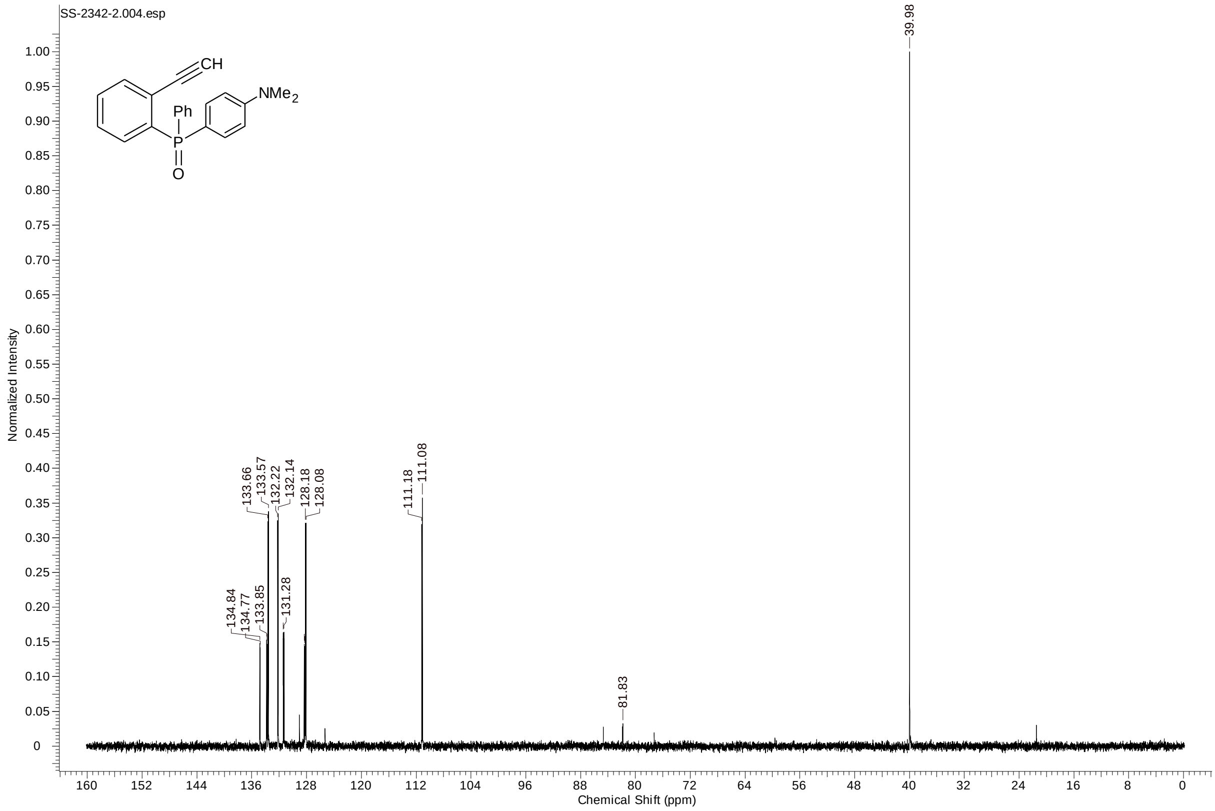

DEPT 135 NMR spectrum of (2-ethynylphenyl)phenyl(*p-N,N*-dimethylaminophenyl)phosphine oxide (**8k**) (125 MHz, $CDCl_3$)

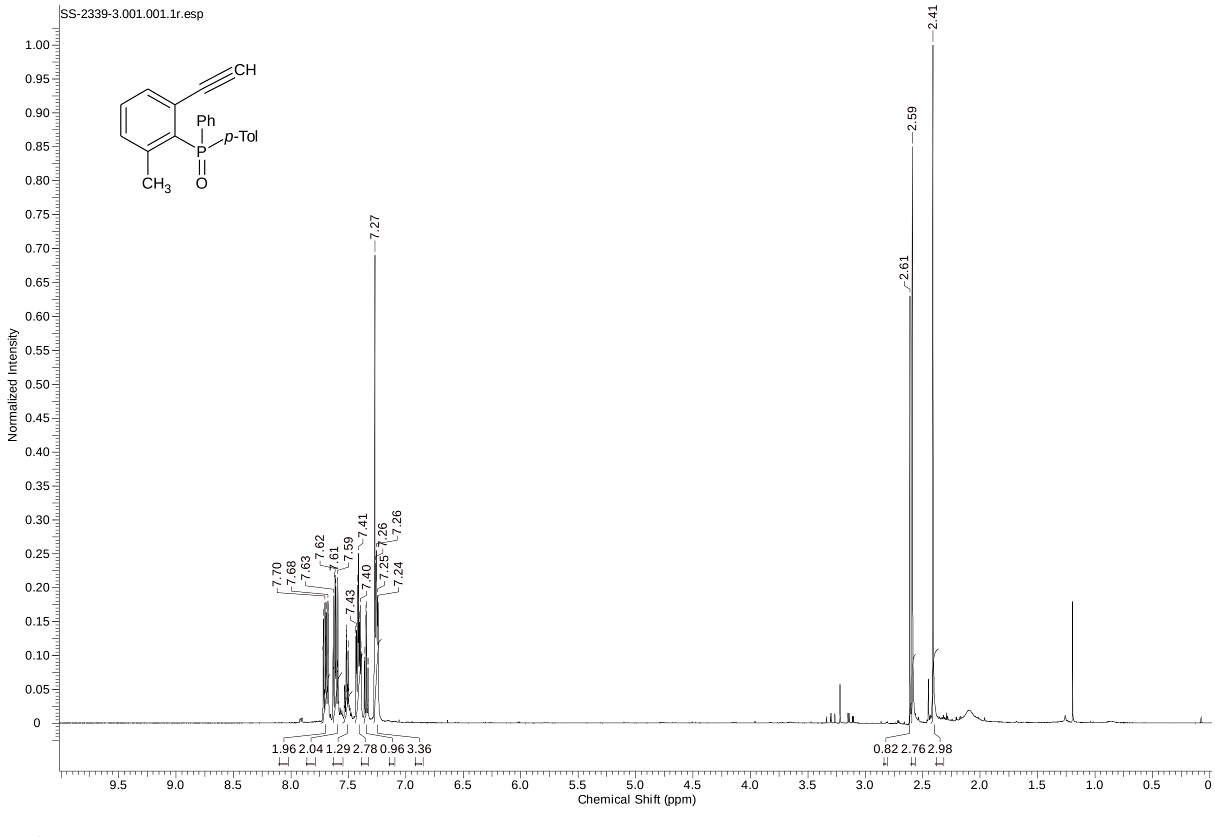

^{1}H NMR spectrum of (2-ethynyl-6-methylphenyl)phenyl(*p*-tolyl)phosphine oxide (**10a**) (500 MHz, $CDCl_3$)

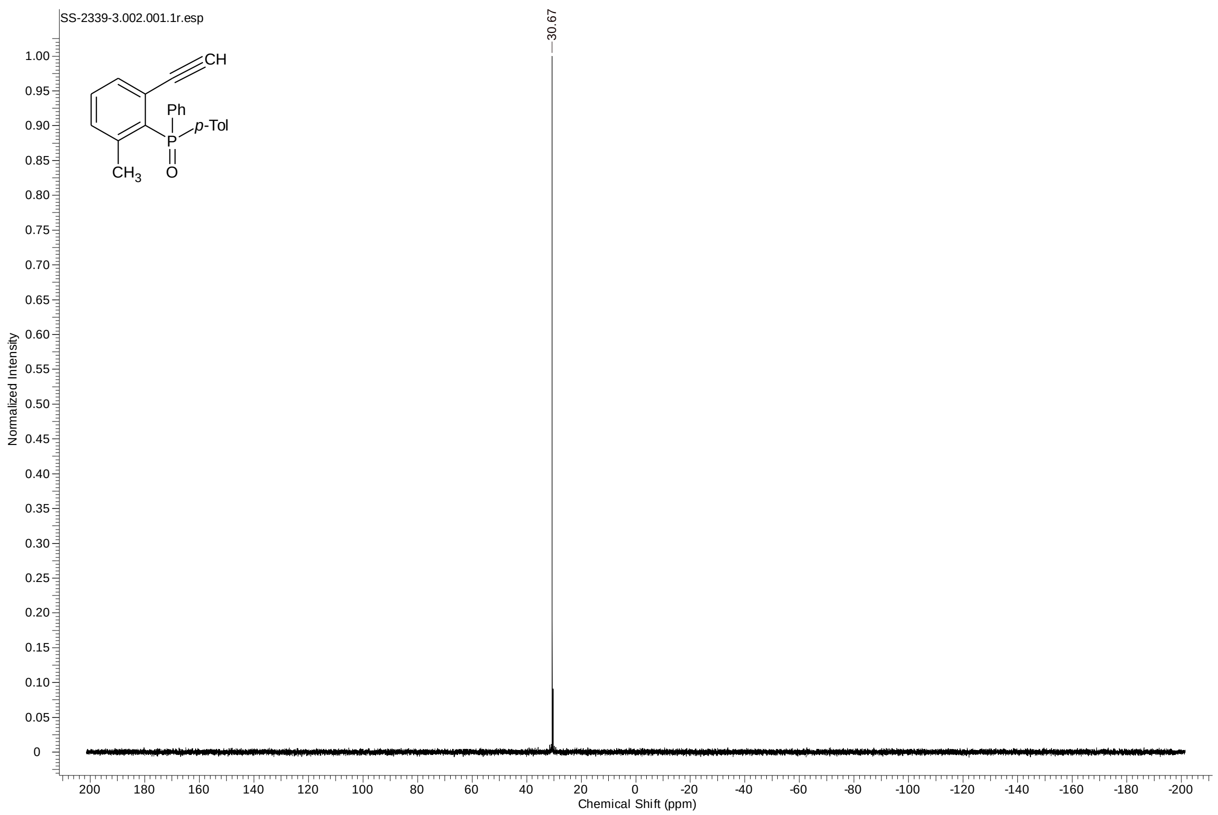

^{31}P NMR spectrum of (2-ethynyl-6-methylphenyl)phenyl(*p*-tolyl)phosphine oxide (**10a**) (500 MHz, $CDCl_3$)

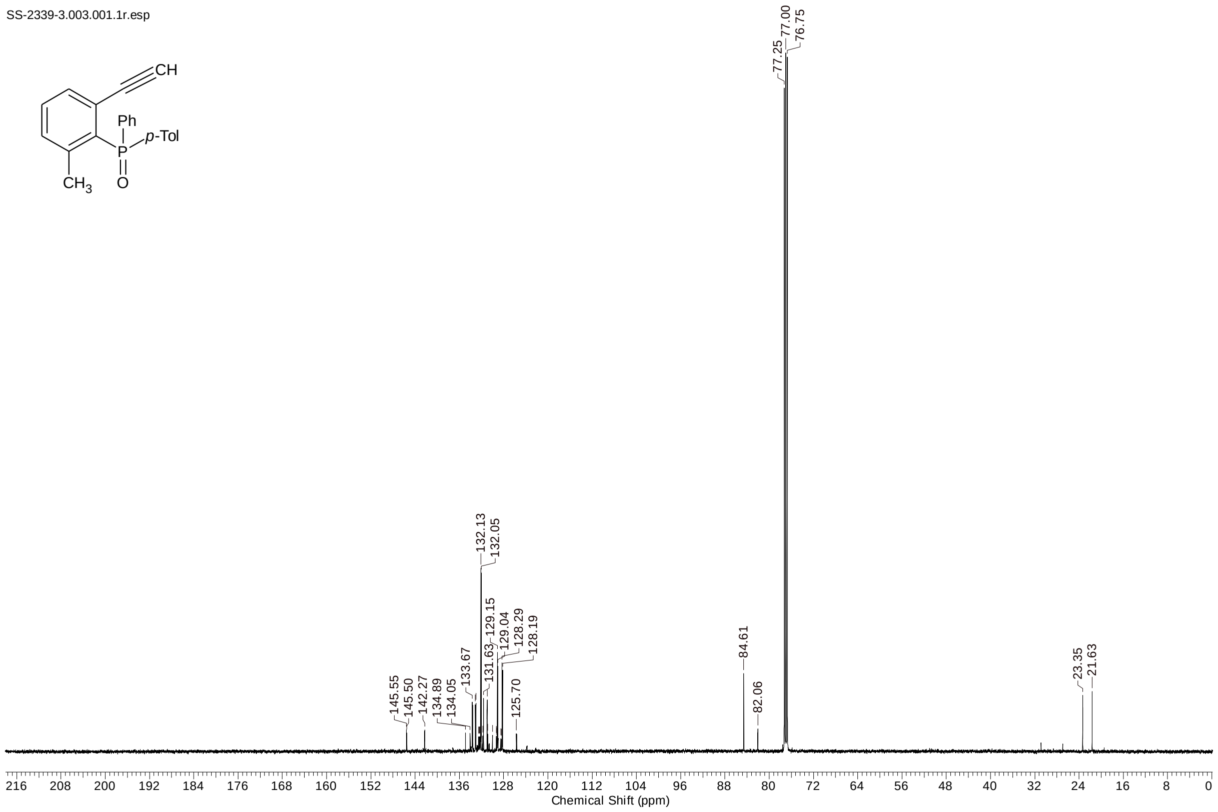

^{13}C{^{1}H} NMR spectrum of (2-ethynyl-6-methylphenyl)phenyl(*p*-tolyl)phosphine oxide (**10a**) (125 MHz, $CDCl_3$)

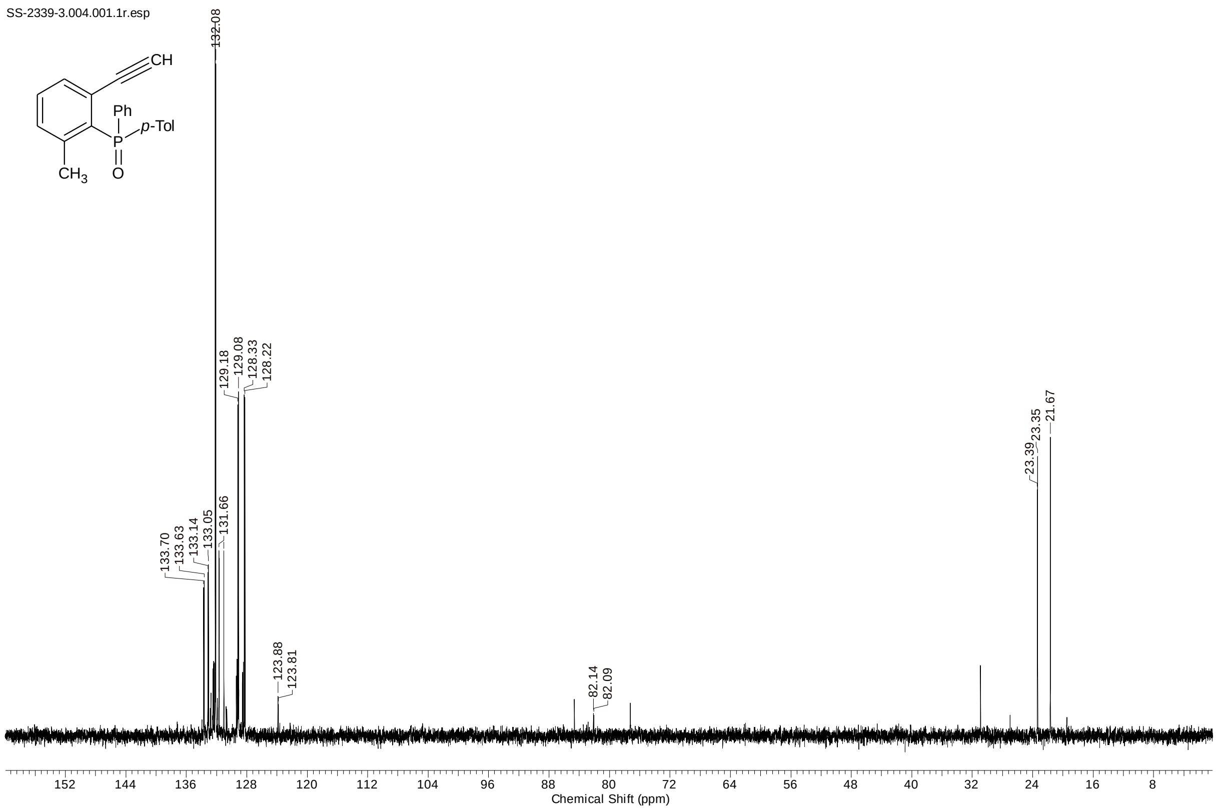

DEPT 135 NMR spectrum of (2-ethynyl-6-methylphenyl)phenyl(*p*-tolyl)phosphine oxide (**10a**) (125 MHz, $CDCl_3$)

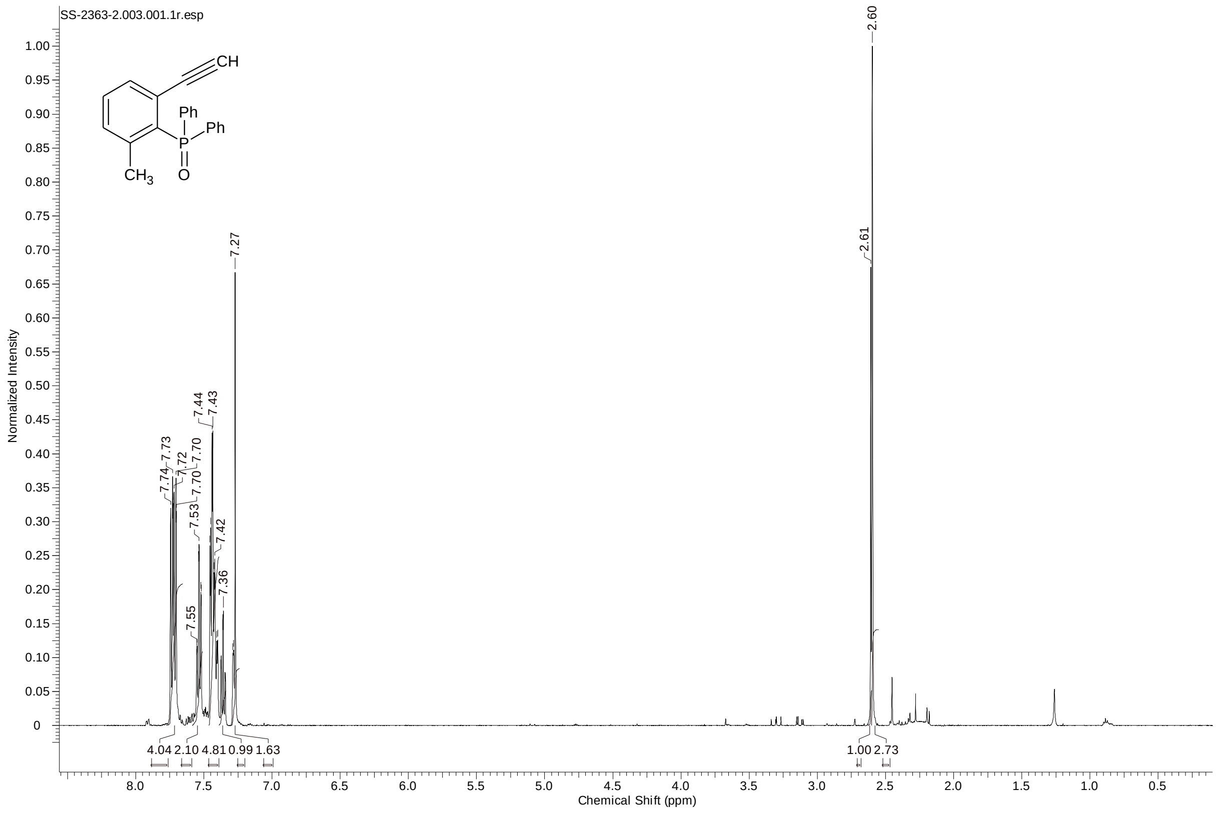

^{1}H NMR spectrum of (2-ethynyl-6-methylphenyl)diphenylphosphine oxide (**10d**) (500 MHz, $CDCl_3$)

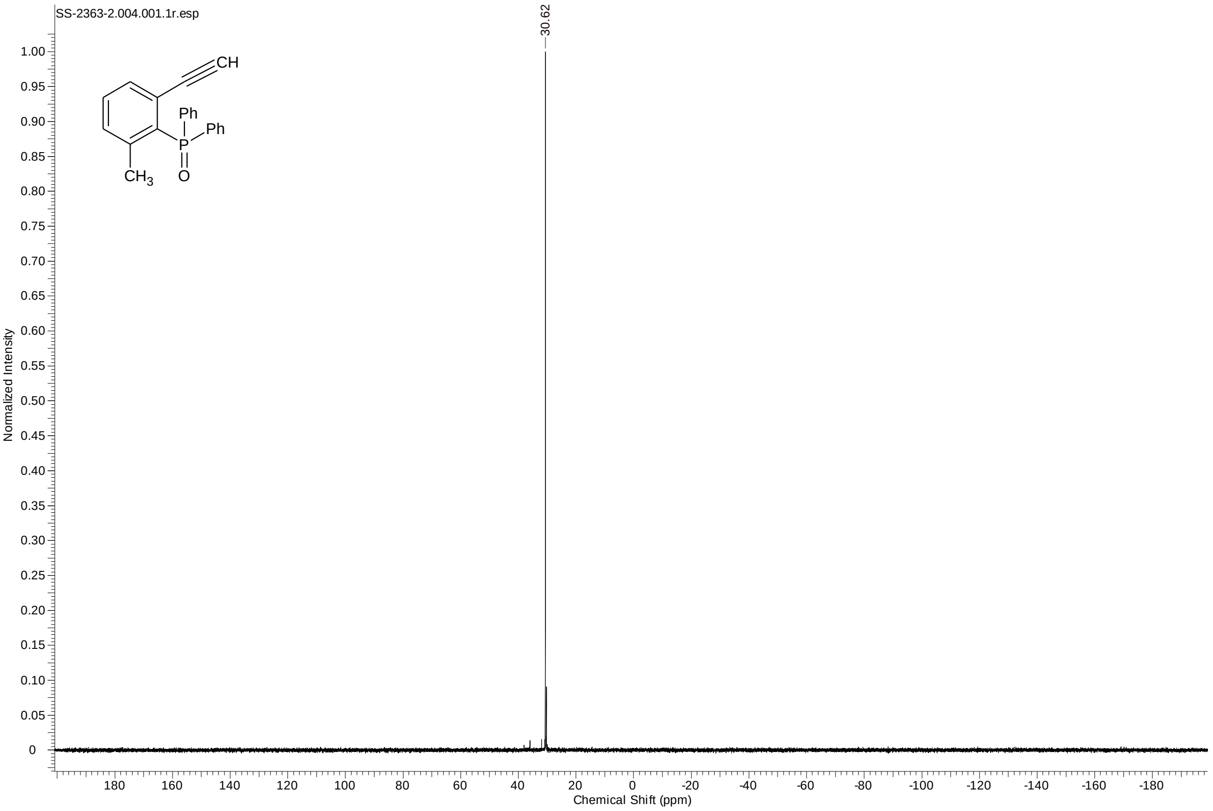

^{31}P NMR spectrum of (2-ethynyl-6-methylphenyl)diphenylphosphine oxide (**10d**) (202 MHz, $CDCl_3$)

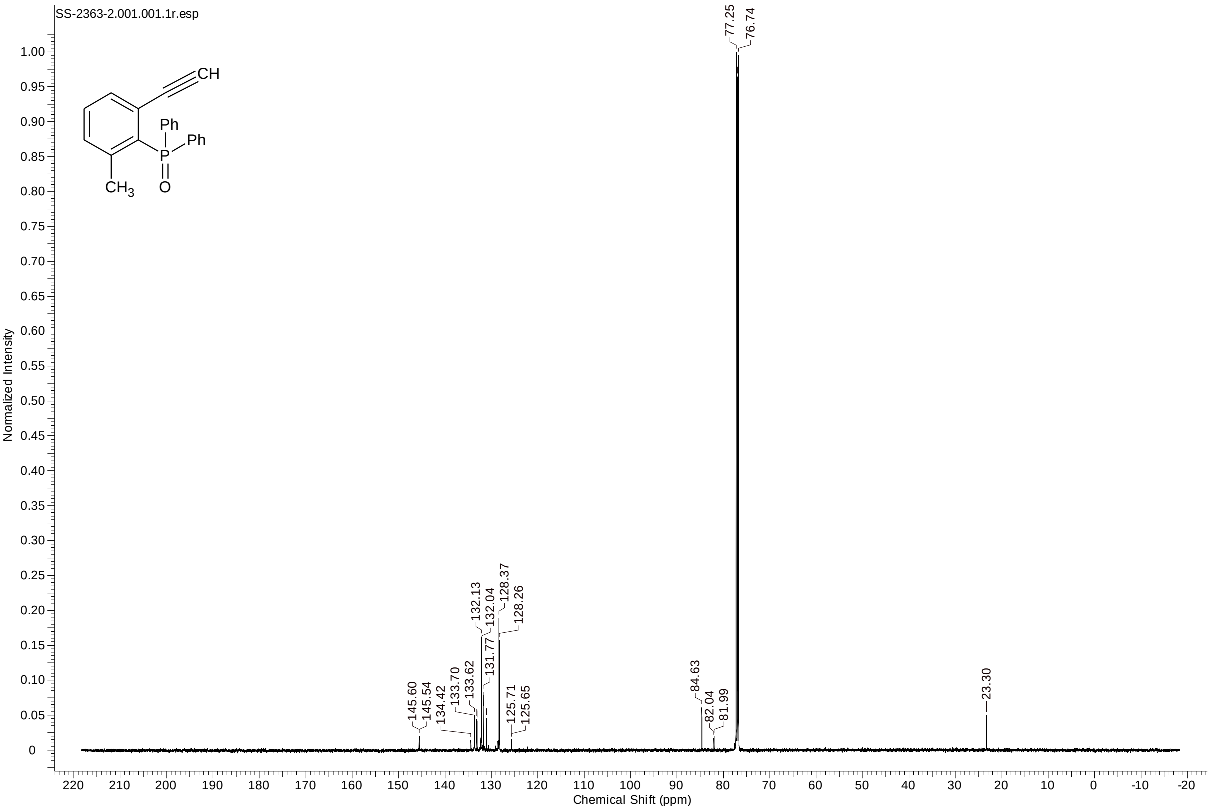

$^{13}C\{^1H\}$ NMR spectrum of (2-ethynyl-6-methylphenyl)diphenylphosphine oxide (**10d**) (125 MHz, $CDCl_3$)

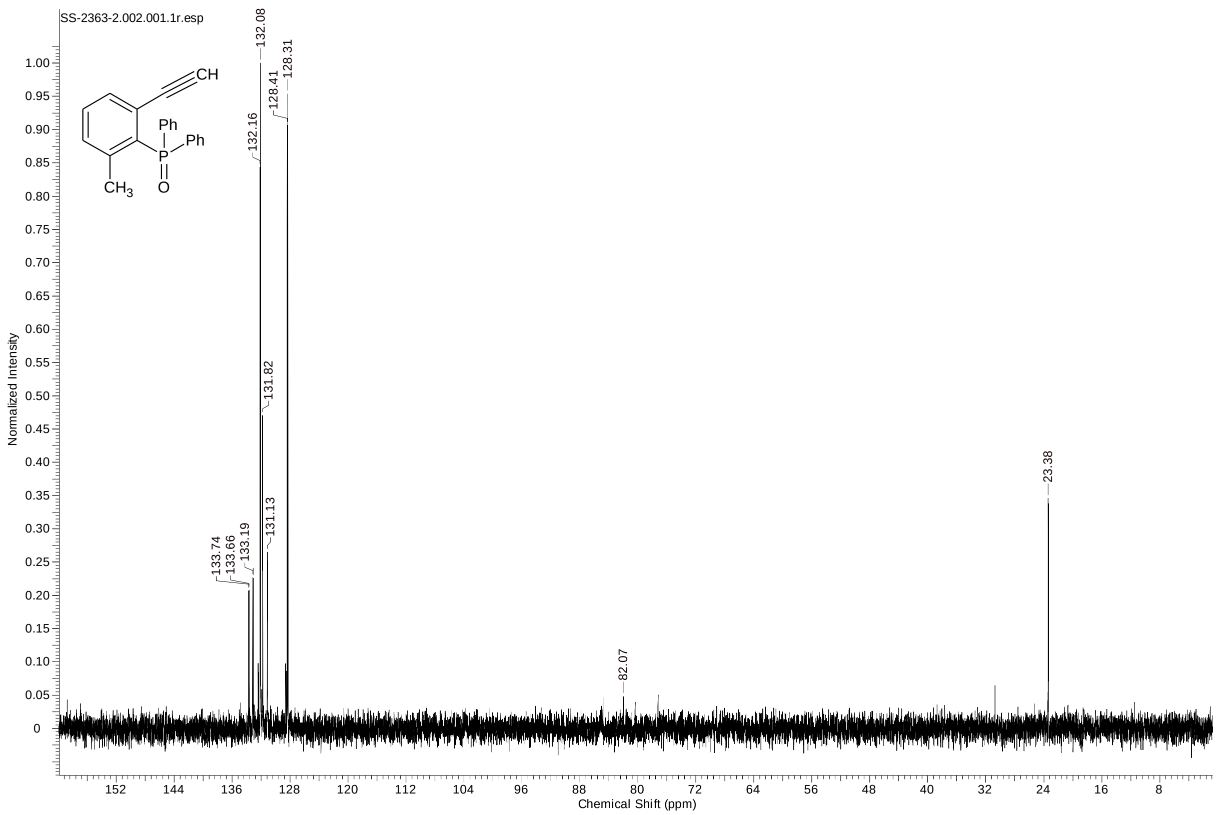

DEPT 135 NMR spectrum of (2-ethynyl-6-methylphenyl)diphenylphosphine oxide (**10d**) (125 MHz, $CDCl_3$)

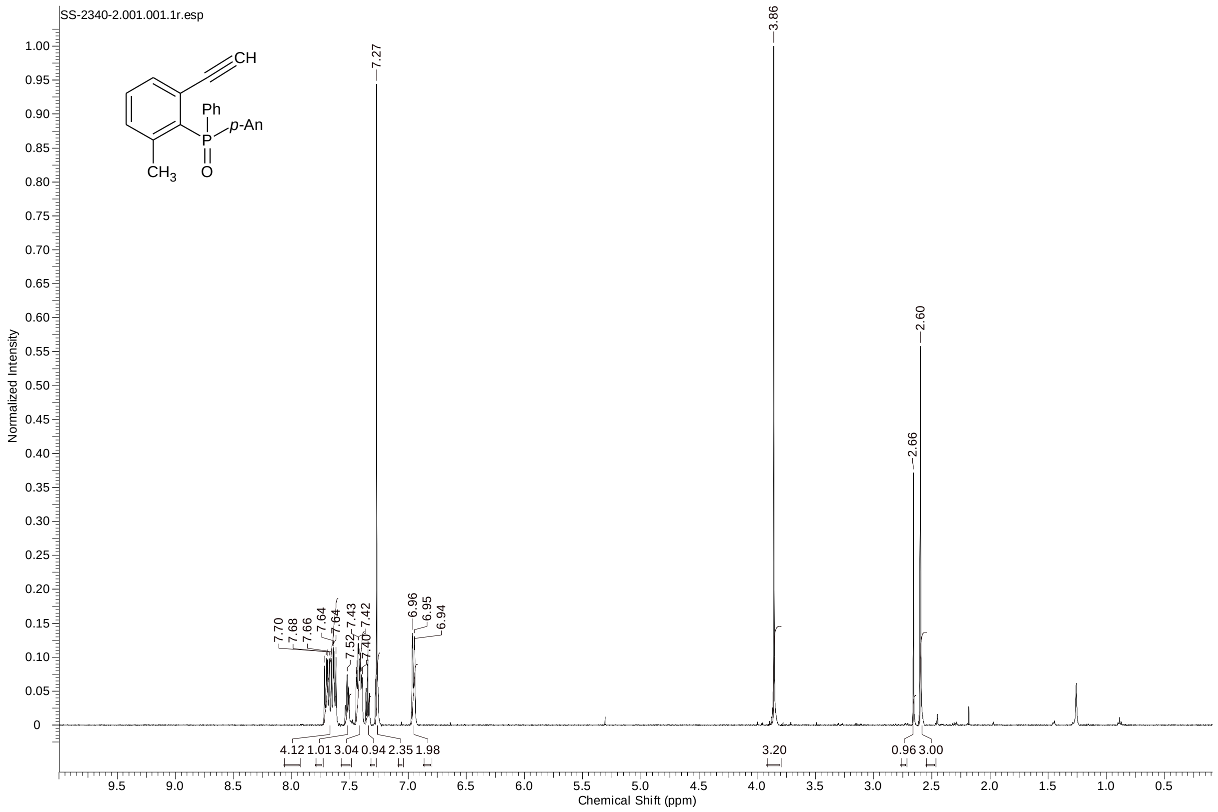

^1H NMR spectrum of (2-ethynyl-6-methylphenyl)phenyl(*p*-anisyl)phosphine oxide (**10e**) (500 MHz, $CDCl_3$)

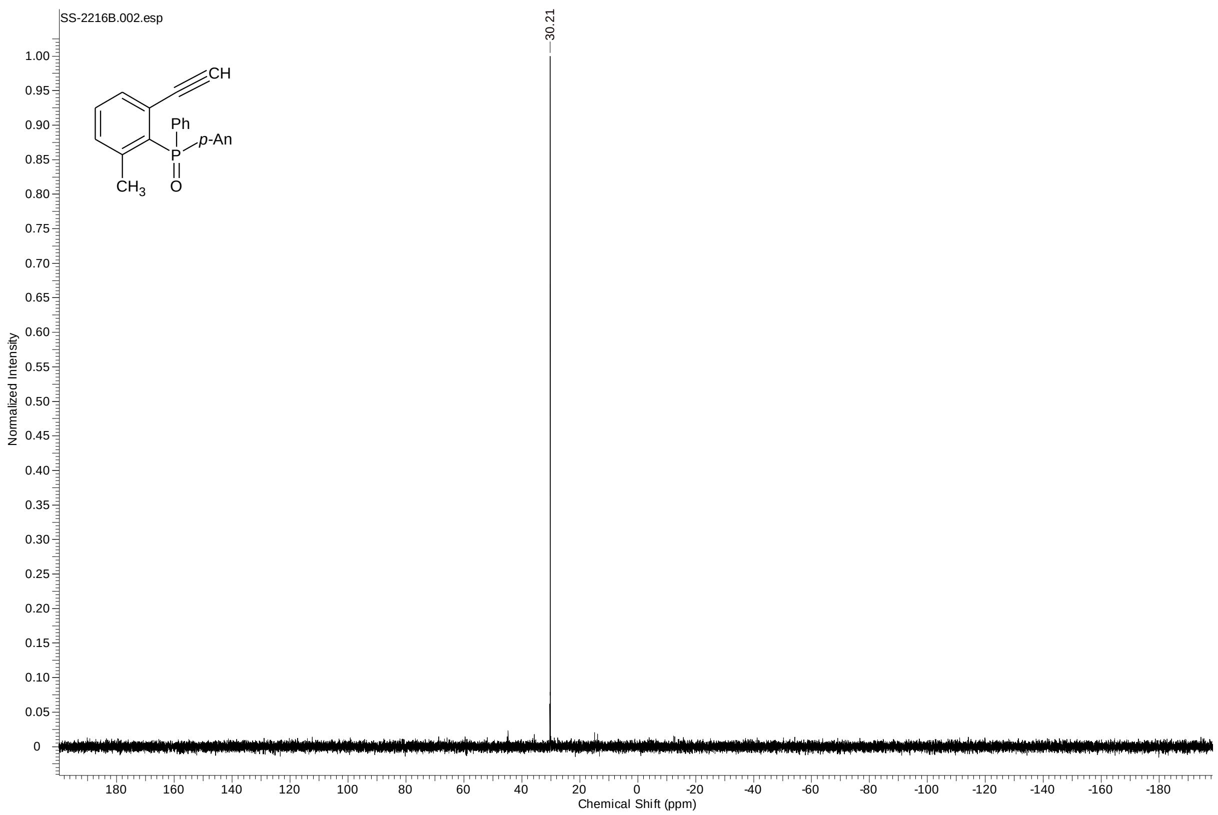

^{31}P NMR spectrum of (2-ethynyl-6-methylphenyl)phenyl(*p*-anisyl)phosphine oxide (**10e**) (202 MHz, $CDCl_3$)

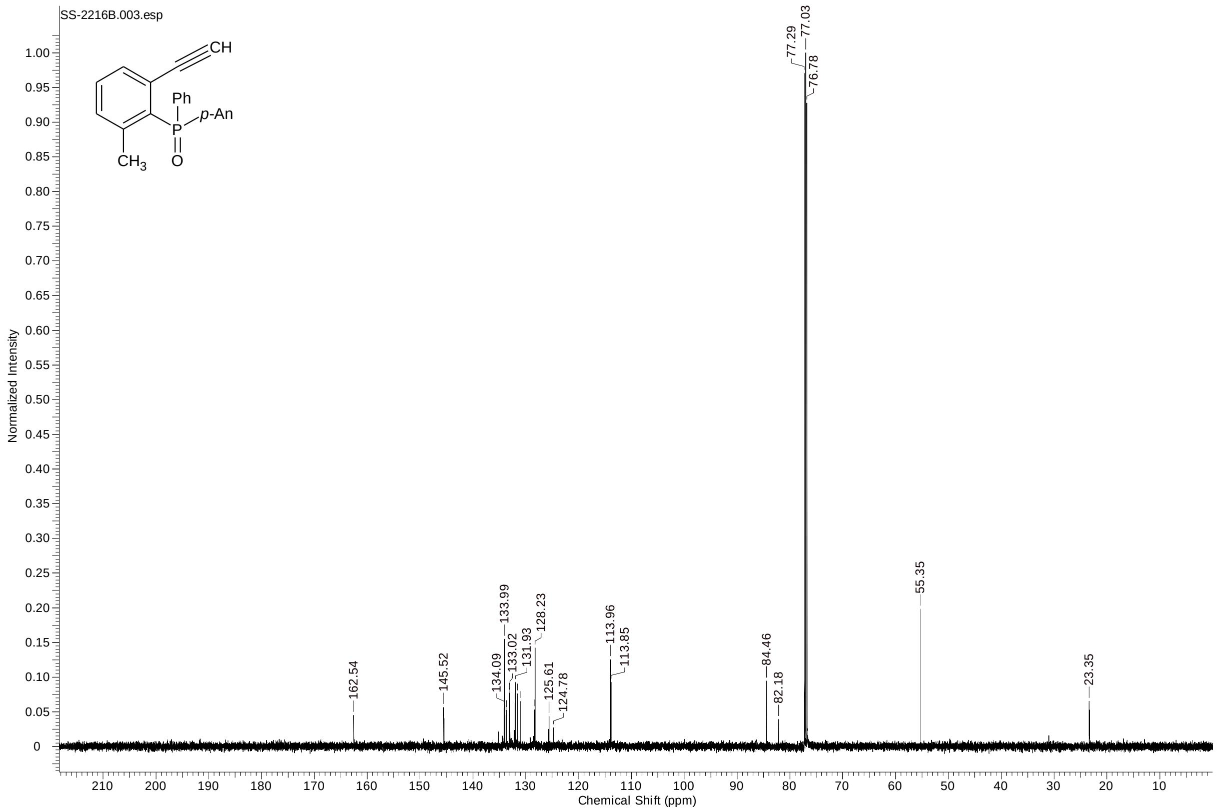

^{13}C{^{1}H} NMR spectrum of (2-ethynyl-6-methylphenyl)phenyl(*p*-anisyl)phosphine oxide (**10e**) (125 MHz, $CDCl_3$)

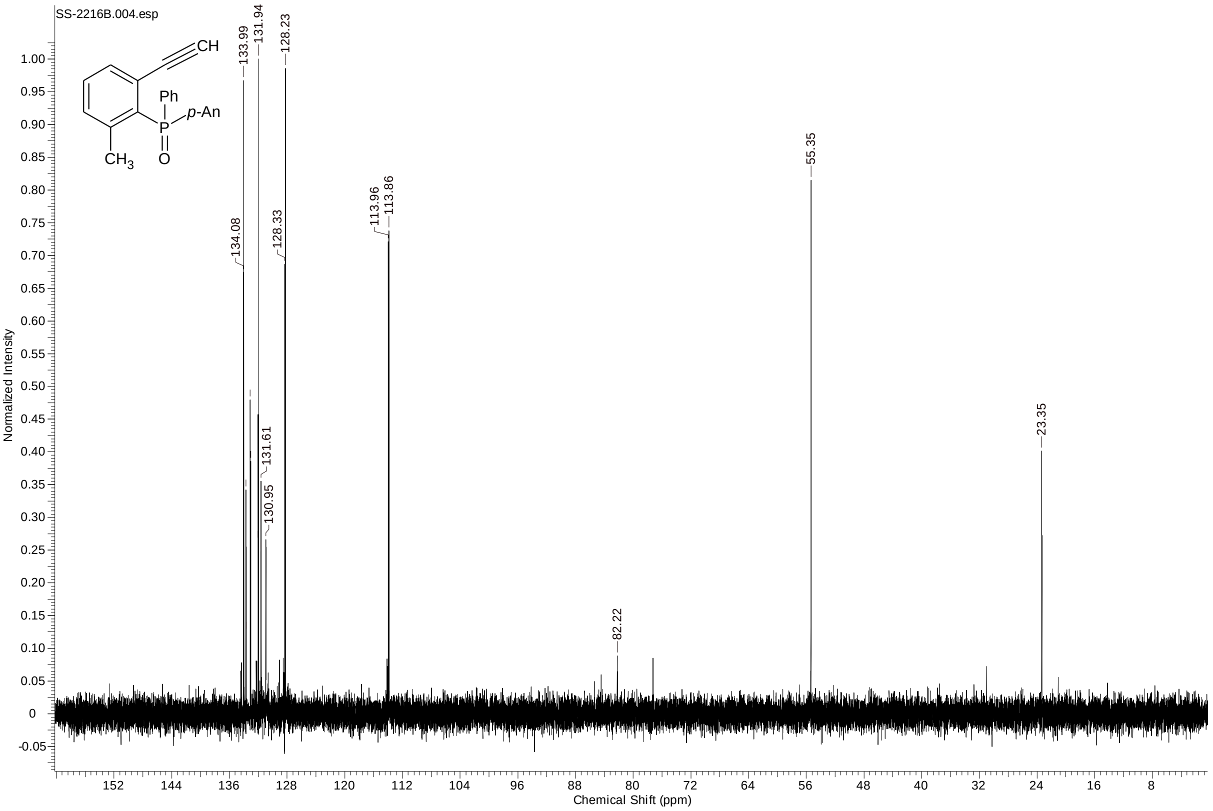

DEPT 135 NMR spectrum of (2-ethynyl-6-methylphenyl)phenyl(*p*-anisyl)phosphine oxide (**10e**) (125 MHz, $CDCl_3$)

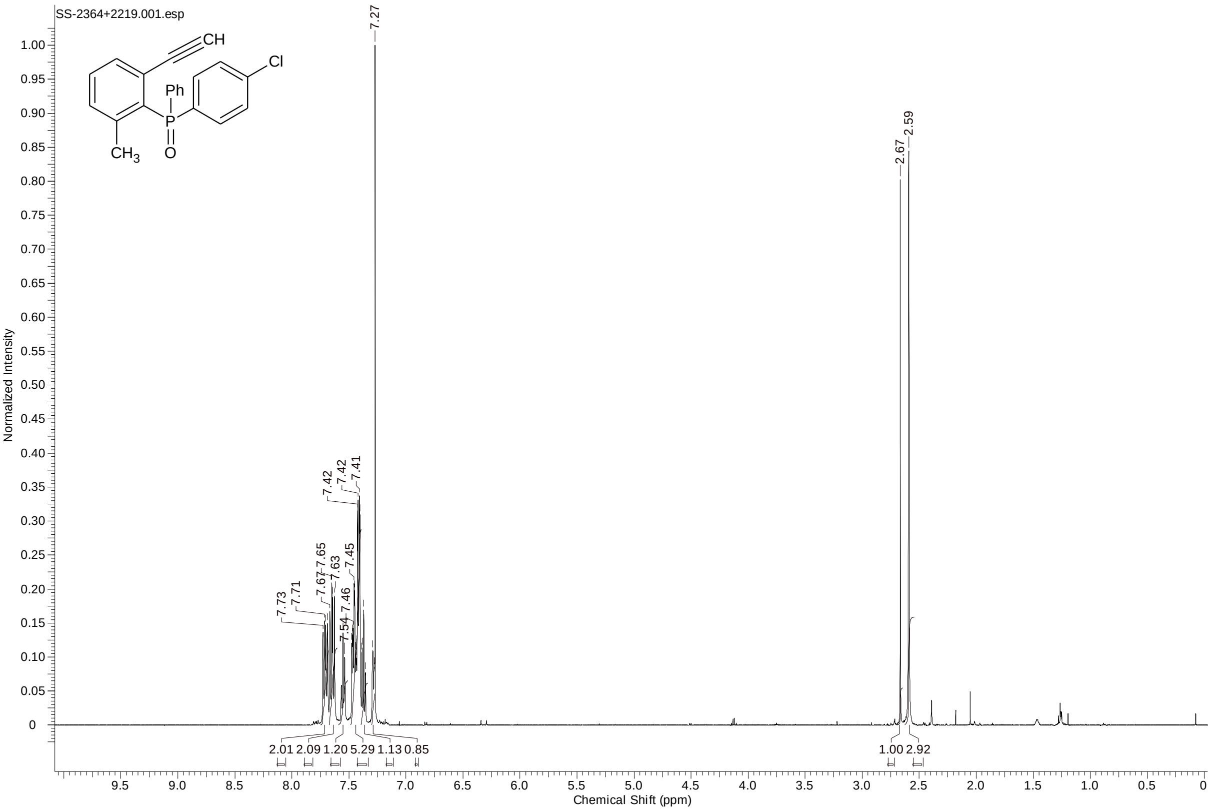

^{1}H NMR spectrum of (2-ethynyl-6-methylphenyl)phenyl(*p*-chlorophenyl)phosphine oxide (**10i**) (500 MHz, $CDCl_3$)

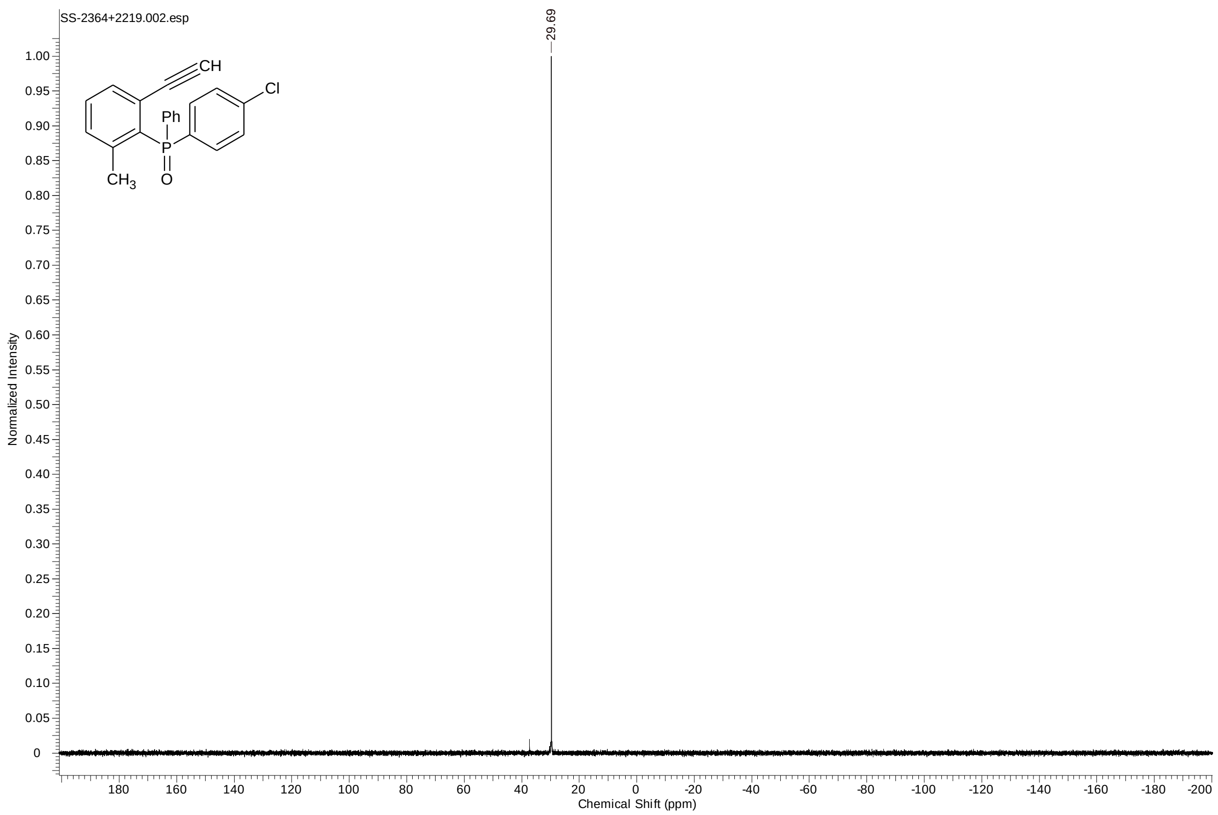

^{31}P NMR spectrum of (2-ethynyl-6-methylphenyl)phenyl(*p*-chlorophenyl)phosphine oxide (**10i**) (202 MHz, $CDCl_3$)

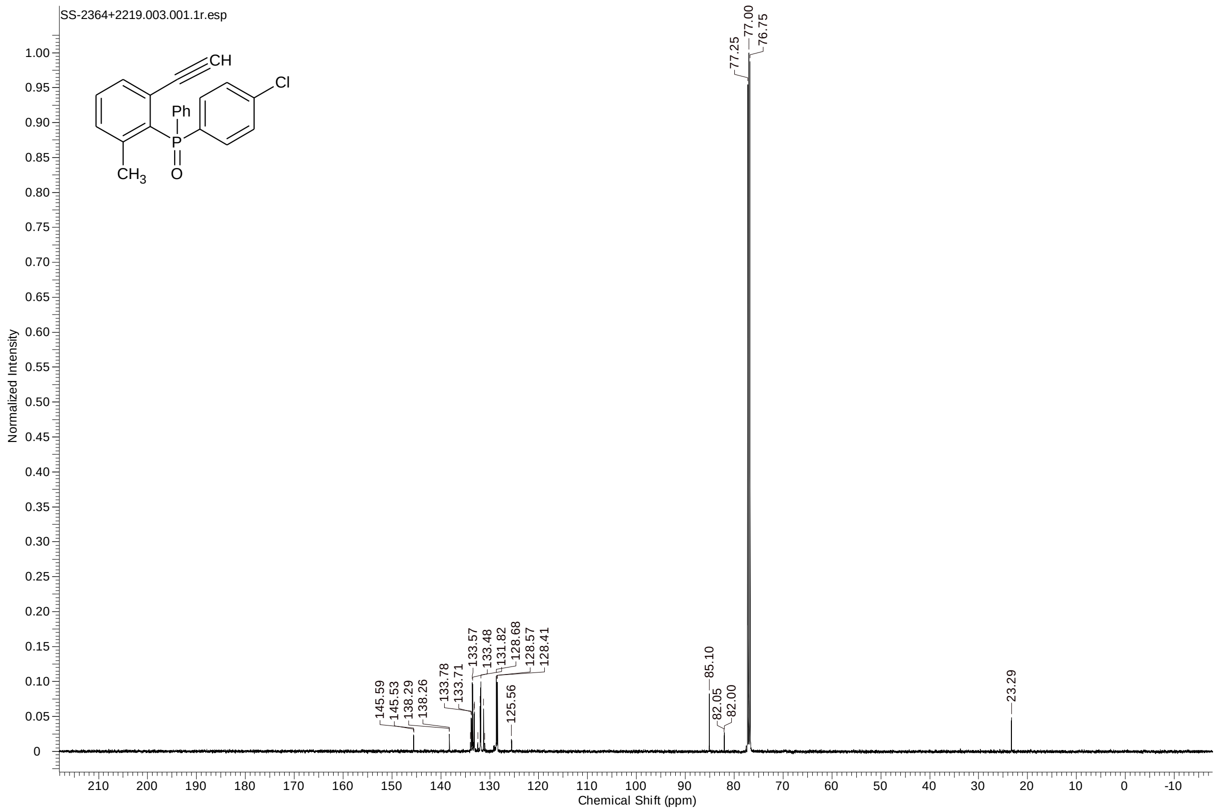

$^{13}C\{^1H\}$ NMR spectrum of (2-ethynyl-6-methylphenyl)phenyl(*p*-chlorophenyl)phosphine oxide (**10i**) (125 MHz, $CDCl_3$)

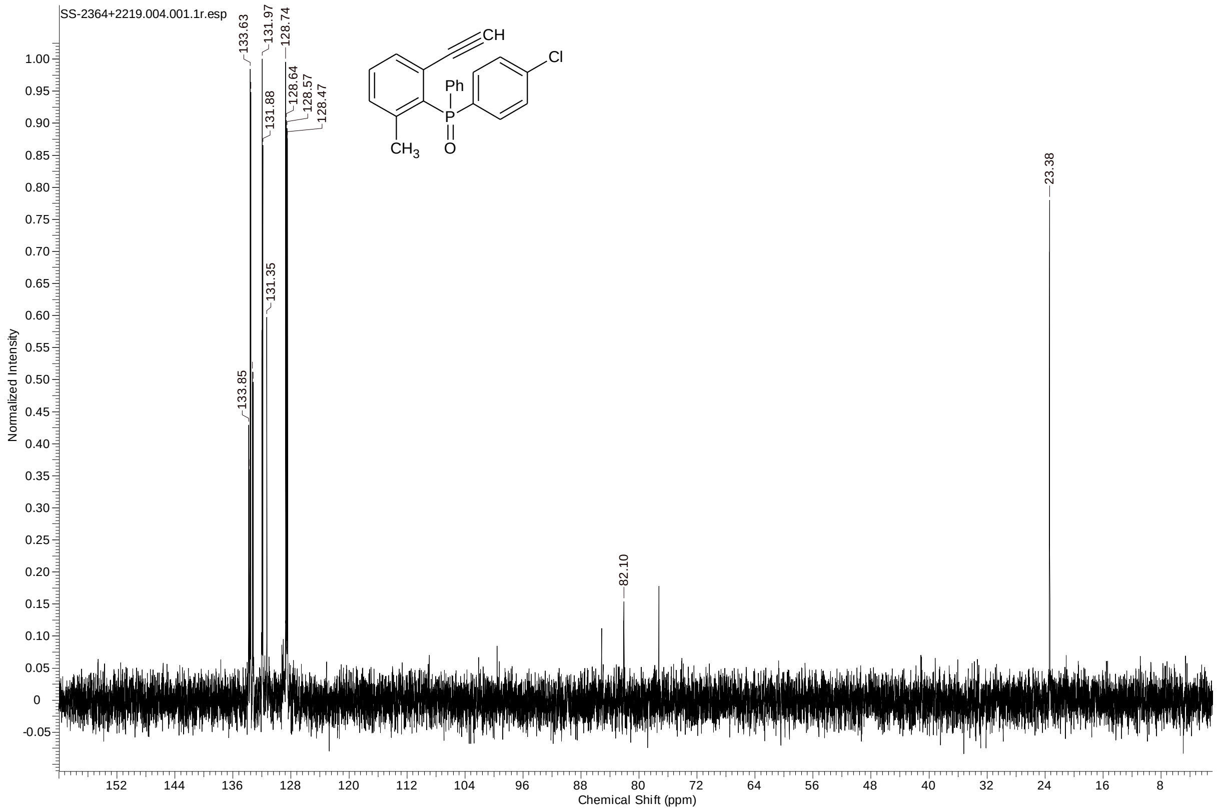

DEPT 135 NMR spectrum of (2-ethynyl-6-methylphenyl)phenyl(*p*-chlorophenyl)phosphine oxide (**10i**) (125 MHz, $CDCl_3$)

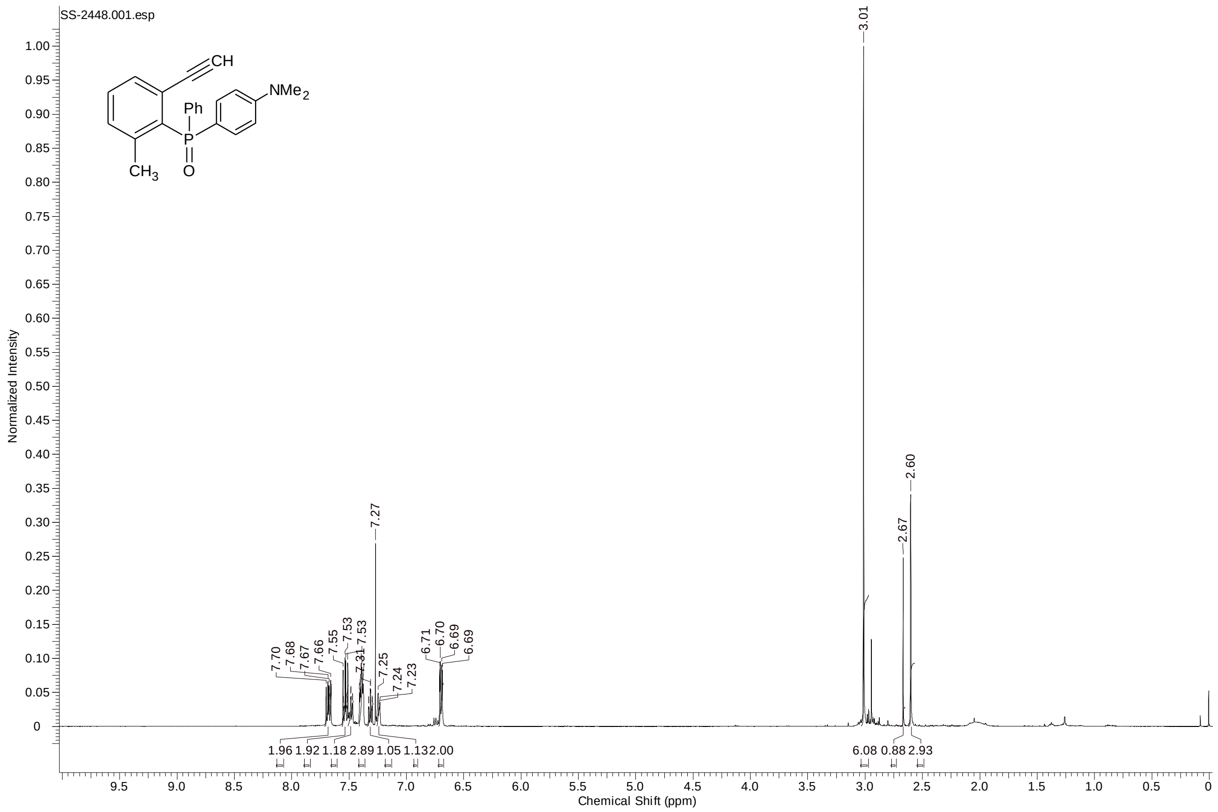

^{1}H NMR spectrum of (2-ethynylphenyl)phenyl(*p-N,N*-dimethylaminophenyl)phosphine oxide (**10k**) (500 MHz, $CDCl_3$)

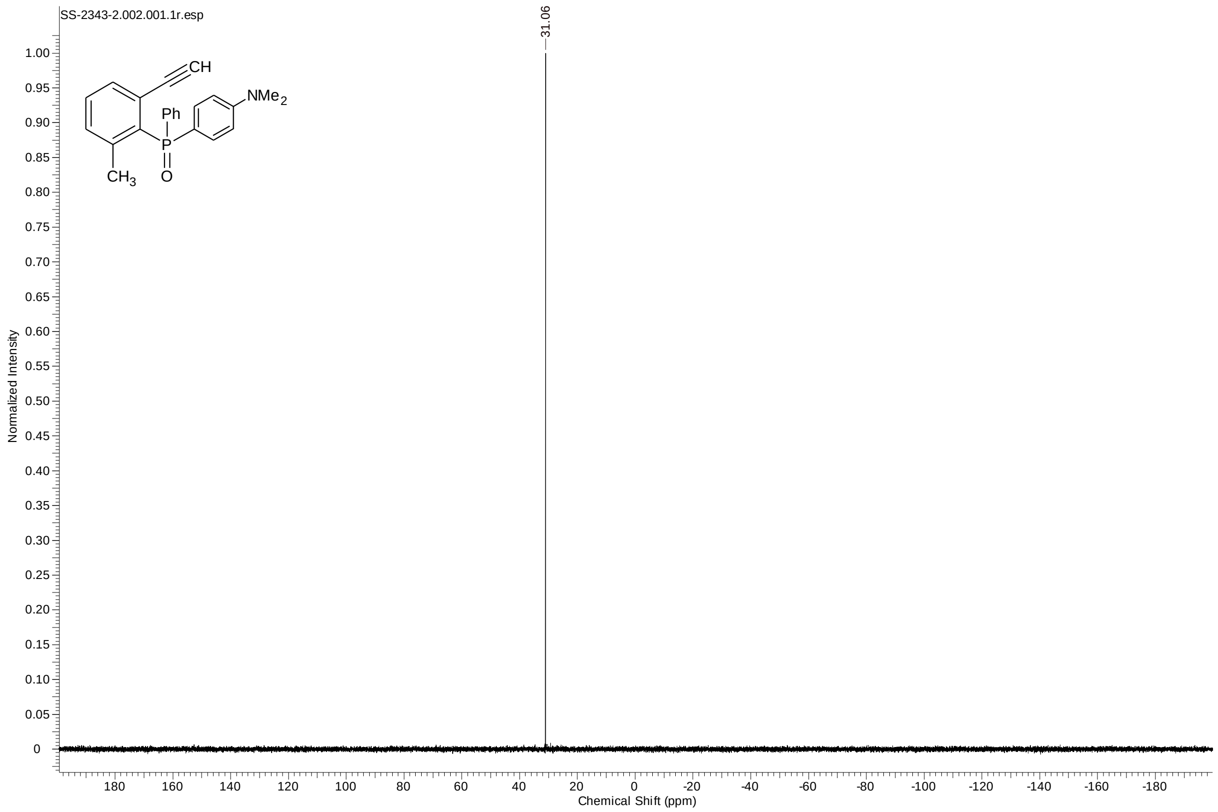

^{31}P NMR spectrum of (2-ethynylphenyl)phenyl(*p-N,N*-dimethylaminophenyl)phosphine oxide (**10k**) (202 MHz, $CDCl_3$)

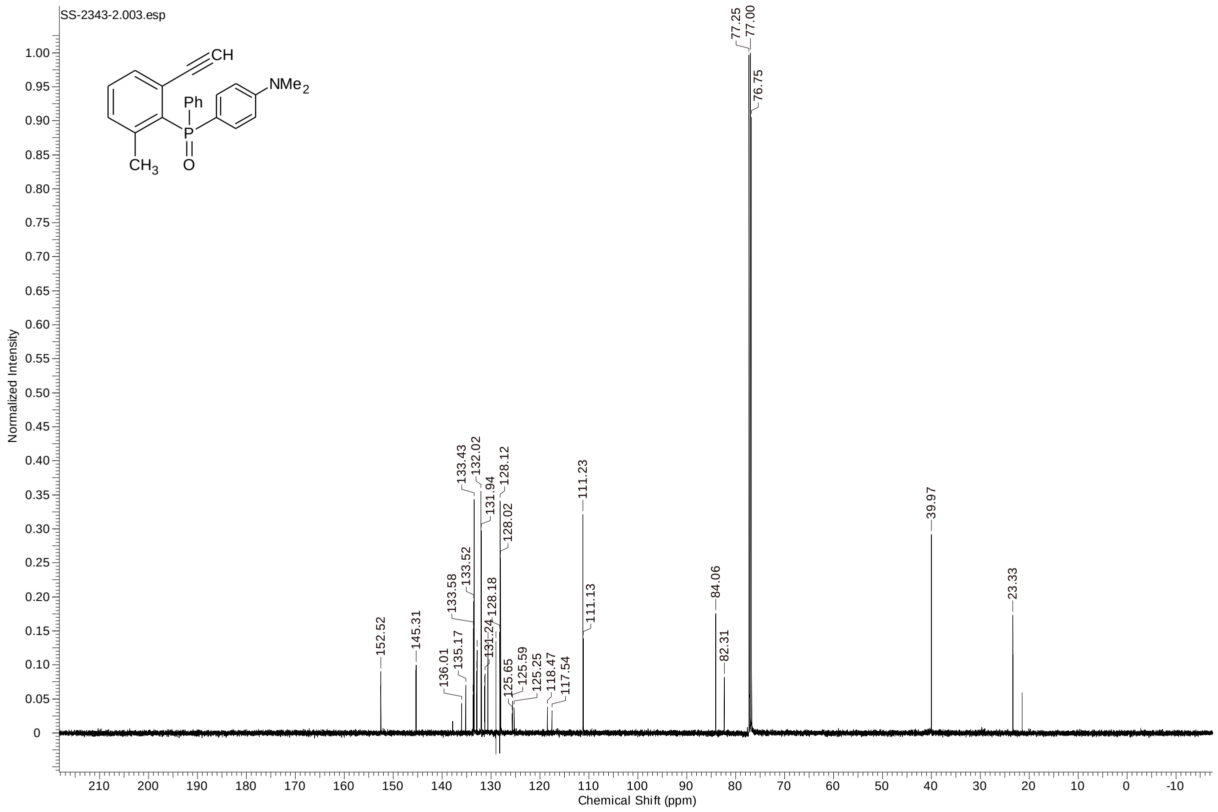

$^{13}C\{^1H\}$ NMR spectrum of (2-ethynylphenyl)phenyl(*p-N,N*-dimethylaminophenyl)phosphine oxide (**10k**) (125 MHz, $CDCl_3$)

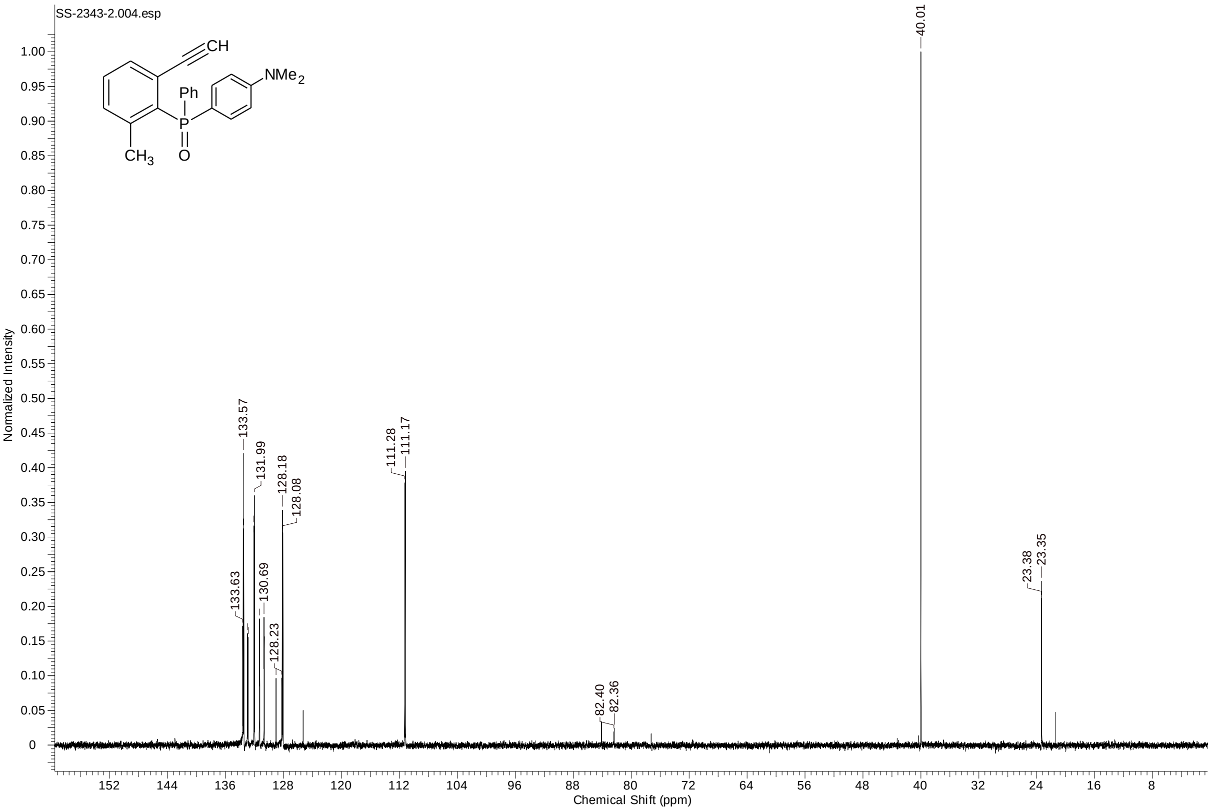

DEPT 135 NMR spectrum of (2-ethynylphenyl)phenyl(*p-N,N*-dimethylaminophenyl)phosphine oxide (**10k**) (125 MHz, $CDCl_3$)

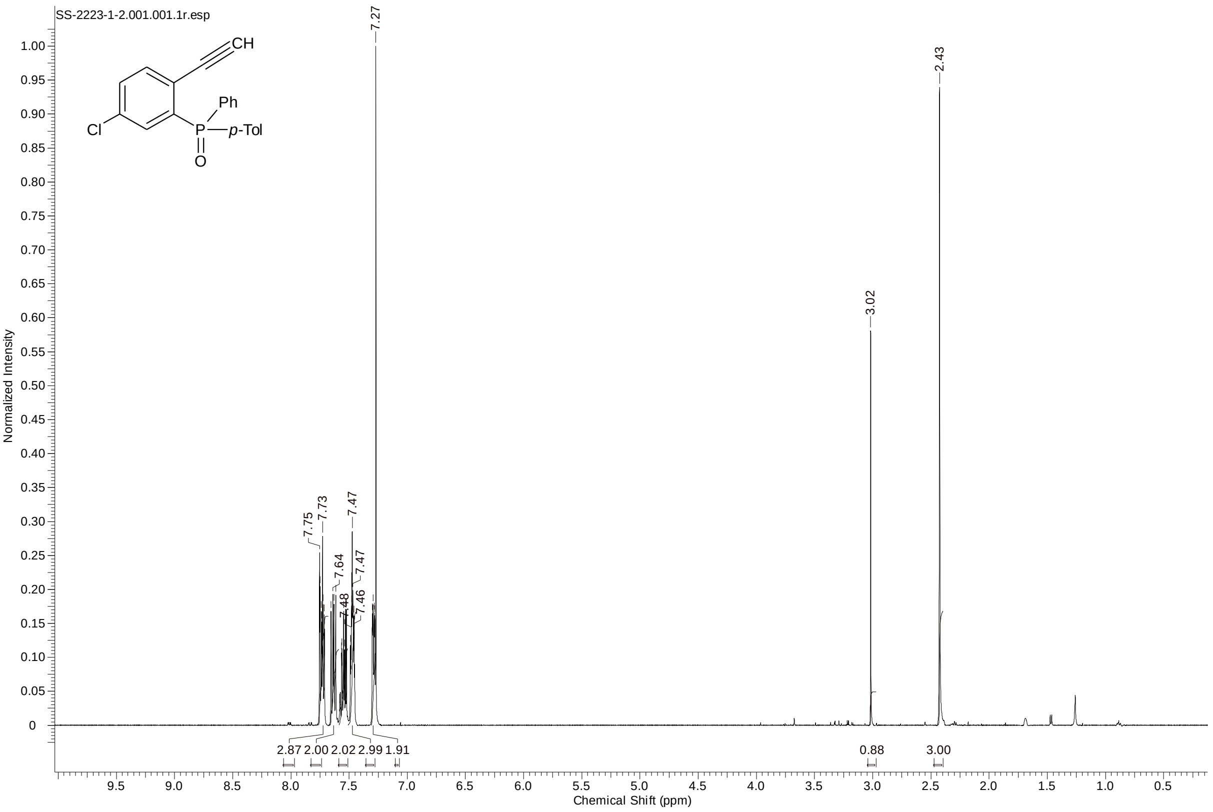

^{1}H NMR spectrum of (5-chloro-2-ethynylphenyl)phenyl(*p*-tolyl)phosphine oxide (**11a**) (500 MHz, $CDCl_3$)

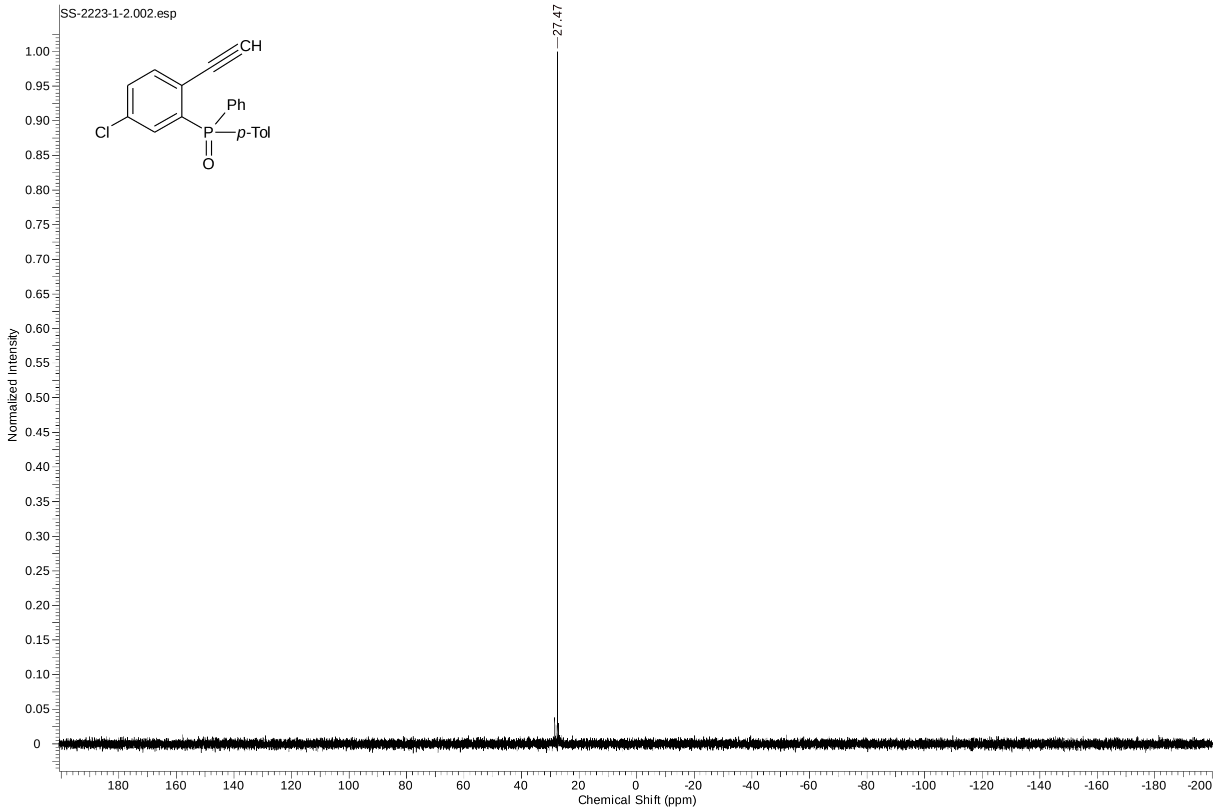

^{31}P NMR spectrum of (5-chloro-2-ethynylphenyl)phenyl(*p*-tolyl)phosphine oxide (**11a**) (202 MHz, $CDCl_3$)

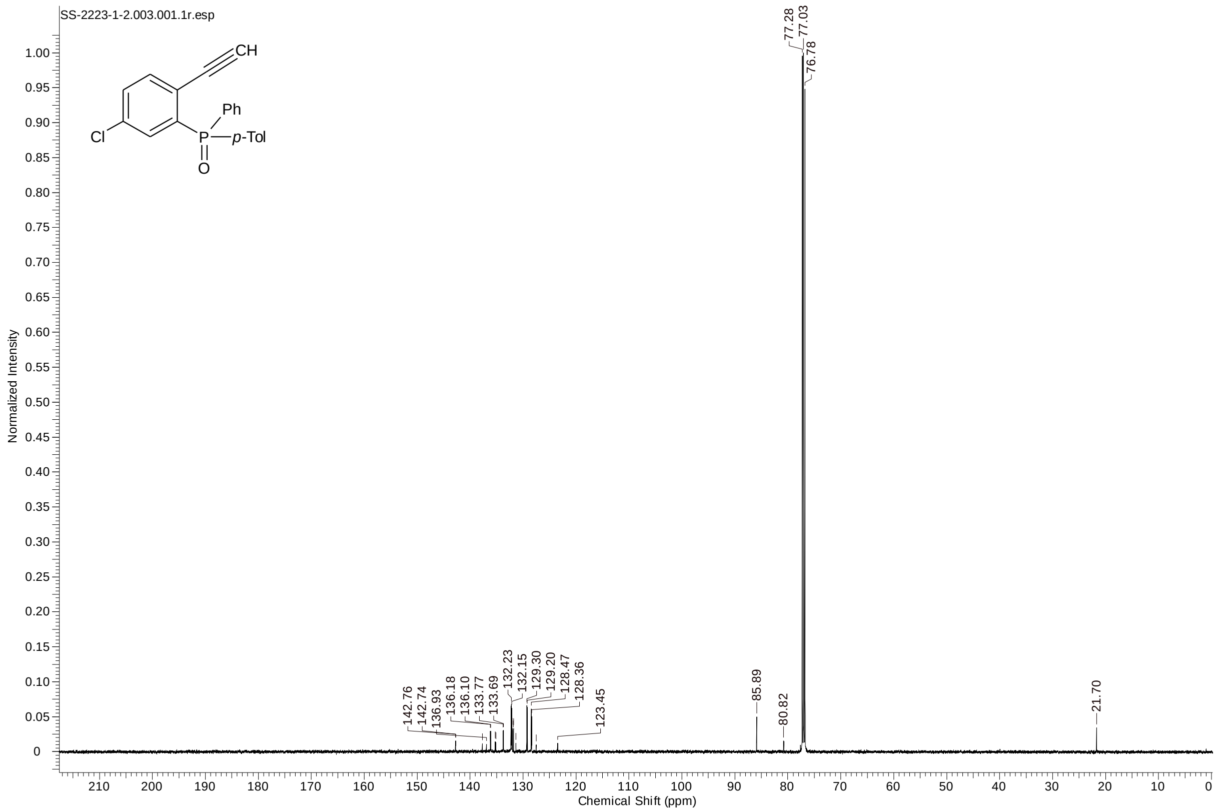

^{13}C{^{1}H} NMR spectrum of (5-chloro-2-ethynylphenyl)phenyl(*p*-tolyl)phosphine oxide (**11a**) (125 MHz, $CDCl_3$)

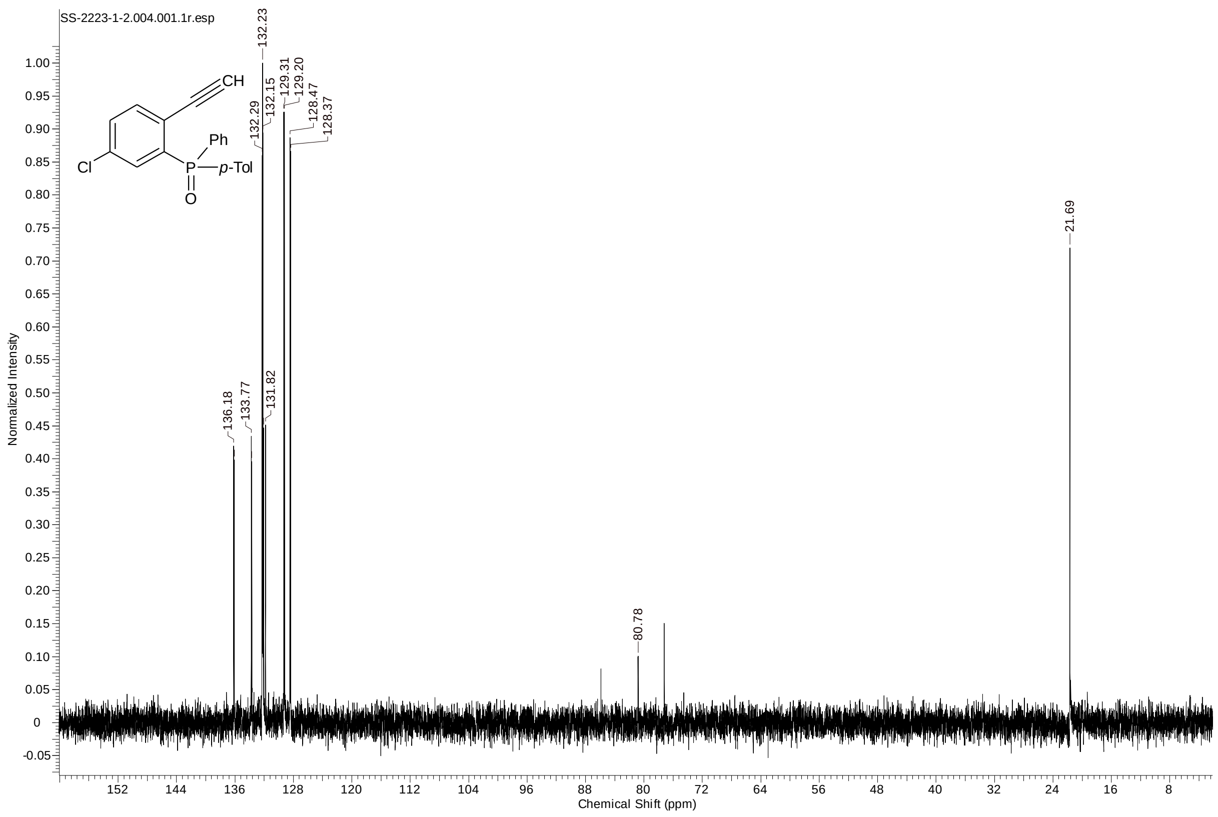

DEPT 135 NMR spectrum of (5-chloro-2-ethynylphenyl)phenyl(*p*-tolyl)phosphine oxide (**11a**) (125 MHz, $CDCl_3$)

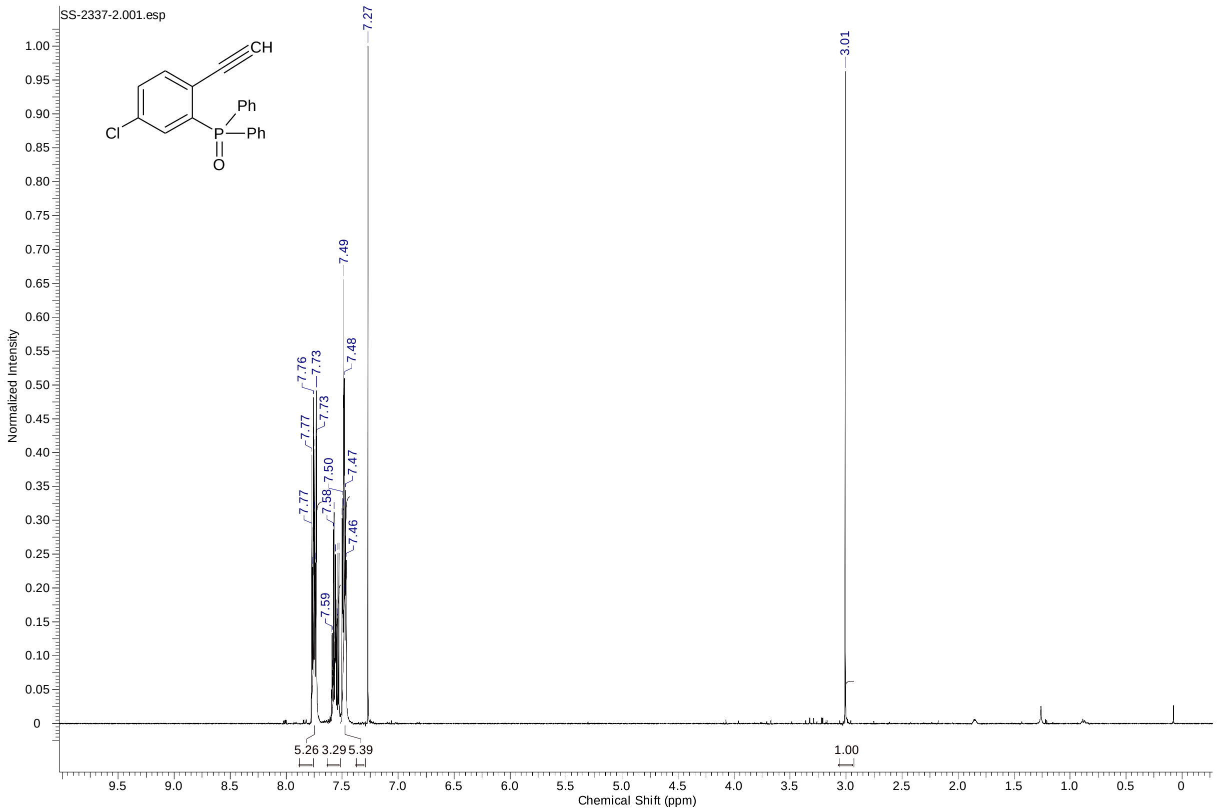

^{1}H NMR spectrum of (5-chloro-2-ethynylphenyl)diphenylphosphine oxide (**11d**) (500 MHz, $CDCl_3$)

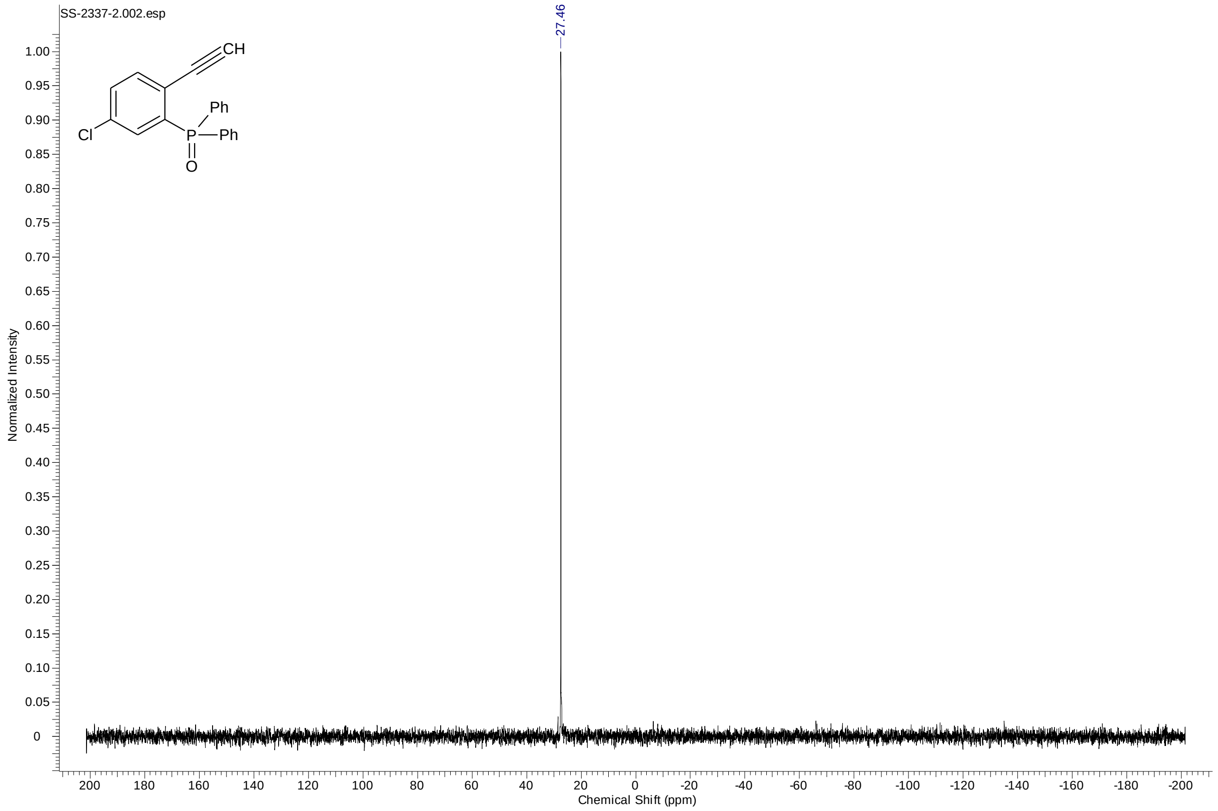

^{31}P NMR spectrum of (5-chloro-2-ethynylphenyl)diphenylphosphine oxide (**11d**) (202 MHz, $CDCl_3$)

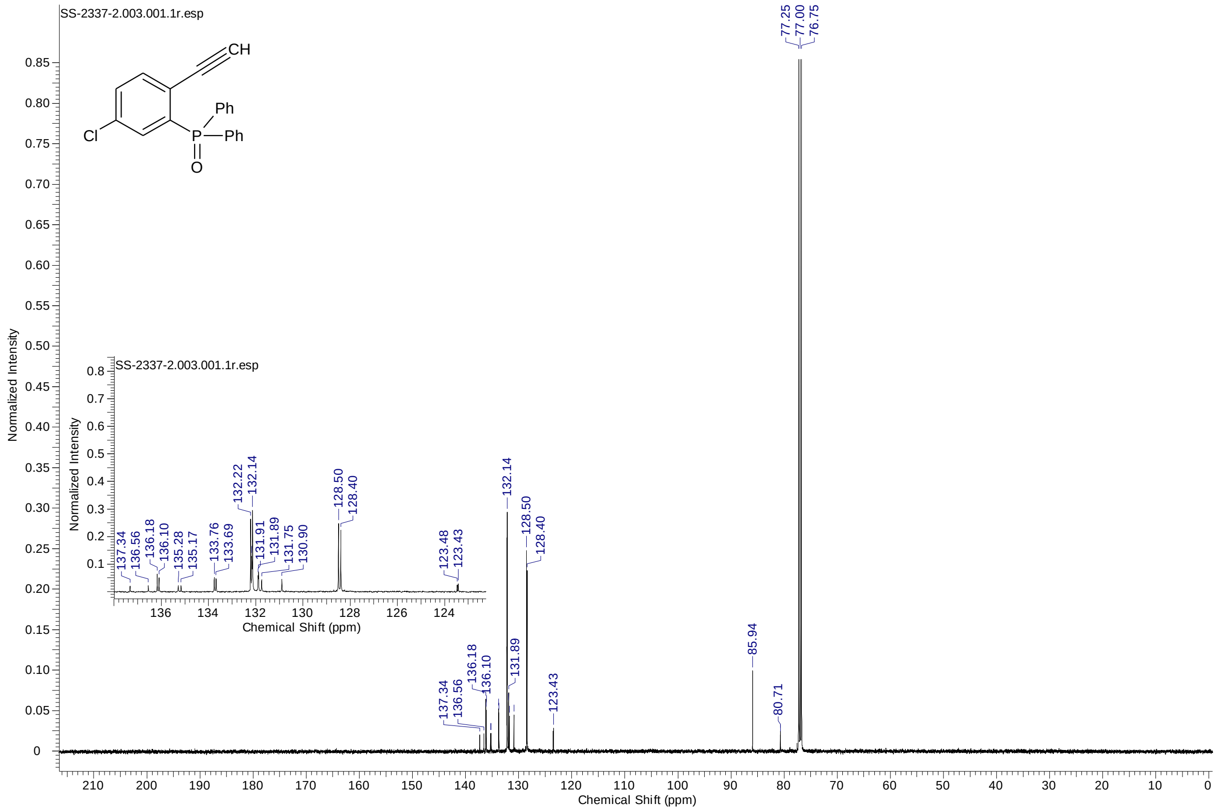

^{13}C{^{1}H} NMR spectrum of (5-chloro-2-ethynylphenyl)diphenylphosphine oxide (**11d**) (125 MHz, $CDCl_3$)

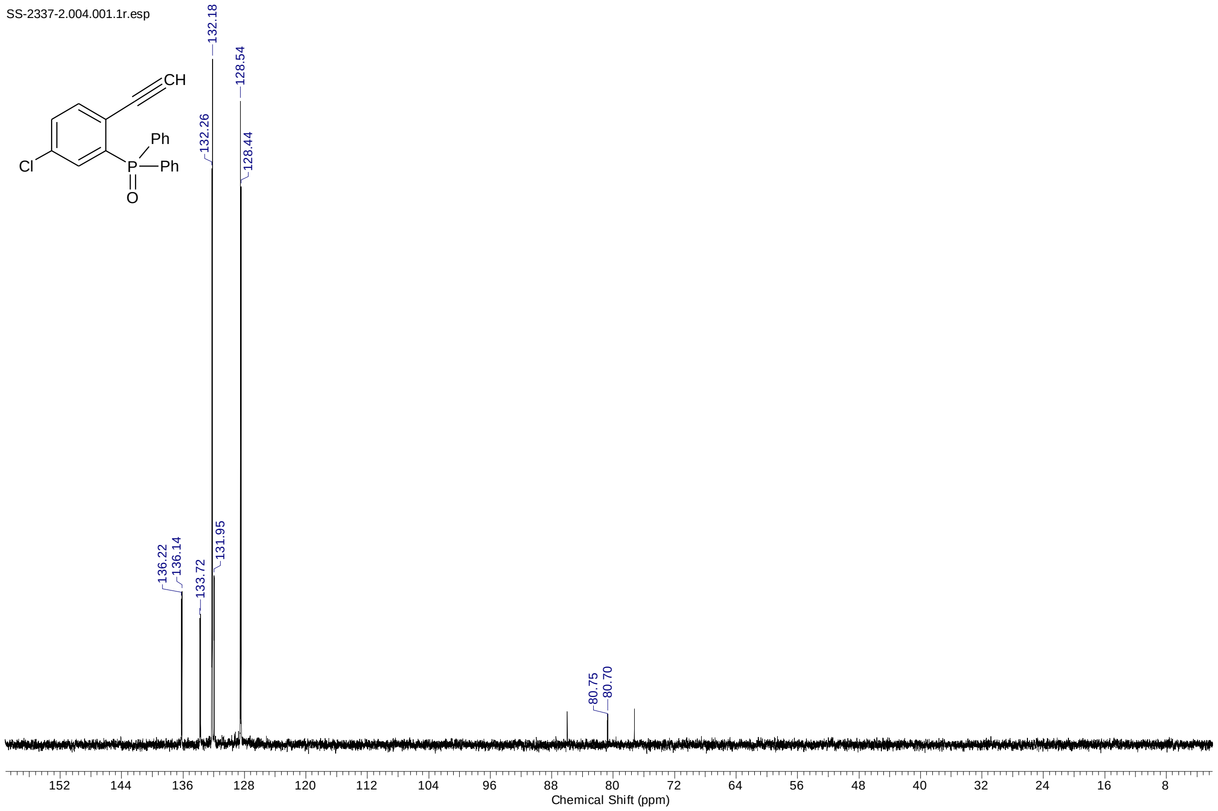

DEPT 135 NMR spectrum of (5-chloro-2-ethynylphenyl)diphenylphosphine oxide (**11d**) (125 MHz, $CDCl_3$)

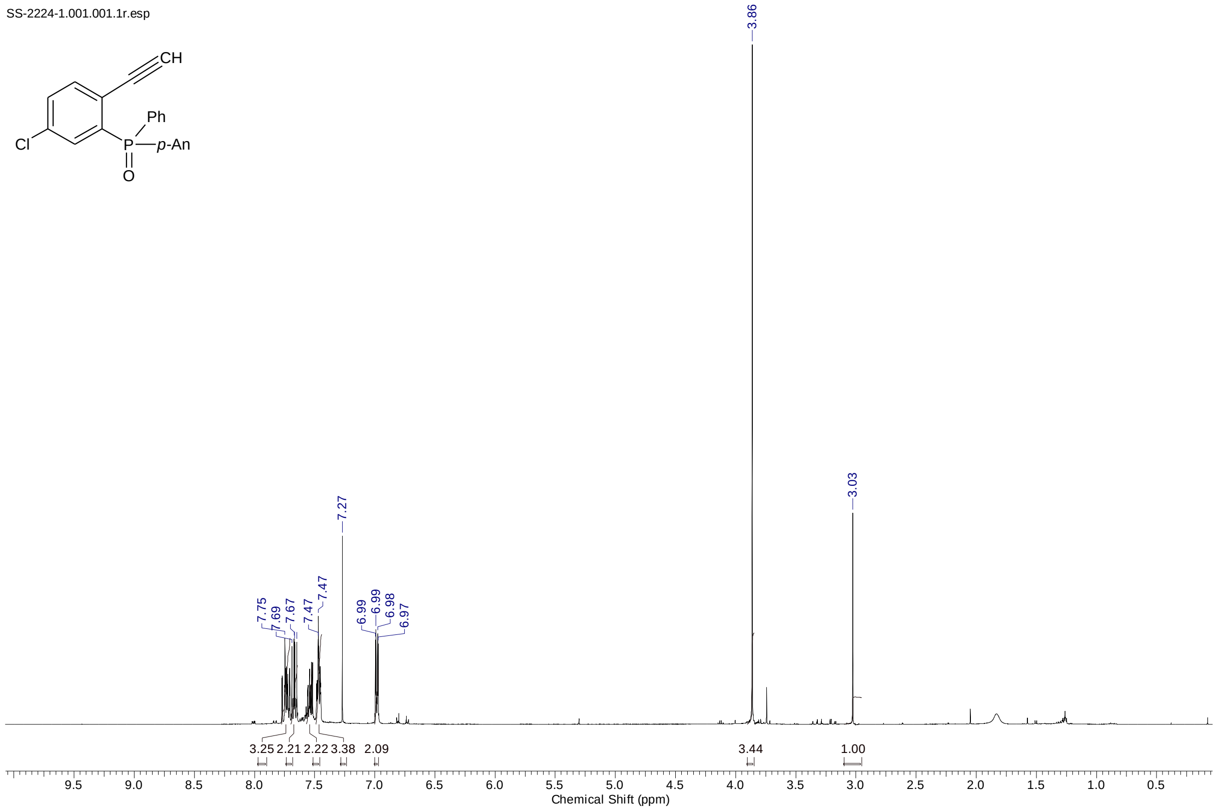

^{1}H NMR spectrum of (*p*-anisyl)(5-chloro-2-ethynylphenyl)phenylphosphine oxide (**11e**) (500 MHz, $CDCl_3$)

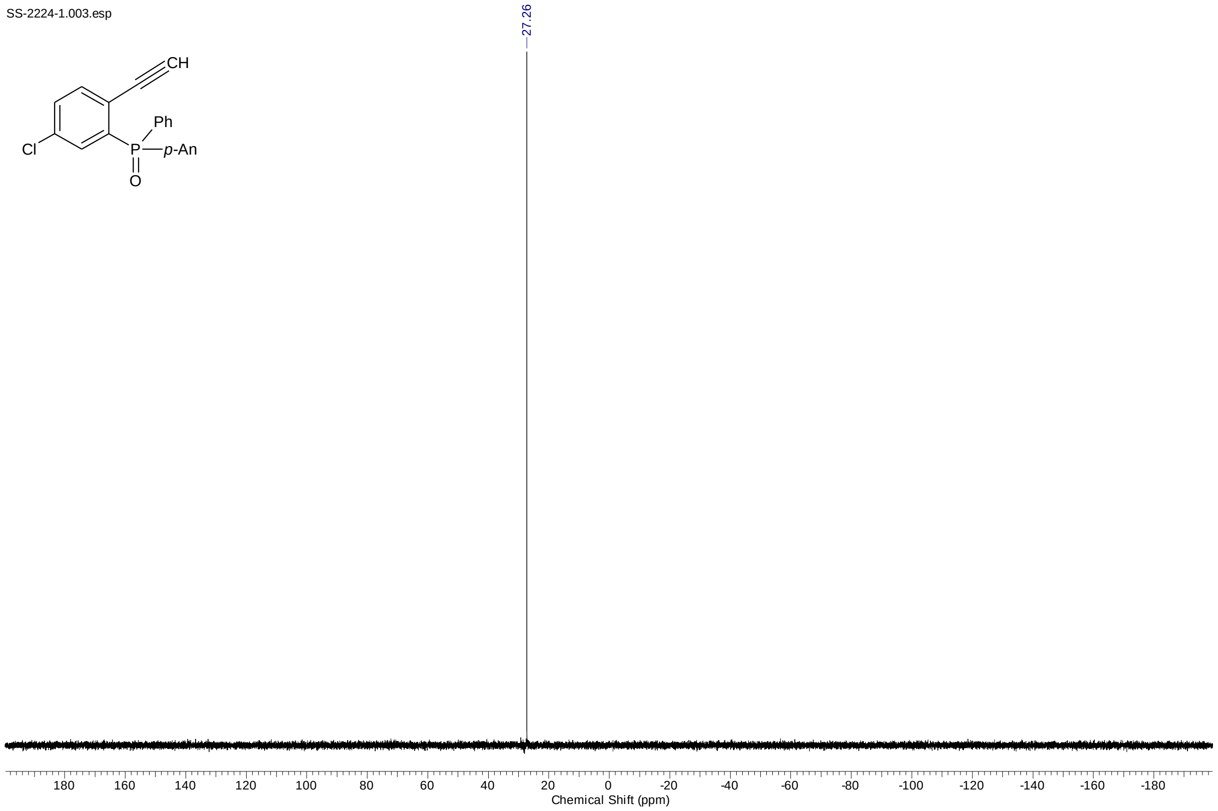

^{31}P NMR spectrum of (*p*-anisyl)(5-chloro-2-ethynylphenyl)phenylphosphine oxide (**11e**) (202 MHz, $CDCl_3$)

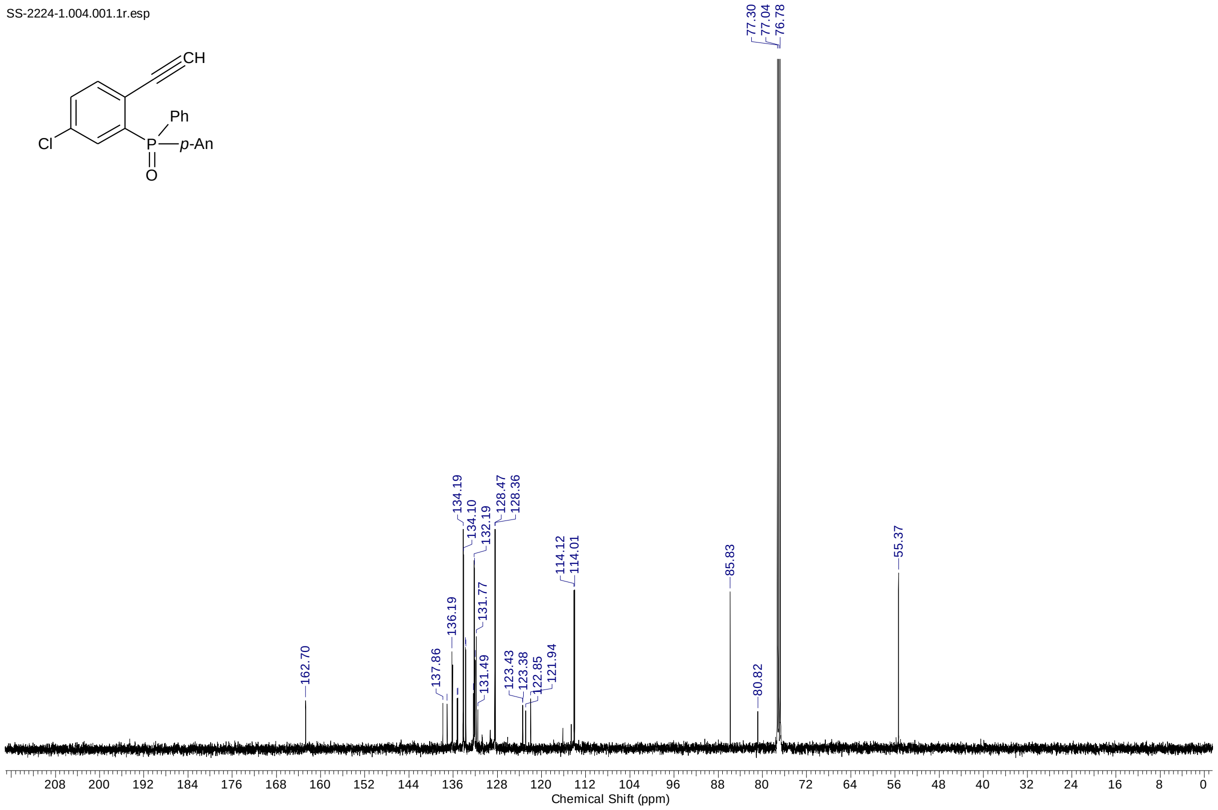

$^{13}C\{^{1}H\}$ NMR spectrum of (*p*-anisyl)(5-chloro-2-ethynylphenyl)phenylphosphine oxide (**11e**) (125 MHz, $CDCl_3$)

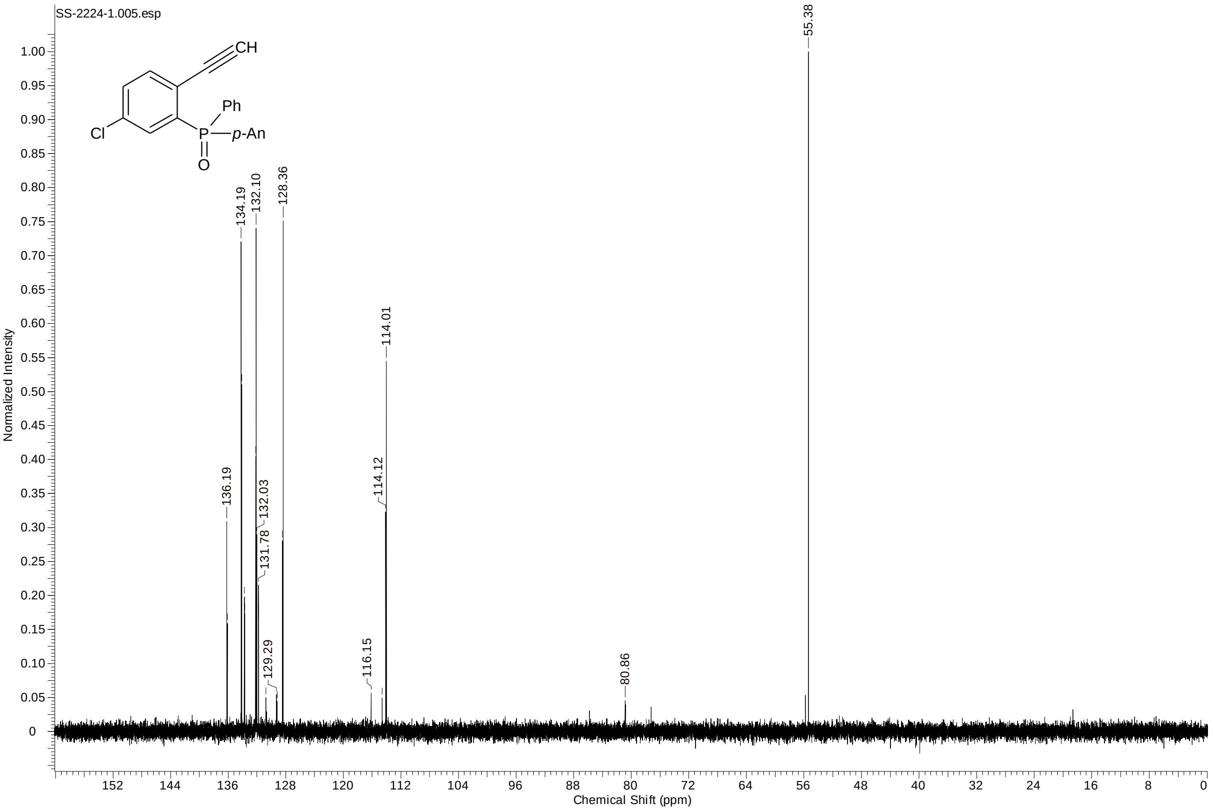

DEPT 135 NMR spectrum of (5-chloro-2-ethynylphenyl)diphenylphosphine oxide (**11e**) (125 MHz, $CDCl_3$)

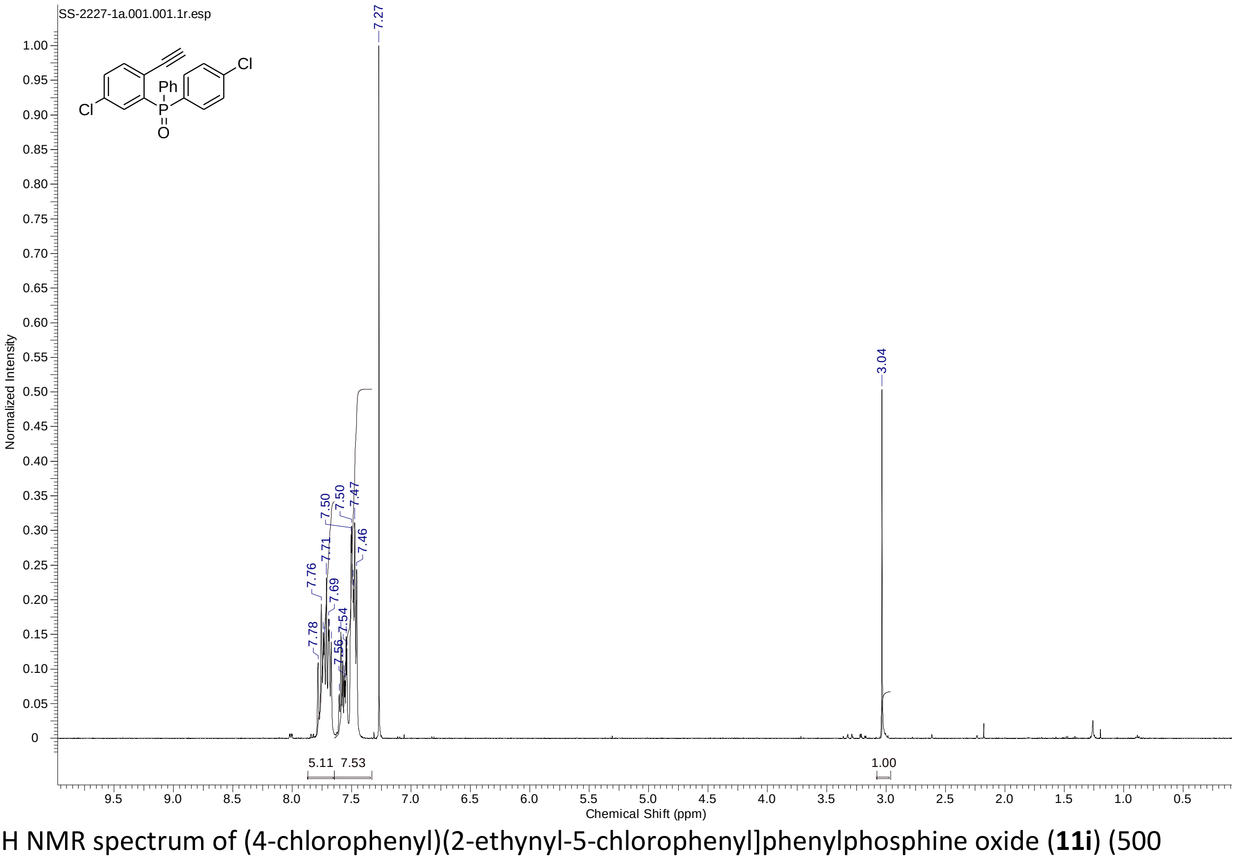

^{1}H NMR spectrum of (4-chlorophenyl)(2-ethynyl-5-chlorophenyl]phenylphosphine oxide (**11i**) (500 MHz, $CDCl_3$)

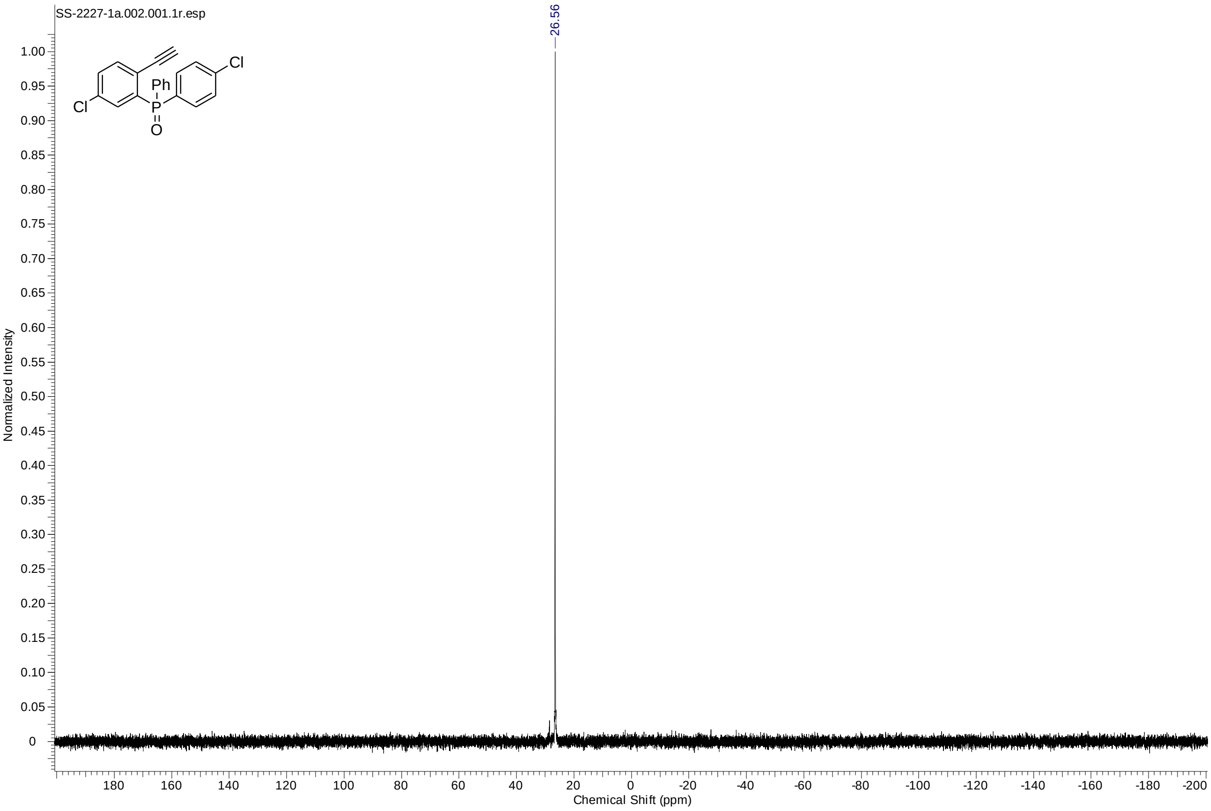

^{31}P NMR spectrum of (4-chlorophenyl)(2-ethynyl-5-chlorophenyl]phenylphosphine oxide (**11i**) (202 MHz, $CDCl_3$)

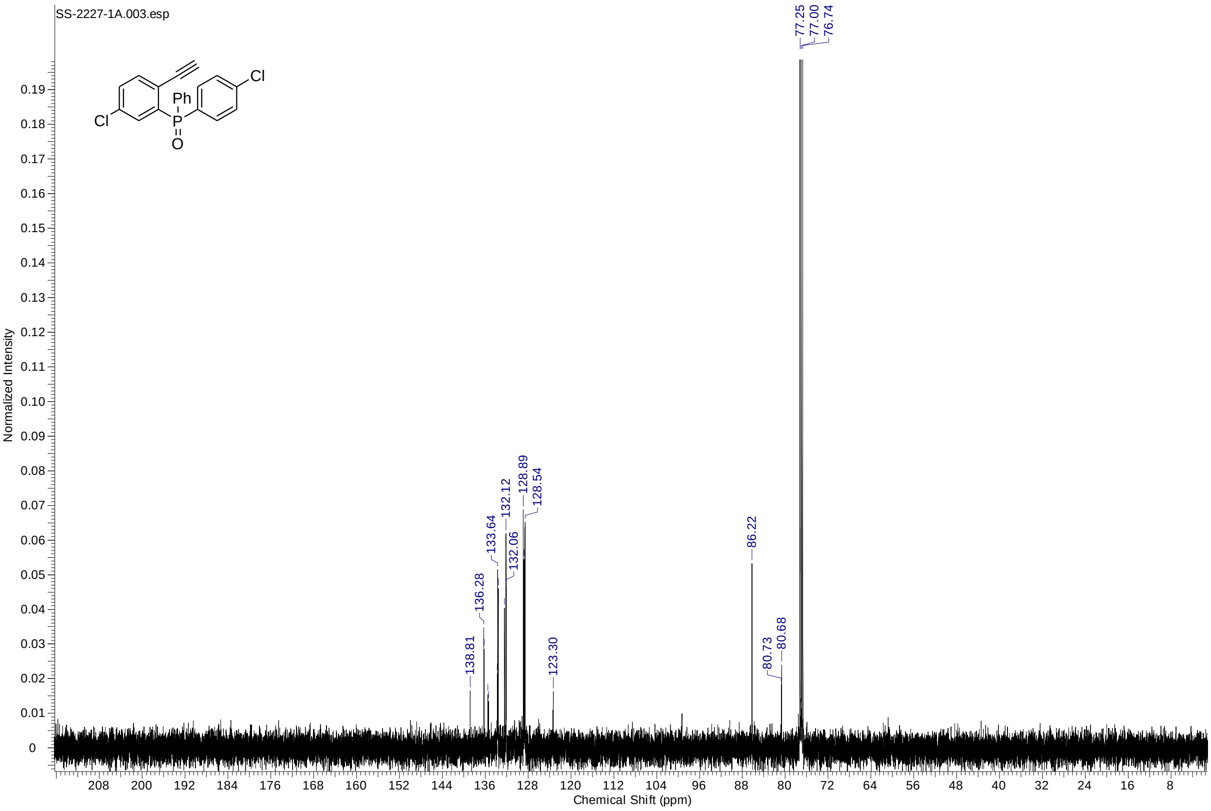

^{13}C{^{1}H} NMR spectrum of (4-chlorophenyl)(2-ethynyl-5-chlorophenyl]phenylphosphine oxide (**11i**) (125 MHz, $CDCl_3$)

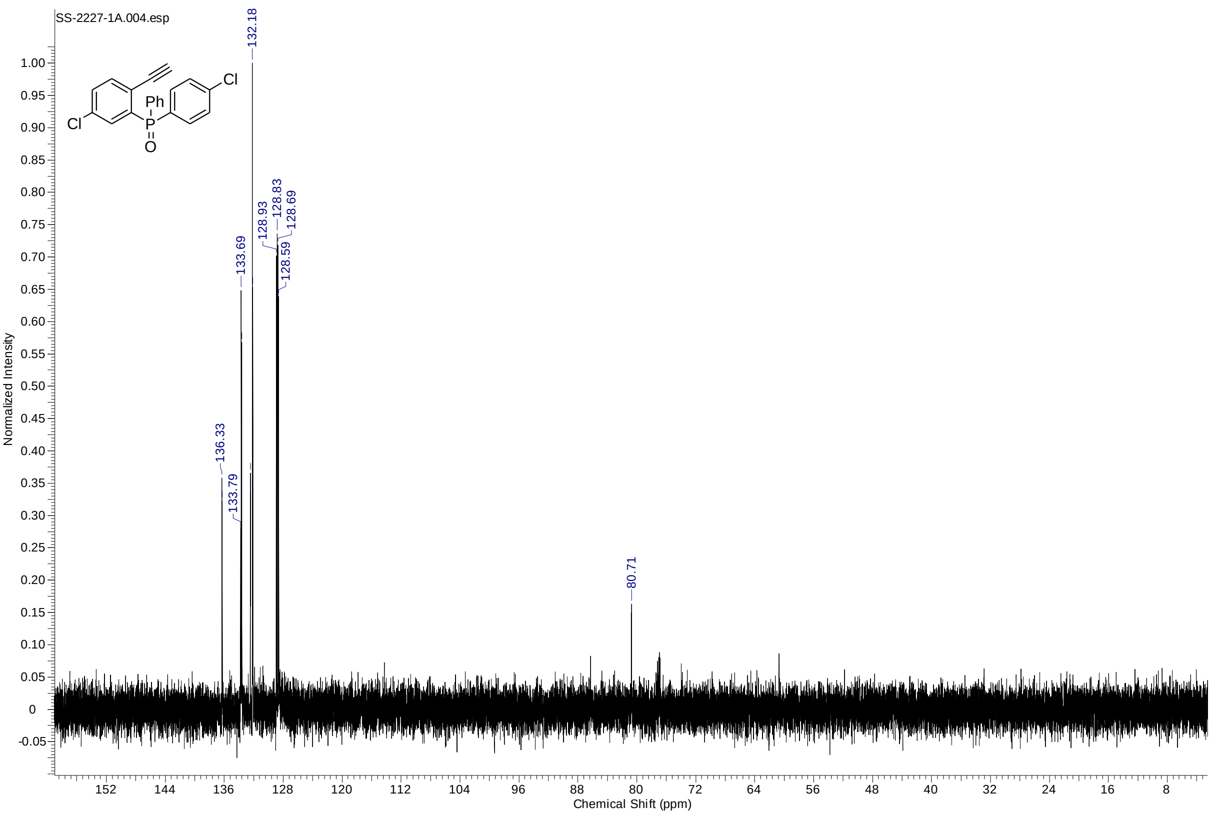

DEPT 135 NMR spectrum of (4-chlorophenyl)(2-ethynyl-5-chlorophenyl]phenylphosphine oxide (**11i**) (125 MHz, $CDCl_3$)

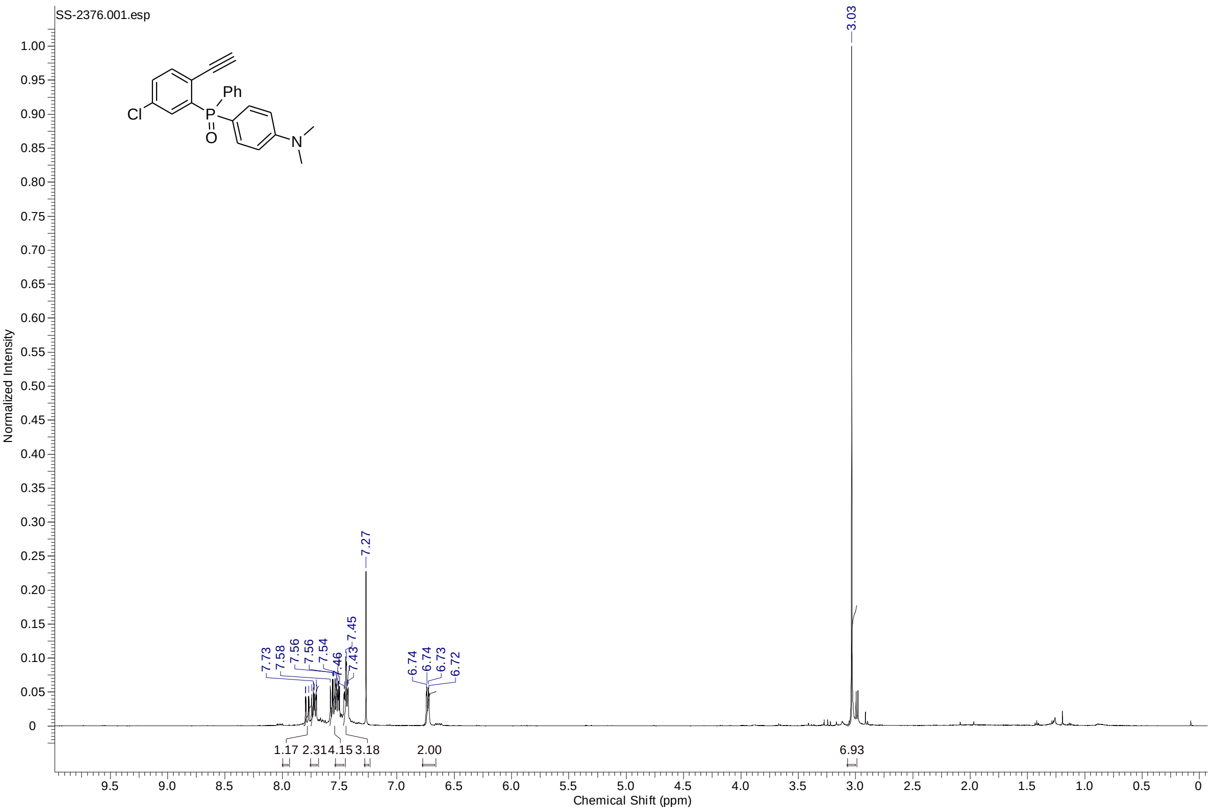

^{1}H NMR spectrum of (4-chlorophenyl)(2-ethynyl-5-chlorophenyl]phenylphosphine oxide (**11k**) (500 MHz, $CDCl_3$)

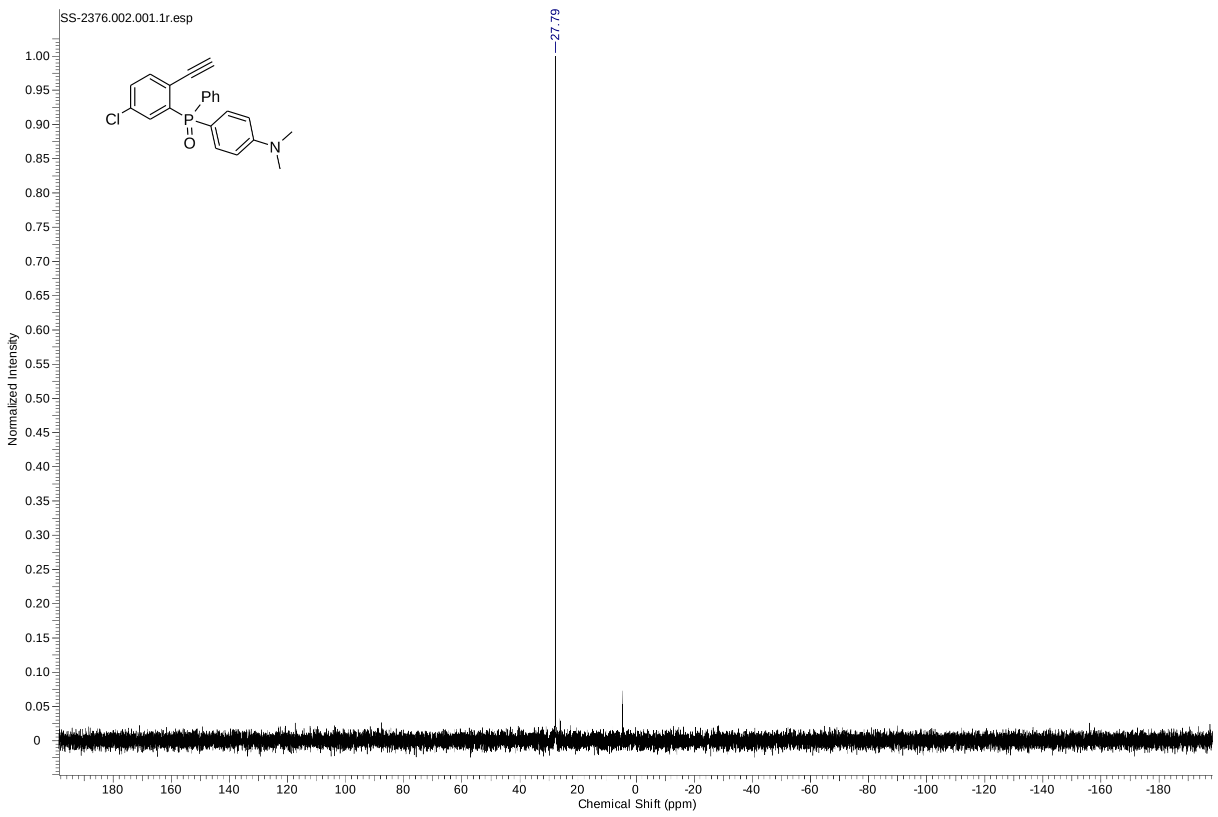

^{31}P NMR spectrum of (4-chlorophenyl)(2-ethynyl-5-chlorophenyl]phenylphosphine oxide (**11k**) (202 MHz, $CDCl_3$)

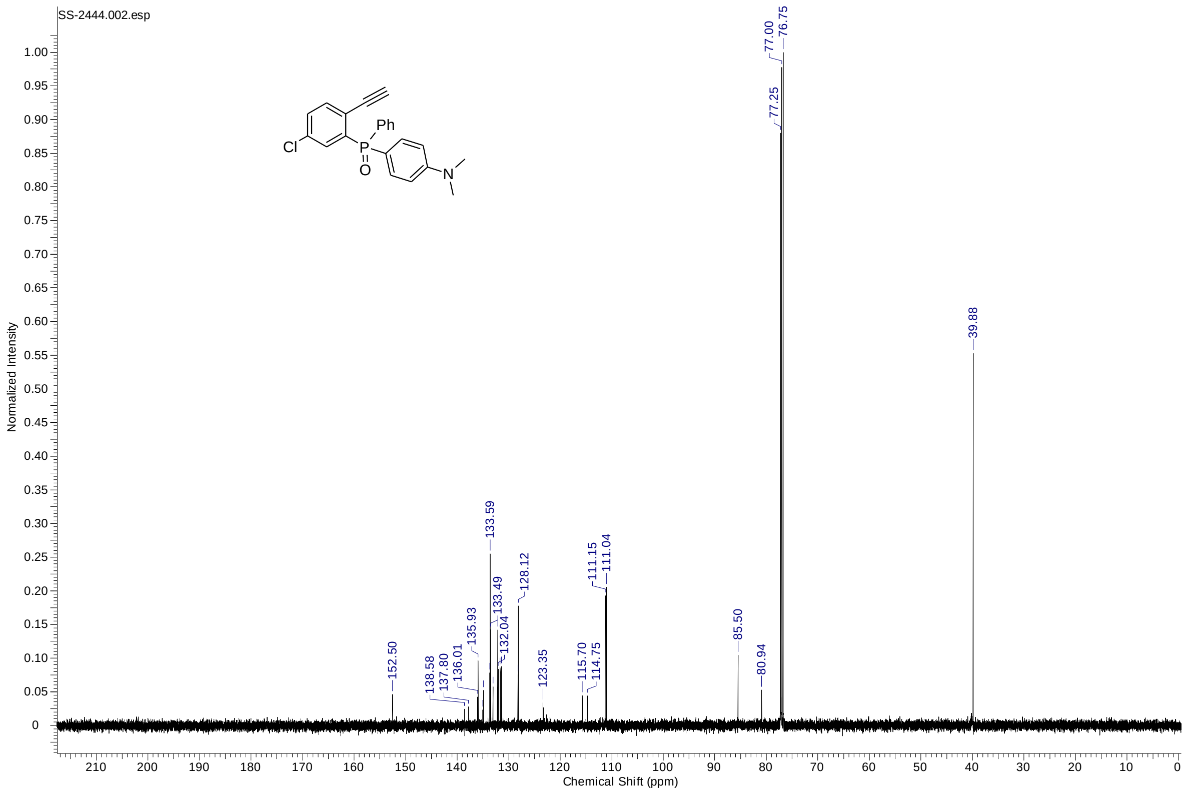

$^{13}C\{^1H\}$ NMR spectrum of (4-chlorophenyl)(2-ethynyl-5-chlorophenyl]phenylphosphine oxide (**11k**) (125 MHz, $CDCl_3$)

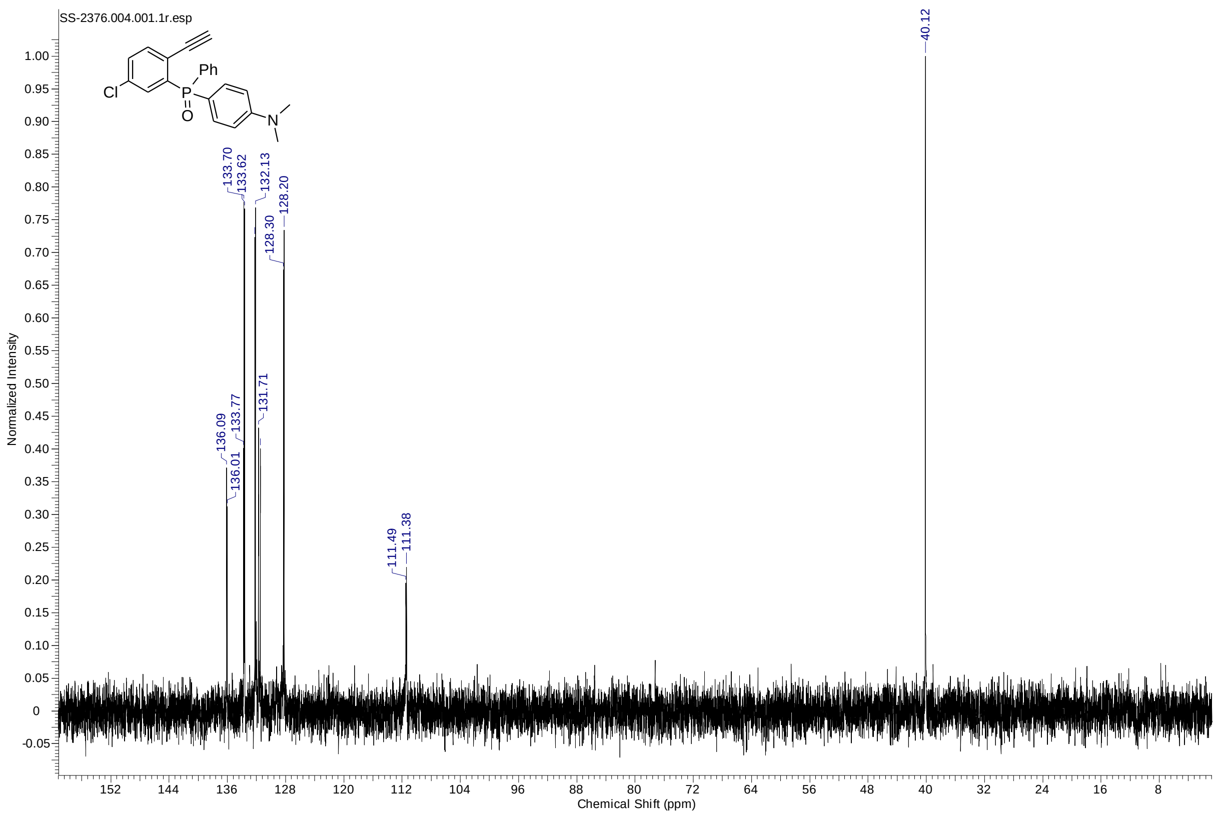

DEPT 135 NMR spectrum of (4-chlorophenyl)(2-ethynyl-5-chlorophenyl]phenylphosphine oxide (**11k**) (125 MHz, $CDCl_3$)

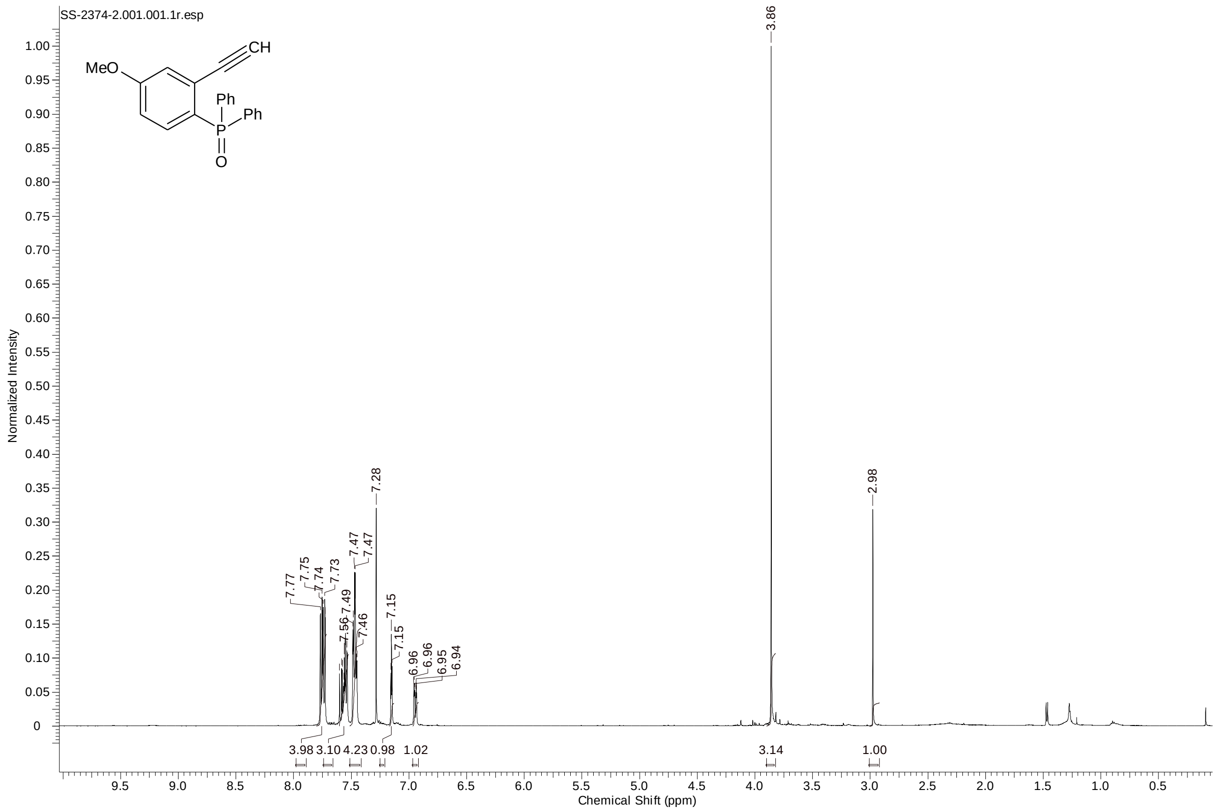

^{1}H NMR spectrum of (2-ethynyl-4-methoxyphenyl]diphenylphosphine oxide (**12d**) (500 MHz, $CDCl_3$)

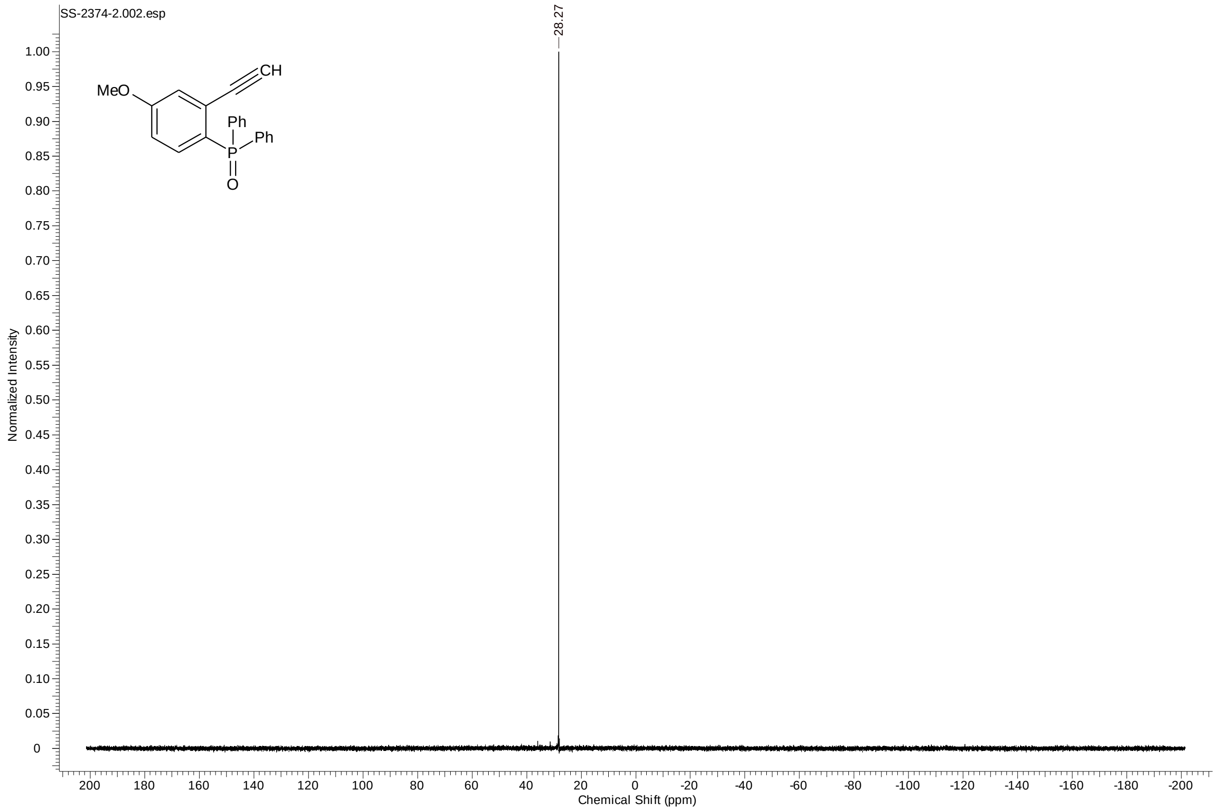

^{31}P NMR spectrum of (2-ethynyl-4-methoxyphenyl]diphenylphosphine oxide (**12d**) (202 MHz, $CDCl_3$)

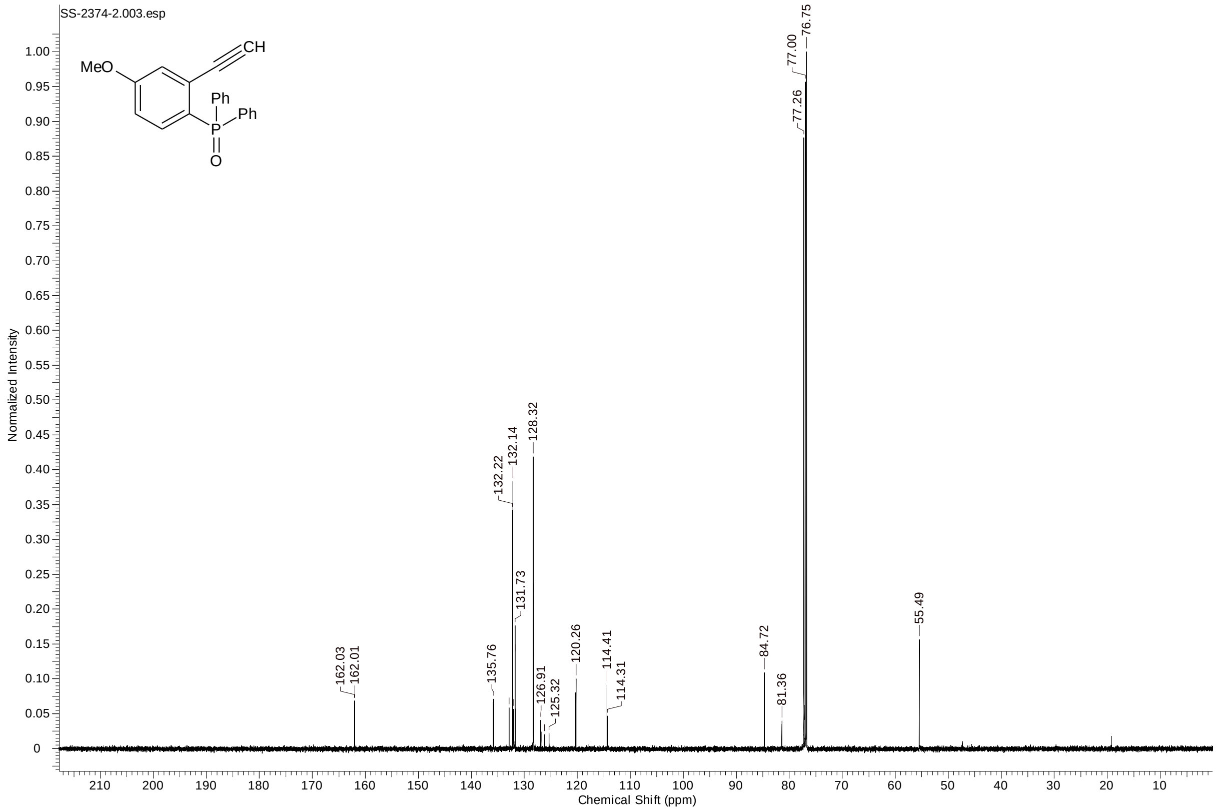

$^{13}C\{^{1}H\}$ NMR spectrum of (2-ethynyl-4-methoxyphenyl]diphenylphosphine oxide (**12d**) (125 MHz, $CDCl_3$)

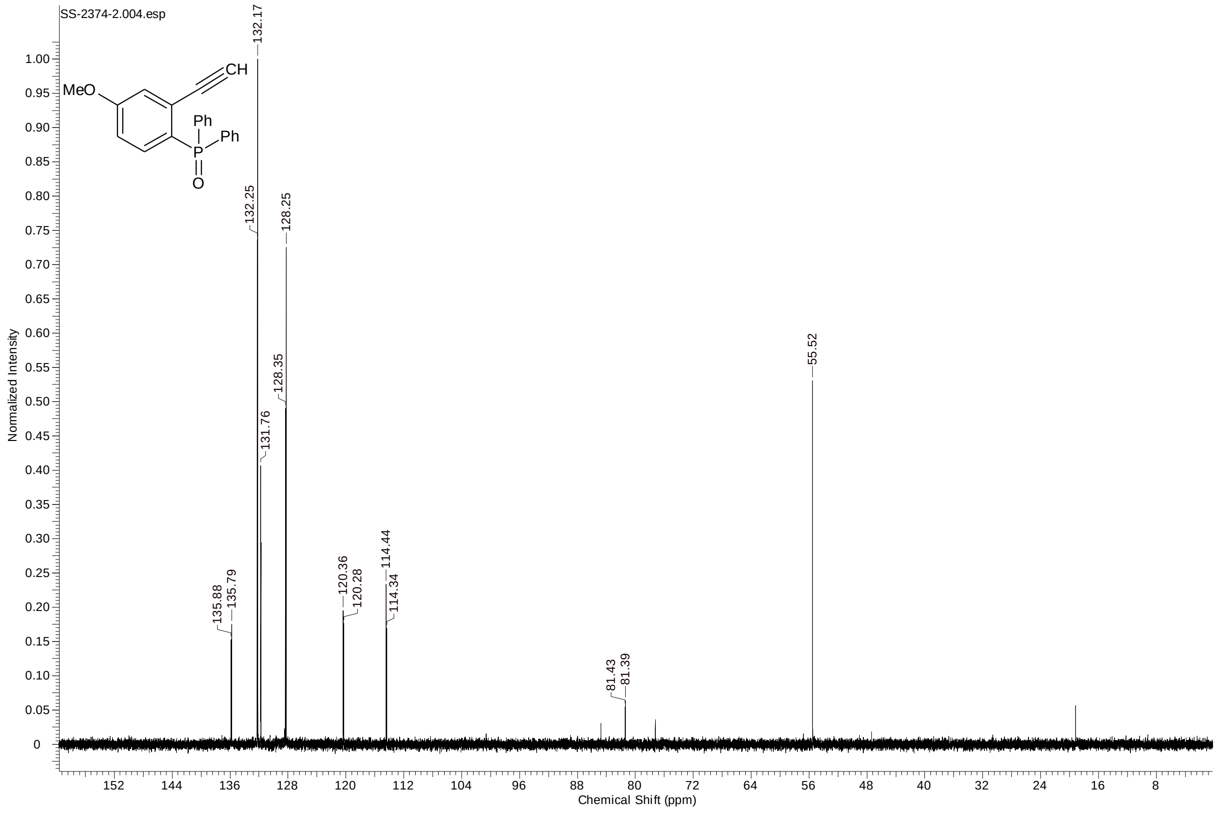

DEPT 135 NMR spectrum of (2-ethynyl-4-methoxyphenyl]diphenylphosphine oxide (**12d**) (125 MHz, $CDCl_3$)

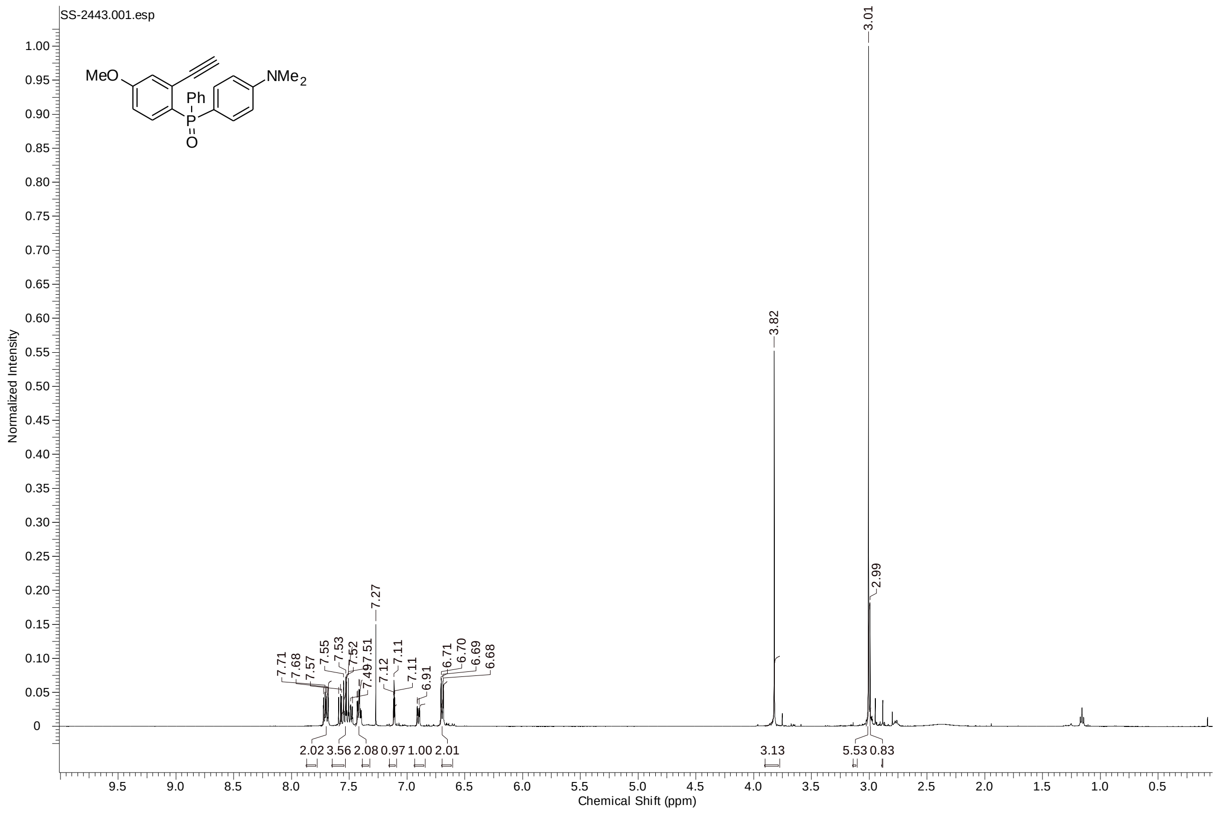

^{1}H NMR spectrum of (2-ethynyl-4-methoxyphenyl](4-dimethylaminophenyl)phenylphosphine oxide (**12k**) (500 MHz, $CDCl_3$)

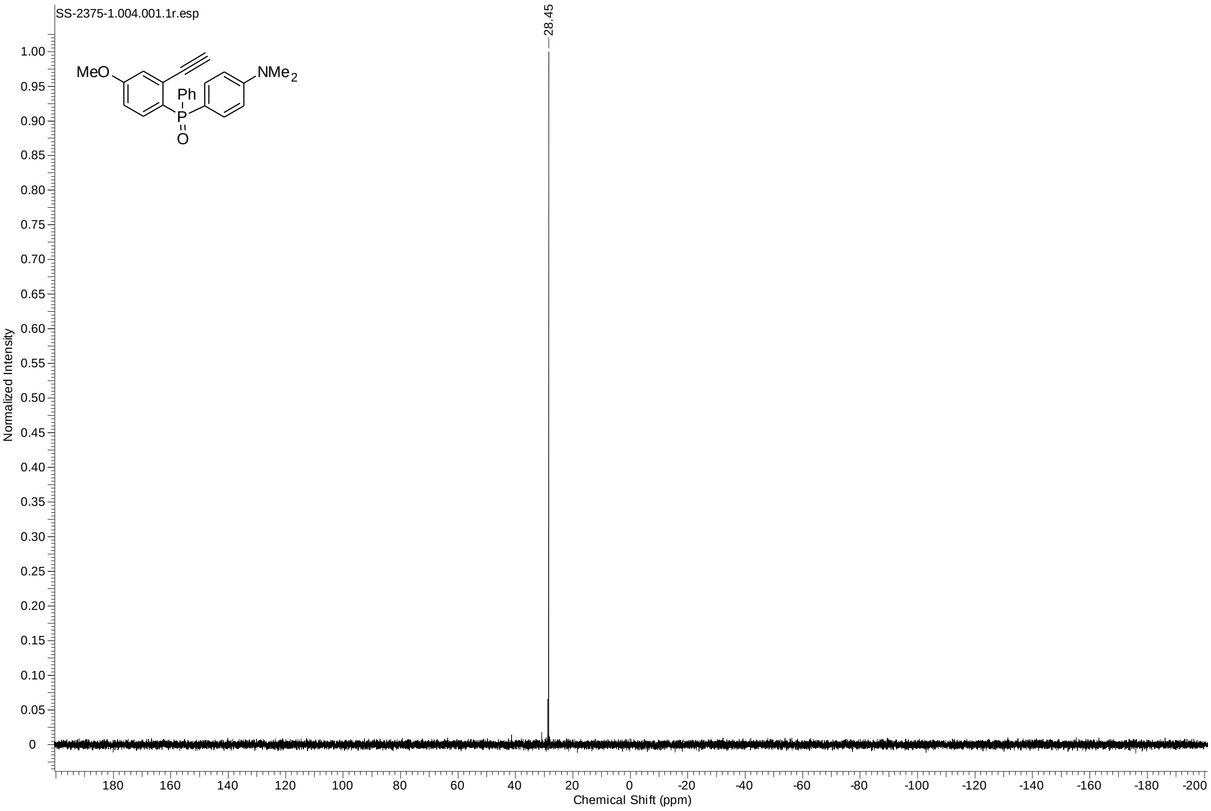

^{31}P NMR spectrum of (2-ethynyl-4-methoxyphenyl](4-dimethylaminophenyl)phenylphosphine oxide (**12k**) (202 MHz, $CDCl_3$)

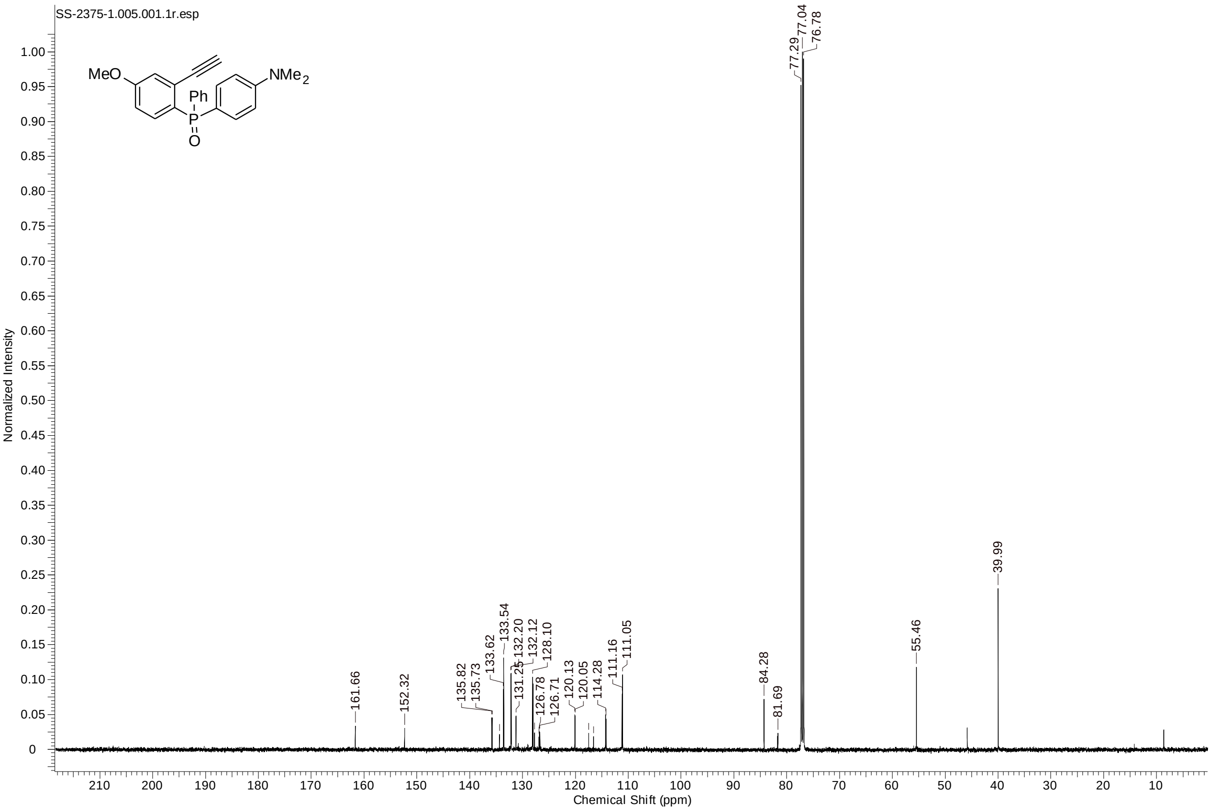

$^{13}C\{^1H\}$ NMR spectrum of (2-ethynyl-4-methoxyphenyl](4-dimethylaminophenyl)phenylphosphine oxide (**12k**) (125 MHz, $CDCl_3$)

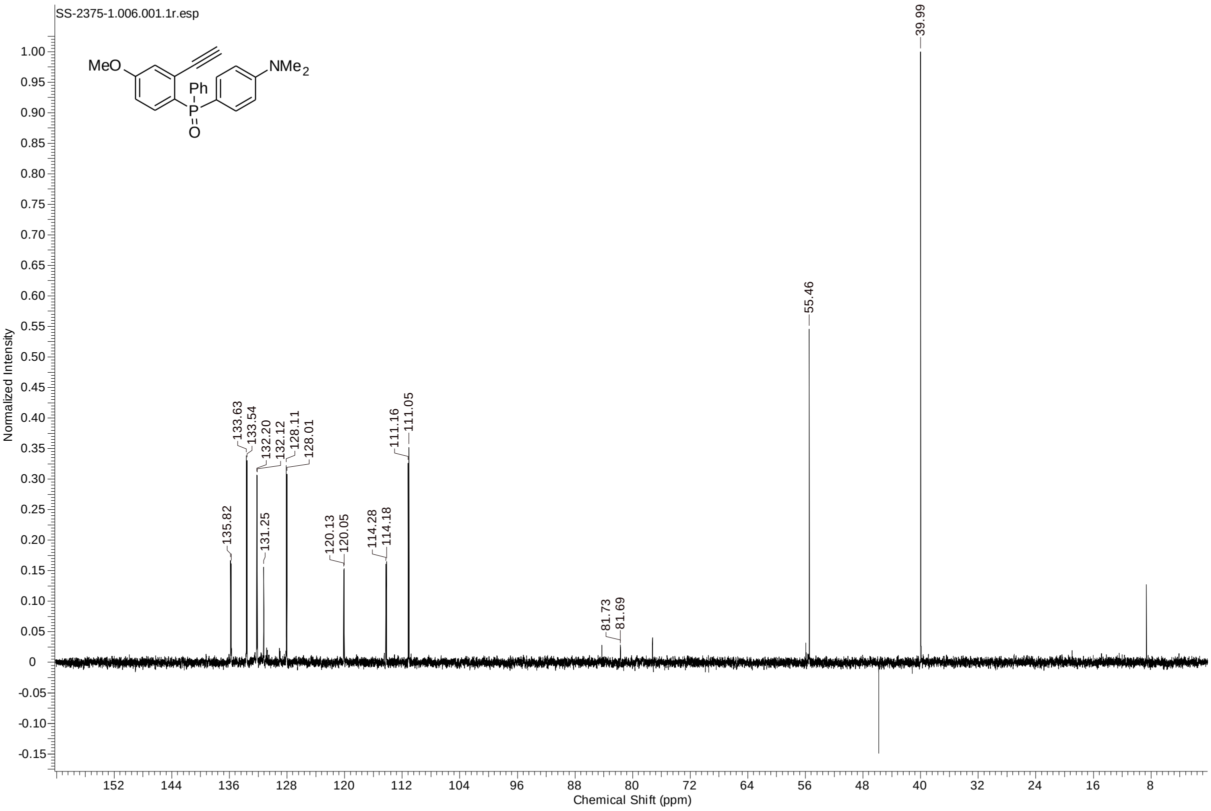

DEPT 135 NMR spectrum of (2-ethynyl-4-methoxyphenyl](4-dimethylaminophenyl)phenylphosphine oxide (**12k**) (125 MHz, $CDCl_3$)

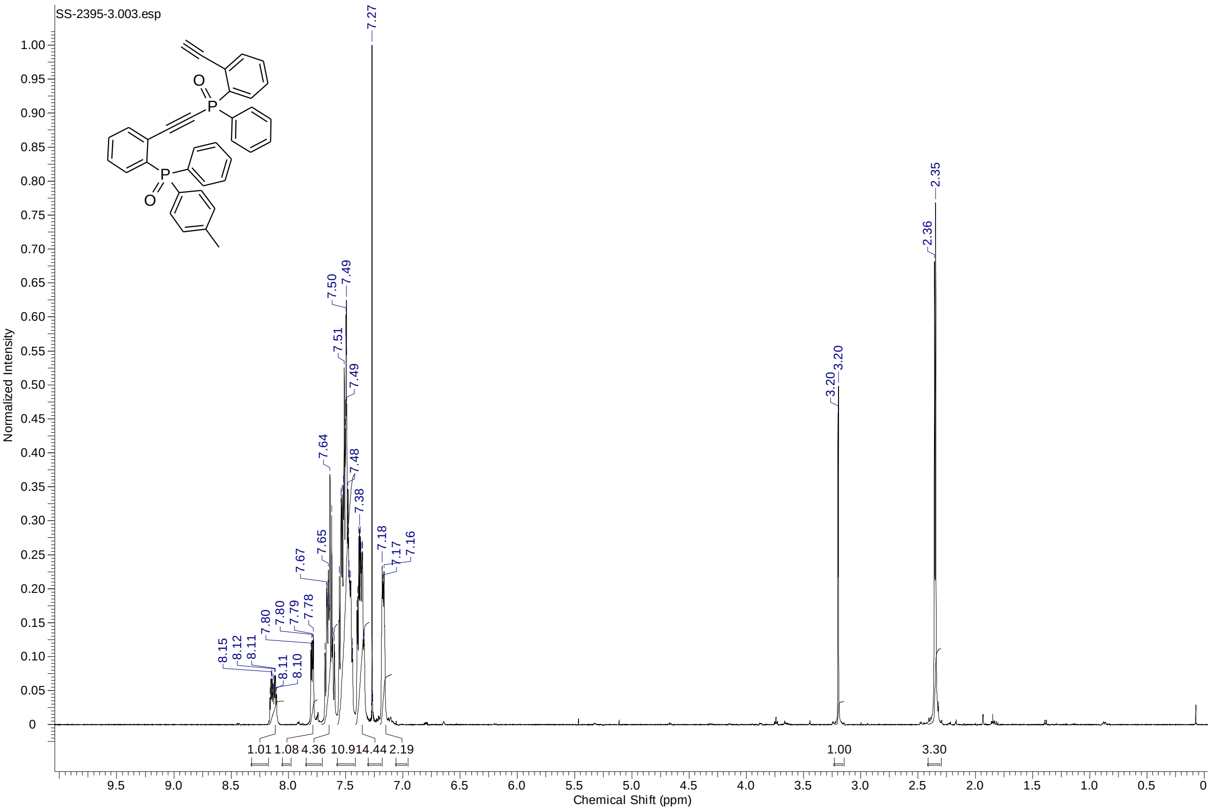

^{1}H NMR spectrum of (2-ethynylphenyl)(phenyl)((2-(phenyl(*p*-tolyl)phosphoryl)phenyl)ethynyl)-phosphine oxide (**9**) (500 MHz, $CDCl_3$)

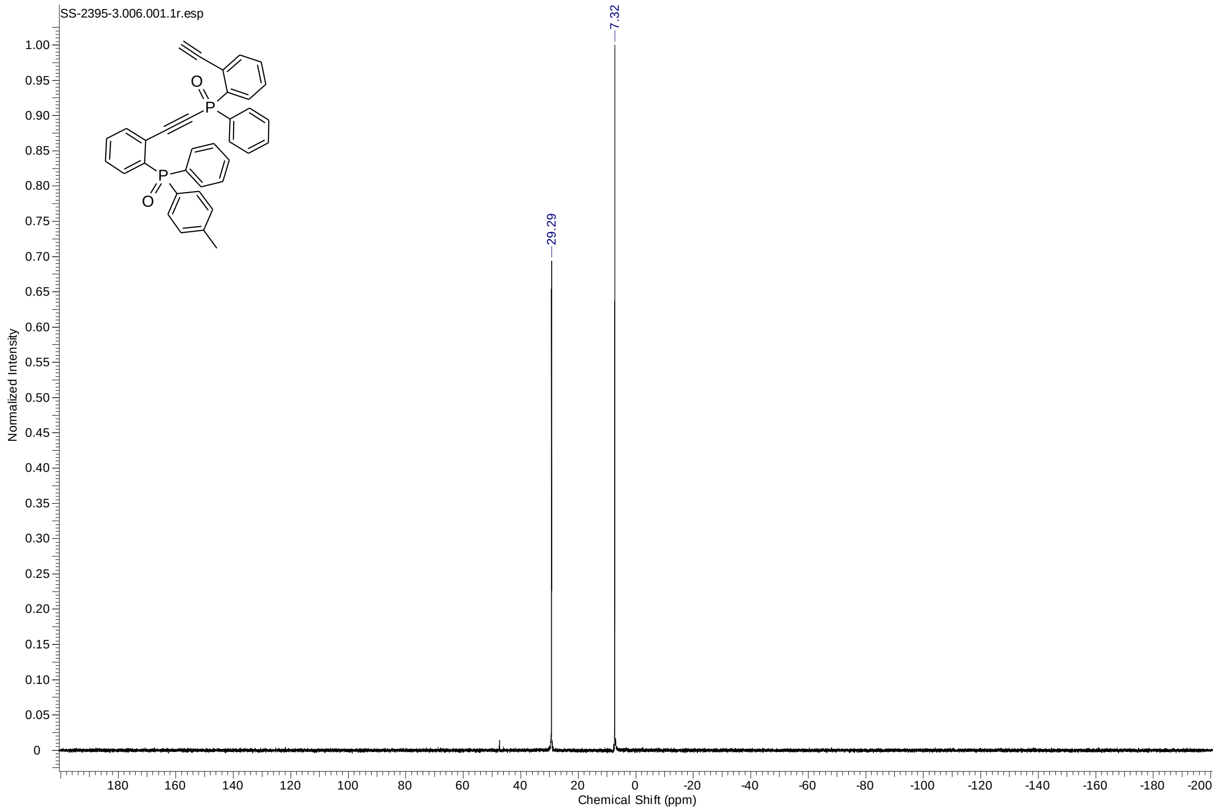

^{31}P NMR spectrum of (2-ethynylphenyl)(phenyl)((2-(phenyl(*p*-tolyl)phosphoryl)phenyl)ethynyl)-phosphine oxide (**9**) (202 MHz, $CDCl_3$)

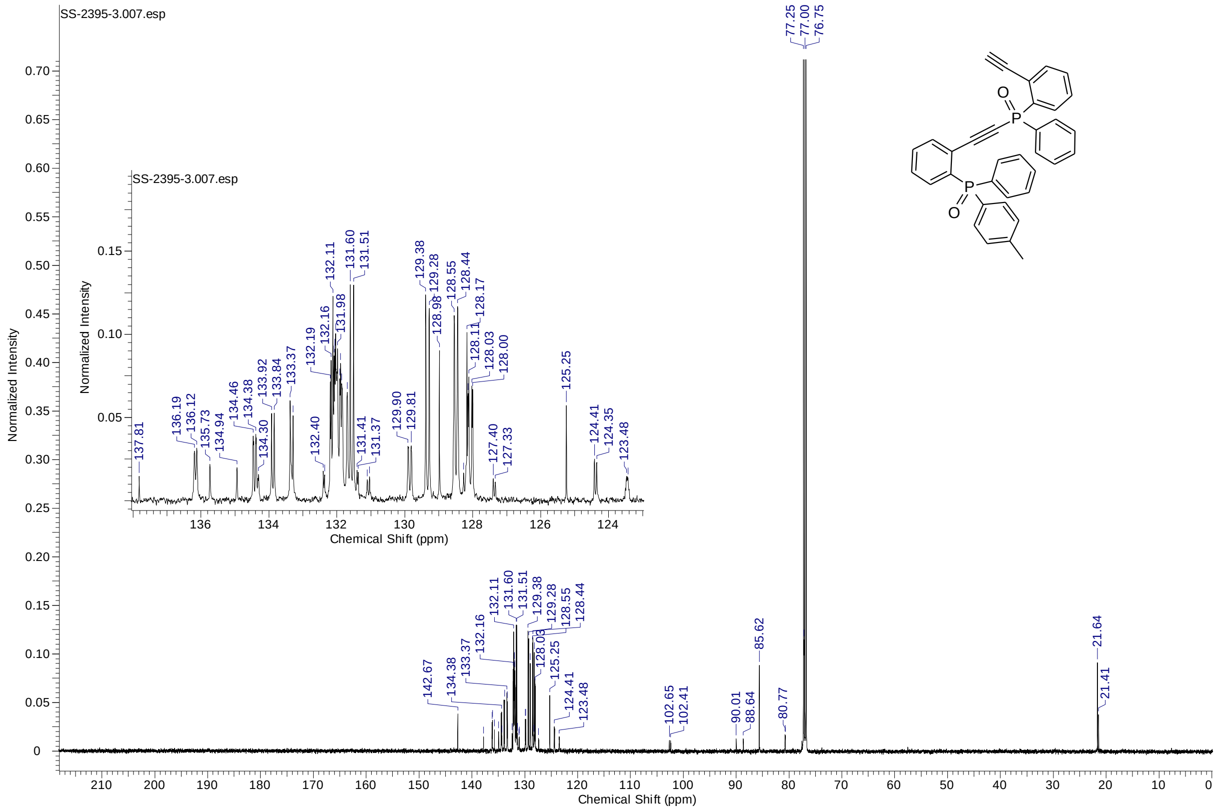

^{13}C NMR spectrum of (2-ethynylphenyl)(phenyl)((2-(phenyl(*p*-tolyl)phosphoryl)phenyl)ethynyl)-phosphine oxide (**9**) (125 MHz, $CDCl_3$)

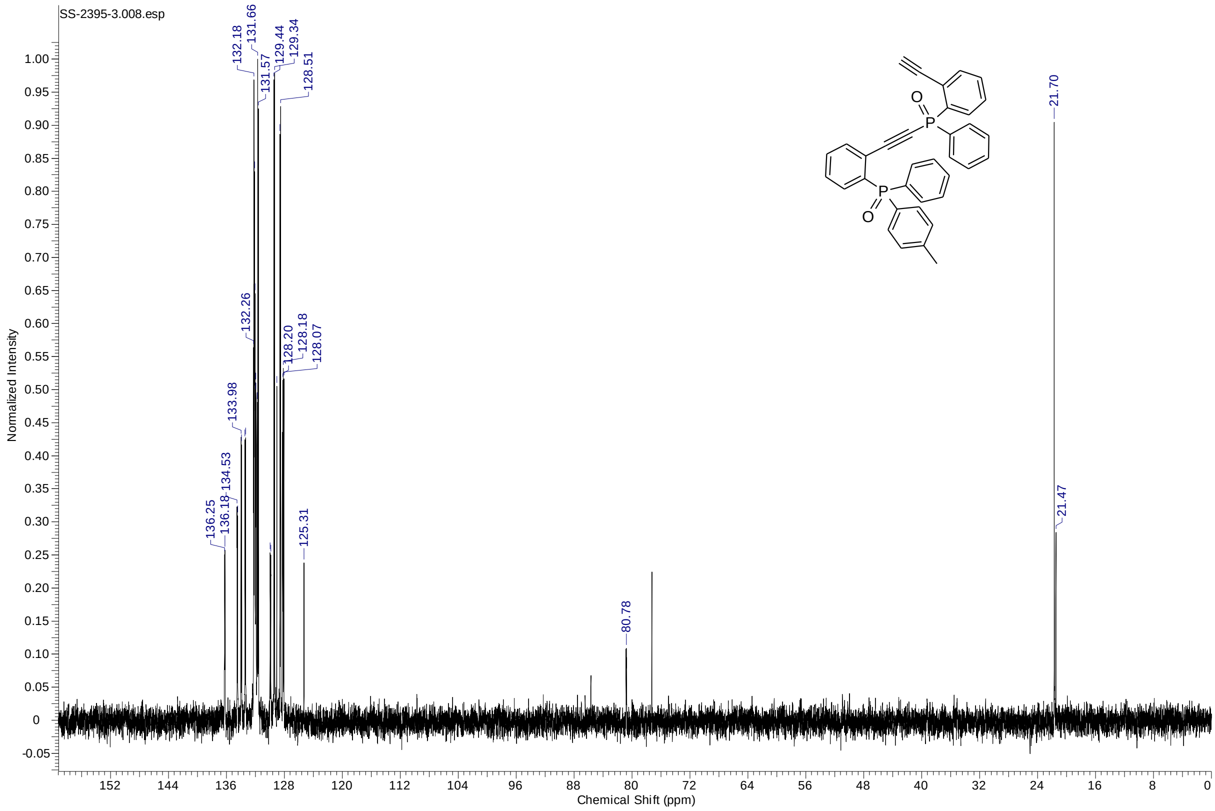

135 DEPT NMR spectrum of (2-ethynylphenyl)(phenyl)((2-(phenyl(*p*-tolyl)phosphoryl)phenyl)ethynyl)phosphine oxide (**9**) (125 MHz, $CDCl_3$)

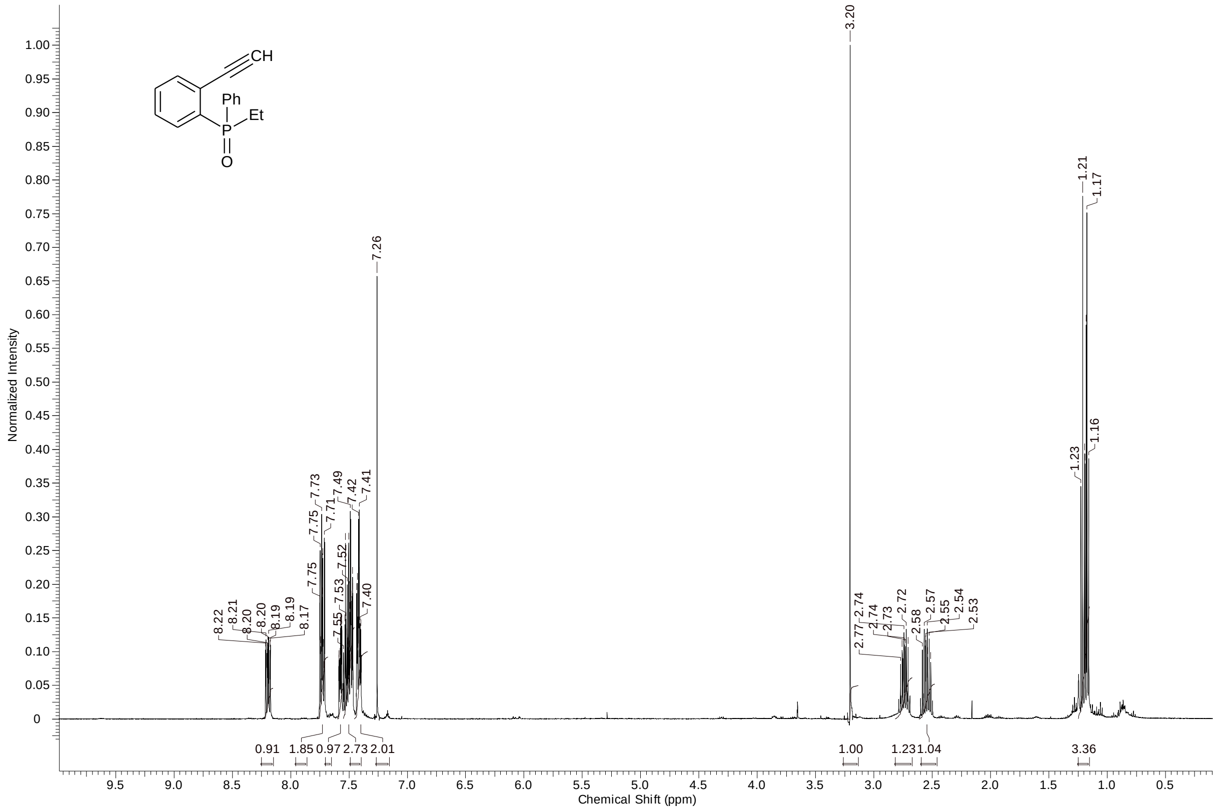

^{1}H NMR spectrum of ethyl(2-ethynylphenyl)phenylphosphine oxide (**13**) (500 MHz, $CDCl_3$)

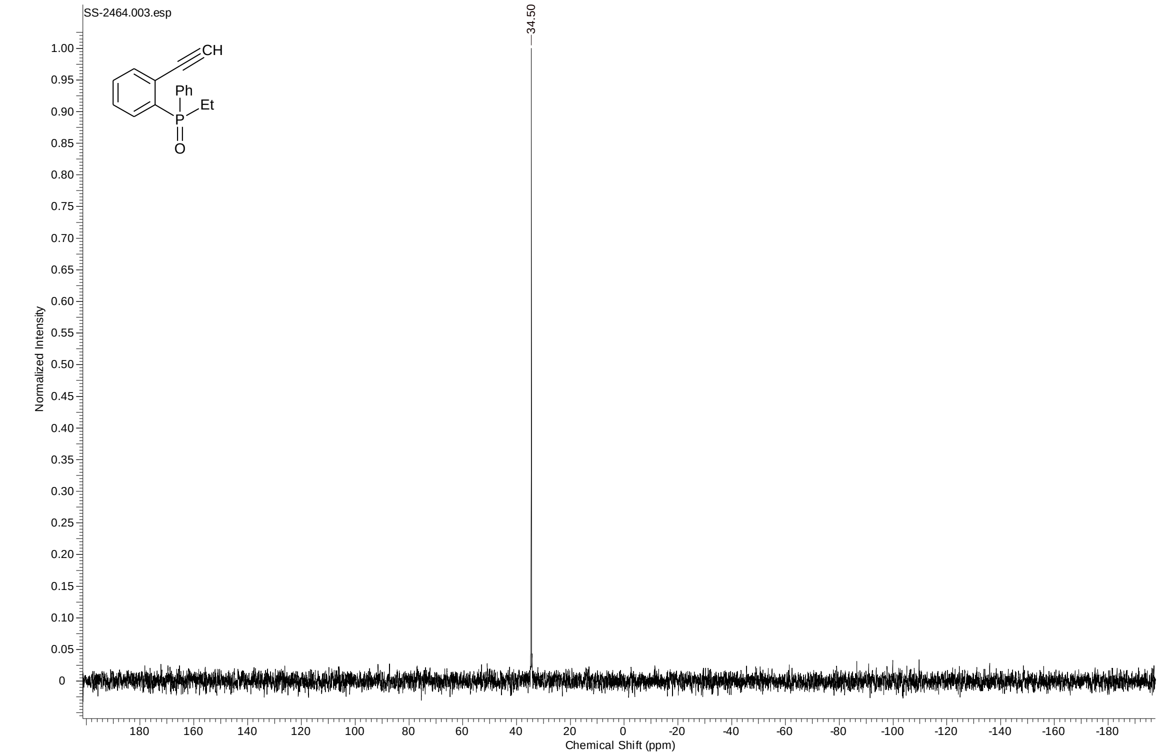

$^{31}P\{^1H\}$ NMR spectrum of ethyl(2-ethynylphenyl)phenylphosphine oxide (**13**) (202 MHz, $CDCl_3$)

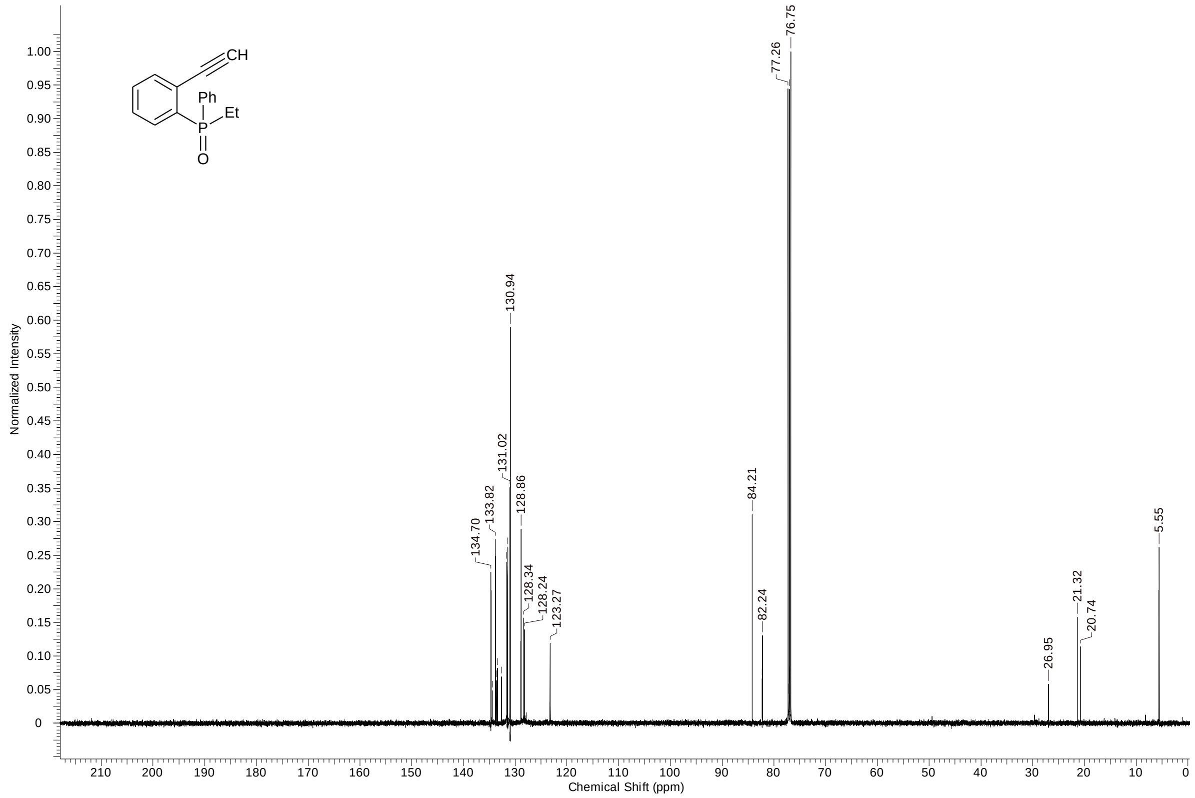

$^{13}C\{^1H\}$ NMR spectrum of ethyl(2-ethynylphenyl)phenylphosphine oxide (**13**) (125 MHz, $CDCl_3$)

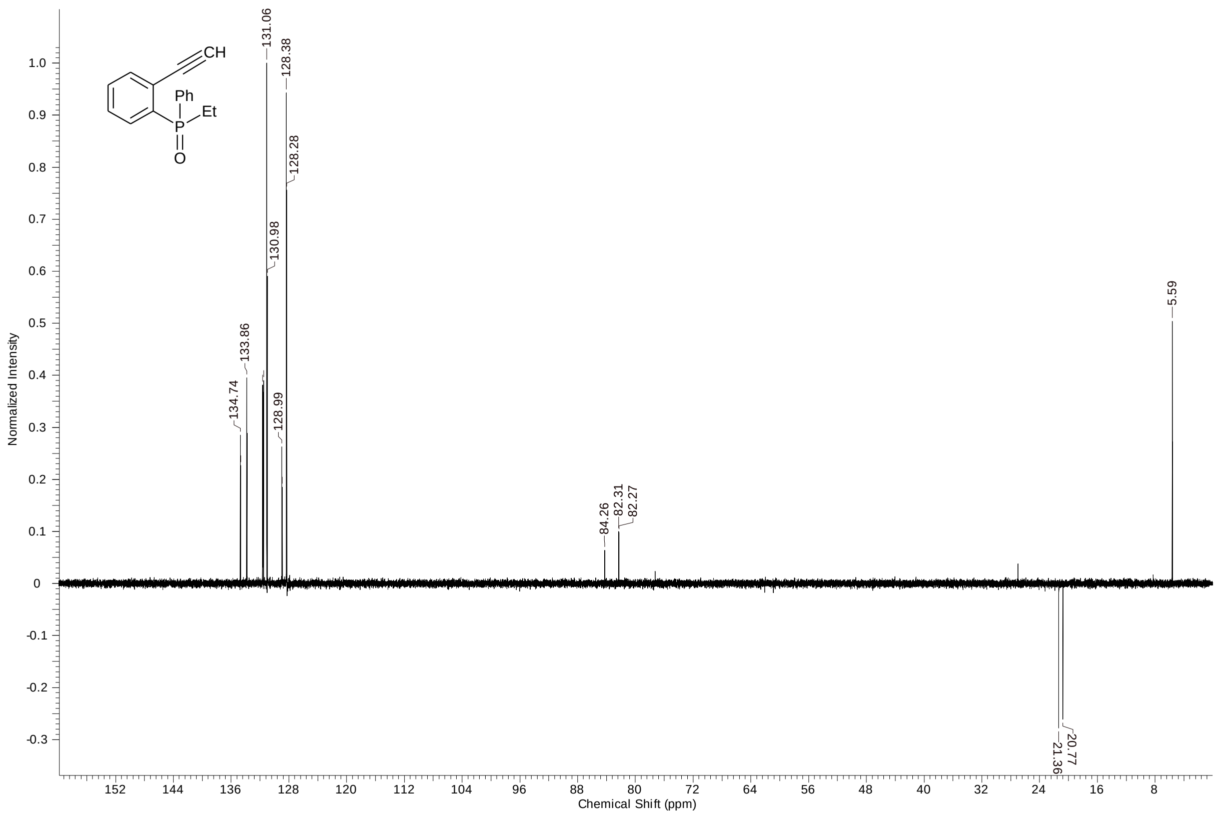

135 DEPT NMR spectrum of ethyl(2-ethynylphenyl)phenylphosphine oxide (**13**) (125 MHz, $CDCl_3$)

Table S1. Crystallographic data for **9**.

	9
Empirical formula	$C_{35}H_{26}O_2P_2$
Formula weight	540.5
Radiation source	Mo-*K*α
Crystallographic System	Triclinic
Space group	*P*-1
a [Å]	8.5228(4)
b [Å]	10.9844(7)
c [Å]	15.7451(9)
α [°]	98.256(5)
β [°]	99.880(4)
γ [°]	101.929(4)
V [$Å^3$]	1396.07(14)
Z	2
Calculated Density [$g\cdot cm^{-1}$]	1.286
T [K]	120(2)
μ [mm^{-1}]	1.187
Final R indices	0.0635
[*I*>2σ(I)]	0.1595
R indices (all data)	0.1313
[*I*>2σ(I)] (all data)	0.1874
CCDC	2094907

[1] Stankevič, M., Włodarczyk, A. *Tetrahedron*, **2013**, *69*, 1, 73-81
[2] Hu, W.; Li, E.-Q.; Duan, Z.; Mathey, F. *J. Org. Chem.* **2020**, 85, 14772-14778.
